# Vitamin C potentiates the killing of *Mycobacterium tuberculosis* by bedaquiline through metabolic disruption

**DOI:** 10.1128/mbio.01484-25

**Published:** 2025-06-25

**Authors:** Catherine Vilchèze, Saranathan Rajagopalan, Raja Sab Kalluru, Niaz Banaei, William R. Jacobs

**Affiliations:** 1 Department of Microbiology and Immunology, Albert Einstein College of Medicine 2006, Bronx, New York, USA; 2 Department of Pathology, Stanford University School of Medicine 10624 , Stanford, California, USA; NYU Langone Health, New York, New York, USA

**Keywords:** tuberculosis, bedaquiline, persistence, antibiotics, sterilization, metabolism, gene expression

## Abstract

Tuberculosis (TB), a disease caused by the bacterium *Mycobacterium tuberculosis* (*Mtb*), continues to pose a major global health threat, exacerbated by the emergence of drug-resistant strains and the lengthy treatment regimens required for effective management. Bedaquiline (BDQ), a key component in novel regimens for multidrug-resistant (MDR) TB, has demonstrated significant efficacy but is threatened by rising resistance. Our study investigates the potential of vitamin C to enhance BDQ’s activity and prevent resistance. We found that combining BDQ with vitamin C sterilized drug-susceptible and MDR *Mtb* cultures *in vitro* within 21 days, achieving a 6-log reduction in colony-forming units. This combination also enhanced *Mtb* killing in infected human macrophages and peripheral blood mononuclear cells. Transcriptomic analysis revealed that the BDQ/vitamin C combination induces widespread metabolic disruption in *Mtb*, characterized by upregulation of stress response and metal ion homeostasis genes and downregulation of energy metabolism and cell wall biosynthesis genes. Mechanistic studies implicated reactive oxygen species and disrupted copper homeostasis as contributing factors to the sterilization effect. These findings highlight the potential of using vitamin C as an adjunct therapy with BDQ, offering a promising strategy to enhance drug efficacy and mitigate emerging drug resistance during MDR-TB treatment.

**IMPORTANCE:**

Tuberculosis (TB) remains a major global health problem, especially as drug-resistant forms become more common and harder to treat. Bedaquiline is one of the most important new drugs for treating these resistant infections, but resistance to bedaquiline is also starting to appear. This study found that the combination of vitamin C and bedaquiline sterilizes *Mycobacterium tuberculosis* cultures *in vitro* while potentiating bedaquiline activity in infected human macrophage cells. The combination appears to overwhelm the bacteria by creating stress and disrupting essential functions, like energy production and metal balance. These results suggest that vitamin C, a safe and inexpensive supplement, could be used alongside existing drugs to make treatment faster and more effective while also helping to prevent resistance.

## INTRODUCTION

Tuberculosis (TB) remains a significant global health challenge. Despite the availability of a vaccine, BCG, and a plethora of medications to treat both drug-susceptible and drug-resistant TB cases, over a million individuals succumb to the disease annually (1). This persistent burden underscores the complexity of treatment. Patients with drug-susceptible TB require a minimum 6-month medication regimen, whereas those with drug-resistant TB may face treatment extending up to 2 years. Such prolonged therapies, often involving drugs with severe side effects (2–4), contribute to patient non-compliance, a significant factor in the development of drug resistance (2, 5, 6). The World Health Organization estimates that, in 2023, nearly half a million people were infected with multi-drug resistant (MDR)- or rifampicin (RIF)-resistant (RR) *Mycobacterium tuberculosis* (*Mtb*), the causative agent of TB, with treatment failure reported in approximately one-third of cases (1). MDR TB is characterized by resistance to isoniazid (INH) and RIF, the two first-line TB drugs. Resistance to second-line TB drugs, which are essential for the treatment of MDR-TB, exacerbates the challenge and highlights the urgent need for shorter, safer, and more effective TB treatments.

Recent clinical trials have shown promise in addressing these challenges. The combination of the newer TB drug bedaquiline (BDQ) with other repurposed or existing TB drugs yielded significant efficacy against RR, MDR, and extensively drug-resistant (XDR) TB (7–11). The Nix-TB and ZeNix clinical trials revealed that the 6-month BPaL regimen, comprising BDQ, pretomanid (PMD), and linezolid (LZD), achieved successful outcomes in 90% of patients with non-responsive MDR or XDR *Mtb* infections (9–11). The TB-PRACTECAL trial explored the addition of moxifloxacin (BPaLM) or clofazimine (BPaLC) to the BPaL regimen. These oral, 6-month regimens were found to be safer and more effective than the standard regimen, with BPaLM achieving a cure rate of 88% (77% for BPaLC, 86% for BPaL) (12). However, these advances are tempered by the emergence of BDQ resistance, reported both in patients previously treated with BDQ and in those without prior exposure (13–15).

BDQ resistance in clinical isolates arises from mutations in *atpE* (encoding the ATP synthase subunit C, the target of BDQ [16, 17]), *Rv0678* (encoding a transcriptional inhibitor of the efflux pump MmpS5-MmpL5), and *pepQ* (encoding an aminopeptidase) (18–22). Mutations in *Rv0678* and *pepQ* also confer resistance to clofazimine, a second-line TB drug used for the treatment of MDR-TB (23, 24), potentially reducing BDQ’s efficacy in patients with prior clofazimine exposure (25). Mutations in *Rv0678* were detected in MDR-*Mtb* clinical isolates from patients who had not been treated with BDQ or clofazimine, suggesting that resistance to BDQ can arise independently of direct drug exposure (26).

To combat the emergence of drug resistance, our previous studies demonstrated that combining TB drugs with compounds that stimulate *Mtb* respiration—such as vitamin C (27, 28), cysteine (27), or *N*-acetylcysteine (29)—can sterilize *Mtb* cultures and prevent the development of resistance. Building on this foundation, we investigated whether adding vitamin C to BDQ therapy could enhance BDQ activity and inhibit the emergence of BDQ-resistant *Mtb* mutants.

## RESULTS

### Vitamin C potentiates BDQ to sterilize *Mtb* cultures

**Fig 1:**
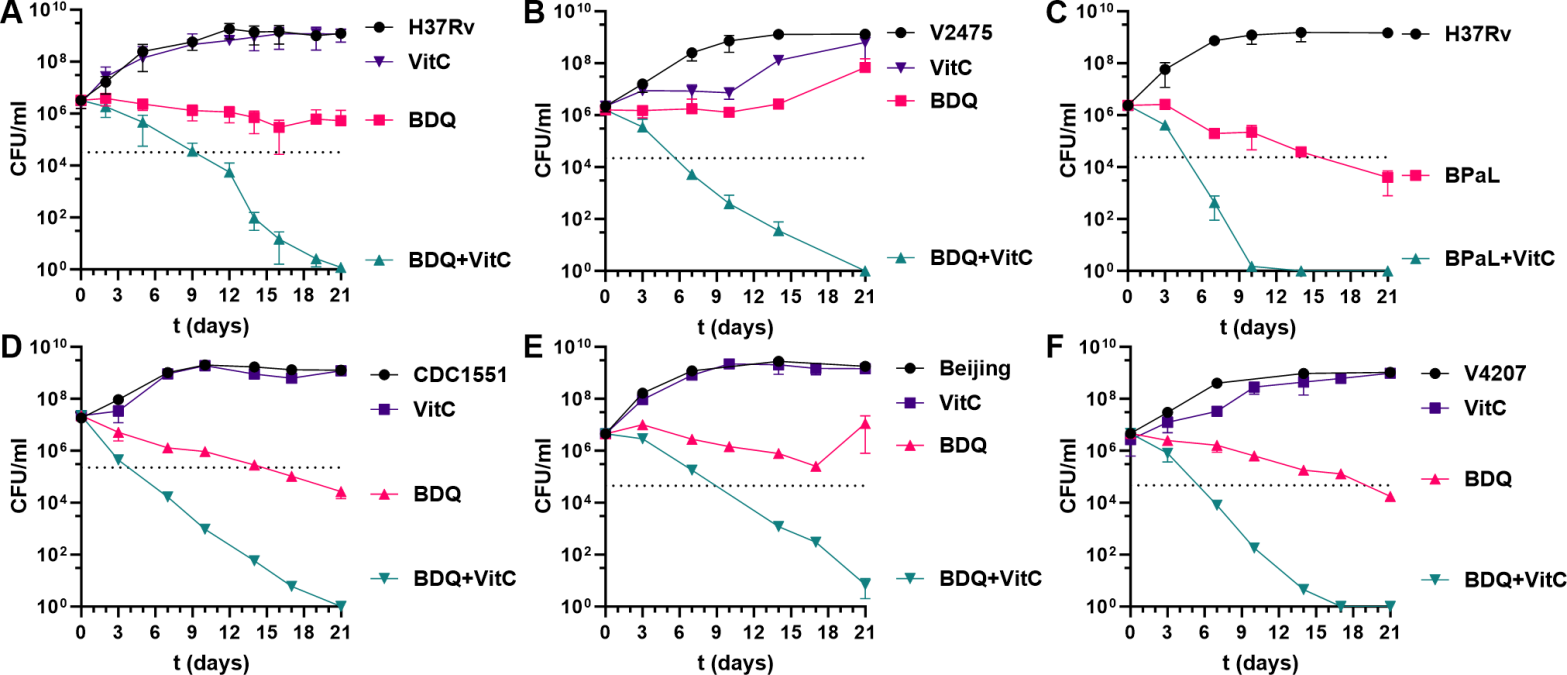
Adjunct activity of vitamin C against drug-susceptible and drug-resistant *Mtb* strains *in vitro***.** The drug-susceptible strains H37Rv (**A**), CDC1551 (**D**), Beijing (**E**), and V4207 (**F**), and the MDR strain V2475 (**B**) were treated with vitamin C (VitC; 1 mM), bedaquiline (BDQ; 0.9 µM), or the combination (BDQ [0.9 µM ] + VitC [1 mM]), added once at t = 0. Vitamin C (VitC; 1 mM) potentiates the BPaL regimen (BDQ; 0.9 µM; PMD 1.7 µM; LZD, 7.4 µM) against H37Rv (**C**). At the indicated time points, samples were taken, serially diluted, and plated to determine CFU/mL. Mean with standard deviation is plotted (*n* = 2-5). The dotted lines indicate a two-log reduction in CFUs, the benchmark for cidal activity.

The potentiation of BDQ by vitamin C was evaluated by measuring colony-forming units (CFUs) in *Mtb* H37Rv cultures treated with BDQ (0.9 µM, five times of BDQ minimum inhibitory concentration [MIC], Fig. S1A) alone or combined with vitamin C (1 mM) for 21 days. A single dose of each compound was added at the time of inoculation. BDQ treatment alone resulted in a modest 1-log reduction in CFUs over 21 days, whereas the addition of vitamin C to BDQ led to a 6-log CFUs reduction and sterilization in the culture (Fig. 1A). The combination of BDQ and vitamin C was not synergistic as the fractional inhibitory concentration index (FICI) value revealed an indifferent interaction between the two compounds (FICI of 1–2.125; Fig. S1B).

In BDQ-treated *Mtb* H37Rv cultures, growth was observed after 28 days, suggesting the emergence of BDQ-resistant mutants. Analysis of two colonies (BDQ-R1 and BDQ-R2) isolated after 28 days of BDQ treatment showed low-level BDQ resistance (MIC 1.4 µM, Fig. S1A), and Sanger sequencing revealed a mutation in *Rv0678* (t131c, L44P). By contrast, no growth was observed in BDQ/vitamin C-treated cultures that were maintained shaking at 37°C for 3 months, indicating that vitamin C adjunct therapy could avert the emergence of BDQ-resistant mutants.

The combination BDQ/vitamin C was tested against two BDQ-resistant *Mtb* strains: the clinical MDR strain V2475 (30) originating from Kwa-Zulu Natal, South Africa, which carries a M139I mutation in *Rv0678* (MIC 0.36 µM, Fig. S1A), and the H37Rv-derived BDQ-R2. The BDQ/vitamin C combination sterilized the MDR *Mtb* V2475 culture within 3 weeks, achieving a 6-log reduction in CFUs (Fig. 1B). By contrast, BDQ alone was bacteriostatic with no CFU reduction (Fig. 1B). Notably, vitamin C alone transiently suppressed V2475 growth during the initial 10 days of treatment. Similarly, the laboratory BDQ-R2 mutant viability was reduced by 5- to 6-log when treated with BDQ (3.6–7.2 µM, 2.5–5 times the MIC) and vitamin C (Fig. S2).

Vitamin C was further tested with the BPaL regimen (BDQ, 0.9 µM; PMD 1.7 µM; LZD, 7.4 µM) and individual components (5× MIC). While BPaL alone resulted in a three-log reduction in CFUs in 21 days, adding vitamin C sterilized the culture within 10 days, representing a 6-log CFU reduction (Fig. 1C). The combination of vitamin C with PMD or LZD alone also sterilized the *Mtb* culture (6-log CFU reduction) within 3 weeks (Fig. S3A and B). Interestingly, LZD was cidal on its own (Fig. S3A), but PMD static activity was transient, with culture growth resuming after 7–10 days (Fig. S3B). In addition, vitamin C potentiated the other TB drugs used in the all-oral TB drug regimens for the treatment of DR-TB, such as moxifloxacin, delamanid, and clofazimine, achieving 6-log CFU reductions when combined (Fig. S3C through E).

Testing the BDQ/vitamin C combination across other BDQ-susceptible *Mtb* strains (CDC1551, Beijing HN878, and V4207 (30), MIC shown in Fig. S1) confirmed its sterilizing potential within 21 days, achieving 6-log reductions in all cases (Fig. 1D through F). Notably, BDQ alone was the least active against H37Rv and Beijing, while BDQ’s activity was cidal against CDC1551 and V4207.

We initially used 1 mM vitamin C as this concentration had no cidal activity against *Mtb* and yet potentiated the activity of the first-line TB drugs INH and RIF (28, 31). Lowering the vitamin C concentration (from 1 mM to 0.1 mM) gradually reduced the potentiation of BDQ by vitamin C (Fig. S4A). Reducing the BDQ concentration (0.18 µM [MIC level] or 0.45 µM) led to culture growth after 7 and 16 days, respectively. The addition of vitamin C to these BDQ-treated *Mtb* cultures remained cidal, but a longer treatment time was required to achieve the 6-log CFU reduction and sterilization (Fig. S4B).

### Vitamin C potentiates BDQ in human macrophages

**Fig 2:**
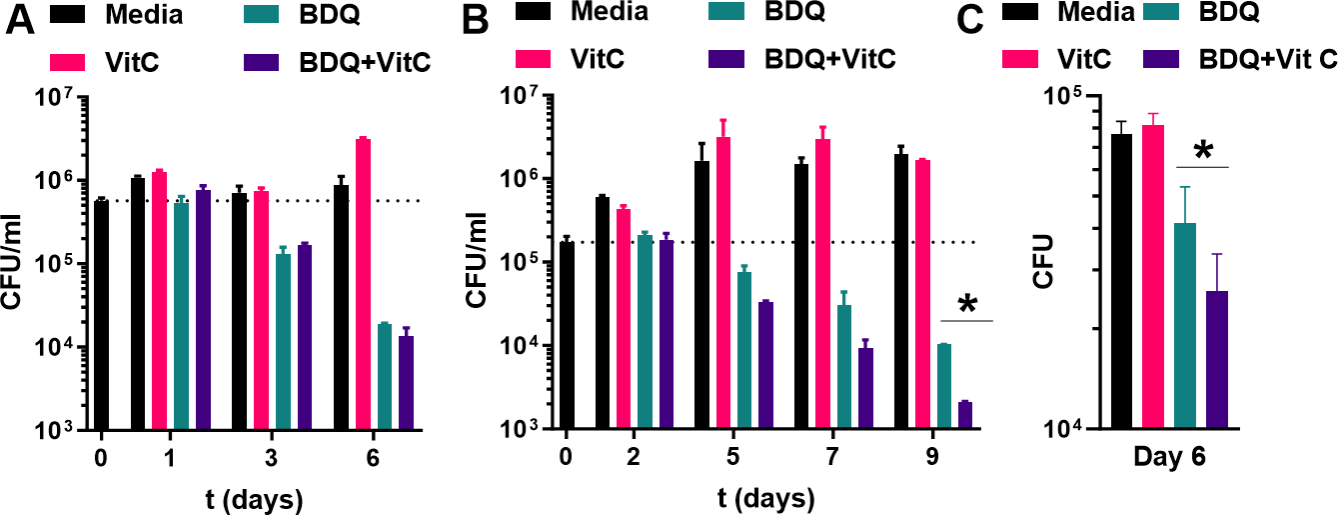
Adjunct activity of vitamin C in macrophages. (**A**) The murine macrophage cell line J774 was infected with *Mtb* H37Rv (MOI of 4) for 4 h prior to treatment with vitamin C (VitC; 1 mM), bedaquiline (BDQ; 0.9 µM), or the combination BDQ (0.9 µM) + VitC (1 mM) (*n* = 2). (**B**) The human macrophage cell line THP1 was infected with *Mtb* H37Rv (MOI of 2) for 4 h prior to treatment with VitC (1 mM), BDQ (0.9 µM), or the combination BDQ (0.9 µM) + VitC (1 mM) (*n* = 2). (**C**) PBMCs were infected with *Mtb* mc^2^6020 (MOI 5–10) for 3 h prior to treatment with VitC (1 mM), BDQ (2.7 µM), or the combination BDQ (2.7 µM) + VitC (1 mM) (*n* = 8-9). At the indicated time points, macrophage cell lines were lysed. The lysates were serially diluted and plated to determine CFU/mL. * denotes significant differences (*P* < 0.05).

The BDQ/vitamin C combination was evaluated in mouse and human macrophage cell lines. In *Mtb*-infected mouse macrophage cell line J774, BDQ and BDQ/vitamin C treatments reduced the *Mtb* bacterial count by 1.5 logs and 1.6 logs, respectively, by day 6 (Fig. 2A). In *Mtb*-infected human macrophage cell line THP1, BDQ reduced CFUs by ~1.2 log, whereas the BDQ/vitamin C combination achieved a ~1.9 log CFU reduction by day 9 (Fig. 2B).

We extended this analysis to human peripheral blood mononuclear cells (PBMCs) infected with *Mtb*. While BDQ alone showed modest activity (~0.3 log CFU reduction), the addition of vitamin C to BDQ significantly enhanced *Mtb* killing, achieving a 0.5 log CFU reduction after 6 days (Fig. 2C). This potentiation was lower compared to the effects of vitamin C on first-line (INH/RIF, ~0.7 log) and second-line (moxifloxacin/amikacin/clofazimine, ~1.1 logs) TB drug combinations (Fig. S5). Importantly, the increased efficacy of BDQ/vitamin C was most relevant in human macrophages.

### The BDQ/vitamin C treatment modulates the expression of genes involved in iron and copper metabolism, uptake, transport, and storage

To investigate the genetic mechanism underlying the sterilization effect of vitamin C combined with BDQ, we performed RNA-seq on *Mtb* cultures treated with vitamin C, BDQ, and BDQ/vitamin C combination for 3 days since differentiation of *Mtb* viability during BDQ and BDQ/vitamin C treatment occurred between day 2 and day 5 (Fig. 1A). We observed contrasting gene expression profiles between BDQ and the BDQ/vitamin C combination.

### Distinct gene expression patterns

Vitamin C alone had a minimal impact on *Mtb* growth and gene expression (Fig. 1A, Table 1). A modest upregulation of oxidative stress response genes was observed (*katG* and *trxC*, 4- to 5-fold), while genes involved in the methylcitrate cycle (*prpD,* 20-fold) and fatty acid biosynthesis (*fabD*, 8-fold) were notably suppressed (Table S1).

**TABLE 1:** Classification of the genes induced or repressed in *Mtb* H37Rv treated with vitamin C (VitC, 1 mM), bedaquiline (BDQ, 0.9 µM), or the combination of BDQ/Vitamin C for 3 days (the percentage relative to the total number of genes in the *Mtb* genome in each category is indicated in parenthesis)

Functional category	VitC		BDQ		BDQ-VitC	
Log_2_ fold change	>2	<−2	>2	<−2	>2	<−2
Virulence, detoxification, adaptation	2 (0.4)	3 (0.6)	4 (0.7)	8 (1.5)	21 (3.9)	28 (5.2)
Lipid metabolism	0 (0.0)	3 (1.3)	9 (3.8)	12 (5.0)	27 (11.3)	30 (12.6)
Information pathways	0 (0.0)	0 (0.0)	18 (7.7)	2 (0.9)	14 (6.0)	49 (21.0)
Cell wall and cell processes	2 (0.3)	4 (0.5)	14 (1.7)	11 (1.5)	51 (6.8)	74 (9.8)
Insertion seqs and phages	0 (0.0)	0 (0.0)	0 (0.0)	7 (7.9)	24 (27.0)	1 (1.1)
PE/PPE	0 (0.0)	0 (0.0)	3 (1.9)	3 (1.9)	48 (30.2)	12 (7.5)
Intermediary metabolism and respiration	3 (0.3)	3 (0.3)	25 (2.7)	13 (1.2)	63 (6.0)	59 (5.6)
Unknown	0 (0.0)	0 (0.0)	0 (0.0)	1 (7.1)	0 (0.0)	3 (21.4)
Regulatory proteins	0 (0.0)	2 (1.0)	4 (2.1)	4 (2.1)	17 (8.9)	21 (10.9)
Conserved hypotheticals	4 (0.4)	6 (0.6)	22 (2.0)	24 (2.2)	70 (6.4)	108 (9.9)

In BDQ treatment, 99 genes upregulated fourfold or more were genes encoding ribosomal proteins, lipid biosynthesis, tryptophan biosynthesis, and NADH dehydrogenase type 1, which were downregulated or not differentially expressed in the BDQ/vitamin C treatment. The most upregulated genes (11- to 12-fold) in BDQ treatment encoded the PPE20/PE15 complex (Table S1), secreted by the ESX3 secretion system (32) and shown to function as a Ca^2+^ importer (33), which could be a response to ATP depletion since high Ca^2+^ concentration increases ATP levels (33).

**Fig 3:**
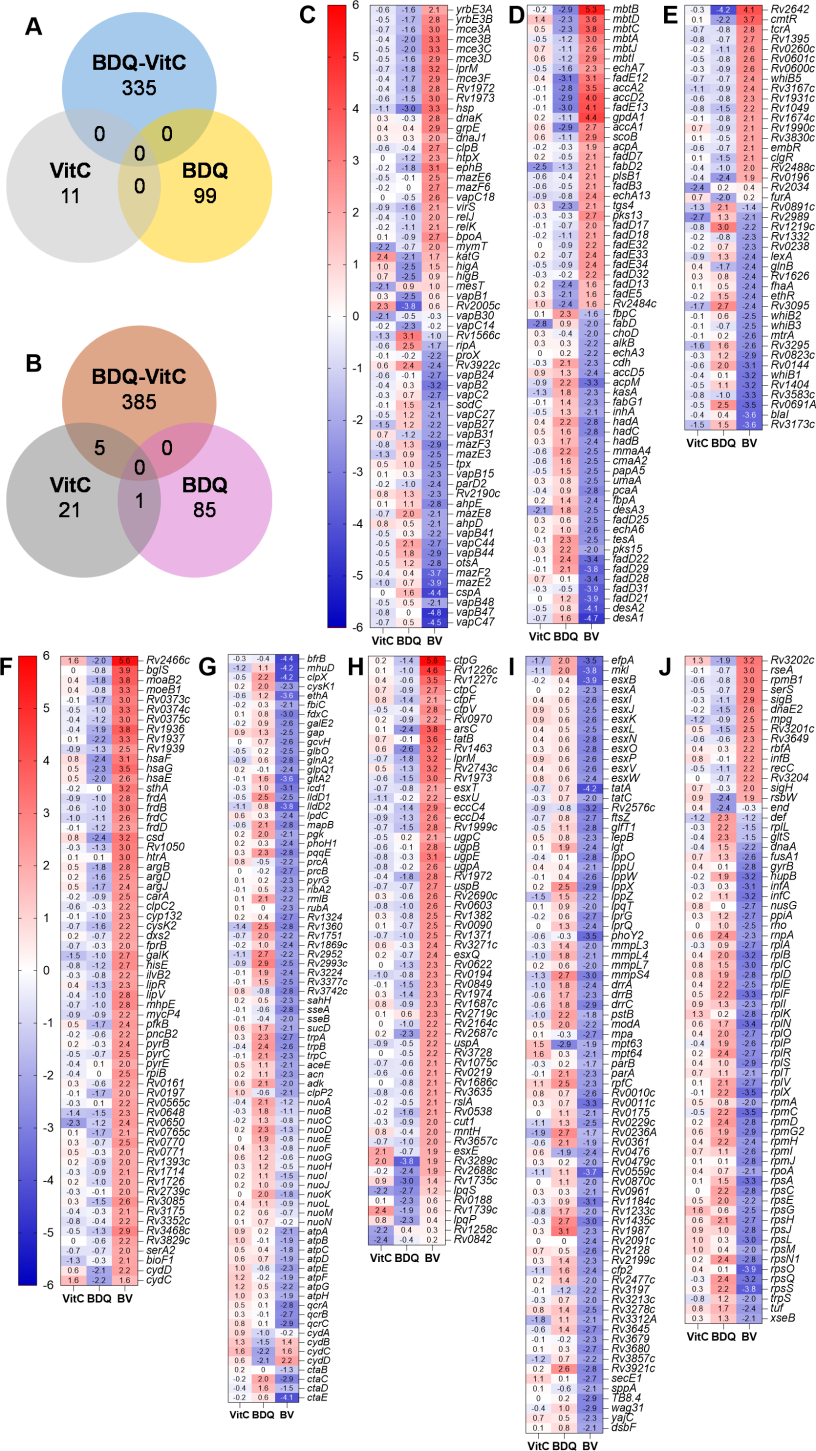
Differentially regulated genes in *Mtb* treated with vitamin C, BDQ, or the combination BDQ/Vitamin C. Number of genes upregulated (**A**) or downregulated (**B**) in *Mtb* H37Rv treated with vitamin C (VitC; 1 mM), bedaquiline (BDQ; 0.9 µM), or the combination (BV; BDQ [0.9 µM] + VitC [1 mM]) for 3 days. The genes differentially regulated were classified based on Mycobrowser website: (C) Virulence, detoxification, and adaptation; (**D**) lipid metabolism, (**E**) regulatory proteins, (**F**) intermediary metabolism and respiration showing upregulated genes in the BDQ/vitamin C combination, (**G**) intermediary metabolism and respiration showing downregulated genes in the BDQ/vitamin C combination, (H) cell wall and cell processes showing upregulated genes in the BDQ/vitamin C combination, (I) cell wall and cell processes showing downregulated genes in the BDQ/vitamin C combination, and (J) information pathways. The color scale represents log_2_ fold change.

In BDQ/vitamin C treatment, the number of differentially regulated genes was three times higher than in BDQ treatment (Fig. 3A and B; Table 1). Notably, the genes upregulated or downregulated in the BDQ/vitamin C treatment were often inversely regulated under BDQ treatment alone (Fig. 3C through J). The most upregulated gene in the BDQ/vitamin C combination was *ctpG* (*Rv1992c,* >50-fold) encoding a metal cation transporter (Fig. 3H and Table S1). Operon partners, *Rv1993c* and *Rv1994c* (*cmtR*) (34), were similarly upregulated 21-fold and 13-fold, respectively (Fig. 3E; Table S1). Other metal cation transporter genes such as *ctpC* (*Rv3270*)*, ctpF* (*Rv1997*), and *ctpV* (*Rv0969*) were also upregulated (4-fold to 7-fold) (Fig. 3H). The second most upregulated gene in BDQ/vitamin C treatment was *Rv2466c* (48-fold, Fig. 3F)*,* which encodes a mycothiol-dependent reductase enzyme critical for oxidative stress survival (35). The other upregulated genes in BDQ/vitamin C treatment encoded proteins involved in the iron-binding mycobactin biosynthesis (MbtABCDIJ, Fig. 3D), heme iron utilization (Esx4, Fig. 3H), stress responses (KatG, DbsA, DnaK, ClpB, DnaJ1, Hsp, and HtpX, Fig. 3C), copper stresses (CadI, Rv2642, BglS, and MymT, Fig. 3C, E and F), arginine biosynthesis (ArgBDJ, Fig. 3F), fumarate reductase (FrdABCD, Fig. 3F), lipid degradation (FadDE, Fig. 3D), lipid catabolism (HsaEFG, Fig. 3F), lipid transport (UgpABCE, Fig. 3H), and sugar transport (UspAB, Fig. 3H).

RNA-seq analysis also revealed a broad downregulation of genes essential for *Mtb* survival, including those involved in energy metabolism (Fig. 3G), protein synthesis (Fig. 3J), and cell wall biosynthesis (Fig. 3D), upon BDQ/vitamin C treatment.

Transcriptomic profiling of *Mtb* treated with BDQ and vitamin C revealed differential expression of genes associated with iron and copper metabolism, suggesting potential mechanisms involving oxidative stress or metal toxicity. To further explore these mechanisms, we tested the involvement of reactive oxygen species (ROS) and metal ions in *Mtb* sterilization by the BDQ/vitamin C combination.

### The potentiation of BDQ by vitamin C involves a multifactorial effect

**Fig 4:**
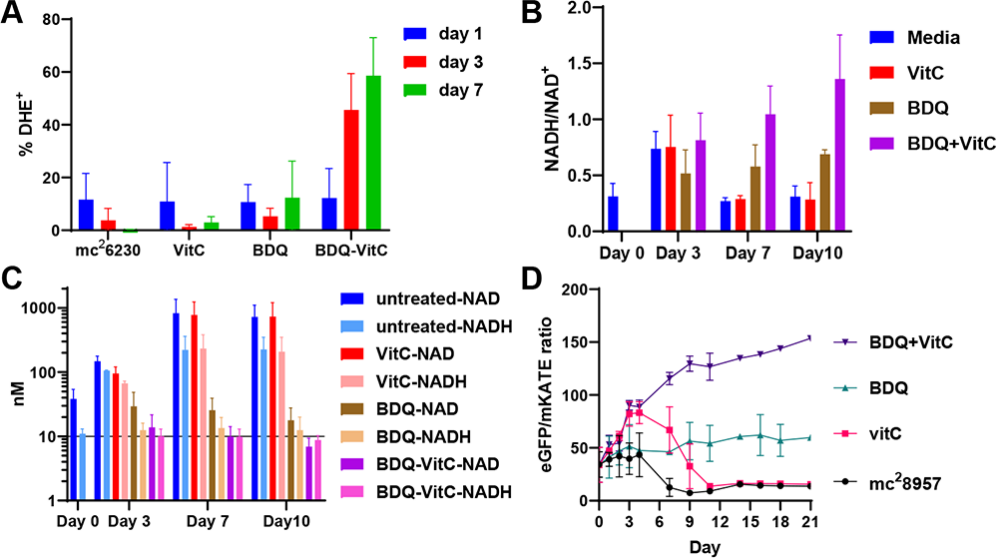
Effect of the combination of BDQ and vitamin C on ROS production, NADH/NAD^+^ level, and labile heme consumption. (**A**) *Mtb* mc^2^6230 was treated with vitamin C (VitC; 1 mM), bedaquiline (BDQ; 0.9 µM), or the combination (BDQ [0.9 µM] + VitC [1 mM]). At the indicated time, samples were taken, stained with dihydroethidium, and analyzed by flow cytometry. (**B, C**) *Mtb* mc^2^6230 was treated with vitamin C (VitC; 1 mM), bedaquiline (BDQ; 0.9 µM), or the combination (BDQ [0.9 µM ] + VitC [1 mM]). At the indicated time, samples were taken, and NADH and NAD^+^ concentrations were measured using the cycling assay described in Materials and Methods. (**D**) mc^2^8957 (mc^2^7901 expressing the HS1-M7A heme sensor) was treated with vitamin C (1 mM), BDQ (0.9 µM), or the combination BDQ (0.9 µM) + vitamin C (1 mM) for up to 21 days. At the indicated times, samples were taken, and fluorescence was measured (eGFP, excitation 480 nm and emission 510 nm; mKATE2, excitation 580 nm and emission 620 nm). Mean with standard deviation is plotted *(n* = 2-4).

RNA-seq analysis indicated upregulation of genes involved in the oxidative stress response in *Mtb* treated with BDQ and vitamin C. Given that vitamin C can react with metal ions like iron and copper to produce reactive oxygen species (ROS), including superoxide, hydrogen peroxide, and hydroxyl radicals (36), we hypothesized that ROS generation might contribute to the bactericidal activity of this combination therapy. To test this hypothesis, we used dihydroethidium to detect superoxide levels in *Mtb* cultures treated with BDQ, vitamin C, or both. We observed an increase in superoxide levels after 3 days of treatment with the BDQ/vitamin C combination, while neither treatment alone induced a similar increase (Fig. 4A).

To investigate the potential impact of oxidative stress on cellular metabolism, we measured the NADH/NAD^+^ redox ratio, a key indicator of metabolic state, which could further imply a repercussion in the cell’s metabolism. The BDQ/vitamin C combination, as well as BDQ alone, disrupted this redox balance after 7 days of treatment (Fig. 4B). While BDQ treatment shifted the redox environment toward a more reduced redox environment, the addition of vitamin C to BDQ seemed to further exacerbate this effect (Fig. 4C).

The upregulation of the mycobactin gene cluster (*mbtABCDEFGIJ,* 3- to 39-fold) and arginine biosynthesis, as well as downregulation of *bfrb* (22-fold) involved in iron storage, suggested that *Mtb* was deprived of iron in the BDQ/vitamin C treatment (37, 38). To investigate this further, we measured intracellular-free ferrous and ferric iron levels but did not observe significant differences between treatment groups (Fig. S6). However, RNA-seq analysis of the BDQ-vitamin C treatment revealed an 18-fold repression of *mhuD* (*Rv3592*), a gene encoding the *Mtb* heme oxygenase (Fig. 3G). Heme oxygenase degrades heme, releasing ferrous ions and potentially contributing to oxidative stress. To directly assess heme levels, we utilized a ratiometric heme biosensor composed of a heme-binding domain of cytochrome b_562_ conjugated to red (mKATE, internal standard) and green (eGFP) fluorescent proteins (39). In the presence of heme, heme binding by the b_562_ domain quenches green fluorescence. The level of labile heme, measured by the ratio eGFP/mKATE, decreased in *Mtb* cultures co-treated with vitamin C and BDQ over the course of 21 days (Fig. 4D). This heme consumption was not specific to the BDQ/vitamin C treatment, as we also observed the same increase in the eGFP/mKATE ratio in *Mtb* co-treated with vitamin C and PMD, LZD, or BPaL (Fig. S7). Vitamin C triggers a transient decrease in heme levels, which was sustained when a drug such as BDQ was added to the vitamin C treatment, suggesting that *Mtb* may be actively preventing further heme degradation through the repression of *mhuD*.

**Fig 5:**
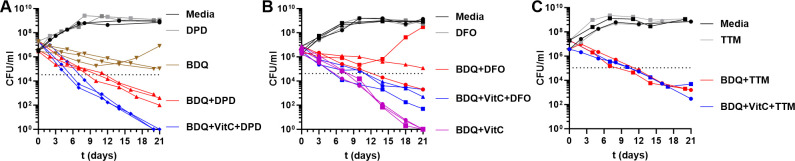
Effect of chelators on the antimycobacterial activity of BDQ and the combination BDQ and vitamin C. *Mtb* H37Rv was treated with vitamin C (VitC; 1 mM), bedaquiline (BDQ; 0.9 µM), the combination (BDQ [0.9 µM] + VitC [1 mM]), and the following ion chelator: dipyridyl (**A**, DPD, 0.1 mM), deferoxamine (**B**, DFO, 0.18 mM), or tetrathiomolybdate (**C**, TTM 0.01 mM), added once at t = 0. At the indicated time points, samples were taken, serially diluted, and plated to determine CFU/mL. Mean with standard deviation is plotted (*n* = 2-3). The dotted lines indicate a two-log reduction in CFUs.

RNA-seq analysis identified the involvement of metal ions, particularly iron and copper, in the BDQ/vitamin C sterilization phenotype. To further explore the role of these metal ions, we tested the impact of metal chelators on the bactericidal activity of the BDQ/vitamin C combination. We used dipyridyl (DPD), a ferrous iron chelator (32), deferoxamine (DFO), a ferric ion chelator (40), and tetrathiomolybdate (TTM), a copper chelator (41). The ferrous ion chelator DPD did not alter the activity of the combination BDQ/vitamin C (the cultures were sterilized within 21 days). However, DPD increased the inhibitory effect of BDQ alone, resulting in at least a 4-log decrease in CFUs after 21 days (Fig. 5A). Notably, the emergence of BDQ-resistant mutants was not observed with prolonged incubation (over 2 months) at 37°C with shaking. By contrast, DFO reduced the bactericidal activity of the BDQ/vitamin C combination but had an inconsistent impact on BDQ treatment alone (Fig. 5B). In three independent experiments, the combination of BDQ and DFO was either bactericidal or bacteriostatic or led to the rapid emergence of BDQ-resistant mutants (BDQ MIC for four colonies isolated after 28 days of treatment ranged between 1.44 and 2.88 µM). The copper chelator TTM eliminated the potentiation of BDQ by vitamin C and increased the bactericidal activity of BDQ alone (Fig. 5C). There was no difference in the killing of *Mtb* by BDQ or BDQ/vitamin C when TTM was present, suggesting an opposite role for copper in BDQ activity and vitamin C potentiation.

Copper homeostasis is complex but critical for *Mtb* survival (42, 43). To test the involvement of copper homeostasis in the sterilization of *Mtb* by the combination of BDQ and vitamin C, we utilized *Mtb* mutants with deletions in the copper-sensitive operon *Rv0967–Rv0969*. This operon, which is highly upregulated in the presence of copper (44), includes a cuprous ion-binding repressor (Rv0967) and a copper exporter (Rv0969) (45). In our RNA-seq data set, the operon *Rv0967-Rv0970* was induced twofold to sevenfold. The deletion of either the entire operon or the repressor alone had limited effect on the potentiation of BDQ by vitamin C, leading to a slower sterilization process and no effect on BDQ inhibitory activity (Fig. S8). This result did not reproduce the effect of copper chelation, illustrating the complexity of copper response in *Mtb.* Deletion of the copper-sensitive operon might not compensate for other pathways involved in copper regulation, such as the metallothionein MymT, the copper transport protein MctB, the multicopper oxidases MmcO, or the regulated copper repressor RicR (46).

RNA-seq analysis indicated a shift in lipid metabolism in *Mtb* treated with BDQ and vitamin C, with upregulation of genes involved in lipid degradation and catabolism and downregulation of genes involved in lipid biosynthesis and envelope biogenesis. We extracted and analyzed the lipid composition in *Mtb* treated with BDQ or BDQ/vitamin C and found that polar and apolar lipids were greatly reduced in the BDQ/vitamin C treatment (Fig. S9). Lastly, to determine whether the addition of vitamin C to BDQ treatment would further exacerbate the ATP depletion caused by BDQ, we measured intracellular ATP levels in *Mtb* treated with BDQ, vitamin C, or the combination and found that the combination of vitamin C to BDQ did not significantly increase ATP depletion in *Mtb* (Fig. S10).

## DISCUSSION

The addition of vitamin C to BDQ leads to the sterilization of *Mtb* cultures *in vitro* and enhances *Mtb* killing in human macrophages. The mechanism underlying this sterilization appears to be complex and multifaceted, involving ROS, metal ion homeostasis (iron, copper), and metabolic disruption, with no single factor solely responsible for the observed sterilization phenotype.

### Mechanistic insights

Our previous studies demonstrated that high concentrations of vitamin C (4 mM) alone could sterilize *Mtb* cultures *in vitro*, with this effect being dependent on the presence of high levels of ferrous ions, driving the Fenton and Haber-Weiss reactions to produce ROS (31). Initially, we hypothesized that the sterilization of *Mtb* cultures by lower doses of vitamin C in combination with BDQ might operate through a similar mechanism. However, our observations challenged this hypothesis. Notably, the addition of 1 mM vitamin C to BDQ treatment did not significantly increase intracellular ferrous ion concentration. Furthermore, the presence of iron chelators (ferric or ferrous) only marginally delayed, if at all, the sterilization of *Mtb* by the BDQ and vitamin C combination. These findings suggest that the potentiation of BDQ by low concentrations of vitamin C involves mechanisms distinct from the iron-mediated ROS generation observed with high concentrations of vitamin C alone.

An earlier study demonstrated that the ferritin BfrB levels increased only in the presence of high iron concentrations in *Mtb*, implying a role for BfrB in protecting *Mtb* from iron toxicity (47). The observed upregulation of the mycobactin cluster (*mbtABCDEFGIJ*) and arginine biosynthesis genes, coupled with the downregulation of the iron storage protein gene *bfrB,* strongly suggested that *Mtb* was experiencing iron deprivation during BDQ and vitamin C treatment (37, 38, 47). However, the genes encoding the Esx3 secretion pathway, essential for iron acquisition (32, 48), were not differentially expressed, and *mmpl4* and *mmpS4,* which export carboxymycobactin for ferrous ion uptake, were repressed (fourfold and sevenfold, respectively). Instead, genes associated with the Esx4 secretion system (*Rv3444c–Rv3450c*) required for heme uptake (49) were upregulated fourfold to sevenfold. Utilizing a heme biosensor, we observed that vitamin C adjunct therapy induced heme consumption. Heme levels steadily decreased in *Mtb* co-treated with vitamin C and BDQ, PMD, or BPaL. A transient decrease in heme levels was also observed in *Mtb* treated with vitamin C alone. This observation, coupled with the concurrent downregulation (18-fold) of the *Mtb* heme oxygenase *mhuD,* further supports heme degradation, which could also release ferrous ions for ROS production.

In the context of oxidative stress, BDQ and vitamin C treatment resulted in elevated superoxide levels, likely leading to damage to cellular components, including DNA, lipids, and proteins, through oxidative modifications. In addition, the transcriptional analysis of the BDQ/vitamin C combination highlighted the upregulation of the SUF operon responsible for assembly or repair of Fe-S clusters (Rv1461–Rv1466 [50]), which may be caused by both ROS generation (51) and iron deprivation (37). ROS can disrupt iron-sulfur (Fe-S) clusters, essential for numerous cellular processes (52). Furthermore, the observed induction of metal ion transporters (*ctpG, ctpV, ctpC,* and *ctpF)* in the BDQ/vitamin C treatment may further exacerbate Fe-S cluster disruption by displacing iron, thereby impacting proteins dependent on Fe-S clusters for activity.

### Metabolic disruption

Prior research has shown that BDQ exhibited bactericidal activity against *Mtb* only at high concentrations (30–300× MIC) (53). Furthermore, this cidal activity typically became apparent only after 2–3 days of treatment. This delay in cidal activity was likely due to the bacterium’s ability to maintain ATP levels through glycolysis, as suggested by Koul et al. (53). In a subsequent study, Mackenzie et al. demonstrated that BDQ-mediated ATP inhibition sensitizes *Mtb* to perturbations in glycolytic pathways, and that BDQ treatment rapidly sterilized *Mtb* mutants with specific defects in the glycolysis pathway *in vitro*. These findings led the authors conclude that combining inhibitors of substrate-level phosphorylation (glycolysis) and oxidative phosphorylation (BDQ) can effectively sterilize *Mtb* (54). Furthermore, an elevated cellular NADH/NAD^+^ ratio can disrupt glycolysis, leading to reduced ATP availability for *Mtb* (53). Our findings indicate that combining vitamin C with BDQ treatment caused a depletion in NAD^+^ levels in *Mtb*, thereby increasing the NADH/NAD^+^ ratio. This NAD^+^ depletion could potentially disrupt glycolytic processes that are dependent on NAD^+^ as a cofactor. We propose that a mechanism similar to that described by Mackenzie et al. contributes to *Mtb* sterilization by the BDQ/vitamin C combination, involving the synergistic interplay between glycolytic disruption caused by an elevated NADH/NAD^+^ ratio and BDQ-mediated ATP depletion. The combined effect of these metabolic perturbations overwhelms *Mtb*’s energy reserves, ultimately leading to bacterial death.

### Role of metal ion chelation

We observed enhanced bactericidal activity when BDQ was combined with either the copper chelator TTM or the ferrous ion chelator DPD. Interestingly, a similar effect between BDQ and the ferric ion chelator DFO was observed in BCG-infected primary human macrophages (55). However, we observed variable results with DFO in *Mtb* cultures treated with BDQ *in vitro* (Fig. 5B). Despite this discrepancy, both sets of findings suggest that iron and copper chelators may have the potential to enhance BDQ therapy. We propose that another facet of the vitamin C mechanism is the deregulation of metal ion homeostasis, thereby enhancing BDQ’s bactericidal activity.

### Conclusion

The potential of vitamin C as an adjunctive therapy to BDQ for the treatment of MDR-TB hinges on achieving pharmacologically relevant concentrations. Intravenous (iv) vitamin C can achieve plasma concentrations of 1 mM (56) or more (57). As all-oral regimens with no injectables are preferred for TB patients, iv injection of vitamin C may not be suitable. By contrast, oral administration of vitamin C (200–400 mg daily) leads to lower saturated plasma concentrations (approximately 70 µM), but with vitamin C concentration in immune cells up to 40 times higher (0.5–3 mM) than in plasma in men and women (58, 59). Similarly, in rat pneumocytes, vitamin C concentration in alveolar macrophages was found to be 30 times higher (3 mM) than in the media (60). The enhanced vitamin C concentration in immune cells and, in particular, alveolar macrophages compared to plasma vitamin C concentration might be enough to enhance BDQ treatment, suggesting that supplementing TB drug treatment with vitamin C oral doses should be tested in TB patients. This study focused on the effect of vitamin C on drug-treated *Mtb*, but vitamin C was shown to have effects on the host. Given the inflammatory nature of TB and the potential for lung tissue damage, supplementation with vitamin C in TB drug regimens may offer additional benefits due to its anti-inflammatory effects and role in collagen synthesis, potentially aiding in lung tissue repair.

Our study provides compelling evidence that the BDQ/vitamin C combination sterilizes *Mtb* through multifactorial mechanisms involving ROS, metal ion homeostasis, and energy metabolism disruptions. Identifying compounds that replicate vitamin C’s mechanisms of action could lead to novel adjunctive therapies. Vitamin C or compounds that mimic vitamin C mode of action offer a promising strategy to enhance the efficacy of BDQ and other existing TB drugs while mitigating drug resistance development in TB treatment.

## MATERIALS AND METHODS

### Bacterial strains, media, and materials

The mycobacterial strains and cell lines were obtained from laboratory stocks. The culture medium to grow the mycobacterial strains was Middlebrook 7H9 (Difco, Sparks, MD) supplemented with 10% (vol/vol) OADC enrichment, 0.2% (vol/vol) glycerol, 0.1 mM sodium propionate, and 0.05% (vol/vol) tyloxapol. Plating was done on Middlebrook 7H10 (Difco, Sparks, MD) supplemented with 10% (vol/vol) OADC enrichment and 0.2% (vol/vol) glycerol. For safety reasons, some experiments used the auxotrophic *Mtb* strains mc^2^6020, mc^2^6230, or mc^2^7901, which are biosafety level 2-safe laboratory *Mtb* H37Rv strains (61). These strains require the following supplements for growth: mc^2^6020 (H37Rv Δ*panCD*Δ*lysA*), D-pantothenate (24 mg/L), and lysine (1 mg/L); mc^2^6230 (H37Rv ΔRD1 Δ*panCD*), D-pantothenate (24 mg/l); and mc^2^7901 (H37Rv Δ*panCD*Δ*leuCD*Δ*metA*), D-pantothenate (24 mg/L), leucine (50 mg/l), and methionine (50 mg/L). Chemicals and biologicals were obtained from Sigma (St Louis, MO) or Thermo Fisher Scientific (Waltham, MA) unless otherwise stated.

### Viability of *Mtb*

The *Mtb* strains were grown shaking at 37°C to the exponential phase (optical density at 600 nm [OD_600_], ~1) and diluted 1/200. Vitamin C and/or drugs were added to the diluted *Mtb* cultures at day 0. Samples were taken at indicated times, diluted with Dulbecco phosphate-buffered saline (DPBS), and plated for CFUs (plating media described above). The plates were incubated at 37°C for up to 2 months. Undiluted, drug-treated samples were plated on Middlebrook 7H10 medium supplemented with 10% (vol/vol) OADC enrichment, 0.2% (vol/vol) glycerol, and 0.4% carbon to eliminate drug carryover.

### NAD^+^/NADH

Cultures of mc^2^6230 (OD_600_, ~0.1) were treated with vitamin C (1 mM) and/or BDQ (0.9 µM) for up to 10 days. At the indicated times, samples were taken for NAD^+^ and NADH measurement (5 mL sample for each). The samples were spun down, and the supernatant was removed. For the NAD^+^ extraction, the pellet was resuspended in 0.2 N HCl (0.5 mL), incubated at 55°C for 10 min, cooled to 0°C, and neutralized with 0.1 N NaOH (0.5 mL). Proteins were precipitated by keeping the suspension on ice for 10 min. After centrifugation, the supernatant was kept on ice for the duration of the measurement. For the NADH extraction, a similar protocol was followed except that the cell pellet was resuspended in 0.2 N NaOH (0.5 mL) and neutralized with 0.1 N HCl (0.5 mL). NAD^+^ and NADH measurements were done using the cycling assay described in (62, 63) in 96-well plate where each well contained 0.01 mL of yeast type II alcohol dehydrogenase (five units), diluted sample (0.09 mL) and reagent mix (0.1 mL, composed of an equal volume of 1.0 M bicine (pH 8.0), 100% ethanol, 40 mM EDTA (pH 8.0), 4.2 mM 3-[4,5-dimethylthiazol-2-yl]−2,5-diphenyltetrazolium bromide and 16.6 mM phenazine methosulfate). The plate was read at 570 nm for 10 min. NAD^+^ and NADH concentrations were determined based on standard curves.

### RNAseq transcriptome analysis

Cultures of H37Rv (OD_600_, ~0.1; 50 mL each) were treated with vitamin C (1 mM) and/or BDQ (0.9 µM) for 3 days. On day 0 (for untreated control) and day 3, the cultures were spun down and resuspended in Qiagen RNA Protect (Qiagen, Germantown, MD; 1 mL) overnight. The suspensions were spun down. The cell pellets were treated with TRIzol (Invitrogen; 1 mL) and lysed using a Fast-Prep apparatus (MP Biomedicals). Total RNA from each sample was extracted using the Direct-zol RNA Miniprep plus kit (Zymo Research, Irvine, CA) according to the manufacturer’s protocol. Quantification of total RNA was performed using Qubit RNA BR Assay Kits (ThermoFischer). Zymo-Seq Ribofree Total RNA Library kit (Zymo Research) was used for cDNA synthesis, ribosomal RNA depletion, and library preparation; 250 ng of input RNA was used. The genomic libraries were sequenced using the Illumina NextSeq 2000 platform using the P1 reagents kit (150 PE). Sequencing was performed in duplicates for both control and treated samples.

Transcriptome data were analyzed using Geneious Prime (v 2024.0.2). Raw reads were trimmed with the BBDuk trimmer (Biomatters Ltd.), and sequence data quality was assessed using FastQC (v0.11.9). Trimmed reads were then mapped to the H37Rv genome (NC000962) using Bowtie2 (v 7.2.2). The number of reads that were aligned to the annotated genes, reads per kilobase per million (RPKM), transcripts per million (TPM), and fragments per kilobase per million mapped reads (FPKM) were counted. The expression level was calculated, and differential expression was assessed using DeSeq2, and expression clustering was performed using log2 fold change. The gene expression in the vitamin C-, BDQ-, and BDQ/vitamin C-treated samples at day 3 was compared to the expression in untreated samples at day 0. A *P*-adj value of less than 0.05 defined significantly differentially expressed genes between untreated and treated samples.

### Minimum inhibitory concentration

*Mtb* cultures were grown to log phase (OD_600_, ~0.8–1) and diluted 1/200 in Middlebrook 7H9 media (see above). The perimeter wells of a sterile 96-well plate were filled with 0.2 mL PBS. Twofold serial dilutions of BDQ in Middlebrook 7H9 media were performed for a final volume of 0.1 mL in each inner well, with the last column used as a no-drug control. The diluted *Mtb* cultures (0.1 mL) were added to each inner well. The plates were incubated, shaking, at 37°C for up to 2 weeks. Optical density was read on a FLUOstar Omega Microplate reader (BMG Labtech). The MIC was determined as the lowest concentration of drug that inhibited *Mtb* growth by 95%.

### Checkerboard assay

*Mtb* H7Rv culture was grown to log phase (OD_600_, ~0.8–1) and diluted 1/200 in Middlebrook 7H9 media (see above). The perimeter wells of a sterile 96-well plate were filled with 0.2 mL PBS, and the inner wells were filled with 0.1 mL of Middlebrook 7H9 media. BDQ (0.64 µg) was added to the B2 well, while wells C2 to G2 received 0.32 µg of BDQ in 0.1 mL of Middlebrook 7H9 media. Twofold serial dilutions were performed from column 2 to 10. In row B2 to B11, 0.28 mg of vitamin C in 0.1 mL of Middlebrook 7H9 media was added. Twofold serial dilutions were performed from row B to row F. 0.1 mL of the diluted *Mtb* H37Rv culture was added to each inner well. The plates were incubated, shaking, at 37°C for up to 2 weeks. Optical density was read on a FLUOstar Omega Microplate reader (BMG Labtech). Wells B2 to F10 contain twofold serial dilutions of both BDQ and vitamin C. Row G and column 11 contain no vitamin C or BDQ, respectively, allowing for the determination of BDQ MIC and vitamin C MIC. FICI values were determined following the formula FICI = FIC BDQ + FIC vitamin C where FIC BDQ = BDQ MIC in the presence of vitamin C/BDQ MIC alone and FIC vitamin C = vitamin C MIC in the presence of BDQ/vitamin C MIC alone.

### Iron concentration

Cultures of mc^2^6230 (OD_600_, ~0.1) were treated with vitamin C (1 mM) and/or BDQ (0.9 µM) for up to 10 days. At the indicated times, a sample (10 mL) was taken and spun down. Supernatant was removed and kept on ice. The cell pellet was washed twice in cold PBS and resuspended in 50 mM NaOH (1.1 mL). The suspension was lysed using a Fast-Prep machine and then spun down again. The supernatant was removed and kept on ice. The supernatants (from the culture or the cell lysate, 0.1 mL) were mixed with 10 mM HCl (0.1 mL) and incubated at 60°C for 2 hours. The samples were cooled to 0°C and treated with an iron detection reagent (0.03 mL). Free ferrous and ferric ion concentrations were measured using the ferrozine colorimetric assay (64). For ferrous ion concentration determination, the iron detection reagent was composed of 192.7 mg of ammonium acetate, 0.05 mL ferrozine solution [64 mg (3-(2-pyridyl)−5,6-bis(phenyl sulfonic acid)−1,2,4-triazine) in 1 mL water], 0.05 mL of neocuproine solution (31.8 mg/mL water), and water for a final volume of 1 mL. For free iron concentration (ferrous and ferric ions), the iron detection reagent was composed of 578.7 mg ammonium acetate, 528.4 mg ascorbic acid, 0.15 mL ferrozine solution (see above), 0.15 mL neocuproine solution (31.8 mg/mL H_2_O), and water for a final volume of 3 mL. The iron detection reagent was left to react for 30 min. The samples were transferred to a 96-well plate, which was read at 550 nm. The iron concentrations were determined based on standard curves and normalized to protein concentrations using the Pierce BCA Protein Assay kit.

### Labile heme consumption assay

The plasmid 159170 expressing the cytosolic heme reporter hHS1-M7A was purchased from Addgene (Watertown, MA). HS1-M7A heme sensor was cloned into a replicative mycobacterial vector using HiFi DNA Assembly cloning (NEB, Ipswich, MA, primers listed in Table S2). The resulting plasmid pYUB3143, which uses the promoter pG13 to express HS1-M7A, was introduced into mc^2^7901 by electroporation to generate the strain mc^2^8957. mc^2^8957 was grown in Middlebrook 7H9-OADC-glycerol-propionate-tyloxapol supplemented with D-pantothenate, leucine, methionine, and kanamycin (20 mg/L) and treated with vitamin C, TB drugs (BDQ, LZD, PMD, or BPaL), or the combination TB drug + vitamin C for up to 21 days. At the indicated times, samples (0.2 mL) were taken, and fluorescence was measured (eGFP, excitation 480 nm and emission 510 nm; mKATE2, excitation 580 nm and emission 620 nm).

### Flow cytometry analysis of superoxide levels

Cultures of mc^2^6230 (OD_600_, ~0.1) were treated with vitamin C (1 mM) and/or BDQ (0.9 µM) for up to 7 days. At the indicated times, samples (1 mL) were taken, washed twice in DPBS, and resuspended in DPBS (0.3 mL). Washed cells (0.1 mL) were mixed with dihydroethidium (5 µM, 0.1 mL), incubated at 37°C in the dark for 30 min, and analyzed by flow cytometry on a BioRad S3e using the yellow-green 561 nm laser. For each sample, 50,000 events were collected.

### Construction of H37Rv Δ*Rv0967* and H37Rv Δ*Rv0967–Rv0969*

*Rv0967* and the operon *Rv0967–0969* were deleted from *Mtb* H37Rv using the specialized transduction protocol (65). Briefly, *Rv0967* and *Rv0967-0969* left and right flanks were amplified by PCR (see primers in Table S2) and cloned into *Van91I-*cut pYUB1471 (see restriction enzymes used for cloning in Table S2). The resulting cosmids were sequenced, pacI-cut, ligated into pacI-digested shuttle phasmid phAE159, packaged *in vitro* using GigapackII (Stratagene, La Jolla, CA), and transduced into *E. coli* HB101 cells. The resulting phasmids were electroporated into mc^2^155, and high-titer phage lysates were prepared to transduce H37Rv. The deletion strains were confirmed by whole genome sequencing (MiSeq Instrument, Illumina, San Diego, CA, following the protocol provided by Illumina).

### Lipid analysis

Cultures of mc^2^6230 (OD_600_, ~0.2) were treated with vitamin C (1 mM) and/or BDQ (0.9 µM) for 2 days and then labeled with ^14^C-acetate (10 µCi in 10 mL culture) for 22 h. The cultures were spun down. The cell pellets were resuspended in 1 mL of water, and the lipids were extracted with chloroform/methanol (2/1, vol/vol; 1 mL) thrice. The organic phases were collected, dried with anhydrous sodium sulfate, and evaporated to dryness under nitrogen. The residues were dissolved in chloroform/methanol (2/1, vol/vol; 0.2 mL). The samples (similar amount of cpm) were loaded onto a Silica gel 60 F254 250 µm aluminum plate (5 × 10 cm) and eluted (elution systems were as follows: hexane/ethyl acetate 98/2, three elutions; chloroform/methanol/water 100/14/08; or chloroform/methanol/water 10/5/1). ^14^C-radiolabeled species were detected by autoradiography after exposure at −80°C for 14 days on X-ray film.

### ATP measurement

Cultures of mc^2^6230 (OD_600_, ~0.1) were treated with vitamin C (1 mM) and/or BDQ (0.9 µM) for up to 10 days. At the indicated time, samples (0.1 mL) were taken and added to 0.5 mL of boiling 0.1M Tris buffer (pH 7.75) containing 3 mM EDTA. Glass beads (0.1 mL) were added to each sample, and the samples were vortexed for 45 s, heated at 100°C for 90 s, and cooled down. Cell debris was removed by centrifugation, and ATP levels were measured using ENLITEN ATP Assay (Promega, Madison, WI) following the manufacturer’s instructions.

### Cell line experiment

*Mtb* H37Rv was grown to exponential phase (OD_600_, ~0.9), spun down, washed twice with DPBS, and sonicated for 10 s. J774 cells were grown in Dulbecco’s modified Eagle medium (DMEM; Gibco) supplemented with 10% heat-inactivated fetal bovine serum (FBS; Gemini Bio-Products, West Sacramento, CA). The cells were seeded at a concentration of 5 × 10^5^ cells in sterile 24-well plates. J774 cells were infected with H37Rv (2 × 10^6^ CFUs; MOI of 4) for 4 h at 37°C in 5% CO_2_ to allow for mycobacterial uptake. Extracellular *Mtb* bacilli were removed by washing the cell monolayers twice with DPBS. DMEM/FBS (1 mL) was added to each well with vitamin C (1 mM) and/or BDQ (0.9 µM).

THP1 cells, grown in RPMI 1640 containing 10% FBS and 1× GlutaMAX (cRPMI), were seeded overnight with phorbol 12-myristate 13-acetate (PMA; 0.04 mg/0.5 mL) at 37°C in 5% CO_2_ at a concentration of 2 × 10^5^ cells in 24-well plates. THP1 cells were infected with *Mtb* (5 × 10^5^ CFUs; MOI of 2, see preparation of the *Mtb* culture above) for 4 h at 37°C in 5% CO_2_ to allow for mycobacterial uptake. Extracellular *Mtb* bacilli were removed by washing the cell monolayers twice with DPBS. RPMI/FBS (1 mL) was added to each well with vitamin C (1 mM) and/or BDQ (0.9 µM).

The *Mtb*-infected J774 or THP1 cells were incubated at 37°C in 5% CO_2_. Medium with all supplements (vitamin C, BDQ) was replenished at every time point. At each time point, the media was removed, and the cells were washed once with DPBS and then lysed with 0.05% aqueous sodium dodecyl sulfate (1 mL). CFUs were determined by plating serially diluted lysates onto Middlebrook 7H10 plates (see above).

### Human PBMC isolation

Peripheral blood mononuclear cells (PBMC) were isolated from the leucocytes of healthy blood donors, purchased from Stanford Health Care Blood Center. Red blood cells were further removed by centrifugation on Lymphoprep (Fresenius Norge), and recovered PBMCs were washed with PBS to remove platelets. Monocytes were isolated using a magnetic cell separation system with anti-CD14 mAb-coated microbeads (Miltenyi Biotec). CD14-positive monocytes were seeded into 24-well plates at 7–10 × 10^5^ cells/well and differentiated into macrophages in complete RPMI 1640 medium (Gibco) supplemented with 10% heat-inactivated human AB^+^ serum, 50 nM β-mercaptoethanol (Gibco), penicillin-streptomycin (Sigma-Aldrich), and 25 ng/mL M-CSF (Peprotech) at 37°C under a humidified 5% CO_2_ atmosphere for 6 days. Medium was replaced on day 2. On day 4, cells were washed with PBS, and the medium was replaced with antibiotic-free medium. The cells were further incubated until day 6 when the infection experiments took place.

### *Ex vivo* infection

Mid-log cultures of *Mtb* mc^2^6020 were pelleted, washed twice in PBS pH 7.4, and resuspended in serum-free RPMI medium at 5–8 × 10^9^ CFU/mL. Bacterial suspensions were passed through a 23-gauge needle to disrupt bacterial clumps. Residual aggregates were removed by low-speed centrifugation at 120 × *g* for 2 minutes. Single-cell bacterial suspension was verified by microscopy. PBMCs at a density of 7–10 × 10^5^ per well were infected with *Mtb* at MOI 5–10:1 for 3 hours. PBMCs were washed twice with PBS to remove extracellular bacteria. After 2 hours of incubation in RPMI medium with human AB^+^ serum, antibiotics (BDQ [2.7 µM], RIF [1.2 µM], INH [7.3 µM], moxifloxacin [13 µM], amikacin [8 µM], clofazimine [11 µM]) and vitamin C (1 mM) were added alone or in combination. Culture medium was changed on day 3, and fresh drugs were added. On day 6, PBMCs were lysed using distilled water containing 0.005% Tween 80, and serial dilutions of the lysates were plated on Middlebrook 7H10 agar medium supplemented with OADC. Colonies were enumerated after about 2–3 weeks of incubation at 37°C.

### Statistics

Differences between groups were analyzed by a two-tailed, unpaired *t*-test using GraphPad Prism 7.05 (San Diego, CA).

## Data Availability

Illumina paired end sequence reads of the RNA seq data from this study are deposited in the SRA database under BioProject ID PRJNA1206187 with SRA accession numbers SRR31886240, SRR31886241, SRR31886242, SRR31886243, SRR31886244, SRR31886245, SRR31886246, and SRR31886247.

## SUPPLEMENTAL MATERIAL

Supplemental figures mbio.01484-25-s0001.pdf Figures S1 to S10.

Table S1 mbio.01484-25-s0002.pdf Log2 fold change and fold change in gene expression in the vitamin C-, BDQ-, and BDQ/vitamin C-treated samples at day 3 compared to the expression in untreated sample at day 0.

Table S2 mbio.01484-25-s0003.pdf Primers used in study.

ASM does not own the copyrights to Supplemental Material that may be linked to, or accessed through, an article. The authors have granted ASM a non-exclusive, world-wide license to publish the Supplemental Material files. Please contact the corresponding author directly for reuse.
