# Supplementary material for: Vitamin C potentiates the killing of Mycobacterium tuberculosis by bedaquiline through metabolic disruption
Source: mBio. 2025 Jun 25;16(8):e01484-25. doi: 10.1128/mbio.01484-25 (PMC12345178; doi:10.1128/mbio.01484-25)
Supplement: Supplemental figures — Figures S1 to S10. [file mbio.01484-25-s0001.pdf]

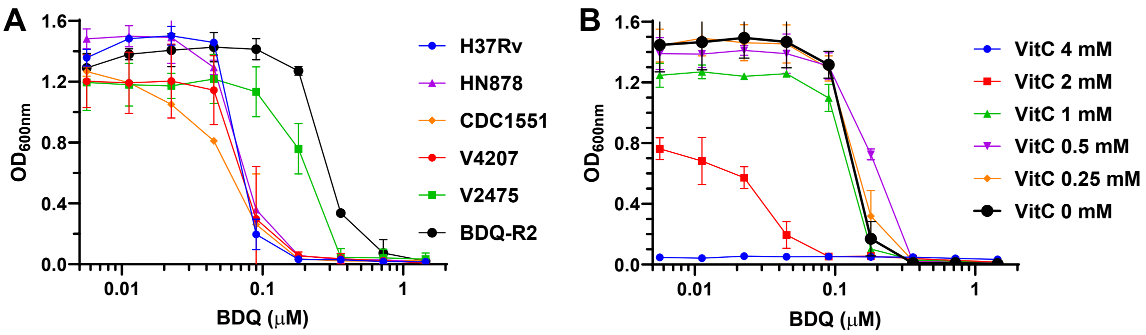

Figure S1. Minimum inhibitory concentration of BDQ. (**A**) BDQ MIC against BDQ-susceptible and BDQ-resistant *Mtb* strains. (**B**) BDQ MIC against *Mtb* H37Rv in presence of increasing concentrations of vitamin C. FIC index was determined using the values 0.18 μM for BDQ MIC and 4 mM for vitamin C MIC. Mean with standard deviation is plotted (*n*= 2-3).

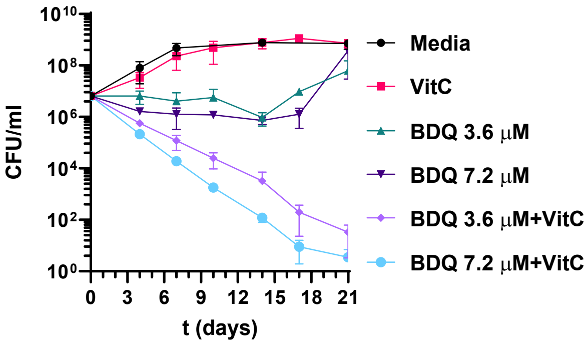

Figure S2. The combination BDQ/vitamin C is effective against an *Mtb* H37Rv BDQ-resistant *Mtb* mutant. *Mtb* H37Rv BDQ-R2, a laboratory-derived BDQ-resistant *Mtb* H37Rv mutant, was treated with vitamin C (VitC, 1 mM), BDQ (3.6 and 7.2 µM) or the combination BDQ/vitamin C, all added once at t=0. At the indicated time points, samples were taken, serially diluted, and plated to determine CFU/ml. Mean with standard deviation is plotted (*n*= 2).

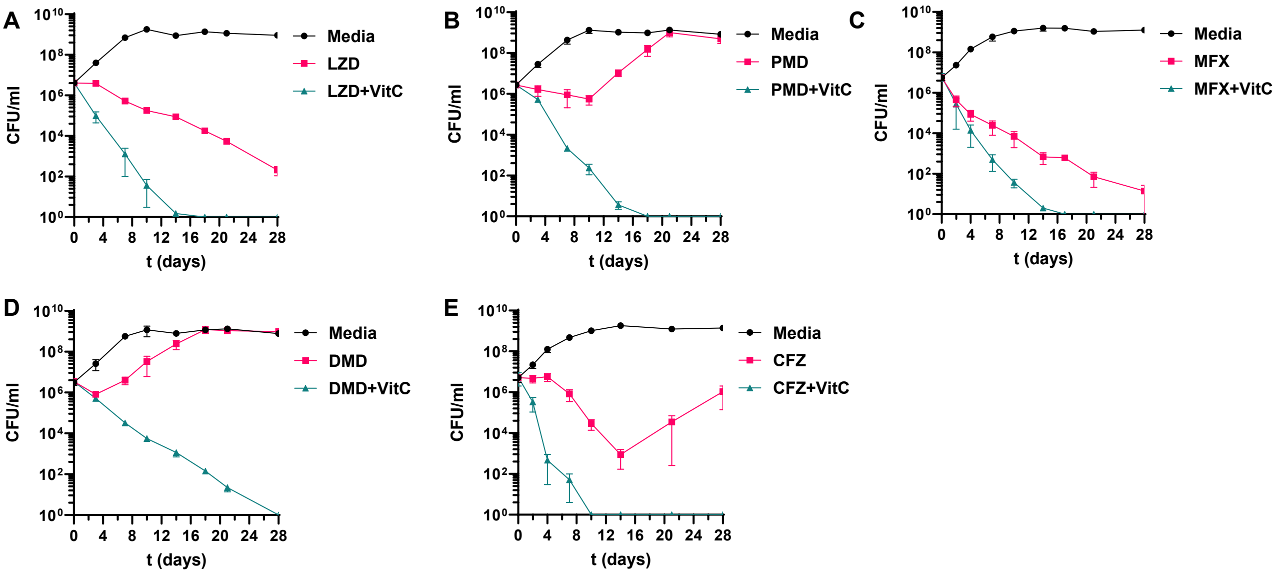

Figure S3. Potentiation of second-line TB drugs with vitamin C. *Mtb* H37Rv was treated with vitamin C (VitC, 1 mM), linezolid (**A**, LZD, 7.4 μM), pretomanid (**B**, PMD, 1.7 μM), moxifloxacin (**C**, MFX, 13 μM), delamanid (**D**, DMD, 0.07 μM), clofazimine (**E**, CFZ, 11 μM) or the combination TB drug/vitamin C, all added once at t=0. At the indicated time points, samples were taken, serially diluted, and plated to determine CFU/ml. Mean with standard deviation is plotted (*n*= 2-3).

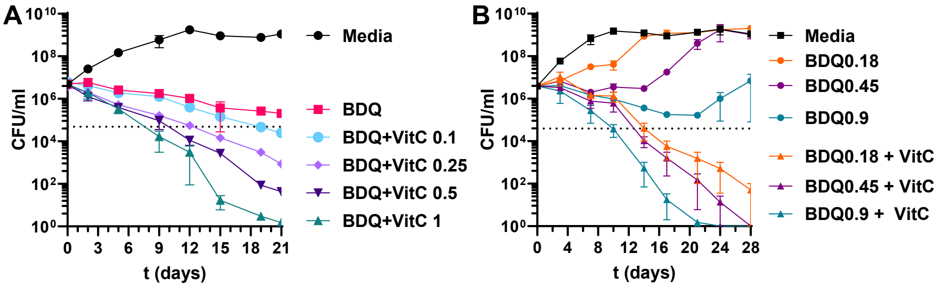

Figure S4. The potentiation of DBQ by vitamin C is dependent on vitamin C and BDQ concentrations. A) *Mtb* H37Rv was treated with vitamin C (VitC, 0.1 to 1 mM), bedaquiline (BDQ, 0.9 µM), or the combination BDQ + VitC, added once at day 0. **B**) *Mtb* H37Rv was treated with vitamin C (VitC, 1 mM), bedaquiline (BDQ, 0.18 to 0.9 µM), or the combination BDQ + VitC, added once at day 0. At the indicated time points, samples were taken, serially diluted, and plated to determine CFU/ml. Mean with standard deviation is plotted (*n*= 2). The dotted lines indicate a two-log reduction in CFUs.

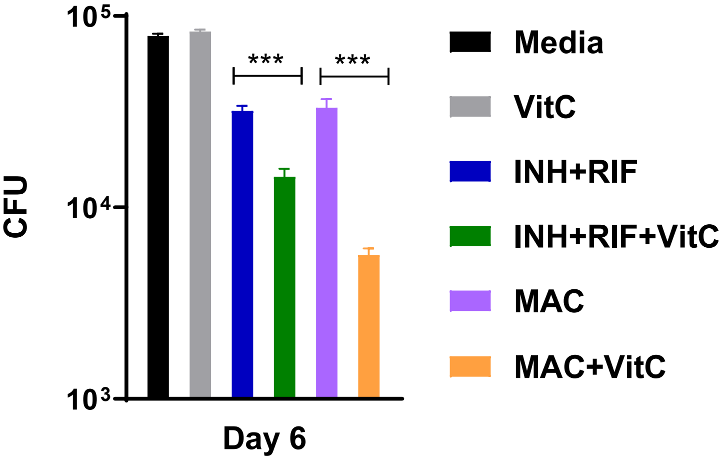

Figure S5. Adjunct activity of vitamin C in PBMCs. PBMCs were infected with *Mtb* mc^{2}6020 (MOI 5-10) for 3 h prior to treatment with vitamin C (VitC, 1 mM), RIF (1.2 µM), INH (7.3 µM), MAC (moxifloxacin (13 µM) + amikacin (8 µM) + clofazimine (11 µM)) or the indicated combinations. On day 6, macrophage cell lines were lysed. The lysates were serially diluted and plated to determine CFU/ml. Mean with standard deviation is plotted (*n*= 12). * denotes significant differences ($p < 0.05$).

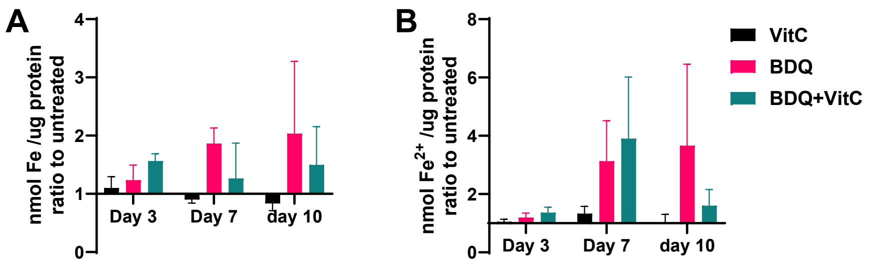

Figure S6. Effect of vitamin C addition on free ferrous and ferric ion intracellular concentrations. *Mtb* $mc^2$6230 was treated with vitamin C (VitC, 1 mM), bedaquiline (BDQ, 0.9 μM) or the combination [BDQ (0.9 μM) + VitC (1mM)]. At the indicated time, samples were taken, and free iron (ferric + ferrous) or ferrous ion levels were measured using the ferrozine colorimetric assay. The data reported is the ratio of treated cells to untreated cells. Mean with standard deviation is plotted (*n*= 2).

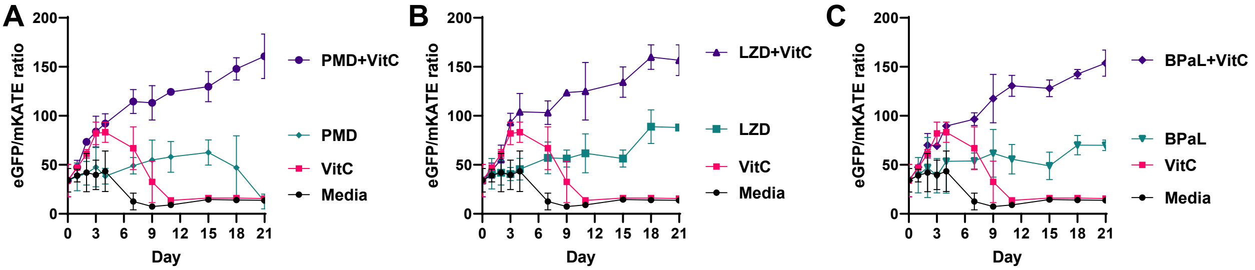

Figure S7. Effect of vitamin C addition on heme consumption in drug-treated *Mtb* cultures. *Mtb* mc^{2}8957 (mc^{2}7901 expressing the HS1-M7A heme sensor) was treated with vitamin C (1 mM), pretomanid (**A**, PMD, 1.7 µM), linezolid (**B**, LZD, 7.4 µM), BPaL (**C**, BDQ, 0.9 µM; PMD 1.7 µM; LZD, 7.4 µM) or the combination drug(s) + vitamin C for up to 21 days. At the indicated times, samples were taken, and fluorescence was measured (eGFP, excitation 480 nm and emission 510 nm; mKATE2, excitation 580 nm and emission 620 nm). Mean with standard deviation is plotted (*n*= 2).

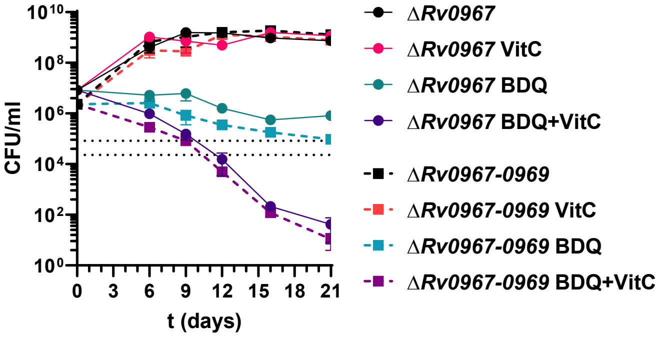

Figure S8. Deletion of the copper-sensitive operon *Rv0967-Rv0969* has limited effect on the potentiation of BDQ by vitamin C. *Mtb* H37Rv Δ*Rv0967* and *Mtb* H37Rv Δ*Rv0967-Rv0969* were treated with vitamin C (VitC, 1 mM), bedaquiline (BDQ, 0.9 μM), or the combination BDQ + VitC, added once at day 0. At the indicated time points, samples were taken, serially diluted, and plated to determine CFU/ml. Mean with standard deviation is plotted (*n*= 2). The dotted lines indicate a two-log reduction in CFUs.

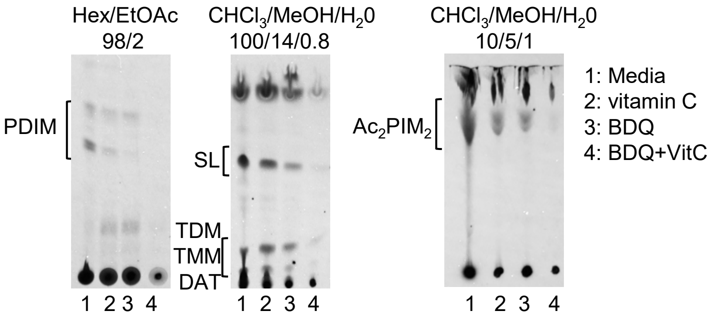

Figure S9. Lipid biosynthesis inhibition by the combination of vitamin C and BDQ. *Mtb* mc^{2}6230 was treated with vitamin C (VitC, 1 mM), bedaquiline (BDQ, 0.9 µM) or the combination [BDQ (0.9 µM) + VitC (1mM)] for two days and then labeled with ^{14}C-acetate (10 µCi) for 22 h. Lipids were extracted and analyzed by thin layer chromatography.

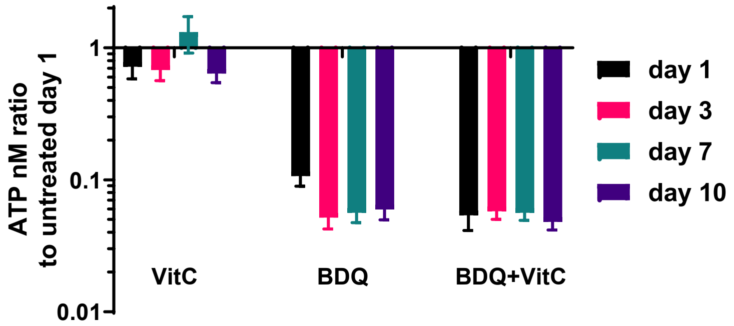

Figure S10. The addition of vitamin C to BDQ does not enhance ATP depletion. *Mtb* mc^{2}6230 was treated with vitamin C (VitC, 1 mM), bedaquiline (BDQ, 0.9 μM) or the combination [BDQ (0.9 μM) + VitC (1mM)] for up to ten days. At the indicated time, samples were taken, and ATP levels were measured using ENLITEN ATP Assay kit following manufacturer's protocol. Mean with standard deviation is plotted (*n*= 2).
