# Supplementary material for: Vitamin C potentiates the killing of Mycobacterium tuberculosis by bedaquiline through metabolic disruption
Source: mBio. 2025 Jun 25;16(8):e01484-25. doi: 10.1128/mbio.01484-25 (PMC12345178; doi:10.1128/mbio.01484-25)
Supplement: Table S1 — Log2 fold change and fold change in gene expression in the vitamin C-, BDQ-, and BDQ/vitamin C-treated samples at day 3 compared to the expression in untreated sample at day 0. [file mbio.01484-25-s0002.pdf]

Table S1: Log2 fold-change and fold change in gene expression in the vitamin C (VitC)-, BDQ- and BDQ/vitamin C (BDQ/VitC)-treated samples at day 3 compared to the expression in untreated sample at day 0 (DE: differential expression)

locus_tag	Name	Log2 VitC	Log2 BDQ	Log2 BDQ/VitC	Fold VitC	Fold BDQ	Fold BDQ/VitC	DE VitC p-value	DE BDQ p-value	DE BDQ/VitC p-value
Rv1992c	ctpG	0.2	-1.4	5.8	1	3	56	0.078159494	3.48E-37	0
Rv2466c	hypothetical protein	1.6	-2.0	5.6	3	4	48	4.44E-32	2.39E-66	0
Rv2383c	mbtB	-0.2	-2.9	5.3	1	8	39	0.182843953	6.94E-106	0
Rv1226c	transmembrane protein	0.1	-1.0	4.6	1	2	25	0.59020368	1.88E-10	1.15E-294
Rv1993c	hypothetical protein	0.4	-1.3	4.4	1	2	21	0.020576187	3.02E-17	2.81E-242
Rv0564c	gpdA1	0.2	-1.1	4.4	1	2	21	0.193034603	2.73E-23	3.94E-254
Rv2642	ArsR family transcriptional regulator	-0.3	-4.2	4.1	1	18	17	0.193994277	1.35E-178	5.24E-191
Rv0975c	fadE13	-0.1	-3.0	4.1	1	8	17	0.49478497	5.36E-111	1.48E-248
Rv1576c	phage capsid protein	0.9	-3.4	4.1	2	10	17	2.26E-14	2.29E-213	6.73E-288
Rv0974c	accD2	-0.1	-2.9	4.0	1	7	16	0.625778532	1.13E-120	1.95E-200
Rv0983	pepD	0.0	-1.6	3.9	1	3	15	0.719310912	2.83E-33	4.29E-272
Rv2641	cadI	-0.6	-5.8	3.9	2	57	15	0.000841977	0	1.22E-202
Rv0186	bglS	0.0	-0.8	3.9	1	2	15	0.824243284	6.24E-12	1.56E-260
Rv2382c	mbtC	0.5	-2.3	3.8	1	5	14	0.008158328	1.24E-64	1.33E-224
Rv1936	monooxygenase	-0.4	-1.9	3.8	1	4	14	0.018415629	4.01E-45	2.97E-229
Rv2650c	prophage protein	0.5	-2.7	3.8	1	7	14	5.84E-05	4.16E-133	3.65E-261
Rv0984	moaB2	0.4	-1.8	3.8	1	4	14	0.00249765	6.70E-60	2.25E-280
Rv2643	arsC	0.1	-2.4	3.8	1	5	14	0.367017864	1.22E-98	9.68E-248
Rv1994c	cmtR	0.1	-2.2	3.7	1	5	13	0.695108726	2.81E-63	1.09E-154
Rv1575	phage protein	-0.1	-2.4	3.7	1	5	13	0.439142378	9.01E-87	1.10E-248
Rv1573	phage protein	-0.3	-0.9	3.7	1	2	13	0.122885576	5.06E-06	2.25E-184
Rv0976c	hypothetical protein	-0.5	-2.3	3.6	1	5	12	0.003763651	2.22E-66	2.55E-187
Rv1224	tatB	0.7	-0.1	3.6	2	1	12	2.46E-08	0.275349445	6.13E-239
Rv2381c	mbtD	1.4	-2.3	3.6	3	5	12	3.17E-23	1.21E-78	1.97E-173
Rv1225c	hypothetical protein	0.0	-0.3	3.6	1	1	12	0.992868114	0.005418046	1.89E-243
Rv1227c	transmembrane protein	0.4	-0.6	3.5	1	1	11	0.015529701	0.000693946	2.63E-197
Rv0973c	accA2	-0.1	-2.8	3.5	1	7	11	0.55885392	1.38E-110	1.53E-169
Rv3535c	hsaG	0.5	-2.3	3.5	1	5	11	0.0134672	1.13E-48	8.84E-135
Rv3206c	moeB1	0.4	-0.8	3.3	1	2	10	0.003318943	1.28E-13	5.86E-191
Rv0251c	hsp	-1.1	-3.0	3.3	2	8	10	1.36E-12	4.12E-131	2.74E-163
Rv0374c	carbon monoxyde dehydrogenase sm	-0.5	-1.0	3.3	1	2	10	0.129588712	0.00041908	1.29E-89
Rv1968	mce3C	-0.5	-2.0	3.3	1	4	10	0.060099653	3.20E-25	2.29E-125
Rv1967	mce3B	-0.4	-2.0	3.3	1	4	10	0.191268757	2.04E-24	3.60E-97
Rv1937	oxygenase	0.1	-2.2	3.3	1	5	10	0.69264577	2.94E-76	1.30E-177
Rv1463	ABC transporter ATP-binding protein	0.6	-2.6	3.2	2	6	9	1.24E-06	1.30E-91	7.15E-184
Rv0185	hypothetical protein	0.1	0.8	3.2	1	2	9	0.612462609	8.88E-07	3.00E-175
Rv1970	lprM	-0.7	-1.8	3.2	2	4	9	0.013537695	4.98E-22	2.43E-118
Rv1464	csd	0.8	-2.4	3.2	2	5	9	8.60E-09	3.75E-121	1.27E-153
Rv2743c	hypothetical protein	0.5	-1.3	3.2	1	2	9	2.52E-05	1.43E-30	6.54E-159
Rv3202c	ATP-dependent DNA helicase	1.3	-1.9	3.2	2	4	9	1.41E-26	6.36E-61	1.04E-186
Rv2713	sthA	-0.2	0.0	3.2	1	1	9	0.051199468	0.719232562	7.87E-119
Rv3534c	hsaF	0.8	-2.4	3.1	2	5	9	3.06E-07	1.45E-85	5.71E-152
Rv1938	ephB	-0.2	-1.8	3.1	1	4	9	0.250000056	1.60E-28	2.71E-126

Rv1462	hypothetical protein	0.9	-3.0	3.1	2	8	9	4.37E-13	5.82E-173	3.95E-163
Rv0972c	fadE12	0.4	-3.1	3.1	1	9	9	0.026343819	9.40E-147	5.50E-140
Rv2834c	ugpE	-0.8	-0.3	3.1	2	1	9	0.002752579	0.102547888	2.61E-128
Rv0372c	hypothetical protein	-0.9	-1.3	3.1	2	2	8	0.008016302	3.26E-08	1.54E-96
Rv0368c	hypothetical protein	-0.6	-1.3	3.1	2	2	8	0.02125916	2.71E-08	1.97E-103
Rv2052c	hypothetical protein	-0.7	-0.5	3.1	2	1	8	5.19E-05	3.45E-05	1.56E-129
Rv0373c	carbon monoxyde dehydrogenase larg	-0.1	-1.7	3.0	1	3	8	0.636547157	2.28E-27	2.04E-137
Rv1222	rseA	-0.1	-0.7	3.0	1	2	8	0.559870645	3.78E-07	4.33E-179
Rv2384	mbtA	-0.5	-1.2	3.0	1	2	8	0.073888643	4.80E-09	3.19E-108
Rv1966	mce3A	-0.7	-1.6	3.0	2	3	8	0.028180362	7.92E-21	8.05E-124
Rv1050	oxidoreductase	-0.3	-1.3	3.0	1	2	8	0.234763855	2.31E-09	4.24E-109
Rv2646	integrase	0.1	-0.8	3.0	1	2	8	0.372696923	7.87E-07	2.26E-136
Rv0375c	carbon monoxyde dehydrogenase me	-0.4	-1.2	3.0	1	2	8	0.145750395	5.39E-07	6.99E-79
Rv0105c	rpmB1	-0.6	-0.8	3.0	2	2	8	0.110144475	0.013732554	2.08E-55
Rv1223	htrA	0.1	0.1	3.0	1	1	8	0.188555431	0.530914377	1.16E-172
Rv2647	hypothetical protein	0.2	-1.4	3.0	1	3	8	0.432765919	9.87E-18	1.53E-115
Rv1553	frdB	-0.6	-1.0	3.0	2	2	8	0.031242054	1.93E-05	4.13E-83
Rv1051c	hypothetical protein	-0.3	-1.3	3.0	1	2	8	0.291735787	4.25E-08	5.84E-79
Rv1973	Mce associated membrane protein	-0.6	-1.5	3.0	1	3	8	0.086896593	3.44E-06	1.25E-63
Rv1971	mce3F	-0.4	-1.7	2.9	1	3	8	0.084422222	4.91E-20	7.88E-88
Rv2503c	scoB	0.6	-1.1	2.9	1	2	8	1.96E-05	2.90E-20	3.40E-100
Rv1806	PE20	-0.4	-2.5	2.9	1	5	8	0.154240226	2.34E-35	3.22E-46
Rv3834c	serS	-0.5	-0.7	2.9	1	2	8	0.001680284	4.69E-05	4.42E-133
Rv2710	sigB	-0.3	-1.1	2.9	1	2	8	0.011003695	8.32E-33	2.34E-102
Rv1969	mce3D	-0.5	-1.6	2.9	1	3	8	0.066816705	1.60E-14	2.57E-107
Rv2712c	hypothetical protein	0.2	-0.6	2.9	1	1	8	0.178338131	7.71E-08	1.59E-110
Rv0351	grpE	0.4	0.4	2.9	1	1	8	0.01179828	0.000114722	1.12E-163
Rv1801	PPE29	-0.7	-1.1	2.9	2	2	8	0.000426069	3.41E-13	4.75E-121
Rv3447c	eccC4	-0.4	-1.4	2.9	1	3	7	0.049813045	2.62E-19	1.90E-123
Rv3205c	hypothetical protein	-0.2	-0.7	2.9	1	2	7	0.113938597	2.89E-09	8.31E-162
Rv2386c	mbtI	0.6	-1.2	2.9	2	2	7	0.000199632	7.50E-15	1.75E-95
Rv1706A	hypothetical protein	0.0	-2.6	2.9	1	6	7	0.890085165	1.09E-49	4.04E-85
Rv2742c	hypothetical protein	-0.2	-0.1	2.9	1	1	7	0.064718587	0.460097763	3.63E-158
Rv3468c	dTDP-glucose 4,6-dehydratase	-0.5	-1.3	2.9	1	2	7	0.003043673	8.58E-24	1.72E-126
Rv3203	lipV	-0.4	-1.0	2.8	1	2	7	0.025231889	1.24E-11	7.25E-132
Rv1999c	transporter	-0.7	-1.5	2.8	2	3	7	0.007084821	4.25E-11	2.12E-88
Rv3512	PE PGRS56	-0.9	-1.6	2.8	2	3	7	0.040062191	0.003943944	1.41E-83
Rv3469c	mhpE	-0.7	-1.0	2.8	2	2	7	0.000722696	1.39E-09	2.99E-101
Rv2833c	ugpB	-0.6	-0.1	2.8	2	1	7	0.000349788	0.475544677	1.60E-117
Rv3471c	hypothetical protein	-1.0	-1.0	2.8	2	2	7	0.007888968	0.000105221	3.19E-67
Rv1574	phage protein	-0.7	-1.5	2.8	2	3	7	8.51E-05	5.22E-28	4.65E-125
Rv1040c	PE8	-0.8	-1.3	2.8	2	3	7	0.006284971	2.93E-09	7.86E-77
Rv3446c	hypothetical protein	-1.3	-1.4	2.8	2	3	7	0.000162531	4.25E-10	8.24E-87
Rv2636	O-phosphotransferase	-0.4	-0.6	2.8	1	1	7	0.071174452	0.005881986	1.25E-83
Rv0376c	hypothetical protein	-0.4	-1.7	2.8	1	3	7	0.093088233	2.21E-26	2.98E-88
Rv1804c	hypothetical protein	-0.3	-1.3	2.8	1	2	7	0.064773599	5.16E-18	3.03E-72

Rv0371c	hypothetical protein	-0.7	-1.2	2.8	2	2	7	0.023827569	1.44E-06	8.12E-70
Rv1972	Mce associated membrane protein	-0.4	-1.8	2.8	1	3	7	0.209725116	9.05E-15	1.48E-62
Rv1965	yrbE3B	-0.5	-1.7	2.8	1	3	7	0.092351457	3.83E-16	1.23E-79
Rv0969	ctpV	-0.5	-0.4	2.8	1	1	7	0.000126226	8.59E-05	6.10E-144
Rv3514	PE PGRS57	-1.7	-1.3	2.8	3	2	7	5.27E-19	0.013129441	3.27E-74
Rv0602c	tcrA	-0.7	-0.8	2.8	2	2	7	0.031070607	0.00254123	2.58E-67
Rv1552	frdA	-0.9	-1.0	2.8	2	2	7	0.000810671	1.17E-09	1.36E-102
Rv1930c	hypothetical protein	-0.3	-1.4	2.8	1	3	7	0.40975066	1.99E-06	5.44E-51
Rv1654	argB	0.5	-1.8	2.8	1	3	7	0.013902926	4.36E-29	3.15E-100
Rv2310	excisionase	-0.4	-1.1	2.8	1	2	7	0.255410933	0.000564429	2.65E-47
Rv0350	dnaK	0.3	-0.3	2.8	1	1	7	0.007808675	0.000741541	6.08E-138
Rv1991c	mazF6	-0.2	0.0	2.7	1	1	7	0.06365063	0.934093972	4.28E-63
Rv2813	hypothetical protein	-0.8	-0.9	2.7	2	2	7	0.008944424	8.69E-07	1.23E-60
Rv3800c	pks13	-0.3	-0.3	2.7	1	1	7	0.013070521	0.013812515	8.25E-149
Rv2317	uspB	-0.7	-0.7	2.7	2	2	7	0.030856406	0.004334105	1.51E-79
Rv0620	galK	-1.5	-1.0	2.7	3	2	6	0.000124987	0.000536039	2.37E-57
Rv1395	HTH-type transcriptional regulator	-0.8	-0.7	2.7	2	2	6	4.93E-09	2.36E-08	1.82E-97
Rv3473c	bpoA	0.1	-0.9	2.7	1	2	6	0.631777833	1.16E-07	3.96E-98
Rv2122c	hisE	-0.8	-1.2	2.7	2	2	6	0.033260827	1.16E-05	3.07E-50
Rv0384c	clpB	-0.6	-0.4	2.7	2	1	6	5.39E-06	1.59E-05	3.01E-141
Rv2501c	accA1	0.6	-2.9	2.7	1	7	6	4.29E-06	1.68E-176	1.04E-130
Rv2487c	PE PGRS42	-0.4	-1.4	2.7	1	3	6	0.093786726	0.00170773	3.36E-71
Rv3270	ctpC	0.7	-0.9	2.7	2	2	6	0.000186424	2.89E-12	1.55E-103
Rv1571	hypothetical protein	0.1	-0.5	2.7	1	1	6	0.800713891	0.041861958	3.67E-88
Rv2546	vapC18	0.0	-0.5	2.6	1	1	6	0.966712873	0.112276905	4.49E-55
Rv3448	eccD4	-0.9	-1.3	2.6	2	2	6	0.000235506	5.33E-12	1.79E-85
Rv1134	hypothetical protein	-0.2	-0.8	2.6	1	2	6	0.521348579	0.038651784	5.19E-27
Rv0260c	transcriptional regulator	-0.2	-1.1	2.6	1	2	6	0.28399471	4.63E-09	3.28E-84
Rv3507	PE PGRS53	-1.0	-1.4	2.6	2	3	6	3.75E-06	0.00384067	6.49E-77
Rv2812	transposase	0.1	-1.0	2.6	1	2	6	0.603057316	2.04E-07	7.62E-77
Rv2744c	35kd ag	0.7	-1.2	2.6	2	2	6	4.61E-08	9.43E-30	8.10E-77
Rv3511	PE PGRS55	-1.2	-1.4	2.6	2	3	6	3.16E-07	0.01078909	1.99E-79
Rv2385	mbtJ	0.7	-1.1	2.6	2	2	6	8.18E-05	5.95E-09	5.18E-86
Rv0094c	hypothetical protein	0.6	-0.7	2.6	2	2	6	0.000994563	3.38E-08	1.17E-76
Rv0601c	two component sensor kinase HK2	-0.5	-0.8	2.6	1	2	6	0.055095315	0.001442367	4.12E-62
Rv3467	hypothetical protein	0.6	-0.6	2.6	2	2	6	0.001851552	5.88E-07	2.80E-79
Rv3085	oxidoreductase SadH	0.3	-1.5	2.6	1	3	6	0.151343158	9.34E-12	2.00E-64
Rv3344c	PE PGRS49	-0.7	-1.4	2.6	2	3	6	0.006671851	0.020206162	1.65E-61
Rv3533c	PPE62	0.4	-1.3	2.6	1	2	6	0.044538064	5.84E-25	1.12E-116
Rv0160c	PE4	-0.9	-0.5	2.6	2	1	6	0.004391033	0.002449937	6.43E-90
Rv0142	hypothetical protein	0.3	-0.6	2.6	1	1	6	0.119802453	0.000323241	6.60E-90
Rv0754	PE PGRS11	-0.3	-3.2	2.6	1	9	6	0.063516294	9.98E-172	2.32E-106
Rv2835c	ugpA	-0.8	-0.2	2.6	2	1	6	1.13E-05	0.2864559	8.78E-73
Rv2690c	integral membrane protein	0.3	-0.6	2.6	1	2	6	0.101363291	7.53E-08	5.47E-110
Rv1450c	PE PGRS27	-1.4	-1.3	2.6	3	2	6	9.43E-14	0.007875491	3.31E-70
Rv1554	frdC	-1.0	-1.1	2.6	2	2	6	0.001430071	3.50E-05	4.35E-60

Rv0603	hypothetical protein	-0.8	-1.0	2.6	2	2	6	0.06434504	0.007207428	2.76E-33
Rv0566c	hypothetical protein	0.3	0.5	2.6	1	1	6	0.070744706	0.003503841	2.56E-79
Rv0600c	two component sensor kinase HK1	-0.3	-0.9	2.6	1	2	6	0.197650883	1.31E-05	1.48E-44
Rv3536c	hsaE	0.5	-2.0	2.6	1	4	6	0.000224171	4.22E-53	5.42E-108
Rv3370c	dnaE2	-0.2	-1.5	2.6	1	3	6	0.301315097	8.92E-35	5.25E-92
Rv1688	mpg	-1.2	-0.9	2.5	2	2	6	0.000317851	0.001125046	2.62E-56
Rv3345c	PE PGRS50	-0.7	-1.4	2.5	2	3	6	0.101989268	0.015120304	5.72E-61
Rv3201c	ATP-dependent DNA helicase	0.5	-1.5	2.5	1	3	6	2.03E-05	1.23E-40	4.82E-116
Rv1655	argD	0.2	-1.6	2.5	1	3	6	0.299680164	2.08E-29	1.13E-91
Rv2465c	rpiB	-0.4	0.0	2.5	1	1	6	0.009042502	0.724066655	6.92E-114
Rv3508	PE PGRS54	-1.1	-1.1	2.5	2	2	6	1.30E-08	0.03288094	1.11E-56
Rv0990c	hypothetical protein	-0.3	-0.6	2.5	1	2	6	0.04299596	9.09E-05	6.19E-101
Rv1383	carA	-0.2	-0.9	2.5	1	2	6	0.183810736	1.88E-07	6.80E-93
Rv0574c	hypothetical protein	-0.3	-0.3	2.5	1	1	6	0.106649336	0.111999252	1.73E-84
Rv1991A	mazE6	-0.5	-0.1	2.5	1	1	6	0.000403398	0.304327734	2.20E-45
Rv1088	PE9	-0.5	-1.0	2.5	1	2	6	0.193927509	0.000760571	5.45E-42
Rv2644c	hypothetical protein	0.3	-0.8	2.5	1	2	6	0.170369193	7.76E-06	2.37E-59
Rv1939	oxidoreductase	-0.9	-1.3	2.5	2	2	6	0.002606703	4.60E-10	1.39E-60
Rv0834c	PE PGRS14	-0.6	-2.2	2.5	2	4	6	1.15E-06	2.04E-89	4.14E-72
Rv1808	PPE32	-0.8	-0.6	2.5	2	2	6	1.76E-06	1.42E-05	1.40E-103
Rv1382	hypothetical protein	0.3	-0.8	2.5	1	2	6	0.222102731	0.000159523	5.99E-56
Rv1587c	hypothetical protein	0.7	-0.7	2.5	2	2	6	0.000283343	1.39E-09	3.03E-83
Rv0770	oxidoreductase	0.3	-0.7	2.5	1	2	6	0.052593101	5.93E-05	1.18E-83
Rv2689c	hypothetical protein	0.1	-0.7	2.5	1	2	6	0.643330488	2.20E-06	8.04E-109
Rv1381	pyrC	-0.5	-0.7	2.5	1	2	6	0.007226016	4.54E-06	1.23E-93
Rv0090	membrane protein	0.1	-0.7	2.5	1	2	6	0.731819536	5.84E-05	3.75E-77
Rv1371	membrane protein	-0.7	-1.0	2.5	2	2	6	0.001573099	9.46E-10	2.60E-87
Rv0122	hypothetical protein	0.8	-1.2	2.5	2	2	6	0.001042296	2.19E-06	2.65E-47
Rv2897c	hypothetical protein	-0.4	-0.8	2.5	1	2	5	0.055187044	0.000249483	1.07E-58
Rv3054c	hypothetical protein	-0.1	-1.0	2.5	1	2	5	0.557321908	8.29E-09	5.13E-68
Rv1067c	PE PGRS19	-1.0	-1.4	2.4	2	3	5	2.08E-07	0.005879559	2.76E-74
Rv1840c	PE PGRS34	-0.7	-1.4	2.4	2	3	5	0.000885919	0.004792158	1.33E-70
Rv3428c	transposase	-0.4	-0.7	2.4	1	2	5	0.03375362	4.72E-05	1.72E-90
Rv0261c	narK3	-0.3	-1.1	2.4	1	2	5	0.072010946	1.38E-11	8.68E-82
Rv1935c	echA13	-0.9	-0.8	2.4	2	2	5	3.73E-05	7.59E-05	2.61E-70
Rv3653	PE PGRS61	-0.8	-1.5	2.4	2	3	5	0.002917161	7.86E-09	1.17E-48
Rv0746	PE PGRS9	-1.7	-1.1	2.4	3	2	5	2.31E-16	0.01441927	3.12E-48
Rv3394c	hypothetical protein	-0.2	-1.2	2.4	1	2	5	0.188474308	5.08E-08	5.13E-76
Rv2283	hypothetical protein	-0.3	-1.0	2.4	1	2	5	0.396932061	0.001502644	1.26E-35
Rv0650	sugar kinase	-2.3	-1.2	2.4	5	2	5	1.51E-15	0.007571316	2.57E-52
Rv2811	hypothetical protein	-0.1	-1.1	2.4	1	2	5	0.667609275	1.86E-07	1.90E-70
Rv3573c	fadE34	-0.5	-0.9	2.4	1	2	5	0.022757209	3.69E-08	1.45E-84
Rv0833	PE PGRS13	-1.3	-1.3	2.4	3	2	5	2.17E-12	0.003260644	6.63E-52
Rv3271c	integral membrane protein	0.6	-0.3	2.4	2	1	5	8.51E-07	0.029032436	1.11E-97
Rv3017c	esxQ	0.2	-0.7	2.4	1	2	5	0.315468558	9.43E-05	8.56E-66
Rv0022c	whiB5	-0.7	-0.7	2.4	2	2	5	0.016569548	0.006291306	2.14E-52

Rv3167c	TetR family transcriptional regulator	-1.1	-0.9	2.4	2	2	5	0.002256461	0.000428588	5.25E-49
Rv2026c	universal stress protein	-0.1	-1.0	2.4	1	2	5	0.684778968	3.25E-08	1.95E-74
Rv2614A	hypothetical protein	0.3	-0.7	2.4	1	2	5	0.377773718	0.05917439	1.38E-26
Rv2730	hypothetical protein	-0.7	-0.5	2.4	2	1	5	0.003422179	0.011247016	6.64E-62
Rv2029c	pfkB	0.5	-1.7	2.4	1	3	5	0.004357805	7.48E-30	2.99E-78
Rv1653	argJ	0.5	-1.7	2.4	1	3	5	0.001589459	6.51E-29	9.69E-70
Rv3564	fadE33	0.2	-0.7	2.4	1	2	5	0.22098574	0.000131309	8.86E-74
Rv1759c	wag22	-0.8	-1.2	2.4	2	2	5	0.000237028	0.010807708	5.68E-52
Rv3222c	hypothetical protein	0.6	-0.8	2.4	1	2	5	1.93E-06	2.27E-11	2.46E-107
Rv0572c	hypothetical protein	1.3	-0.6	2.4	2	1	5	3.94E-17	0.001761822	3.15E-68
Rv1380	pyrB	0.2	-0.3	2.4	1	1	5	0.335508405	0.068492639	5.40E-65
Rv0278c	PE-PGRS family protein PE PGRS3	-1.0	-1.2	2.3	2	2	5	8.18E-08	0.006499902	5.63E-61
Rv3649	helicase	-0.6	-0.6	2.3	2	1	5	0.016714903	0.0050634	5.41E-80
Rv1577c	phage prohead protease	-0.1	-2.5	2.3	1	5	5	0.630268379	2.59E-71	2.65E-66
Rv0100	hypothetical protein	0.0	-0.8	2.3	1	2	5	0.997952508	1.92E-05	3.16E-51
Rv0622	membrane protein	0.0	-1.3	2.3	1	2	5	0.934029551	2.33E-15	7.48E-72
Rv3659c	conjugal transfer protein	-0.1	-1.0	2.3	1	2	5	0.494157815	7.83E-08	1.74E-71
Rv0096	PPE1	-1.2	-0.3	2.3	2	1	5	1.11E-08	0.134035137	2.09E-54
Rv0194	multidrug ABC transporter ATPase/pe	-1.0	-0.8	2.3	2	2	5	1.67E-06	5.65E-07	8.42E-84
Rv2490c	PE PGRS43	-0.9	-1.1	2.3	2	2	5	6.64E-05	0.013183163	2.49E-10
Rv0849	MFS-type transporter	-0.1	-1.5	2.3	1	3	5	0.469463293	1.09E-38	2.04E-92
Rv1452c	PE PGRS28	-1.4	-1.1	2.3	3	2	5	2.99E-16	0.009143267	1.12E-56
Rv3084	lipR	0.4	-1.3	2.3	1	2	5	0.060309137	3.84E-10	1.03E-48
Rv1931c	transcriptional regulator	-0.6	-0.8	2.3	1	2	5	0.017344618	0.000867879	2.16E-56
Rv2837c	bifunctional oligoribonuclease/PAP pl	0.6	0.1	2.3	2	1	5	7.41E-06	0.432000942	2.74E-83
Rv1974	membrane protein	0.3	-1.4	2.3	1	3	5	0.313627343	2.45E-08	7.16E-40
Rv1995	hypothetical protein	-0.1	-0.8	2.3	1	2	5	0.767112256	7.37E-06	5.81E-61
Rv3640c	transposase	-0.7	-0.6	2.3	2	2	5	0.001218966	0.00112384	4.56E-68
Rv3622c	PE32	-0.5	-0.5	2.3	1	1	5	0.059478107	0.057864025	1.53E-47
Rv0648	alpha-mannosidase	-1.4	-1.5	2.3	3	3	5	0.001404011	7.09E-17	2.43E-59
Rv1243c	PE PGRS23	-1.3	-1.1	2.3	2	2	5	3.20E-10	0.034286862	1.44E-45
Rv1687c	ABC transporter ATP-binding protein	0.8	-0.9	2.3	2	2	5	4.69E-05	3.87E-05	2.34E-57
Rv3388	PE PGRS52	-1.2	-1.1	2.3	2	2	5	3.09E-13	0.01113723	1.50E-68
Rv2719c	membrane protein	0.1	0.6	2.3	1	2	5	0.63344739	0.000230931	2.60E-74
Rv1394c	cyp132	-0.1	-1.0	2.3	1	2	5	0.399366216	3.91E-16	5.97E-80
Rv0971c	echA7	-0.5	-1.6	2.3	1	3	5	0.006146436	3.46E-28	2.70E-68
Rv0563	htpX	0.0	-1.2	2.3	1	2	5	0.802377045	3.60E-30	4.47E-69
Rv2974c	hypothetical protein	-0.1	-0.7	2.3	1	2	5	0.464048525	8.64E-06	5.98E-65
Rv1555	frdD	-0.1	-1.2	2.3	1	2	5	0.598851978	7.67E-09	5.97E-51
Rv3022A	PE29	-0.4	-1.1	2.3	1	2	5	0.039978146	6.49E-11	1.76E-49
Rv3470c	ilvB2	-0.3	-0.8	2.2	1	2	5	0.034002768	1.97E-07	1.74E-76
Rv0571c	hypothetical protein	0.6	-1.6	2.2	2	3	5	4.41E-06	6.27E-35	6.27E-77
Rv1049	transcriptional repressor	-0.8	-1.5	2.2	2	3	5	0.000242306	7.29E-17	1.55E-61
Rv2164c	hypothetical protein	0.0	-1.0	2.2	1	2	5	0.950878524	2.86E-14	3.53E-79
Rv3563	fadE32	0.0	-0.9	2.2	1	2	5	0.831019453	0.000194966	4.88E-56
Rv1588c	hypothetical protein	0.6	-0.9	2.2	2	2	5	0.000177039	5.70E-12	3.24E-60

Rv2634c	PE PGRS46	-1.0	-1.1	2.2	2	2	5	2.17E-05	0.002415334	5.70E-08
Rv2371	PE PGRS40	0.6	-1.3	2.2	1	3	5	0.02221671	5.86E-09	4.53E-37
Rv1673c	hypothetical protein	0.5	-0.9	2.2	1	2	5	0.004425474	9.95E-07	1.96E-57
Rv3839	hypothetical protein	0.5	-2.0	2.2	1	4	5	0.000829798	1.20E-59	2.18E-80
Rv2687c	antibiotic ABC transporter permease	0.2	-2.3	2.2	1	5	5	0.183650007	8.63E-81	1.68E-72
Rv0836c	hypothetical protein	0.4	-1.6	2.2	1	3	5	0.036489994	1.65E-29	5.16E-70
Rv2224d	hypothetical protein CDS	-0.6	-1.5	2.2	1	3	5	0.067291107	1.21E-09	8.53E-43
Rv1068c	PE PGRS20	0.0	-1.1	2.2	1	2	5	0.777508227	0.019764925	4.49E-65
Rv3379c	dxs2	0.2	-0.7	2.2	1	2	5	0.291270758	1.58E-05	6.48E-55
Rv2839c	infB	0.8	-0.1	2.2	2	1	5	2.16E-10	0.162431543	3.77E-96
Rv1621c	cydD	0.6	-2.1	2.2	2	4	5	0.000212858	5.01E-63	6.76E-83
Rv3590c	PE PGRS58	-1.1	-1.1	2.2	2	2	5	2.56E-09	0.001318147	6.10E-45
Rv3449	mycP4	-0.9	-0.9	2.2	2	2	5	0.000119457	3.05E-07	4.98E-63
Rv2003c	hypothetical protein	0.4	-2.4	2.2	1	5	5	0.019309177	7.07E-53	8.40E-61
Rv2316	uspA	-0.9	-0.5	2.2	2	1	5	0.000343853	0.02738407	7.20E-58
Rv0279c	PE PGRS4	-1.0	-1.1	2.2	2	2	5	2.42E-09	0.002652804	4.26E-61
Rv2667	clpC2	-0.6	-0.3	2.2	1	1	5	0.005959349	0.049883055	1.07E-68
Rv3352c	oxidoreductase	-0.8	-0.4	2.2	2	1	5	0.010231194	0.165169555	9.96E-42
Rv3829c	dehydrogenase	0.0	-0.6	2.2	1	2	5	0.764111558	2.03E-05	1.81E-77
Rv0631c	recC	-0.5	-1.1	2.2	1	2	5	0.00151535	2.65E-09	6.76E-85
Rv2659c	prophage integrase	0.1	-1.9	2.2	1	4	5	0.557316825	2.61E-61	9.09E-81
Rv3445c	esxU	-1.1	-0.2	2.2	2	1	5	0.000246833	0.415391287	3.77E-39
Rv3728	membrane protein	0.0	-0.5	2.2	1	1	5	0.794330078	0.000316762	3.08E-77
Rv2838c	rbfA	0.4	0.3	2.2	1	1	5	0.003029534	0.017763301	4.11E-81
Rv0565c	monooxygenase	-0.3	0.1	2.2	1	1	4	0.035068856	0.562180658	1.32E-75
Rv0395	hypothetical protein	-1.0	-0.4	2.2	2	1	4	0.000108782	0.158700199	9.57E-39
Rv0848	cysK2	-0.7	-1.6	2.2	2	3	4	3.07E-07	1.03E-40	4.19E-88
Rv0573c	pncB2	-0.2	-0.6	2.2	1	2	4	0.223993719	3.84E-05	1.16E-63
Rv0161	oxidoreductase	0.3	-0.6	2.2	1	2	4	0.154632487	0.000563063	1.32E-60
Rv3801c	fadD32	-0.3	-0.2	2.2	1	1	4	0.012609852	0.034626728	9.14E-76
Rv0970	integral membrane protein	0.2	-0.9	2.2	1	2	4	0.152284104	1.29E-13	1.86E-78
Rv3204	DNA-methyltransferase	0.0	-0.7	2.2	1	2	4	0.913630042	4.42E-06	5.18E-77
Rv0378	hypothetical protein	-1.1	-0.2	2.2	2	1	4	7.23E-05	0.535718879	1.96E-37
Rv2654c	antitoxin	-0.7	-0.9	2.1	2	2	4	0.101844374	0.076716451	2.75E-11
Rv2126c	PE PGRS37	-0.8	-1.1	2.1	2	2	4	0.000279946	0.013528456	8.46E-35
Rv1674c	transcriptional regulator	-0.1	-0.8	2.1	1	2	4	0.643681642	0.000245932	1.69E-47
Rv1582c	phage protein	-0.2	-0.7	2.1	1	2	4	0.206244429	1.73E-08	1.50E-76
Rv3175	amidase	-0.4	-0.3	2.1	1	1	4	0.006606729	0.041246171	4.45E-74
Rv0689c	hypothetical protein	-0.7	-1.0	2.1	2	2	4	0.059924818	0.000934303	2.62E-28
Rv2653c	toxin	-1.0	-1.2	2.1	2	2	4	0.0079855	2.41E-05	2.86E-31
Rv2490a	hypothetical protein CDS	-0.2	-0.6	2.1	1	2	4	0.401625007	0.024651444	4.36E-35
Rv3367	PE PGRS51	-0.9	-1.2	2.1	2	2	4	1.54E-07	0.002626667	9.02E-63
Rv1569	bioF1	-1.3	-0.3	2.1	2	1	4	1.21E-11	0.08698823	1.48E-65
Rv0259c	hypothetical protein	0.4	-1.5	2.1	1	3	4	0.042312269	2.87E-22	1.02E-58
Rv1075c	hypothetical protein	-0.7	-0.6	2.1	2	2	4	0.000130908	5.74E-05	4.04E-65
Rv1441c	PE PGRS26	-0.8	-1.0	2.1	2	2	4	0.000103231	0.031085877	2.81E-58

Rv2891	hypothetical protein	-0.9	-0.8	2.1	2	2	4	0.002227707	0.01276293	4.14E-38
Rv0219	transmembrane protein	-0.6	-0.6	2.1	2	2	4	0.023110585	0.010557012	5.09E-47
Rv0765c	oxidoreductase	0.1	-1.7	2.1	1	3	4	0.718744721	1.60E-31	1.15E-63
Rv0745	hypothetical protein	-0.3	-1.1	2.1	1	2	4	0.278512484	5.28E-06	4.03E-34
Rv2577	hypothetical protein	0.2	-1.1	2.1	1	2	4	0.282380146	2.23E-12	6.20E-67
Rv1686c	ABC transporter permease	0.0	-0.8	2.1	1	2	4	0.990871765	0.001233146	8.57E-41
Rv1990c	transcriptional regulator	0.7	-0.9	2.1	2	2	4	2.01E-07	1.09E-10	4.65E-32
Rv3635	transmembrane protein	-0.8	-0.6	2.1	2	1	4	4.65E-05	0.000602606	1.94E-76
Rv3513c	fadD18	-0.2	-1.1	2.1	1	2	4	0.326129945	2.87E-15	8.14E-67
Rv3087	diacyglycerol O-acyltransferase	0.0	-2.2	2.1	1	5	4	0.993037644	9.11E-56	1.33E-55
Rv0649	fabD2	-2.5	-1.3	2.1	6	2	4	1.34E-11	1.37E-05	3.62E-30
Rv1715	fadB3	-0.6	-0.5	2.1	2	1	4	0.004740137	0.044272624	8.25E-56
Rv3558	PPE64	0.3	-0.7	2.1	1	2	4	0.072879068	5.34E-07	6.76E-64
Rv0736	rslA	-0.2	-0.5	2.1	1	1	4	0.357659711	0.03627121	2.91E-53
Rv0887c	hypothetical protein	-0.4	-0.6	2.1	1	2	4	0.104430953	0.011665427	6.96E-41
Rv2832c	ugpC	-0.5	0.2	2.1	1	1	4	0.003951501	0.249998265	8.89E-73
Rv2961	transposase	0.8	-1.1	2.1	2	2	4	6.18E-05	4.69E-09	1.04E-52
Rv0578c	PE PGRS7	-0.7	-1.2	2.1	2	2	4	0.000133151	0.004365298	5.14E-55
Rv3444c	esxT	-0.7	-0.8	2.1	2	2	4	0.025364042	0.004810624	4.88E-32
Rv3082c	virS	-0.9	-1.6	2.1	2	3	4	0.000593184	1.13E-21	2.99E-55
Rv3358	relK	-0.2	-1.1	2.1	1	2	4	0.597048761	5.47E-06	3.76E-35
Rv3830c	TetR family transcriptional regulator	0.1	-0.5	2.1	1	1	4	0.684708526	0.012583495	2.36E-48
Rv1267c	embR	-0.6	-0.7	2.1	2	2	4	0.001427409	7.37E-06	8.11E-65
Rv3836	hypothetical protein	0.3	0.3	2.1	1	1	4	0.011324532	0.028571269	3.40E-73
Rv1997	ctpF	0.8	-1.4	2.1	2	3	4	3.97E-10	8.03E-24	1.63E-65
Rv1091	PE PGRS22	-0.5	-0.8	2.1	1	2	4	0.013484382	0.01915351	1.59E-44
Rv0915c	PPE14	-0.7	-1.1	2.1	2	2	4	0.000503848	4.16E-14	6.50E-60
Rv3349c	transposase	-0.5	-0.7	2.1	1	2	4	0.013946565	6.58E-05	2.23E-61
Rv2745c	clgR	0.1	-1.5	2.1	1	3	4	0.484716915	1.07E-33	8.97E-78
Rv2768c	PPE43	0.0	-0.8	2.1	1	2	4	0.788597492	1.01E-05	2.25E-46
Rv1551	plsB1	-0.6	-0.4	2.1	1	1	4	0.002637061	0.014909518	1.23E-65
Rv2917	hypothetical protein	-0.7	-0.8	2.1	2	2	4	0.000268854	1.02E-07	7.48E-62
Rv1714	oxidoreductase	-0.3	-0.9	2.1	1	2	4	0.236029629	5.80E-06	3.72E-38
Rv1964	yrbE3A	-0.6	-1.6	2.1	2	3	4	0.003546208	2.94E-23	2.24E-52
Rv2515c	hypothetical protein	-0.6	-1.0	2.1	2	2	4	0.000973536	2.08E-10	3.30E-62
Rv2728c	hypothetical protein	-0.9	-1.2	2.1	2	2	4	0.000390087	2.03E-09	4.22E-44
Rv1087	PE PGRS21	-0.6	-1.1	2.1	1	2	4	0.001354025	2.09E-10	1.12E-48
Rv0119	fadD7	0.2	-0.5	2.1	1	1	4	0.398143707	0.008977522	2.03E-46
Rv3088	tgs4	0.3	-2.3	2.1	1	5	4	0.115264718	3.57E-72	2.09E-64
Rv0538	membrane protein	-0.2	-1.6	2.1	1	3	4	0.146788256	7.04E-35	2.34E-51
Rv3395c	hypothetical protein	0.8	-0.8	2.0	2	2	4	4.02E-05	1.00E-05	2.48E-37
Rv1758	cut1	-0.3	-0.9	2.0	1	2	4	0.087740045	1.88E-11	4.90E-66
Rv3223c	sigH	0.7	-0.3	2.0	2	1	4	1.64E-08	0.00072512	4.85E-44
Rv2407	ribonuclease Z	0.0	-0.8	2.0	1	2	4	0.908172821	0.000258708	4.45E-43
Rv0886	fprB	-0.7	-0.3	2.0	2	1	4	0.000165836	0.160449245	1.41E-58
Rv2615c	PE PGRS45	-0.8	-0.8	2.0	2	2	4	1.74E-05	4.52E-07	1.51E-56

Rv0924c	mntH	0.8	-0.4	2.0	2	1	4	7.32E-08	0.018291118	1.13E-62
Rv3466	hypothetical protein	0.7	-0.8	2.0	2	2	4	2.96E-05	6.78E-11	2.61E-52
Rv3657c	membrane protein	-0.7	-1.0	2.0	2	2	4	0.01011469	0.000318635	7.66E-38
Rv1586c	phage integrase	0.5	-1.1	2.0	1	2	4	0.000117607	3.66E-24	1.98E-76
Rv3333c	hypothetical protein	-0.7	-0.7	2.0	2	2	4	0.00469791	0.000697741	4.10E-43
Rv1726	oxidoreductase	0.2	-1.0	2.0	1	2	4	0.193668602	2.01E-09	3.11E-57
Rv0197	oxidoreductase	-0.1	-1.7	2.0	1	3	4	0.384960794	4.19E-48	1.16E-76
Rv2488c	LuxR family transcriptional regulator	-0.4	-1.0	2.0	1	2	4	0.01204062	3.51E-13	3.64E-58
Rv2741	PE PGRS47	0.2	-0.7	2.0	1	2	4	0.214753763	2.30E-07	1.98E-55
Rv0728c	serA2	-0.7	-0.7	2.0	2	2	4	0.000597438	0.00047787	7.32E-45
Rv3357	relJ	-0.4	-1.0	2.0	1	2	4	0.179231645	3.52E-05	4.72E-32
Rv0034	hypothetical protein	0.4	-0.5	2.0	1	1	4	0.26296095	0.141712014	6.92E-23
Rv0771	4-carboxymuconolactone decarboxyl	0.5	-0.3	2.0	1	1	4	0.024410211	0.182576234	6.60E-40
Rv1393c	monoxygenase	0.2	-0.9	2.0	1	2	4	0.131949792	1.84E-12	1.23E-58
Rv0352	dnaJ1	0.3	0.3	2.0	1	1	4	0.053970734	0.012553514	3.98E-85
Rv0382c	pyrE	-0.4	0.1	2.0	1	1	4	0.007533025	0.32192384	3.26E-58
Rv3506	fadD17	-0.1	-0.7	2.0	1	2	4	0.674186516	1.56E-05	1.64E-57
Rv2739c	transferase	-0.3	-0.1	2.0	1	1	4	0.041001947	0.699865587	7.81E-49
Rv2607	pdxH	0.1	-0.6	2.0	1	2	4	0.762234888	0.001599083	4.38E-51
Rv1145	transmembrane transport protein	-0.1	-0.3	2.0	1	1	4	0.416210476	0.163656163	2.26E-52
Rv3515c	fadD19	-0.5	-1.0	2.0	1	2	4	0.000489907	1.61E-19	1.02E-71
Rv1803c	PE PGRS32	-0.2	-0.8	2.0	1	2	4	0.540714913	1.62E-06	4.89E-07
Rv3656c	hypothetical protein	0.3	-1.1	2.0	1	2	4	0.310158445	1.03E-06	9.38E-33
Rv3559c	oxidoreductase	-0.2	-0.6	2.0	1	2	4	0.345398687	0.000900349	1.35E-51
Rv0629c	recD	-0.2	-0.7	2.0	1	2	4	0.160073462	1.06E-06	1.68E-65
Rv3658c	transmembrane protein	-0.7	-1.1	2.0	2	2	4	0.001315017	3.20E-11	8.33E-52
Rv1802	PPE30	-0.2	-0.4	2.0	1	1	4	0.267597038	0.024597892	1.43E-62
Rv0766c	cyp123	0.0	-1.7	2.0	1	3	4	0.833344962	7.51E-39	1.13E-52
Rv2807	hypothetical protein	0.3	-0.3	2.0	1	1	4	0.079776438	0.044012882	2.75E-55
Rv1325c	PE PGRS24	-0.5	-1.2	2.0	1	2	4	0.006102575	0.000323449	3.66E-42
Rv2077A	hypothetical protein	0.1	-0.5	2.0	1	1	4	0.788865651	0.097618744	6.61E-28
Rv0747	PE PGRS10	-0.5	-1.1	2.0	1	2	4	0.009778446	0.009340857	2.66E-40
Rv3540c	ltp2	0.3	-1.1	2.0	1	2	4	0.053431456	3.02E-15	8.75E-60
Rv2028c	universal stress protein	-0.2	-1.0	2.0	1	2	4	0.303086611	7.43E-08	1.48E-41
Rv3288c	usfY	2.2	-4.0	2.0	5	16	4	1.38E-76	0	8.81E-53
Rv0124	PE PGRS2	-1.0	-0.7	2.0	2	2	4	2.44E-09	9.26E-05	1.40E-40
Rv2265	integral membrane protein	-0.9	-0.7	2.0	2	2	4	2.74E-05	0.002482971	2.34E-45
Rv0186A	mymT	-2.2	-0.7	2.0	5	2	4	2.30E-33	8.76E-06	4.55E-31
Rv2064	cobG	-0.3	-0.9	2.0	1	2	4	0.217092423	3.32E-05	2.52E-44
Rv3560c	fadE30	0.0	-0.9	2.0	1	2	4	0.820375222	9.62E-07	3.86E-47
Rv1818c	PE PGRS33	-1.6	-1.1	1.9	3	2	4	2.79E-17	0.000877166	8.98E-36
Rv1809	PPE33	0.1	-0.4	1.9	1	1	4	0.376797895	0.005069623	3.58E-61
Rv0851c	short-chain type dehydrogenase/redu	0.3	-0.9	1.9	1	2	4	0.037331878	1.33E-10	1.10E-52
Rv3174	short-chain dehydrogenase/reductase	-1.6	0.2	1.9	3	1	4	2.82E-07	0.482812556	2.02E-39
Rv1188	proline dehydrogenase	0.1	-1.0	1.9	1	2	4	0.5589488	2.32E-06	1.90E-44
Rv0729	xylB	-0.8	-0.4	1.9	2	1	4	3.66E-05	0.043763858	2.56E-44

Rv2380c	mbtE	0.7	-1.6	1.9	2	3	4	1.18E-09	3.62E-56	1.06E-61
Rv1039c	PPE15	-0.3	-0.5	1.9	1	1	4	0.22850176	0.002365999	3.31E-48
Rv0196	HTH-type transcriptional regulator	-0.4	-2.4	1.9	1	5	4	0.101075118	1.45E-60	4.53E-45
Rv3541c	hypothetical protein	-0.2	-1.1	1.9	1	2	4	0.333949539	3.13E-10	1.10E-42
Rv1189	sigI	-0.3	-0.6	1.9	1	2	4	0.213165223	0.001634603	9.44E-42
Rv0661c	vapC7	-0.5	-0.8	1.9	1	2	4	0.109521274	0.004254244	1.50E-28
Rv0769	oxidoreductase	0.3	-0.4	1.9	1	1	4	0.110262609	0.025717028	4.34E-55
Rv3832c	hypothetical protein	-0.5	0.2	1.9	1	1	4	0.012740332	0.208562196	6.93E-50
Rv0109	PE PGRS1	-1.0	-0.5	1.9	2	1	4	2.36E-07	0.004280612	5.96E-35
Rv3553	oxidoreductase	0.1	-1.0	1.9	1	2	4	0.654671322	4.28E-09	4.64E-51
Rv3202a	hypothetical protein CDS	-0.1	-1.4	1.9	1	3	4	0.487158642	3.35E-16	1.41E-39
Rv1834	lipZ	-0.3	-0.8	1.9	1	2	4	0.170454555	3.38E-06	7.49E-49
Rv2053c	fxsA	-0.7	-0.2	1.9	2	1	4	5.95E-10	0.107087028	2.12E-74
Rv0977	PE PGRS16	-0.5	-1.0	1.9	1	2	4	0.000434598	2.77E-09	3.22E-48
Rv3538	dehydrogenase	0.5	-1.1	1.9	1	2	4	0.002830622	6.57E-15	1.62E-50
Rv0570	nrdZ	0.2	-0.8	1.9	1	2	4	0.130920364	2.76E-10	6.48E-63
Rv0325	hypothetical protein	0.5	-0.9	1.9	1	2	4	0.048431694	5.06E-05	1.00E-33
Rv1723	hydrolase	-0.1	-1.4	1.9	1	3	4	0.419803995	1.49E-19	2.15E-49
Rv1568	bioA	-0.6	0.4	1.9	2	1	4	3.26E-05	0.020284675	5.92E-59
Rv0493c	hypothetical protein	-1.2	-0.6	1.9	2	2	4	1.45E-09	0.000652381	3.83E-55
Rv1468c	PE PGRS29	-0.2	-1.1	1.9	1	2	4	0.242439796	8.23E-11	1.00E-51
Rv3904c	esxE	2.1	-0.7	1.9	4	2	4	1.46E-23	0.000805907	1.17E-36
Rv3013	hypothetical protein	0.5	-0.5	1.9	1	1	4	0.026779317	0.026811634	1.89E-35
Rv2004c	hypothetical protein	1.5	-3.3	1.9	3	10	4	4.33E-26	3.26E-194	1.54E-52
Rv3415c	hypothetical protein	-0.4	-0.5	1.9	1	1	4	0.085436725	0.014617082	2.78E-42
Rv3018c	PPE46	-0.7	-0.5	1.9	2	1	4	0.000308512	0.001104542	1.49E-36
Rv1817	flavoprotein	0.7	-1.4	1.9	2	3	4	2.90E-07	5.44E-35	1.05E-61
Rv3090	hypothetical protein	0.0	-0.3	1.9	1	1	4	0.789395217	0.126629911	3.18E-52
Rv3289c	transmembrane protein	2.0	-3.8	1.9	4	14	4	1.44E-62	1.05E-234	2.09E-50
Rv3450c	eccB4	-0.7	-0.4	1.9	2	1	4	0.000169589	0.051222213	7.84E-54
Rv2485c	lipQ	-0.9	-0.6	1.9	2	2	4	2.53E-06	0.000366386	3.00E-51
Rv2502c	accD1	-0.2	-1.3	1.9	1	3	4	0.143154524	1.40E-26	1.48E-65
Rv1866	hypothetical protein	0.1	-0.6	1.9	1	1	4	0.531170692	0.000461197	7.57E-58
Rv1585c	phage protein	1.1	-2.2	1.9	2	5	4	1.14E-13	1.39E-60	3.00E-36
Rv3837c	phosphoglycerate mutase	0.6	-0.5	1.9	2	1	4	3.26E-07	1.10E-07	1.68E-68
Rv1461	hypothetical protein	1.1	-2.7	1.9	2	7	4	4.62E-17	2.57E-176	6.03E-50
Rv0033	acpA	-0.2	-0.3	1.9	1	1	4	0.432536068	0.254729222	1.67E-24
Rv2688c	antibiotic ABC transporter ATP-bindin	-0.2	-2.4	1.9	1	5	4	0.28350928	3.25E-109	1.84E-54
Rv3565	aspB	0.2	-0.7	1.9	1	2	4	0.18524449	2.89E-06	4.00E-52
Rv0140	hypothetical protein	1.2	-1.3	1.9	2	2	4	2.49E-26	6.64E-33	1.80E-34
Rv1719	transcriptional regulator	0.0	-0.2	1.9	1	1	4	0.91647809	0.204969353	1.19E-45
Rv2541	hypothetical protein	-1.8	-0.6	1.9	3	2	4	6.19E-07	0.056442315	1.65E-22
Rv1190	hypothetical protein	0.1	-1.1	1.9	1	2	4	0.521802093	5.01E-08	1.88E-39
Rv3287c	rsbW	0.9	-2.4	1.9	2	5	4	3.89E-11	3.36E-92	1.39E-53
Rv1570	bioD	-0.4	-0.5	1.9	1	1	4	0.01946846	0.026001343	2.72E-45
Rv0881	tRNA/rRNA methyltransferase	-0.3	-0.4	1.9	1	1	4	0.080067209	0.025346143	1.75E-51

Rv0095c	hypothetical protein	0.8	-1.0	1.9	2	2	4	5.46E-09	2.47E-17	6.65E-45
Rv3505	fadE27	-0.2	-0.8	1.9	1	2	4	0.164860182	8.02E-07	4.35E-50
Rv0532	PE PGRS6	-0.2	-0.8	1.8	1	2	4	0.295948902	0.026626677	1.30E-27
Rv1735a	hypothetical protein CDS	0.2	-2.5	1.8	1	6	4	0.496779023	6.29E-47	2.03E-31
Rv3752c	cytidine/deoxycytidylate deaminase	-1.1	-0.5	1.8	2	1	4	5.36E-06	0.074190072	2.83E-36
Rv2157c	murF	-0.5	-0.8	1.8	1	2	4	0.009646106	2.39E-11	5.53E-60
Rv2836c	dinF	0.8	-0.4	1.8	2	1	4	0.000159644	0.011254744	1.68E-48
Rv2645	hypothetical protein	-1.1	-0.7	1.8	2	2	4	9.78E-06	4.05E-05	2.07E-29
Rv2816c	CRISPR-associated endoribonuclease	1.4	-2.7	1.8	3	7	4	3.54E-19	5.04E-136	1.27E-32
Rv1030	kdpB	0.3	-0.1	1.8	1	1	4	0.01519236	0.414492551	1.32E-57
Rv1406	fmt	-0.6	-0.9	1.8	2	2	4	0.00786232	5.75E-10	1.54E-49
Rv2727c	miaA	-0.1	-0.9	1.8	1	2	4	0.75489752	1.17E-08	3.76E-49
Rv3020c	esxS	0.7	-0.7	1.8	2	2	4	0.003423096	0.001431531	8.34E-39
Rv2670c	hypothetical protein	0.0	0.0	1.8	1	1	4	0.797898322	0.776220421	1.28E-46
Rv0592	mce2D	0.0	-0.4	1.8	1	1	4	0.989675326	0.01003103	2.05E-45
Rv1675c	cmr	-0.1	-0.3	1.8	1	1	4	0.549649879	0.18755446	1.55E-32
Rv2158c	murE	-1.0	-1.1	1.8	2	2	4	3.64E-05	2.27E-18	1.69E-34
Rv1268c	hypothetical protein	0.0	-1.1	1.8	1	2	4	0.862392865	1.18E-07	3.34E-32
Rv2692	ceoC	0.1	-0.1	1.8	1	1	4	0.438741492	0.329444375	7.35E-46
Rv1291c	hypothetical protein	0.2	-0.5	1.8	1	1	3	0.488474672	0.065970207	4.02E-21
Rv0266c	oplA	-0.6	-0.5	1.8	2	1	3	6.57E-07	4.80E-05	1.49E-61
Rv3368c	oxidoreductase	0.0	-0.1	1.8	1	1	3	0.86615123	0.57103179	3.20E-46
Rv0417	thiG	-0.3	-0.7	1.8	1	2	3	0.236167214	1.96E-05	1.79E-43
Rv2121c	hisG	-0.3	-0.7	1.8	1	2	3	0.277909275	0.000314971	1.19E-30
Rv2089c	pepE	0.9	-1.2	1.8	2	2	3	2.10E-07	8.38E-16	3.60E-46
Rv0630c	recB	-0.2	-0.6	1.8	1	2	3	0.091168049	0.000294664	4.65E-51
Rv2853	PE PGRS48	-0.7	-0.9	1.8	2	2	3	2.88E-05	4.52E-08	6.69E-40
Rv2318	uspC	-0.2	-1.4	1.8	1	3	3	0.293169159	6.52E-20	3.59E-44
Rv2624c	universal stress protein	-0.5	-0.8	1.8	1	2	3	0.011765487	7.49E-06	9.37E-36
Rv0370c	oxidoreductase	-0.5	-0.7	1.8	1	2	3	0.009371308	5.17E-06	1.75E-45
Rv1530	adh	0.7	-0.7	1.8	2	2	3	1.24E-05	3.89E-06	2.62E-44
Rv3638	transposase	0.3	-0.1	1.8	1	1	3	0.216242419	0.665237592	5.72E-47
Rv0625c	transmembrane protein	-0.3	-0.4	1.8	1	1	3	0.072396864	0.037262356	3.14E-42
Rv1407	fmu	0.1	-1.1	1.8	1	2	3	0.496921908	4.13E-15	2.40E-45
Rv0381c	hypothetical protein	-0.4	0.2	1.8	1	1	3	0.009949228	0.220838821	1.53E-46
Rv2063A	mazF7	-0.3	-0.2	1.8	1	1	3	0.206876074	0.452968919	5.82E-27
Rv2065	cobH	-0.9	-0.4	1.8	2	1	3	0.002087458	0.113814344	1.23E-32
Rv2372c	rRNA small subunit methyltransferase	0.5	-1.5	1.8	1	3	3	0.001157275	3.10E-41	3.20E-43
Rv0306	oxidoreductase	0.0	-1.6	1.8	1	3	3	0.932413431	7.44E-22	4.32E-36
Rv1003	rRNA small subunit methyltransferase	-0.4	-0.3	1.8	1	1	3	0.014234541	0.100176156	1.11E-39
Rv3500c	yrbE4B	0.2	-0.3	1.8	1	1	3	0.14136386	0.032998745	5.80E-47
Rv0852	hypothetical protein	-0.1	-0.6	1.8	1	2	3	0.753072544	0.001570156	5.71E-35
Rv1652	argC	-1.1	-1.7	1.8	2	3	3	1.38E-09	3.64E-36	4.32E-31
Rv3895c	eccB2	-0.2	-0.5	1.8	1	1	3	0.323203948	0.001496308	1.94E-40
Rv3650	PE33	-0.5	-0.6	1.8	1	2	3	0.038693308	0.009321703	1.68E-30
Rv2802c	arginine/hypothetical protein	-0.3	-0.7	1.8	1	2	3	0.161829279	6.10E-05	2.88E-45

Rv0324	transcriptional regulator	0.0	-0.8	1.8	1	2	3	0.789602899	1.11E-07	5.01E-47
Rv3112	moaD1	0.1	-0.2	1.8	1	1	3	0.693959303	0.495862533	5.76E-29
Rv3532	PPE61	-0.5	-0.4	1.8	1	1	3	0.003178884	0.00772949	2.32E-48
Rv0068	oxidoreductase	0.2	0.0	1.8	1	1	3	0.243033292	0.881991588	6.07E-37
Rv2573	2-dehydropantoate 2-reductase	-0.8	-0.8	1.7	2	2	3	0.000420016	2.63E-05	6.46E-36
Rv3529c	hypothetical protein	0.0	-1.7	1.7	1	3	3	0.776717794	1.63E-41	1.20E-49
Rv0540	hypothetical protein	0.7	-0.3	1.7	2	1	3	0.000233123	0.129594233	1.26E-36
Rv3595c	PE PGRS59	0.1	-0.8	1.7	1	2	3	0.578463689	4.21E-07	2.12E-26
Rv0846c	oxidase	-0.2	-1.3	1.7	1	2	3	0.188753505	5.82E-34	1.67E-52
Rv3080c	pknK	0.1	-1.9	1.7	1	4	3	0.595795919	1.67E-66	1.23E-56
Rv0853c	pdc	0.0	-1.0	1.7	1	2	3	0.756534697	2.63E-13	7.47E-51
Rv2319c	universal stress protein	0.7	-1.6	1.7	2	3	3	0.000340682	1.35E-27	8.64E-41
Rv2491	hypothetical protein	-0.5	-0.7	1.7	1	2	3	0.042939391	0.001736963	1.73E-25
Rv1088a	hypothetical protein CDS	-0.7	0.5	1.7	2	1	3	0.005736551	0.019997521	3.22E-35
Rv2980	hypothetical protein	-0.9	-0.9	1.7	2	2	3	2.59E-05	4.34E-05	3.04E-28
Rv1661	pks7	0.5	-0.7	1.7	1	2	3	0.014769098	5.50E-07	8.68E-35
Rv1076	lipU	1.1	-0.4	1.7	2	1	3	1.51E-10	0.010483848	5.31E-44
Rv0358	hypothetical protein	-1.0	0.0	1.7	2	1	3	1.99E-05	0.875135574	8.79E-30
Rv2505c	fadD35	-0.2	-0.4	1.7	1	1	3	0.302134057	0.004387935	5.38E-46
Rv3654c	hypothetical protein	-1.1	-0.9	1.7	2	2	3	0.000167402	0.005358658	4.16E-22
Rv2804c	hypothetical protein	-0.6	-0.4	1.7	1	1	3	0.018868232	0.116532833	3.16E-29
Rv0091	mtn	0.0	-0.1	1.7	1	1	3	0.856621868	0.53095098	1.52E-42
Rv2073c	oxidoreductase	-0.4	-0.7	1.7	1	2	3	0.030986159	1.53E-05	1.06E-36
Rv0032	bioF2	-0.5	0.1	1.7	1	1	3	0.009824149	0.477879568	8.21E-46
Rv1963c	mce3R	0.2	-0.7	1.7	1	2	3	0.197177014	2.54E-06	2.49E-42
Rv3113	phosphatase	-0.4	-0.8	1.7	1	2	3	0.10443854	1.05E-05	2.63E-37
Rv0416	thiS	-0.1	-0.4	1.7	1	1	3	0.607360147	0.109092366	6.96E-28
Rv0768	aldA	-1.3	0.1	1.7	2	1	3	1.76E-12	0.676064004	1.15E-44
Rv0737	transcriptional regulator	-0.4	-0.7	1.7	1	2	3	0.073211052	0.001208074	2.68E-35
Rv2598	hypothetical protein	-0.4	-0.3	1.7	1	1	3	0.064517073	0.17101174	1.03E-32
Rv0077c	oxidoreductase	-0.3	-0.8	1.7	1	2	3	0.159982719	1.06E-05	1.63E-36
Rv1945	hypothetical protein	1.4	-1.9	1.7	3	4	3	6.06E-28	2.26E-65	8.51E-48
Rv1084	hypothetical protein	-0.7	-1.6	1.7	2	3	3	1.47E-06	6.96E-51	4.80E-53
Rv1396c	PE PGRS25	-0.6	-0.8	1.7	1	2	3	0.000267785	7.21E-09	2.87E-24
Rv0386	transcriptional regulator	0.6	-0.9	1.7	2	2	3	1.11E-06	5.79E-16	1.65E-54
Rv1085c	hemolysin-like protein	-0.8	-0.4	1.7	2	1	3	4.91E-06	0.005631854	3.90E-40
Rv0855	far	-0.4	-0.6	1.7	1	2	3	0.012518045	8.05E-05	1.03E-39
Rv2693c	integral membrane protein	0.9	-1.5	1.7	2	3	3	1.00E-08	6.68E-34	1.50E-51
Rv2769c	PE27	0.1	-0.6	1.7	1	2	3	0.505830968	0.002671747	4.61E-32
Rv2529	hypothetical protein	-0.9	-0.1	1.7	2	1	3	1.47E-06	0.54549865	3.50E-35
Rv1455	hypothetical protein	-0.5	-0.5	1.7	1	1	3	0.009116586	0.013501925	1.48E-34
Rv1908c	katG	2.4	-2.1	1.7	5	4	3	1.57E-105	1.05E-101	1.19E-38
Rv1528c	papA4	-0.4	-0.5	1.7	1	1	3	0.052941809	0.006103124	3.30E-38
Rv2231B	vapB16	0.4	-0.5	1.7	1	1	3	0.28540634	0.222247264	7.72E-13
Rv3402c	hypothetical protein	1.0	-0.6	1.7	2	1	3	2.41E-13	9.14E-05	3.14E-40
Rv0985c	mscL	0.9	-1.4	1.7	2	3	3	2.29E-10	1.30E-39	2.23E-28

Rv0331	dehydrogenase/reductase	-0.9	0.1	1.7	2	1	3	8.88E-07	0.602846179	3.66E-46
Rv3552	CoA-transferase subunit beta	0.3	-0.6	1.7	1	1	3	0.101817207	0.0017754	4.53E-32
Rv1662	pks8	0.8	-0.4	1.7	2	1	3	0.030098123	0.000802919	3.42E-36
Rv0492c	GMC-type oxidoreductase	0.0	-1.2	1.7	1	2	3	0.896051574	2.81E-21	1.22E-46
Rv3562	fadE31	0.5	-0.2	1.7	1	1	3	0.001487248	0.364749107	4.46E-40
Rv2616	hypothetical protein	0.6	-1.3	1.7	2	3	3	0.000370304	5.43E-16	8.28E-35
Rv2071c	cobM	0.1	-0.2	1.7	1	1	3	0.576040119	0.309778225	1.30E-31
Rv2250c	HTH-type transcriptional regulator	-1.0	-0.3	1.7	2	1	3	1.89E-07	0.129313612	1.76E-37
Rv3369	hypothetical protein	-0.2	-0.8	1.7	1	2	3	0.27143332	3.47E-06	8.37E-34
Rv0210	hypothetical protein	-0.2	-0.3	1.7	1	1	3	0.20082285	0.132675516	1.28E-42
Rv3591c	hydrolase	-1.6	0.0	1.7	3	1	3	3.06E-11	0.985713624	1.82E-31
Rv3315c	cdd	0.5	-0.2	1.7	1	1	3	0.027271014	0.502315382	1.06E-30
Rv0123	hypothetical protein	2.1	-1.3	1.7	4	2	3	3.24E-42	7.20E-14	2.13E-21
Rv3504	fadE26	-0.1	-0.9	1.7	1	2	3	0.58977054	6.01E-12	1.97E-43
Rv1465	nitrogen fixation related protein	0.9	-2.2	1.6	2	5	3	1.46E-09	1.11E-62	1.13E-39
Rv1349	irtB	0.2	-0.5	1.6	1	1	3	0.277921295	0.006463409	1.22E-37
Rv2422	hypothetical protein	-1.3	-0.5	1.6	2	1	3	0.000317556	0.076939225	1.58E-18
Rv1651c	PE PGRS30	0.0	-0.7	1.6	1	2	3	0.849109829	1.57E-06	4.81E-30
Rv0673	echA4	0.1	-1.4	1.6	1	3	3	0.597638084	2.41E-24	9.21E-37
Rv0742	hypothetical protein	-0.4	-0.9	1.6	1	2	3	0.031688755	6.89E-05	1.71E-32
Rv1402	priA	-0.7	-0.5	1.6	2	1	3	4.35E-05	0.009246201	1.05E-43
Rv1583c	phage protein	-0.5	-1.7	1.6	1	3	3	0.058232088	1.36E-21	1.90E-27
Rv0895	diacylglycerol O-acyltransferase	-0.2	0.2	1.6	1	1	3	0.264448095	0.410980957	2.91E-37
Rv0738	hypothetical protein	-1.2	-1.0	1.6	2	2	3	1.70E-05	5.75E-05	5.76E-25
Rv0494	HTH-type transcriptional regulator	0.9	-1.0	1.6	2	2	3	3.02E-08	1.11E-07	6.72E-32
Rv2165c	rRNA small subunit methyltransferase	0.1	-0.2	1.6	1	1	3	0.206567685	0.140350874	2.13E-39
Rv3892c	PPE69	-0.9	-0.3	1.6	2	1	3	2.08E-05	0.106718322	7.93E-39
Rv2797c	hypothetical protein	-0.5	-0.5	1.6	1	1	3	0.003171735	0.001628917	3.56E-41
Rv0244c	fadE5	0.3	-2.1	1.6	1	4	3	0.001818278	4.80E-104	1.73E-35
Rv2025c	cation efflux system protein	-0.2	-1.3	1.6	1	3	3	0.184654854	1.23E-23	3.91E-38
Rv0393	hypothetical protein	-0.4	-0.6	1.6	1	2	3	0.015957614	6.70E-05	7.81E-31
Rv0794c	oxidoreductase	0.2	-0.7	1.6	1	2	3	0.169822078	2.04E-06	1.31E-35
Rv2803	hypothetical protein	-1.0	-0.1	1.6	2	1	3	9.12E-06	0.630101527	1.38E-27
Rv0779c	transmembrane protein	-0.6	-0.6	1.6	2	1	3	0.00772744	0.009099427	8.38E-27
Rv1163	narJ	-0.3	-0.6	1.6	1	2	3	0.052035025	2.33E-05	3.46E-37
Rv2484c	diacylglycerol O-acyltransferase	1.0	-2.4	1.6	2	5	3	6.07E-18	4.81E-112	5.36E-45
Rv3544c	fadE28	1.1	-1.3	1.6	2	2	3	8.31E-16	2.01E-23	2.41E-42
Rv2027c	dosT	0.0	-0.8	1.6	1	2	3	0.765048476	3.93E-09	5.76E-36
Rv1656	argF	0.4	-1.0	1.6	1	2	3	0.016024932	8.95E-10	6.72E-31
Rv2086	hypothetical protein	0.0	-2.9	1.6	1	8	3	0.861926351	6.91E-68	2.86E-25
Rv1736c	narX	-0.4	-1.1	1.6	1	2	3	0.033243027	5.14E-22	7.89E-48
Rv1852	ureG	0.3	-0.3	1.6	1	1	3	0.09109022	0.115337066	5.61E-34
Rv3542c	hypothetical protein	0.4	-0.9	1.6	1	2	3	0.010343279	1.44E-09	4.31E-38
Rv1865c	short-chain type dehydrogenase	-0.3	-0.5	1.6	1	1	3	0.175796715	0.012497611	4.27E-28
Rv2270	lppN	0.9	-1.3	1.6	2	2	3	3.90E-06	6.25E-12	2.33E-25
Rv2560	hypothetical protein	-0.2	-0.6	1.6	1	1	3	0.098719174	1.31E-05	1.07E-27

Rv3663c	dppD	0.1	-0.8	1.6	1	2	3	0.590406471	3.41E-08	1.23E-39
Rv2499c	oxidase regulatory-like protein	0.9	-2.5	1.6	2	5	3	3.31E-11	6.73E-111	7.54E-29
Rv3543c	fadE29	0.4	-1.7	1.6	1	3	3	0.005011378	1.52E-39	3.33E-42
Rv3089	fadD13	0.2	-2.4	1.6	1	5	3	0.087624221	4.35E-96	4.71E-39
Rv2686c	antibiotic ABC transporter permease	0.4	-1.8	1.6	1	4	3	0.007487718	1.75E-50	8.84E-37
Rv3655c	hypothetical protein	-1.7	-0.6	1.6	3	2	3	1.02E-07	0.026322645	9.31E-20
Rv0322	udgA	0.2	-0.2	1.6	1	1	3	0.231420933	0.121677734	6.26E-37
Rv2635	hypothetical protein	-0.3	-0.8	1.6	1	2	3	0.229289638	0.000196646	9.11E-24
Rv0838	lpqR	-0.5	0.0	1.6	1	1	3	0.000386479	0.752209568	2.47E-41
Rv2159c	hypothetical protein	-0.1	-1.4	1.6	1	3	3	0.583589927	2.90E-38	6.96E-39
Rv3546	fadA5	-0.4	-1.2	1.6	1	2	3	0.023236376	1.17E-17	6.92E-39
Rv3660c	hypothetical protein	0.0	-0.7	1.6	1	2	3	0.982340333	1.33E-08	8.78E-35
Rv1638	uvrA	0.8	-0.9	1.6	2	2	3	1.15E-14	1.59E-19	5.52E-48
Rv3817	phosphotransferase	1.0	-1.1	1.6	2	2	3	2.53E-11	2.24E-11	2.25E-33
Rv0575c	oxidoreductase	0.0	-0.8	1.6	1	2	3	0.83224175	3.11E-05	6.95E-24
Rv3026c	hypothetical protein	0.1	-0.4	1.6	1	1	3	0.563394732	0.063221423	3.16E-31
Rv1429	hypothetical protein	0.0	-1.0	1.6	1	2	3	0.937851794	1.39E-16	2.93E-44
Rv1550	fadD11	-0.7	-0.1	1.6	2	1	3	8.58E-06	0.712317137	1.67E-39
Rv1975	hypothetical protein	0.5	-1.2	1.6	1	2	3	0.006600711	7.20E-14	2.70E-32
Rv1482c	hypothetical protein	0.0	-0.8	1.6	1	2	3	0.830265481	4.20E-05	1.75E-30
Rv2269c	hypothetical protein	0.4	-1.8	1.6	1	4	3	0.097628946	5.76E-16	6.28E-15
Rv2671	ribD	-0.3	0.4	1.6	1	1	3	0.179592809	0.021877503	5.35E-38
Rv0953c	oxidoreductase	-0.9	0.0	1.6	2	1	3	1.78E-05	0.830794168	1.57E-31
Rv0152c	PE2	-0.4	0.6	1.6	1	2	3	0.006525141	1.19E-05	4.97E-45
Rv2012	hypothetical protein	0.0	-0.8	1.6	1	2	3	0.93486183	1.51E-07	7.47E-25
Rv1768	PE PGRS31	0.2	-0.6	1.6	1	2	3	0.356378509	0.000244559	1.05E-27
Rv1716	hypothetical protein	-0.6	0.3	1.6	2	1	3	0.000715592	0.081603506	3.55E-32
Rv0752c	fadE9	0.7	-1.3	1.6	2	2	3	2.57E-07	1.57E-25	1.54E-35
Rv0837c	hypothetical protein	-0.8	-1.3	1.6	2	2	3	2.99E-05	4.82E-23	1.03E-33
Rv0553	menC	-0.7	-0.5	1.6	2	1	3	4.13E-05	0.004477477	7.22E-31
Rv1004c	membrane protein	-1.4	-0.8	1.6	3	2	3	6.94E-17	7.49E-07	7.45E-40
Rv1431	hypothetical protein	0.0	-0.9	1.6	1	2	3	0.786075792	7.79E-12	4.23E-41
Rv3575c	LacI family transcriptional regulator	0.2	-0.2	1.6	1	1	3	0.267333538	0.28251249	2.32E-37
Rv1620c	cydC	1.6	-2.2	1.6	3	5	3	4.14E-27	2.43E-67	2.22E-39
Rv3912	anti-sigma-M factor RsmA	-0.9	-0.5	1.6	2	1	3	8.85E-07	0.001768239	1.57E-33
Rv1203c	hypothetical protein	0.5	-0.5	1.5	1	1	3	0.004841845	0.00499181	1.06E-29
Rv1951c	hypothetical protein	-0.3	-0.6	1.5	1	1	3	0.26623955	0.038037957	6.91E-17
Rv1727	hypothetical protein	0.6	-0.5	1.5	1	1	3	0.006071499	0.039641308	2.38E-25
Rv1263	amiB2	-0.7	-0.5	1.5	2	1	3	5.62E-05	0.00131664	2.58E-37
Rv3111	moaC1	-0.1	0.4	1.5	1	1	3	0.615275002	0.044466826	4.52E-28
Rv3015c	hypothetical protein	-0.3	-0.6	1.5	1	2	3	0.096716548	0.000205299	1.64E-30
Rv0534c	menA	-0.2	-0.1	1.5	1	1	3	0.143655752	0.521678231	5.35E-29
Rv0162c	adhE1	-0.1	0.1	1.5	1	1	3	0.724347015	0.486096451	5.68E-36
Rv1718	hypothetical protein	-0.5	0.1	1.5	1	1	3	0.003237708	0.715752283	4.70E-32
Rv3189	hypothetical protein	0.3	-1.1	1.5	1	2	3	0.038749676	3.05E-17	1.62E-39
Rv1148c	hypothetical protein	1.2	-1.7	1.5	2	3	3	2.68E-20	2.90E-52	4.94E-38

Rv2379c	mbtF	0.2	-0.9	1.5	1	2	3	0.111690037	7.92E-20	1.50E-45
Rv3350c	PPE56	0.1	-0.6	1.5	1	1	3	0.52624722	6.23E-08	3.15E-41
Rv0326	hypothetical protein	-0.1	0.0	1.5	1	1	3	0.672406567	0.882905434	9.14E-26
Rv0304c	PPE5	0.0	-0.1	1.5	1	1	3	0.793051586	0.381534617	3.08E-45
Rv1956	higA	1.0	-2.5	1.5	2	6	3	2.77E-12	1.19E-94	6.27E-35
Rv2625c	zinc metalloprotease Rip3	-0.5	-1.4	1.5	1	3	3	0.001037729	4.16E-30	4.52E-39
Rv2831	echA16	-0.9	0.2	1.5	2	1	3	8.77E-05	0.424716802	3.90E-28
Rv1358	transcriptional regulator	-0.2	-1.0	1.5	1	2	3	0.060948666	8.49E-20	1.13E-42
Rv3332	nagA	-0.8	0.0	1.5	2	1	3	2.70E-05	0.969299445	1.13E-31
Rv2162c	PE PGRS38	0.2	-0.7	1.5	1	2	3	0.07408706	4.02E-08	1.12E-23
Rv3433c	bifunctional ADP-dependent (S)-NAD(	-1.1	-0.4	1.5	2	1	3	2.40E-08	0.029867457	1.37E-34
Rv1579c	phage protein	0.6	-0.7	1.5	2	2	3	0.001209713	0.000566513	2.05E-22
Rv0776c	hypothetical protein	-1.1	-0.6	1.5	2	2	3	6.21E-07	0.000760753	1.92E-28
Rv2044c	hypothetical protein	0.8	-1.3	1.5	2	3	3	0.000334676	1.24E-10	5.83E-20
Rv0255c	cobQ1	0.1	-0.4	1.5	1	1	3	0.628287658	0.006192745	9.76E-34
Rv2123	PPE37	-0.6	-0.7	1.5	2	2	3	0.000107215	1.51E-06	1.39E-33
Rv1934c	fadE17	0.0	-0.3	1.5	1	1	3	0.97800263	0.152151691	1.09E-29
Rv0397	hypothetical protein	0.2	-0.9	1.5	1	2	3	0.343488202	7.80E-11	6.52E-23
Rv1348	irtA	0.4	-0.4	1.5	1	1	3	0.003954437	0.001083531	2.65E-41
Rv2685	arsB1	1.2	-1.1	1.5	2	2	3	1.18E-20	7.67E-23	1.46E-33
Rv2691	ceoB	-0.8	0.7	1.5	2	2	3	3.07E-07	1.75E-06	7.32E-38
Rv3851	membrane protein	-0.3	-0.1	1.5	1	1	3	0.154140994	0.668891456	2.00E-22
Rv2088	pknJ	0.5	-0.8	1.5	1	2	3	0.000542414	6.67E-10	2.16E-39
Rv2056c	rpsN2	-0.6	-0.1	1.5	1	1	3	0.041915994	0.718492881	5.32E-18
Rv0305c	PPE6	0.3	-0.7	1.5	1	2	3	0.034060004	6.64E-11	5.58E-45
Rv2823c	CRISPR-associated protein Cas10/Cs	1.1	-0.9	1.5	2	2	3	1.25E-18	3.03E-15	9.87E-39
Rv3835	hypothetical protein	-0.3	0.7	1.5	1	2	3	0.020105195	5.43E-10	1.23E-34
Rv1147	hypothetical protein	0.8	-1.6	1.5	2	3	3	1.36E-08	7.74E-39	8.33E-36
Rv2554c	Holliday junction resolvase	0.2	-1.5	1.5	1	3	3	0.309461496	1.29E-35	1.92E-37
Rv3019c	esxR	0.3	-0.2	1.5	1	1	3	0.112454439	0.443854938	1.50E-25
Rv2179c	3'-5' exoribonuclease	-0.7	-0.1	1.5	2	1	3	0.004318817	0.696401599	8.83E-22
Rv3359	oxidoreductase	0.1	-0.6	1.5	1	1	3	0.453713639	0.000784042	7.76E-31
Rv2037c	transmembrane protein	0.1	-1.8	1.5	1	4	3	0.611025629	2.38E-49	4.74E-31
Rv2252	diacylglycerol kinase	0.3	-0.6	1.5	1	2	3	0.052312585	2.00E-05	1.66E-35
Rv3561	fadD3	0.2	-0.1	1.5	1	1	3	0.303014551	0.704086581	1.31E-25
Rv0369c	membrane oxidoreductase	-0.6	-0.5	1.5	2	1	3	0.003579872	0.009876164	2.27E-25
Rv3183	transcriptional regulator	0.1	-1.5	1.5	1	3	3	0.701350559	8.23E-19	1.84E-24
Rv0106	hypothetical protein	-0.2	0.1	1.5	1	1	3	0.166665832	0.527209492	1.99E-34
Rv0826	hypothetical protein	0.9	-3.0	1.5	2	8	3	1.48E-09	1.27E-130	1.46E-31
Rv0415	thiO	-0.3	-0.7	1.5	1	2	3	0.105988542	3.35E-07	5.26E-34
Rv1027c	kdpE	0.6	-1.3	1.5	2	2	3	0.000980064	2.09E-15	4.96E-25
Rv3366	spoU	0.4	0.2	1.5	1	1	3	0.016080373	0.378639246	1.26E-23
Rv2892c	PPE45	-0.4	-0.6	1.5	1	2	3	0.022979809	0.000408313	2.93E-31
Rv2264c	hypothetical protein	-0.6	-0.6	1.5	2	2	3	8.06E-05	2.58E-05	2.47E-29
Rv0099	fadD10	0.3	-0.6	1.5	1	1	3	0.07160511	2.55E-06	6.88E-29
Rv2231A	vapC16	0.8	-0.4	1.5	2	1	3	0.000207925	0.052481459	2.16E-22

Rv1832	gcvB	0.9	-1.4	1.5	2	3	3	3.94E-11	1.06E-42	2.15E-41
Rv2862c	hypothetical protein	-0.9	-0.3	1.5	2	1	3	1.53E-05	0.129018171	3.31E-19
Rv0674	hypothetical protein	-0.4	-0.8	1.5	1	2	3	0.028800247	1.39E-07	8.08E-26
Rv0968	hypothetical protein	-0.5	-0.2	1.5	1	1	3	0.000157164	0.206939554	1.11E-39
Rv3083	FAD-containing monooxygenase Mym	0.2	-0.8	1.5	1	2	3	0.262202226	3.61E-08	6.53E-32
Rv0320	hypothetical protein	0.3	-0.1	1.5	1	1	3	0.06560283	0.77086687	2.52E-25
Rv0411c	glnH	-0.4	-1.4	1.5	1	3	3	0.000699345	4.88E-41	1.83E-33
Rv2559c	hypothetical protein	-0.2	-1.8	1.5	1	3	3	0.206037993	1.04E-43	7.29E-33
Rv0410c	pknG	-0.5	-1.3	1.5	1	2	3	7.44E-07	6.02E-27	2.75E-40
Rv3833	AraC family transcriptional regulator	-0.8	-1.0	1.5	2	2	3	1.82E-06	4.86E-11	1.10E-30
Rv1961	hypothetical protein	-0.1	-0.4	1.5	1	1	3	0.618161071	0.027351926	1.90E-25
Rv0398c	hypothetical protein	-0.2	-1.0	1.5	1	2	3	0.430069657	8.46E-08	5.17E-23
Rv0083	oxidoreductase	-1.1	-0.5	1.4	2	1	3	2.13E-11	0.245688492	3.79E-31
Rv0628c	hypothetical protein	-0.1	-1.3	1.4	1	3	3	0.415622986	7.08E-32	1.00E-34
Rv2000	hypothetical protein	0.2	0.1	1.4	1	1	3	0.333172073	0.497411363	1.57E-35
Rv0767c	HTH-type transcriptional regulator	-1.4	-0.9	1.4	3	2	3	1.44E-14	1.27E-10	1.59E-31
Rv3079c	hypothetical protein	0.1	-1.6	1.4	1	3	3	0.450535878	2.03E-37	8.62E-29
Rv3743c	ctpJ	-0.8	-0.7	1.4	2	2	3	1.18E-08	5.53E-09	4.11E-36
Rv1851	ureF	0.3	0.0	1.4	1	1	3	0.146254382	0.82872301	5.64E-25
Rv1735c	membrane protein	0.9	-3.0	1.4	2	8	3	1.43E-07	5.62E-128	7.42E-27
Rv1717	hypothetical protein	-0.7	0.0	1.4	2	1	3	0.00251241	0.967364448	7.74E-20
Rv1125	hypothetical protein	0.6	-1.0	1.4	2	2	3	1.81E-05	1.88E-13	6.49E-31
Rv3664c	dppC	-0.2	-0.4	1.4	1	1	3	0.41915168	0.054427534	9.08E-23
Rv3517	hypothetical protein	0.6	0.0	1.4	2	1	3	5.42E-05	0.960595229	2.16E-24
Rv3643	hypothetical protein	-0.1	-0.2	1.4	1	1	3	0.735359719	0.486665095	3.73E-14
Rv3195	hypothetical protein	-0.1	-0.4	1.4	1	1	3	0.731175728	0.004894793	1.00E-30
Rv3502c	3-oxoacyl-ACP reductase	1.3	-1.9	1.4	3	4	3	3.43E-21	2.66E-49	2.34E-30
Rv2282c	LysR family HTH-type transcriptional r	-0.5	-0.1	1.4	1	1	3	0.010017249	0.50732749	4.88E-27
Rv2309A	hypothetical protein	-0.7	0.1	1.4	2	1	3	0.004051518	0.717691213	1.01E-19
Rv3665c	dppB	-0.2	-0.9	1.4	1	2	3	0.387034434	3.56E-09	9.74E-29
Rv3353c	hypothetical protein	-0.4	0.0	1.4	1	1	3	0.073013083	0.932080701	1.48E-18
Rv3551	CoA-transferase subunit alpha	1.0	-0.4	1.4	2	1	3	3.11E-14	0.006556638	1.09E-30
Rv2668	hypothetical protein	0.5	-0.6	1.4	1	1	3	0.012365282	0.003334172	4.06E-21
Rv2378c	mbtG	0.2	-0.7	1.4	1	2	3	0.216548954	5.71E-07	1.98E-31
Rv2072c	cobL	-0.3	-0.4	1.4	1	1	3	0.065221314	0.013320035	2.38E-30
Rv1138a	hypothetical protein CDS	0.4	-0.2	1.4	1	1	3	0.056409822	0.356802363	1.50E-18
Rv3178	nitroreductase	-0.7	-0.6	1.4	2	2	3	0.016037517	0.007548623	4.54E-18
Rv1124	ephC	-0.1	-0.6	1.4	1	1	3	0.522107688	0.000294502	8.88E-28
Rv0330c	hypothetical protein	-0.7	-0.4	1.4	2	1	3	9.05E-05	0.007845651	1.46E-26
Rv3188	hypothetical protein	-0.6	-1.8	1.4	1	4	3	0.001352394	9.36E-42	6.40E-29
Rv3771c	hypothetical protein	0.9	-0.6	1.4	2	2	3	6.72E-07	0.000617013	8.75E-21
Rv3537	kstD	0.6	-1.1	1.4	1	2	3	1.13E-05	3.66E-21	2.77E-34
Rv2910c	hypothetical protein	-0.5	0.1	1.4	1	1	3	0.024499001	0.666043252	7.86E-22
Rv3170	aofH	-0.2	-0.8	1.4	1	2	3	0.173114421	1.21E-08	1.74E-29
Rv2765	hydrolase	-0.6	-0.1	1.4	2	1	3	0.011027455	0.455146206	1.07E-22
Rv3296	lhr	0.1	-1.0	1.4	1	2	3	0.676020155	1.34E-22	6.42E-38

Rv2483c	plsC	0.7	-1.7	1.4	2	3	3	2.65E-06	3.72E-36	6.22E-28
Rv2966c	methyltransferase	0.6	-0.6	1.4	2	2	3	0.005362615	9.34E-05	2.05E-28
Rv3387	transposase	0.3	-0.9	1.4	1	2	3	0.202830573	3.10E-06	2.15E-25
Rv2305	hypothetical protein	-0.3	-0.2	1.4	1	1	3	0.033891369	0.196750094	1.11E-29
Rv1584c	phage protein	0.5	-1.9	1.4	1	4	3	0.012135419	3.61E-33	8.44E-20
Rv2738c	hypothetical protein	-0.1	0.1	1.4	1	1	3	0.644207532	0.793238396	4.35E-22
Rv1028A	kdpF	0.3	0.0	1.4	1	1	3	0.365652457	0.930748759	2.74E-12
Rv1657	argR	0.8	-1.3	1.4	2	2	3	9.91E-05	1.66E-10	8.22E-19
Rv3070	fluoride ion transporter CrcB	0.0	-0.7	1.4	1	2	3	0.904589771	0.000144966	1.36E-22
Rv2545	vapB18	-0.3	0.0	1.4	1	1	3	0.179729858	0.988802069	3.01E-17
Rv1622c	cydB	1.3	-1.5	1.4	2	3	3	5.36E-27	1.43E-36	4.72E-31
Rv3239c	transmembrane transport protein	0.3	-0.9	1.4	1	2	3	0.008658072	1.85E-13	6.65E-37
Rv0591	mce2C	0.2	-0.7	1.4	1	2	3	0.176548213	2.97E-07	8.93E-28
Rv2570	hypothetical protein	0.5	-1.8	1.4	1	4	3	0.022751844	8.55E-18	3.13E-14
Rv0832	PE PGRS12	-0.1	-0.7	1.4	1	2	3	0.639681289	4.37E-05	4.05E-28
Rv2963	integral membrane protein	-0.2	-1.6	1.4	1	3	3	0.164773284	1.13E-39	1.66E-35
Rv3905c	esxF	1.8	-0.7	1.4	3	2	3	1.13E-20	5.82E-05	9.14E-20
Rv0614	hypothetical protein	-0.7	-0.5	1.4	2	1	3	1.44E-05	0.000668968	5.57E-25
Rv0904c	accD3	-0.2	-0.7	1.4	1	2	3	0.212457475	6.97E-07	5.49E-29
Rv2320c	rocE	-0.4	-1.3	1.4	1	3	3	0.009079063	1.47E-25	2.31E-30
Rv3712	ligase	-0.8	0.0	1.4	2	1	3	2.07E-06	0.99789959	9.59E-25
Rv1921c	lppF	0.4	-0.7	1.4	1	2	3	0.00493279	2.67E-07	1.29E-29
Rv1670	hypothetical protein	-0.4	-1.7	1.4	1	3	3	0.082613157	7.22E-21	2.05E-18
Rv1269c	hypothetical protein	0.3	-0.4	1.4	1	1	3	0.16214536	0.058563081	9.39E-20
Rv3787c	S-adenosyl-L-methionine-dependent [illegible]	-0.6	-0.4	1.4	2	1	3	5.98E-05	0.042866323	4.00E-27
Rv1165	typA	0.1	0.1	1.4	1	1	3	0.306802276	0.498774969	1.67E-35
Rv2826c	hypothetical protein	0.1	0.0	1.3	1	1	3	0.689807978	0.977331101	8.57E-30
Rv2571c	transmembrane protein	0.7	-1.0	1.3	2	2	3	2.73E-05	6.30E-09	5.32E-24
Rv2398c	cysW	1.6	-0.1	1.3	3	1	3	2.98E-28	0.574085535	1.19E-23
Rv0989c	grcC2	0.6	0.5	1.3	2	1	3	1.77E-06	1.75E-05	1.65E-27
Rv1537	dinX	0.2	-0.6	1.3	1	1	3	0.139139132	0.000414788	1.19E-23
Rv1581c	phage protein	-0.2	-0.3	1.3	1	1	3	0.367848271	0.046041118	5.36E-23
Rv0254c	cobU	1.0	-1.0	1.3	2	2	3	8.01E-06	8.37E-07	1.54E-17
Rv2964	purU	0.1	-1.2	1.3	1	2	3	0.639275245	1.93E-22	7.42E-31
Rv1129c	transcriptional regulator	-0.1	-0.2	1.3	1	1	3	0.525820497	0.129408647	3.42E-31
Rv2311	hypothetical protein	-0.1	-0.1	1.3	1	1	3	0.614510949	0.482089347	4.33E-20
Rv3472	hypothetical protein	-0.8	-0.2	1.3	2	1	3	0.000492869	0.25933545	2.44E-21
Rv1875	hypothetical protein	0.1	-0.4	1.3	1	1	3	0.47138923	0.017065315	2.19E-18
Rv2353c	PPE39	-0.3	-0.1	1.3	1	1	3	0.123498415	0.226200447	3.47E-33
Rv0764c	cyp51	0.3	-1.9	1.3	1	4	3	0.015110718	4.97E-68	2.96E-29
Rv2253	hypothetical protein	0.4	-0.9	1.3	1	2	3	0.028466867	1.27E-07	8.79E-24
Rv1259	udgB	-0.2	-0.1	1.3	1	1	3	0.181767824	0.633679868	6.25E-23
Rv0986	adhesion component ABC transporter	-0.1	0.2	1.3	1	1	3	0.352667759	0.196931216	5.33E-14
Rv3762c	hydrolase	0.6	-0.3	1.3	1	1	3	1.85E-05	0.010718035	1.06E-30
Rv0396	hypothetical protein	-0.1	0.2	1.3	1	1	3	0.79546205	0.331544263	2.22E-18
Rv2263	oxidoreductase	0.1	-0.7	1.3	1	2	3	0.69966145	6.32E-05	8.91E-25

Rv1062	hypothetical protein	0.9	-1.4	1.3	2	3	2	2.02E-08	1.08E-22	4.28E-23
Rv0132c	fgd2	0.0	-1.2	1.3	1	2	2	0.892627559	5.68E-21	9.05E-27
Rv0131c	fadE1	0.1	-1.0	1.3	1	2	2	0.464406135	5.34E-12	1.55E-21
Rv2805	hypothetical protein	-0.2	0.1	1.3	1	1	2	0.27699583	0.649887634	7.04E-18
Rv0963c	hypothetical protein	-0.4	-0.2	1.3	1	1	2	0.082904101	0.1470038	3.64E-26
Rv3792	aftA	-0.2	0.4	1.3	1	1	2	0.102293485	0.023563037	2.52E-28
Rv0872c	PE PGRS15	-0.8	-1.2	1.3	2	2	2	5.82E-08	1.09E-27	2.32E-15
Rv3180c	ribonuclease VapC45	-0.2	-1.4	1.3	1	3	2	0.336497276	2.21E-19	2.28E-22
Rv0536	galE3	-0.2	-0.2	1.3	1	1	2	0.406743899	0.302716724	1.90E-21
Rv0995	rimJ	0.3	0.1	1.3	1	1	2	0.029129778	0.499584939	2.24E-26
Rv1028c	kdpD	-0.2	-1.6	1.3	1	3	2	0.045592343	2.02E-50	3.81E-33
Rv1722	carboxylase	0.1	-0.2	1.3	1	1	2	0.681229116	0.296274631	3.17E-29
Rv2669	GCN5-like N-acetyltransferase	0.3	-0.5	1.3	1	1	2	0.037728763	0.003125879	1.74E-23
Rv3501c	yrbE4A	0.3	-0.5	1.3	1	1	2	0.079566561	0.000219703	1.42E-27
Rv1467c	fadE15	0.9	-1.4	1.3	2	3	2	3.50E-12	5.05E-37	4.13E-25
Rv3903c	hypothetical protein	-0.2	-0.4	1.3	1	1	2	0.194321084	0.002493393	2.08E-24
Rv2370c	hypothetical protein	-0.1	0.0	1.3	1	1	2	0.417392405	0.879216651	7.30E-22
Rv2251	flavoprotein	-0.8	-0.7	1.3	2	2	2	4.74E-08	9.55E-09	3.83E-26
Rv1944c	hypothetical protein	0.0	0.0	1.3	1	1	2	0.992453028	0.996857941	7.65E-18
Rv1707	transmembrane protein	-1.1	0.6	1.3	2	2	2	1.78E-18	2.52E-07	9.53E-24
Rv1790	PPE27	0.2	-1.0	1.3	1	2	2	0.262885094	1.12E-15	5.37E-28
Rv3530c	oxidoreductase	0.1	-1.3	1.3	1	2	2	0.564678974	2.16E-22	4.10E-19
Rv1112	GTP-binding protein	0.5	0.3	1.3	1	1	2	0.003066724	0.104852717	6.41E-25
Rv1278	hypothetical protein	1.0	-0.8	1.3	2	2	2	1.68E-17	4.84E-12	4.70E-32
Rv3539	PPE63	-0.2	-0.8	1.3	1	2	2	0.099802738	4.19E-11	2.23E-29
Rv2862A	vapB23	-0.4	0.7	1.3	1	2	2	0.092215903	0.012668262	1.34E-15
Rv2540c	aroF	-1.1	-0.6	1.3	2	2	2	2.42E-06	4.14E-07	4.09E-18
Rv1276c	hypothetical protein	0.9	-0.4	1.3	2	1	2	2.23E-05	0.05642775	8.18E-17
Rv0217c	lipW	-0.1	-0.1	1.3	1	1	2	0.437129517	0.782060753	2.36E-24
Rv0377	HTH-type transcriptional regulator	-1.2	-0.2	1.3	2	1	2	1.68E-14	0.107028449	2.51E-29
Rv2912c	TetR family HTH-type transcriptional r	0.0	-0.3	1.3	1	1	2	0.919354279	0.152022398	8.92E-21
Rv2497c	bkdA	0.8	-2.4	1.3	2	5	2	5.31E-09	6.63E-128	5.62E-27
Rv1705c	PPE22	0.0	-0.5	1.3	1	1	2	0.795819058	0.000180741	4.01E-25
Rv2352c	PPE38	0.4	-0.3	1.3	1	1	2	0.367303589	0.028112785	1.95E-13
Rv0318c	integral membrane protein	-0.7	0.4	1.3	2	1	2	0.000160785	0.015090548	4.87E-23
Rv3014c	ligA	-0.4	0.2	1.3	1	1	2	0.002844069	0.249273318	2.80E-29
Rv2156c	murX	0.0	-0.2	1.3	1	1	2	0.738331372	0.031493615	3.19E-29
Rv0389	purT	-0.3	-0.5	1.3	1	1	2	0.049035709	0.000166156	2.43E-24
Rv3554	fdxB	0.6	-0.3	1.3	2	1	2	5.70E-06	0.012782869	6.40E-30
Rv0359	zinc metalloprotease	-0.3	0.1	1.3	1	1	2	0.199210164	0.757940602	3.46E-21
Rv0880	HTH-type transcriptional regulator	-0.9	0.5	1.3	2	1	2	4.79E-05	0.007314005	4.33E-21
Rv3166c	hypothetical protein	-0.4	-0.4	1.3	1	1	2	0.008651843	0.009380392	4.20E-22
Rv2268c	cyp128	-0.1	-0.2	1.3	1	1	2	0.60261911	0.145782372	1.06E-26
Rv2489c	hypothetical protein	-0.5	-0.3	1.3	1	1	2	0.047453146	0.206332543	1.51E-11
Rv2601	speE	0.8	-0.1	1.3	2	1	2	1.16E-07	0.688227379	1.43E-25
Rv3481c	integral membrane protein	-0.1	0.1	1.3	1	1	2	0.688174217	0.531374954	3.63E-23

Rv3705A	hypothetical protein	-0.2	-0.3	1.3	1	1	2	0.462184298	0.113710633	4.94E-15
Rv3342	methyltransferase	-0.8	-0.8	1.3	2	2	2	1.67E-05	5.22E-07	1.32E-20
Rv0414c	thiE	-0.6	-0.6	1.3	2	1	2	0.006136319	0.005862217	6.40E-18
Rv2439c	proB	0.3	-0.1	1.3	1	1	2	0.102187825	0.8152397	4.27E-18
Rv3545c	cyp125	0.7	-1.1	1.3	2	2	2	3.01E-08	3.10E-19	4.33E-27
Rv2707	hypothetical protein	0.3	-0.3	1.3	1	1	2	0.012659438	0.003425428	9.73E-19
Rv2623	TB31.7	1.1	-1.1	1.2	2	2	2	1.92E-19	4.50E-20	1.86E-27
Rv0226c	transmembrane protein	-0.3	-0.1	1.2	1	1	2	0.024527812	0.686174428	6.02E-27
Rv2824c	CRISPR-associated endoribonuclease	0.7	-1.2	1.2	2	2	2	2.23E-05	1.03E-17	3.87E-22
Rv2504c	scoA	-0.2	-0.3	1.2	1	1	2	0.213511069	0.021621582	2.75E-30
Rv0209	hypothetical protein	-1.0	-0.3	1.2	2	1	2	1.74E-08	0.095371357	1.26E-20
Rv1266c	pknH	-0.1	-0.5	1.2	1	1	2	0.455253547	1.22E-06	4.42E-32
Rv2999	lppY	0.1	0.0	1.2	1	1	2	0.422358694	0.899129376	1.44E-22
Rv1221	sigE	0.1	-0.7	1.2	1	2	2	0.492125288	1.34E-10	3.29E-28
Rv0335c	PE6	-0.3	-0.5	1.2	1	1	2	0.084110005	0.00534388	4.91E-19
Rv2695	hypothetical protein	-0.9	-0.5	1.2	2	1	2	1.91E-05	0.007581747	7.71E-19
Rv3298c	lpqC	0.0	-0.5	1.2	1	1	2	0.769313794	0.004237167	3.03E-24
Rv0585c	integral membrane protein	-0.4	-0.2	1.2	1	1	2	0.00086557	0.053908069	1.37E-29
Rv3327	transposase fusion protein	-0.1	-0.2	1.2	1	1	2	0.244521101	0.139206182	7.40E-29
Rv2281	pitB	1.5	-1.4	1.2	3	3	2	5.58E-35	5.46E-35	3.41E-26
Rv0297	PE PGRS5	-0.9	-0.6	1.2	2	2	2	3.57E-12	5.07E-07	1.19E-24
Rv1214c	PE14	0.3	-0.8	1.2	1	2	2	0.092822463	3.69E-05	2.11E-16
Rv0263c	hypothetical protein	0.2	-0.3	1.2	1	1	2	0.30636258	0.059812978	8.95E-22
Rv2962c	PGL/p-HBAD biosynthesis rhamnosylt	-0.3	-0.3	1.2	1	1	2	0.050232837	0.038847929	5.03E-23
Rv3196	hypothetical protein	-0.3	0.0	1.2	1	1	2	0.136022614	0.882500672	2.33E-19
Rv0943c	monooxygenase	-1.5	0.1	1.2	3	1	2	1.84E-09	0.71743559	2.77E-13
Rv0944	formamidopyrimidine-DNA glycosylas	-1.7	0.2	1.2	3	1	2	1.67E-08	0.418530135	1.64E-15
Rv0807	hypothetical protein	-0.6	-0.9	1.2	1	2	2	0.00946078	6.40E-07	4.44E-18
Rv3331	sugI	-0.4	-0.2	1.2	1	1	2	0.013325965	0.233084806	1.27E-26
Rv0847	lpqS	-2.2	-2.7	1.2	5	7	2	2.03E-15	1.56E-77	1.06E-23
Rv1384	carB	0.0	-0.2	1.2	1	1	2	0.999406056	0.068469621	2.35E-26
Rv2731	hypothetical protein	0.0	-1.0	1.2	1	2	2	0.753373496	2.25E-17	8.07E-26
Rv0800	pepC	-0.1	-0.1	1.2	1	1	2	0.730779584	0.742228111	2.27E-24
Rv1599	hisD	0.5	-1.3	1.2	1	2	2	6.72E-05	2.90E-30	8.68E-29
Rv0604	lpqO	-0.5	0.0	1.2	1	1	2	0.006645704	0.866512744	3.46E-18
Rv2569c	hypothetical protein	-0.2	-0.5	1.2	1	1	2	0.159722315	0.000784505	2.70E-22
Rv2341	lppQ	0.1	-0.1	1.2	1	1	2	0.647264554	0.477879582	6.51E-16
Rv1277	hypothetical protein	1.2	-0.9	1.2	2	2	2	6.06E-13	1.14E-15	1.35E-20
Rv3294c	hypothetical protein	1.0	-0.3	1.2	2	1	2	2.64E-10	0.028799766	9.86E-17
Rv2652c	prophage protein	-0.8	-1.2	1.2	2	2	2	1.87E-05	4.25E-15	4.74E-18
Rv0323c	hypothetical protein	0.9	-0.5	1.2	2	1	2	1.14E-08	0.001375348	2.77E-20
Rv3063	cstA	0.0	0.0	1.2	1	1	2	0.745905818	0.879955844	1.87E-25
Rv3729	transferase	-0.3	-0.2	1.2	1	1	2	0.006188378	0.109952269	1.21E-29
Rv1601	hisB	0.7	-0.8	1.2	2	2	2	8.98E-09	1.87E-15	1.19E-18
Rv3292	hypothetical protein	1.4	-0.9	1.2	3	2	2	2.86E-28	2.09E-10	7.26E-23
Rv0927c	oxidoreductase	-0.7	0.2	1.2	2	1	2	4.34E-05	0.21943937	2.31E-21

Rv0380c	RNA methyltransferase	-0.4	0.4	1.2	1	1	2	0.059807034	0.020144514	8.67E-21
Rv1385	pyrF	0.0	-0.4	1.2	1	1	2	0.828041095	0.041393438	6.55E-21
Rv2718c	nrdR	0.6	-0.8	1.2	2	2	2	4.09E-07	1.82E-12	3.35E-16
Rv3580c	cysS1	0.1	-0.4	1.2	1	1	2	0.378881103	0.00092077	5.70E-30
Rv0290	eccD3	0.1	0.0	1.2	1	1	2	0.345263595	0.885441062	9.49E-25
Rv3071	hypothetical protein	0.2	-0.8	1.2	1	2	2	0.194182134	5.69E-09	4.11E-22
Rv1432	dehydrogenase	-0.2	0.1	1.2	1	1	2	0.240905092	0.45066432	3.72E-23
Rv2711	ideR	-0.9	0.0	1.2	2	1	2	2.36E-09	0.839967794	1.54E-25
Rv1742	hypothetical protein	-0.4	-0.5	1.2	1	1	2	0.018339906	0.001935593	5.47E-17
Rv2423	hypothetical protein	-0.7	0.2	1.2	2	1	2	0.00025534	0.188281793	6.25E-24
Rv1460	transcriptional regulator	-1.7	-1.8	1.2	3	3	2	3.18E-21	1.85E-57	5.99E-26
Rv2801c	mazF9	0.2	-0.5	1.2	1	1	2	0.263414309	0.014078417	4.14E-14
Rv2249c	glpD1	-1.4	0.1	1.2	3	1	2	5.47E-17	0.631363317	1.88E-23
Rv0216	hydratase	0.2	-0.1	1.2	1	1	2	0.267295045	0.432645301	3.25E-23
Rv1329c	dinG	-0.2	-0.2	1.2	1	1	2	0.089073239	0.205107655	6.04E-25
Rv0252	nirB	1.9	-0.7	1.2	4	2	2	9.14E-64	3.81E-09	5.33E-27
Rv2528c	mrr	0.5	-0.3	1.2	1	1	2	0.003527532	0.027943765	7.75E-21
Rv0448c	hypothetical protein	-0.1	-0.3	1.2	1	1	2	0.388686407	0.049885365	1.33E-20
Rv0593	lprL	0.1	-0.3	1.2	1	1	2	0.634161817	0.026419137	1.38E-24
Rv2973c	recG	0.3	0.0	1.2	1	1	2	0.007890261	0.848521046	3.88E-27
Rv0797	insertion sequence element IS1547 tr	-0.1	-0.2	1.2	1	1	2	0.432863263	0.054977753	1.73E-27
Rv0662c	vapB7	-0.7	-0.6	1.2	2	1	2	0.016381549	0.051717328	2.33E-09
Rv1518	hypothetical protein	-0.2	-0.3	1.2	1	1	2	0.275955186	0.074747936	2.52E-20
Rv2637	dedA	-0.7	0.4	1.2	2	1	2	6.20E-05	0.035352328	2.35E-20
Rv1517	hypothetical protein	-0.2	-0.1	1.2	1	1	2	0.333814736	0.678202068	1.26E-15
Rv0458	aldehyde dehydrogenase	-0.6	-0.3	1.2	2	1	2	4.81E-05	0.044392994	1.18E-21
Rv2566	hypothetical protein	-0.5	-0.3	1.2	1	1	2	0.000160054	0.026434961	6.96E-27
Rv2170	GCN5-like N-acetyltransferase	-0.2	-0.2	1.2	1	1	2	0.369202669	0.393193539	5.06E-18
Rv1064c	lpqV	-1.1	-0.7	1.2	2	2	2	2.98E-06	0.000206952	2.31E-14
Rv2874	dipZ	0.0	-0.8	1.2	1	2	2	0.881944789	5.44E-11	2.32E-25
Rv3746c	PE34	-0.1	-0.8	1.2	1	2	2	0.712186062	2.48E-05	2.72E-14
Rv1725c	hypothetical protein	0.1	-1.7	1.2	1	3	2	0.650519221	1.29E-33	1.24E-18
Rv0688	ferredoxin reductase	-0.2	-0.1	1.2	1	1	2	0.202522023	0.37751059	8.29E-25
Rv1456c	antibiotic ABC transporter permease	-0.1	-0.1	1.2	1	1	2	0.543191093	0.387747618	7.53E-19
Rv2039c	sugar ABC transporter permease	-0.3	-0.5	1.2	1	1	2	0.104651172	9.52E-05	2.55E-22
Rv3577	hypothetical protein	-0.6	0.3	1.2	2	1	2	9.15E-05	0.057765278	3.39E-22
Rv0399c	lpqK	1.0	-0.2	1.2	2	1	2	3.49E-10	0.068957365	4.20E-21
Rv2051c	ppm1	0.3	0.3	1.2	1	1	2	0.018905688	0.002924529	4.22E-26
Rv0078	transcriptional regulator	-0.4	-0.7	1.2	1	2	2	0.023570422	2.40E-05	5.44E-12
Rv2684	arsA	0.9	-0.3	1.2	2	1	2	2.95E-14	0.009631805	5.86E-19
Rv2863	vapC23	-0.5	0.5	1.2	1	1	2	0.012659537	0.023256474	2.55E-13
Rv2236c	cobD	-0.2	-0.8	1.2	1	2	2	0.339280277	2.78E-07	2.03E-19
Rv1002c	dolichyl-phosphate-mannose--proteir	0.0	-0.8	1.2	1	2	2	0.973074514	7.57E-13	1.82E-25
Rv2943	insertion sequence element IS1533 tr	-0.6	0.3	1.2	1	1	2	0.001720864	0.113017664	6.12E-19
Rv3011c	gatA	0.1	0.0	1.2	1	1	2	0.355539228	0.909240614	6.10E-21
Rv0874c	hypothetical protein	-0.1	-1.5	1.2	1	3	2	0.652493167	2.79E-39	4.11E-23

Rv3177	peroxidase	0.0	-0.5	1.2	1	1	2	0.872017673	0.000278131	1.82E-17
Rv0085	hycP	-1.2	0.3	1.1	2	1	2	1.58E-10	0.152284016	1.85E-19
Rv2626c	hrp1	1.1	-1.1	1.1	2	2	2	3.18E-14	7.50E-14	3.73E-16
Rv1890c	hypothetical protein	0.4	-0.6	1.1	1	2	2	0.022136627	5.71E-06	6.68E-21
Rv1012	hypothetical protein	-0.5	0.2	1.1	1	1	2	0.042695858	0.559862158	1.71E-10
Rv1563c	treY	0.0	0.5	1.1	1	1	2	0.843606337	5.56E-05	1.11E-24
Rv3713	cobQ2	0.0	0.4	1.1	1	1	2	0.856512262	0.093215675	8.00E-18
Rv0141c	hypothetical protein	0.5	-0.4	1.1	1	1	2	4.89E-06	0.000583704	2.31E-14
Rv1761c	hypothetical protein	0.9	-0.6	1.1	2	2	2	3.54E-06	0.000684953	4.35E-12
Rv2622	methyltransferase	-0.4	0.3	1.1	1	1	2	0.026852951	0.142356262	5.88E-16
Rv1867	hypothetical protein	0.2	-0.7	1.1	1	2	2	0.217162321	3.09E-08	7.16E-22
Rv2498c	citE	1.2	-3.0	1.1	2	8	2	3.27E-16	6.62E-190	2.84E-20
Rv2286c	hypothetical protein	0.1	-0.8	1.1	1	2	2	0.536114429	8.45E-07	1.09E-17
Rv3578	arsB2	0.1	0.1	1.1	1	1	2	0.702014366	0.35736708	1.38E-22
Rv2924c	fpg	-0.5	-0.7	1.1	1	2	2	0.008272604	7.20E-09	1.87E-17
Rv3550	echA20	0.4	-0.5	1.1	1	1	2	0.033422917	0.012612954	1.38E-14
Rv0561c	oxidoreductase	0.1	-0.4	1.1	1	1	2	0.330060387	0.001124431	2.99E-22
Rv3398c	idsA1	-1.3	0.3	1.1	2	1	2	4.89E-13	0.045006698	4.47E-18
Rv0576	transcriptional regulator	-0.4	-0.6	1.1	1	2	2	0.005923065	1.05E-06	3.41E-23
Rv0253	nirD	1.8	-0.7	1.1	3	2	2	5.93E-25	0.000337777	1.87E-14
Rv0790c	hypothetical protein	0.4	-0.4	1.1	1	1	2	0.013226185	0.003548378	1.00E-20
Rv0584	glycosidase	-0.1	-0.2	1.1	1	1	2	0.308424783	0.098314151	1.52E-24
Rv2539c	aroK	-0.3	-0.4	1.1	1	1	2	0.141309215	0.005272661	4.95E-18
Rv3076	hypothetical protein	-0.2	-0.4	1.1	1	1	2	0.226320449	0.042919634	2.85E-14
Rv1998c	hypothetical protein	0.5	-1.3	1.1	1	2	2	0.002328044	3.63E-18	7.60E-17
Rv3314c	deoA	0.1	-0.1	1.1	1	1	2	0.385834923	0.651702617	3.53E-20
Rv2481c	hypothetical protein	0.8	-1.2	1.1	2	2	2	0.000382427	3.99E-08	3.54E-10
Rv3107c	agpS	-0.1	0.0	1.1	1	1	2	0.673159487	0.764923627	2.50E-20
Rv1556	HTH-type transcriptional regulator	-0.3	-0.3	1.1	1	1	2	0.054664466	0.033485046	5.94E-19
Rv2036	hypothetical protein	-0.9	-1.8	1.1	2	3	2	2.05E-06	1.30E-32	1.60E-16
Rv3347c	PPE55	0.1	-0.6	1.1	1	1	2	0.637509502	1.37E-08	2.43E-23
Rv2414c	hypothetical protein	-0.6	-0.7	1.1	1	2	2	0.000107277	4.67E-05	6.75E-20
Rv1605	hisF	0.1	-0.7	1.1	1	2	2	0.517278356	4.67E-08	1.05E-20
Rv1737c	narK2	0.5	-0.8	1.1	1	2	2	0.001135509	1.73E-08	2.12E-20
Rv3308	pmmB	0.1	0.1	1.1	1	1	2	0.630627045	0.620838967	1.22E-18
Rv2714	hypothetical protein	-0.2	-0.2	1.1	1	1	2	0.102090488	0.128339737	1.15E-18
Rv0804	hypothetical protein	0.1	-0.2	1.1	1	1	2	0.536615782	0.153807535	3.23E-16
Rv1066	hypothetical protein	-1.8	-2.0	1.1	3	4	2	4.50E-11	5.42E-39	2.46E-13
Rv1251c	hypothetical protein	0.1	0.4	1.1	1	1	2	0.340776035	0.008083912	7.69E-24
Rv3109	moaA1	-0.8	0.5	1.1	2	1	2	0.000349617	0.005545288	1.98E-14
Rv2333c	stp	0.0	-0.1	1.1	1	1	2	0.769439705	0.294992378	1.57E-22
Rv1288	hypothetical protein	-0.3	-0.2	1.1	1	1	2	0.024226342	0.181970103	2.17E-18
Rv1733c	transmembrane protein	0.3	-0.2	1.1	1	1	2	0.017755029	0.11965084	1.24E-18
Rv0964c	hypothetical protein	0.1	0.0	1.1	1	1	2	0.412345237	0.843187679	7.81E-16
Rv2893	oxidoreductase	0.0	-0.6	1.1	1	2	2	0.8830234	6.33E-05	5.16E-16
Rv0214	fadD4	0.3	-1.2	1.1	1	2	2	0.006540788	7.49E-24	3.85E-22

Rv0488	amino acid transporter	0.3	-0.7	1.1	1	2	2	0.048829415	2.15E-06	1.55E-15
Rv2078	hypothetical protein	-0.5	0.4	1.1	1	1	2	0.028762228	0.122872225	2.74E-12
Rv3831	hypothetical protein	0.9	-0.4	1.1	2	1	2	8.01E-11	0.006559148	3.86E-15
Rv3463	hypothetical protein	-0.1	0.3	1.1	1	1	2	0.641471865	0.125163906	1.27E-15
Rv3355c	integral membrane protein	0.1	-0.4	1.1	1	1	2	0.654507881	0.006943262	5.99E-12
Rv3375	amiD	-0.2	0.4	1.1	1	1	2	0.243142093	0.024273978	3.44E-19
Rv1347c	mbtK	0.2	-0.8	1.1	1	2	2	0.296430079	2.79E-07	3.90E-17
Rv2464c	DNA glycosylase	-0.1	1.1	1.1	1	2	2	0.685019909	1.84E-14	2.17E-21
Rv2651c	prophage protease	-1.2	-1.7	1.1	2	3	2	3.63E-16	1.97E-07	1.40E-16
Rv3297	nei	-0.1	-1.3	1.1	1	3	2	0.351777305	2.02E-26	6.92E-18
Rv2113	integral membrane protein	-0.2	-0.2	1.1	1	1	2	0.171079246	0.347365863	3.86E-18
Rv1191	hypothetical protein	-0.7	-0.5	1.1	2	1	2	0.000117898	0.015752156	2.35E-15
Rv2030c	hypothetical protein	1.4	-1.4	1.1	3	3	2	2.61E-16	2.37E-31	1.47E-18
Rv1286	adenylyl-sulfate kinase	0.7	-1.3	1.1	2	3	2	1.35E-07	1.76E-36	1.58E-17
Rv3788	hypothetical protein	0.0	0.0	1.1	1	1	2	0.858002072	0.970761109	2.38E-16
Rv2063	mazE7	-1.8	0.0	1.1	3	1	2	4.02E-08	0.884423293	5.37E-09
Rv0130	htdZ	-0.2	-0.5	1.1	1	1	2	0.295972129	0.018136961	2.10E-12
Rv3439c	hypothetical protein	-0.1	0.1	1.1	1	1	2	0.300519583	0.331014242	5.35E-18
Rv1619	hypothetical protein	1.8	-1.6	1.1	4	3	2	4.56E-49	5.30E-42	2.87E-18
Rv1123c	bpoB	0.8	-0.2	1.1	2	1	2	1.60E-09	0.087266283	7.82E-19
Rv0772	purD	0.2	-0.2	1.1	1	1	2	0.173105019	0.195952707	3.75E-19
Rv0329c	hypothetical protein	-0.1	-1.2	1.1	1	2	2	0.755337307	3.44E-17	2.52E-15
Rv0360c	hypothetical protein	-0.6	0.4	1.1	2	1	2	0.006348238	0.106896584	3.47E-13
Rv1753c	PPE24	0.5	-0.6	1.1	1	2	2	0.013389275	4.44E-09	4.94E-17
Rv2828c	hypothetical protein	0.0	-0.6	1.1	1	1	2	0.864092692	7.95E-06	2.31E-19
Rv0753c	mmsA	0.2	-1.4	1.1	1	3	2	0.125478619	9.49E-42	7.96E-22
Rv0355c	PPE8	0.5	-0.1	1.1	1	1	2	0.000212417	0.244467302	2.86E-21
Rv0093c	membrane protein	0.5	-0.6	1.1	1	2	2	0.000291793	3.09E-05	9.83E-18
Rv2754c	thyX	-0.2	0.5	1.1	1	1	2	0.109478189	0.004266511	1.45E-18
Rv0456c	echA2	-0.4	0.0	1.1	1	1	2	0.050700781	0.865853334	4.84E-17
Rv2191	hypothetical protein	0.8	-0.3	1.1	2	1	2	2.24E-09	0.049652886	7.26E-19
Rv3165c	hypothetical protein	-0.2	-0.2	1.1	1	1	2	0.273254401	0.2795797	3.10E-13
Rv1940	ribA1	-0.9	-0.2	1.1	2	1	2	5.67E-05	0.214906688	2.36E-16
Rv1120c	hypothetical protein	-0.6	0.0	1.1	2	1	2	0.001497119	0.900944651	4.53E-14
Rv2977c	thiL	0.5	-1.0	1.1	1	2	2	0.006424795	9.08E-15	1.52E-18
Rv2408	PE24	0.5	-0.5	1.1	1	1	2	0.005940314	0.005658221	7.54E-16
Rv3106	fprA	-0.6	0.0	1.1	2	1	2	8.65E-05	0.827451484	2.44E-18
Rv3290c	lat	1.4	-3.1	1.0	3	9	2	6.92E-14	7.51E-26	2.71E-11
Rv0151c	PE1	-0.4	0.2	1.0	1	1	2	0.001206985	0.036503083	1.04E-21
Rv0283	eccB3	-0.3	0.2	1.0	1	1	2	0.060908026	0.043085457	2.87E-23
Rv3421c	hypothetical protein	-0.3	-0.2	1.0	1	1	2	0.106469462	0.203326714	4.14E-14
Rv3176c	mesT	-2.1	0.9	1.0	4	2	2	4.40E-31	8.03E-09	2.58E-14
Rv1344	mbtL	-0.5	0.3	1.0	1	1	2	0.007467768	0.239119073	1.97E-11
Rv0917	betP	-0.6	-0.1	1.0	2	1	2	8.33E-05	0.478845785	1.31E-19
Rv3346c	transmembrane protein	0.3	-0.5	1.0	1	1	2	0.154988066	0.000144773	2.05E-17
Rv2153c	murG	-1.0	-0.3	1.0	2	1	2	6.28E-07	0.059192079	4.48E-17

Rv2496c	bkdB	0.2	-1.9	1.0	1	4	2	0.062196675	1.55E-88	4.95E-20
Rv2058c	rpmB2	-1.6	0.3	1.0	3	1	2	6.29E-08	0.245527994	2.15E-09
Rv2729c	integral membrane protein	-0.5	-0.6	1.0	1	1	2	0.000607975	6.26E-07	5.42E-21
Rv0231	fadE4	0.0	0.4	1.0	1	1	2	0.944602587	0.000707151	9.57E-21
Rv3351c	hypothetical protein	-0.9	0.3	1.0	2	1	2	2.03E-05	0.209126447	3.38E-15
Rv2806	membrane protein	-0.5	0.2	1.0	1	1	2	0.041379743	0.355605994	7.93E-10
Rv1519	hypothetical protein	0.0	0.2	1.0	1	1	2	0.816201408	0.446154799	2.24E-12
Rv1663	pks17	-0.3	0.1	1.0	1	1	2	0.189778831	0.494324366	1.54E-12
Rv0978c	PE PGRS17	-0.9	0.0	1.0	2	1	2	1.28E-05	0.965053268	1.69E-13
Rv3432c	gadB	-0.2	-0.1	1.0	1	1	2	0.070542821	0.509255005	3.73E-18
Rv2627c	hypothetical protein	0.9	-1.1	1.0	2	2	2	4.72E-10	1.02E-19	1.87E-17
Rv3886c	mycP2	-0.4	-0.3	1.0	1	1	2	0.015052078	0.028577614	8.37E-17
Rv2116	lppK	0.1	-0.3	1.0	1	1	2	0.406730323	0.106274013	1.18E-14
Rv2032	acg	1.1	-0.9	1.0	2	2	2	4.60E-14	2.05E-09	7.77E-15
Rv2922A	acyP	0.9	-2.4	1.0	2	5	2	3.36E-05	7.85E-38	1.17E-08
Rv3797	fadE35	-0.6	0.0	1.0	1	1	2	5.33E-05	0.706782166	1.25E-18
Rv0120c	fusA2	0.5	-0.8	1.0	1	2	2	0.000173717	3.38E-12	6.62E-19
Rv2230c	GTP cyclohydrolase	0.6	0.0	1.0	2	1	2	1.78E-05	0.753602365	2.83E-15
Rv1604	impA	0.3	-0.6	1.0	1	2	2	0.042414511	1.12E-07	3.84E-21
Rv0699	hypothetical protein	-0.5	0.7	1.0	1	2	2	0.030633304	0.000817992	6.84E-13
Rv0499	hypothetical protein	-0.5	0.2	1.0	1	1	2	0.008161575	0.304601495	6.86E-14
Rv0784	hypothetical protein	0.3	-2.3	1.0	1	5	2	0.044868063	1.86E-64	7.62E-14
Rv2767c	membrane protein	0.1	-0.2	1.0	1	1	2	0.567566743	0.428581547	1.93E-10
Rv2875	mpt70	0.4	-0.8	1.0	1	2	2	0.005361796	1.92E-07	3.13E-15
Rv2307c	hypothetical protein	0.3	-1.0	1.0	1	2	2	0.084357629	1.92E-14	1.32E-16
Rv0552	hypothetical protein	0.0	-0.2	1.0	1	1	2	0.984862267	0.277827908	7.61E-18
Rv0107c	ctpI	0.4	0.2	1.0	1	1	2	0.000566172	0.179206201	1.11E-21
Rv0086	hycQ	-1.2	0.5	1.0	2	1	2	2.60E-17	0.004176835	2.02E-17
Rv2787	hypothetical protein	0.5	0.0	1.0	1	1	2	0.0001577	0.867000694	4.14E-19
Rv1119c	hypothetical protein	0.0	-0.3	1.0	1	1	2	0.920627501	0.348867205	1.49E-06
Rv1659	argH	-0.2	-0.4	1.0	1	1	2	0.118441485	0.005549812	6.84E-17
Rv3227	aroA	-0.5	-0.2	1.0	1	1	2	0.000136591	0.087330114	1.88E-17
Rv0271c	fadE6	0.6	-1.9	1.0	1	4	2	1.69E-06	3.02E-65	1.22E-19
Rv3364c	hypothetical protein	0.6	0.2	1.0	2	1	2	0.003655659	0.363953257	1.12E-09
Rv0672	fadE8	0.5	-1.8	1.0	1	3	2	0.000256986	7.89E-56	1.07E-17
Rv1330c	pncB1	-0.3	-0.7	1.0	1	2	2	0.08118261	4.62E-08	3.58E-16
Rv0802c	succinyl-CoA transferase	0.1	-0.5	1.0	1	1	2	0.66807349	0.00038374	5.21E-14
Rv3618	monooxygenase	0.0	-0.2	1.0	1	1	2	0.826746077	0.25741135	9.31E-17
Rv3503c	fdxD	1.2	-1.5	1.0	2	3	2	1.14E-08	1.30E-28	6.28E-11
Rv1235	lpqY	0.8	-0.9	1.0	2	2	2	5.29E-10	1.83E-15	1.38E-17
Rv3293	pcd	1.3	-0.8	1.0	2	2	2	7.86E-25	1.56E-11	5.39E-17
Rv2873	mpt83	1.4	-1.0	1.0	3	2	2	1.09E-18	2.32E-12	3.35E-12
Rv1664	pks9	0.1	-0.2	1.0	1	1	2	0.622409678	0.170623444	8.81E-13
Rv3625c	mesJ	0.3	-1.2	1.0	1	2	2	0.120916063	6.05E-21	3.18E-16
Rv0763c	ferredoxin	0.2	-1.7	1.0	1	3	2	0.531855758	7.85E-14	5.10E-07
Rv0621	membrane protein	-0.5	-0.6	1.0	1	2	2	0.003686917	2.27E-05	1.11E-15

Rv0353	hspR	0.3	-0.5	1.0	1	1	2	0.028865351	3.76E-06	1.12E-18
Rv0442c	PPE10	0.2	-0.5	1.0	1	1	2	0.347083121	6.29E-05	8.32E-19
Rv2552c	aroE	-0.8	-1.2	1.0	2	2	2	1.69E-07	7.89E-22	3.73E-13
Rv3666c	dppA	-0.3	-0.7	1.0	1	2	2	0.01380589	2.53E-08	9.00E-18
Rv1907c	hypothetical protein	2.0	-1.6	1.0	4	3	2	2.87E-56	1.40E-43	2.55E-13
Rv3571	kshB	-0.7	-0.5	1.0	2	1	2	4.29E-06	0.000204415	1.83E-17
Rv2800	hydrolase	-1.3	-0.2	1.0	2	1	2	6.37E-18	0.108137158	2.28E-15
Rv2822c	CRISPR type III-associated protein Cs	2.3	-1.0	1.0	5	2	2	6.13E-48	3.51E-08	3.24E-12
Rv3838c	pheA	-0.7	-0.4	1.0	2	1	2	0.000695605	0.002360061	3.54E-19
Rv1285	cysD	-0.2	-1.3	1.0	1	2	2	0.214488342	1.50E-23	1.47E-15
Rv1745c	idi	1.0	-1.4	1.0	2	3	2	1.36E-11	1.14E-21	1.73E-13
Rv2923c	hypothetical protein	0.2	-0.5	1.0	1	1	2	0.464504434	0.003408811	4.35E-10
Rv0264c	hypothetical protein	-0.1	-0.1	1.0	1	1	2	0.769008723	0.446597454	6.88E-14
Rv0195	two component transcriptional regula	0.6	-1.5	1.0	2	3	2	0.002549851	3.65E-19	6.42E-09
Rv0967	csoR	-1.3	0.6	1.0	2	2	2	1.31E-23	1.10E-08	6.16E-14
Rv0551c	fadD8	0.0	0.1	1.0	1	1	2	0.932080328	0.34771853	1.68E-17
Rv1514c	glycosyltransferase	0.9	0.4	1.0	2	1	2	7.79E-08	0.034227224	2.09E-14
Rv2829c	vapC22	-0.2	-0.2	1.0	1	1	2	0.418936414	0.118438389	2.39E-11
Rv3522	ltp4	-0.3	0.1	1.0	1	1	2	0.039569512	0.516016497	7.17E-14
Rv0327c	cyp135A1	-1.0	0.4	1.0	2	1	2	6.74E-09	0.001065669	6.31E-17
Rv2825c	hypothetical protein	-0.1	-0.5	1.0	1	1	2	0.405845259	2.38E-05	9.48E-18
Rv2415c	hypothetical protein	-0.5	-0.3	1.0	1	1	2	0.00524486	0.087272105	2.79E-13
Rv3262	fbiB	1.0	0.1	1.0	2	1	2	1.69E-11	0.367334133	6.57E-16
Rv2038c	sugar ABC transporter ATP-binding pro	-0.2	-0.5	1.0	1	1	2	0.260979738	0.000419343	1.24E-13
Rv3110	moaB1	-0.7	0.7	1.0	2	2	2	0.003658548	0.006247339	8.86E-10
Rv1527c	pks5	-0.2	-0.4	1.0	1	1	2	0.140177655	0.000105818	8.89E-20
Rv0669c	neutral ceramidase	-0.1	0.9	1.0	1	2	2	0.465812386	5.18E-17	9.43E-15
Rv0233	nrdB	1.0	-1.8	1.0	2	3	2	3.39E-11	5.00E-50	1.98E-15
Rv0037c	MFS-type transporter	0.0	-0.6	1.0	1	2	2	0.875588389	5.50E-08	5.57E-17
Rv2864c	penicillin-binding lipoprotein	-0.2	0.1	1.0	1	1	2	0.132809682	0.41758948	5.60E-16
Rv2401A	membrane protein	-0.3	-0.3	1.0	1	1	2	0.139354053	0.239244902	2.91E-09
Rv3652	PE PGRS60	0.2	-0.3	1.0	1	1	2	0.389916709	0.164781207	3.07E-10
Rv2340c	PE PGRS39	-0.2	-1.0	1.0	1	2	2	0.235265959	2.26E-18	1.16E-09
Rv0751c	mmsB	-0.1	-0.8	1.0	1	2	2	0.458582134	2.70E-09	1.12E-14
Rv2363	amiA2	-0.5	0.0	1.0	1	1	2	0.003318905	0.979800704	3.28E-15
Rv3033	hypothetical protein	0.3	0.5	1.0	1	1	2	0.058122709	0.011612589	7.61E-11
Rv2732c	transmembrane protein	0.1	-0.4	1.0	1	1	2	0.454493939	0.004095685	7.85E-15
Rv2915c	hypothetical protein	0.2	-0.6	0.9	1	1	2	0.179853173	2.43E-05	8.99E-15
Rv2854	hypothetical protein	-0.2	-0.1	0.9	1	1	2	0.16086591	0.366308608	2.44E-15
Rv0267	narU	0.5	-0.5	0.9	1	1	2	0.000188555	5.05E-05	1.72E-17
Rv0291	mycP3	0.0	-0.1	0.9	1	1	2	0.799249042	0.640976047	1.17E-13
Rv0412c	membrane protein	-1.0	-0.6	0.9	2	1	2	3.73E-21	4.49E-07	1.69E-18
Rv3027c	GCN5-like N-acetyltransferase	0.7	0.2	0.9	2	1	2	1.81E-06	0.227439129	2.26E-13
Rv2578c	hypothetical protein	-1.0	-0.3	0.9	2	1	2	8.41E-10	0.079899124	1.21E-12
Rv1186c	hypothetical protein	-0.1	-0.6	0.9	1	2	2	0.50533096	9.73E-07	1.49E-16
Rv2820c	CRISPR type III-associated RAMP prot	1.4	-0.4	0.9	3	1	2	1.23E-23	0.001566829	5.49E-15

Rv1878	glnA3	0.2	-0.2	0.9	1	1	2	0.182715432	0.133896502	3.47E-16
Rv1671	membrane protein	1.1	-1.7	0.9	2	3	2	3.26E-10	3.59E-27	3.98E-09
Rv1469	ctpD	0.1	0.2	0.9	1	1	2	0.326548774	0.096437689	1.82E-14
Rv0567	methyltransferase/methylase	0.0	0.7	0.9	1	2	2	0.80638814	1.48E-08	1.76E-16
Rv0118c	oxcA	2.2	-0.4	0.9	5	1	2	8.69E-67	0.000965101	1.35E-14
Rv0008c	cell wall synthesis protein CwsA	-1.4	0.0	0.9	3	1	2	9.06E-13	0.871503765	2.21E-10
Rv2717c	hypothetical protein	-0.2	0.6	0.9	1	2	2	0.250163509	3.55E-05	5.94E-16
Rv2066	cobIJ	0.1	0.2	0.9	1	1	2	0.416438615	0.20627942	1.66E-12
Rv2267c	hypothetical protein	0.3	0.2	0.9	1	1	2	0.073365242	0.250460235	3.53E-12
Rv0577	TB27.3	-0.1	-0.8	0.9	1	2	2	0.726834534	2.90E-09	3.04E-14
Rv1471	trxB1	0.5	0.0	0.9	1	1	2	0.016030972	0.762811758	5.19E-11
Rv0101	nrp	0.6	-1.0	0.9	1	2	2	1.67E-07	4.77E-25	9.02E-19
Rv0062	celA1	0.8	-0.1	0.9	2	1	2	7.05E-06	0.537625433	3.20E-12
Rv3097c	lipY	-0.2	0.5	0.9	1	1	2	0.261665199	0.000452433	5.43E-16
Rv2766c	short-chain type dehydrogenase/redu	-0.2	0.1	0.9	1	1	2	0.338815011	0.465943672	1.85E-12
Rv3548c	short-chain type dehydrogenase/redu	0.9	-0.6	0.9	2	1	2	2.50E-10	3.28E-06	3.49E-14
Rv2801A	mazE9	-0.5	-0.4	0.9	1	1	2	0.08087428	0.108560779	1.20E-06
Rv2002	fabG3	0.8	-0.8	0.9	2	2	2	1.84E-08	4.71E-09	3.41E-13
Rv0235c	transmembrane protein	0.5	0.2	0.9	1	1	2	0.000446439	0.095348145	2.44E-15
Rv1833c	haloalkane dehalogenase	0.7	-0.8	0.9	2	2	2	1.32E-08	8.72E-13	5.17E-15
Rv0092	ctpA	0.3	-0.3	0.9	1	1	2	0.034377688	0.029616552	8.86E-17
Rv3077	hydrolase	-0.4	0.2	0.9	1	1	2	0.002504279	0.335555406	1.55E-15
Rv2591	PE PGRS44	0.7	-1.0	0.9	2	2	2	5.45E-08	3.54E-13	7.36E-15
Rv1669	hypothetical protein	-0.7	-0.9	0.9	2	2	2	0.001591939	6.07E-06	2.52E-07
Rv3341	metA	-0.6	-0.5	0.9	1	1	2	0.000522322	2.35E-05	6.41E-15
Rv2608	PPE42	0.4	0.0	0.9	1	1	2	0.004682529	0.899045237	3.22E-15
Rv0535	5'-methylthioadenosine phosphorylas	-0.7	0.4	0.9	2	1	2	2.96E-05	0.011272289	7.44E-12
Rv3549c	short-chain type dehydrogenase/redu	0.8	-1.1	0.9	2	2	2	1.02E-09	6.40E-19	3.65E-14
Rv0344c	lpqJ	-0.3	-0.5	0.9	1	1	2	0.055664127	0.001203113	6.67E-13
Rv2287	yjcE	0.1	-0.2	0.9	1	1	2	0.320839385	0.141439513	1.99E-14
Rv0428c	GCN5-like N-acetyltransferase	-0.3	0.8	0.9	1	2	2	0.031964162	2.38E-05	4.60E-10
Rv0184	hypothetical protein	-0.2	1.2	0.9	1	2	2	0.187972808	1.27E-24	3.50E-16
Rv1989c	hypothetical protein	-0.7	-0.9	0.9	2	2	2	1.24E-06	1.44E-12	3.60E-11
Rv2050	RNA polymerase-binding protein Rbp/	0.6	-0.7	0.9	1	2	2	2.03E-05	2.67E-08	1.54E-11
Rv2796c	lppV	-0.4	0.5	0.9	1	1	2	0.029134167	0.00611207	1.13E-08
Rv2057c	rpmG1	-1.1	0.4	0.9	2	1	2	2.09E-05	0.174723659	3.48E-07
Rv2599	membrane protein	-0.6	0.4	0.9	1	1	2	0.003401264	0.028883416	2.36E-10
Rv3579c	23S rRNA (guanosine(2251)-2'-O)-me	-0.2	0.7	0.9	1	2	2	0.09025348	3.11E-08	2.03E-13
Rv1958c	hypothetical protein	0.8	-1.3	0.9	2	2	2	3.00E-09	5.83E-25	1.59E-13
Rv1495	mazF4	0.0	-0.5	0.9	1	1	2	0.951268112	0.00303984	4.08E-09
Rv0084	hycD	-1.0	0.1	0.9	2	1	2	3.71E-09	0.52412078	9.12E-12
Rv3055	TetR family transcriptional regulator	-0.4	-0.1	0.9	1	1	2	0.009616377	0.61286396	9.24E-12
Rv2886c	resolvase	1.4	-1.1	0.9	3	2	2	6.81E-11	5.91E-18	1.30E-10
Rv0385	monooxygenase	-0.8	-0.3	0.9	2	1	2	1.45E-07	0.031167964	1.44E-12
Rv3845	hypothetical protein	-0.1	0.0	0.9	1	1	2	0.712440999	0.927622201	1.03E-10
Rv1947	hypothetical protein	1.1	-0.7	0.9	2	2	2	2.19E-11	5.00E-06	1.61E-10

Rv1013	pks16	0.1	-0.4	0.9	1	1	2	0.487515508	0.002739192	4.14E-13
Rv1164	narI	0.1	-0.2	0.9	1	1	2	0.24847269	0.07462432	1.10E-10
Rv0762c	hypothetical protein	0.4	-1.4	0.9	1	3	2	0.044222983	4.61E-24	8.96E-12
Rv1206	fadD6	0.2	0.5	0.9	1	1	2	0.076883461	0.000500891	1.17E-14
Rv1414	hypothetical protein	-0.6	0.7	0.9	1	2	2	0.011237375	0.007422594	1.32E-07
Rv3031	1,4-alpha-glucan-branching protein	-0.3	0.5	0.9	1	1	2	0.164425133	0.00134679	4.44E-11
Rv0347	membrane protein	0.1	-0.4	0.9	1	1	2	0.519062473	0.001113783	2.18E-14
Rv2041c	sugar ABC transporter substrate-bindi	0.7	-1.3	0.9	2	2	2	2.54E-07	9.96E-28	2.91E-13
Rv0379	secE2	0.4	0.7	0.9	1	2	2	0.024038744	2.45E-05	1.31E-08
Rv1496	transport system kinase	-0.1	-0.5	0.9	1	1	2	0.577452812	0.00011818	7.08E-13
Rv1204c	hypothetical protein	-0.4	0.0	0.9	1	1	2	0.023184937	0.743222253	1.71E-12
Rv0828c	deaminase	0.6	-0.8	0.9	2	2	2	0.000587141	8.98E-07	4.70E-10
Rv1955	higB	0.7	-2.5	0.9	2	6	2	2.13E-05	4.13E-87	8.23E-08
Rv3399	S-adenosylmethionine-dependent me	-0.5	0.1	0.9	1	1	2	0.000553124	0.405920156	2.91E-12
Rv1754c	hypothetical protein	-0.2	0.2	0.9	1	1	2	0.104756789	0.0857837	4.80E-14
Rv2701c	suhB	0.7	0.0	0.9	2	1	2	3.97E-06	0.926517588	1.39E-12
Rv3062	ligB	-0.1	-0.6	0.9	1	2	2	0.4892726	4.14E-07	7.45E-14
Rv3860	hypothetical protein	-0.3	0.1	0.9	1	1	2	0.050352453	0.405541649	2.44E-14
Rv2828A	hypothetical protein	-0.5	-0.7	0.9	1	2	2	0.027244279	3.23E-05	7.22E-09
Rv0791c	hypothetical protein	-0.3	-0.1	0.8	1	1	2	0.026529089	0.526864971	9.29E-14
Rv1578c	phage protein	0.1	-2.5	0.8	1	5	2	0.425167317	3.24E-58	5.88E-10
Rv3702c	egtC	-0.1	0.0	0.8	1	1	2	0.480799258	0.894033523	2.82E-11
Rv3422c	tRNA threonylcarbamoyladenosine bi	-0.1	0.0	0.8	1	1	2	0.509982422	0.884286081	2.50E-10
Rv3556c	fadA6	0.3	-1.1	0.8	1	2	2	0.048209588	5.84E-20	5.13E-13
Rv1510	hypothetical protein	0.1	0.1	0.8	1	1	2	0.425615645	0.538154865	5.26E-13
Rv3731	ligC	-0.5	0.1	0.8	1	1	2	0.000739067	0.609045017	2.66E-12
Rv2226	hypothetical protein	0.2	-0.4	0.8	1	1	2	0.057856129	0.001248422	2.66E-13
Rv1600	hisC1	0.6	-1.2	0.8	2	2	2	9.56E-08	8.95E-26	4.17E-08
Rv2848c	cobB	-0.6	-0.7	0.8	2	2	2	0.000638774	5.85E-08	2.86E-13
Rv0136	cyp138	-0.7	-0.3	0.8	2	1	2	8.98E-07	0.009988712	8.17E-13
Rv2661c	hypothetical protein	-2.1	-0.9	0.8	4	2	2	1.88E-42	4.95E-14	1.03E-13
Rv3861	hypothetical protein	-1.1	-0.3	0.8	2	1	2	9.13E-05	0.119283047	2.35E-06
Rv2913c	D-amino acid aminohydrolase	-0.5	0.3	0.8	1	1	2	0.000101099	0.011791323	3.22E-14
Rv2033c	hypothetical protein	-1.0	-0.3	0.8	2	1	2	3.14E-06	0.086017115	1.47E-09
Rv0401	transmembrane protein	-0.8	0.3	0.8	2	1	2	0.000105353	0.120685524	5.82E-08
Rv1135c	PPE16	0.1	0.1	0.8	1	1	2	0.505452411	0.239823832	9.56E-14
Rv1787	PPE25	0.0	-0.7	0.8	1	2	2	0.789643948	2.27E-08	4.97E-13
Rv0539	dolichyl-phosphate sugar synthase	-0.9	-1.8	0.8	2	4	2	2.04E-08	3.32E-39	3.86E-09
Rv0916c	PE7	-0.9	-0.6	0.8	2	2	2	0.000632763	0.001470932	2.14E-07
Rv1912c	fadB5	0.8	-0.6	0.8	2	1	2	9.46E-09	9.38E-06	1.59E-11
Rv3644c	DNA polymerase	0.3	-0.2	0.8	1	1	2	0.013421141	0.086453977	1.59E-12
Rv2975c	hypothetical protein	0.1	-0.2	0.8	1	1	2	0.606492459	0.234008337	1.60E-07
Rv0332	hypothetical protein	-0.6	0.6	0.8	1	2	2	0.000365137	4.75E-05	3.84E-12
Rv3221A	rshA	0.1	-0.3	0.8	1	1	2	0.274556916	0.021433835	3.45E-13
Rv1516c	sugar transferase	-0.3	0.2	0.8	1	1	2	0.092340837	0.395733005	2.30E-10
Rv2208	cobS	0.0	-0.6	0.8	1	2	2	0.965167114	2.52E-05	3.59E-11

Rv2673	aftC	0.4	0.5	0.8	1	1	2	0.006305502	0.000140387	6.51E-13
Rv2001	hypothetical protein	0.5	-0.6	0.8	1	2	2	0.00215646	2.90E-05	1.14E-10
Rv3040c	hypothetical protein	-0.5	0.7	0.8	1	2	2	0.003199629	0.000107827	1.86E-11
Rv2231c	cobC	0.4	0.3	0.8	1	1	2	0.002321414	0.110034137	5.17E-12
Rv3123	hypothetical protein	-0.3	-0.2	0.8	1	1	2	0.06052131	0.161229805	5.73E-11
Rv1960c	parD1	0.0	-1.0	0.8	1	2	2	0.859966322	1.00E-08	8.06E-08
Rv3887c	eccD2	0.7	0.2	0.8	2	1	2	5.95E-07	0.075571963	2.42E-11
Rv0687	NAD-dependent oxidoreductase	-0.4	0.2	0.8	1	1	2	0.002441309	0.203753846	6.99E-15
Rv3406	dioxygenase	-0.9	-1.2	0.8	2	2	2	1.70E-09	2.37E-22	3.63E-12
Rv3286c	sigF	0.4	-1.3	0.8	1	3	2	0.005768938	1.60E-30	1.09E-10
Rv2934	ppsD	-1.1	1.3	0.8	2	2	2	3.36E-22	2.53E-38	1.01E-13
Rv0929	pstC2	0.8	-0.9	0.8	2	2	2	2.09E-07	7.33E-13	3.82E-12
Rv2023c	hypothetical protein	0.4	0.2	0.8	1	1	2	0.019443172	0.277460774	2.18E-08
Rv0115a	hypothetical protein CDS	1.2	-1.3	0.8	2	3	2	5.16E-12	2.20E-14	7.04E-07
Rv0735	sigL	1.1	0.2	0.8	2	1	2	1.26E-12	0.380634938	3.06E-09
Rv3594	hypothetical protein	-0.8	0.3	0.8	2	1	2	1.86E-06	0.069557067	3.66E-11
Rv0183	lysophospholipase	-0.8	1.6	0.8	2	3	2	2.71E-08	2.15E-30	1.05E-11
Rv2458	mmuM	-0.5	0.0	0.8	1	1	2	0.000940844	0.965535259	1.18E-10
Rv1376	hypothetical protein	0.5	0.0	0.8	1	1	2	0.000100828	0.885704169	4.05E-12
Rv1011	ispE	-1.0	0.2	0.8	2	1	2	3.72E-06	0.239814766	3.63E-10
Rv1146	transmembrane transport protein	0.0	-0.3	0.8	1	1	2	0.731835354	0.023766953	2.94E-12
Rv0517	acyltransferase	0.3	0.3	0.8	1	1	2	0.027268367	0.021926733	2.88E-13
Rv2572c	aspS	0.2	0.1	0.8	1	1	2	0.051006202	0.607469522	5.00E-12
Rv2664	hypothetical protein	-0.1	-1.4	0.8	1	3	2	0.758650378	1.58E-21	1.98E-09
Rv3768	hypothetical protein	-0.5	0.1	0.8	1	1	2	0.01066654	0.487288702	2.50E-08
Rv2117	hypothetical protein	0.3	0.1	0.8	1	1	2	0.146038825	0.525406939	8.03E-08
Rv0143c	transmembrane protein	-0.5	0.5	0.8	1	1	2	2.88E-05	3.78E-06	1.55E-12
Rv3313c	add	-0.5	0.5	0.8	1	1	2	0.000703321	0.003563917	3.02E-10
Rv0212c	nadR	1.1	-1.1	0.8	2	2	2	4.15E-13	2.53E-21	6.78E-12
Rv3181c	antitoxin VapB45	-1.6	-1.3	0.8	3	2	2	2.28E-14	4.44E-18	2.49E-08
Rv1685c	hypothetical protein	0.3	-0.9	0.8	1	2	2	0.072462548	9.55E-10	2.58E-09
Rv3066	DeoR family transcriptional regulator	-0.3	0.8	0.8	1	2	2	0.022522516	1.34E-07	1.31E-11
Rv3899c	hypothetical protein	-0.4	-0.3	0.8	1	1	2	0.006849104	0.058016538	1.67E-10
Rv3617	ephA	-0.9	-0.2	0.8	2	1	2	1.46E-07	0.079306018	2.86E-11
Rv0965c	hypothetical protein	0.1	-0.1	0.8	1	1	2	0.789415305	0.453063348	5.62E-08
Rv2373c	dnaJ2	0.5	-0.9	0.8	1	2	2	2.95E-05	1.08E-14	1.03E-12
Rv3168	aminoglycoside phosphotransferase	0.1	-0.2	0.8	1	1	2	0.481114535	0.081075746	1.82E-12
Rv2600	integral membrane protein	0.1	0.0	0.8	1	1	2	0.78497278	0.940093758	3.96E-07
Rv1562c	treZ	-0.1	0.3	0.8	1	1	2	0.529284365	0.029890639	1.63E-12
Rv3608c	folP1	-0.1	0.0	0.8	1	1	2	0.332528039	0.992057777	4.98E-10
Rv0840c	pip	-0.3	-0.5	0.8	1	1	2	0.037467604	0.000413975	7.49E-08
Rv3192	hypothetical protein	-0.1	-0.4	0.8	1	1	2	0.421573122	0.005703499	9.86E-10
Rv3228	hypothetical protein	-0.9	0.1	0.8	2	1	2	8.29E-09	0.678536173	2.60E-10
Rv0991c	hypothetical protein	-0.3	-0.1	0.8	1	1	2	0.011057084	0.388686913	2.08E-12
Rv3793	embC	0.0	0.8	0.8	1	2	2	0.802638745	5.58E-08	2.01E-12
Rv0958	magnesium chelatase	-0.4	-0.7	0.8	1	2	2	0.006923094	2.06E-08	5.58E-10

Rv2035	hypothetical protein	-1.5	-1.2	0.8	3	2	2	3.46E-18	1.45E-18	1.49E-09
Rv1230c	membrane protein	-0.2	0.0	0.8	1	1	2	0.18929667	0.921687833	3.62E-09
Rv0319	pcp	0.3	0.4	0.8	1	1	2	0.099770771	0.0242868	5.23E-08
Rv1008	tatD	0.1	0.1	0.8	1	1	2	0.663027685	0.598184873	2.11E-09
Rv1762c	hypothetical protein	0.0	0.4	0.8	1	1	2	0.86785423	0.009409224	2.01E-10
Rv2850c	magnesium chelatase	-0.3	-1.0	0.8	1	2	2	0.058862201	2.31E-21	5.43E-13
Rv0841	transmembrane protein	-0.9	-0.9	0.8	2	2	2	2.10E-06	1.03E-07	7.60E-08
Rv2555c	alaS	0.4	-0.9	0.8	1	2	2	0.000364841	7.67E-19	1.24E-12
Rv0089	methyltransferase	0.1	-0.7	0.8	1	2	2	0.41066259	8.56E-07	1.13E-09
Rv3086	adhD	0.1	-1.6	0.8	1	3	2	0.660642817	4.01E-42	1.56E-10
Rv1239c	corA	0.5	-0.4	0.8	1	1	2	0.000169319	0.005270886	1.03E-09
Rv2677c	hemY	-0.4	-0.4	0.8	1	1	2	0.011333609	0.000767406	2.56E-09
Rv2887	HTH-type transcriptional regulator	-0.1	-1.4	0.8	1	3	2	0.55220975	4.93E-24	4.46E-10
Rv2967c	pca	0.6	0.0	0.8	1	1	2	2.04E-06	0.826714118	5.91E-13
Rv1954c	hypothetical protein	0.6	-3.3	0.8	1	10	2	0.000802035	7.02E-259	1.07E-12
Rv2658c	prophage protein	0.4	-1.8	0.8	1	4	2	0.052322714	4.45E-31	5.74E-07
Rv3630	integral membrane protein	-0.2	0.3	0.8	1	1	2	0.142610807	0.032559448	6.16E-10
Rv2896c	hypothetical protein	-0.6	-1.0	0.8	2	2	2	0.001011242	2.30E-12	8.76E-09
Rv1526c	glycosyltransferase	-0.6	-0.6	0.8	1	1	2	8.51E-05	8.56E-06	4.26E-10
Rv3913	trxB2	0.3	-0.3	0.8	1	1	2	0.01176671	0.034080982	2.38E-11
Rv1666c	cyp139	-0.5	0.3	0.8	1	1	2	0.00198941	0.095595803	4.60E-09
Rv2983	2-phospho-L-lactate guanylyltransfera	-2.0	0.5	0.8	4	1	2	8.59E-23	0.00364503	1.28E-08
Rv2046	lppI	-1.0	0.7	0.8	2	2	2	1.36E-06	0.000395064	2.23E-08
Rv1115	hypothetical protein	0.0	0.4	0.8	1	1	2	0.841856394	0.016804963	2.92E-09
Rv2740	ephG	0.2	0.5	0.8	1	1	2	0.121236522	0.000611593	1.42E-07
Rv1032c	trcS	0.2	0.3	0.8	1	1	2	0.162065871	0.025653977	4.77E-11
Rv1853	ureD	0.9	-1.1	0.8	2	2	2	4.42E-10	2.11E-12	5.13E-08
Rv3047c	hypothetical protein	0.3	0.1	0.7	1	1	2	0.021140749	0.439192357	9.61E-09
Rv2161c	hypothetical protein	0.0	-0.5	0.7	1	1	2	0.71465777	7.19E-05	2.62E-09
Rv3850	hypothetical protein	-0.3	0.0	0.7	1	1	2	0.132405341	0.983975487	5.40E-09
Rv0039c	transmembrane protein	0.3	-1.0	0.7	1	2	2	0.085839395	6.20E-08	4.28E-07
Rv1602	hisH	1.0	-1.0	0.7	2	2	2	4.09E-16	9.92E-17	1.44E-10
Rv2726c	dapF	-0.1	-0.2	0.7	1	1	2	0.364185709	0.044041793	1.42E-09
Rv0366c	hypothetical protein	-0.7	0.1	0.7	2	1	2	0.000411353	0.615090351	3.11E-06
Rv1031	kdpC	0.5	-0.4	0.7	1	1	2	0.000541859	0.009698504	4.46E-08
Rv1603	hisA	0.1	-0.8	0.7	1	2	2	0.532837055	3.54E-13	8.61E-11
Rv1515c	hypothetical protein	0.9	0.5	0.7	2	1	2	1.79E-07	0.002489406	5.80E-10
Rv3730c	hypothetical protein	-0.1	0.1	0.7	1	1	2	0.307499887	0.605374144	2.52E-10
Rv1426c	lipO	0.5	0.2	0.7	1	1	2	2.18E-05	0.090385902	5.30E-10
Rv0817c	hypothetical protein	0.1	0.3	0.7	1	1	2	0.599077186	0.096658304	2.75E-09
Rv2631	RNA-splicing ligase RtcB	0.6	-0.8	0.7	2	2	2	1.06E-07	4.37E-14	1.95E-10
Rv0063	oxidoreductase	0.0	0.2	0.7	1	1	2	0.841388802	0.172985071	1.20E-09
Rv1313c	insertion sequence element IS1557 tr	-0.1	0.2	0.7	1	1	2	0.395426465	0.033262205	1.41E-11
Rv2628	hypothetical protein	0.5	-0.8	0.7	1	2	2	0.000744682	2.23E-08	2.07E-08
Rv0843	dehydrogenase	-0.6	-0.2	0.7	2	1	2	0.00052514	0.122202974	4.21E-08
Rv0456A	mazF1	-0.2	0.0	0.7	1	1	2	0.333929004	0.978610826	3.85E-05

Rv0193c	hypothetical protein	-0.8	0.3	0.7	2	1	2	7.10E-10	0.007257556	5.64E-11
Rv1849	ureB	0.0	1.3	0.7	1	2	2	0.903280241	9.52E-11	4.70E-08
Rv3163c	hypothetical protein	-0.1	0.1	0.7	1	1	2	0.705148713	0.670696781	4.02E-10
Rv3242c	hypothetical protein	1.0	-1.5	0.7	2	3	2	2.27E-11	7.24E-32	6.06E-09
Rv2234	ptpA	1.0	-0.8	0.7	2	2	2	9.10E-12	1.57E-08	9.62E-08
Rv2612c	pgsA1	0.8	-0.3	0.7	2	1	2	1.16E-09	0.017594072	8.07E-09
Rv2723	integral membrane protein	0.0	0.5	0.7	1	1	2	0.951243835	0.000112927	1.80E-08
Rv1427c	fadD12	-0.1	-0.1	0.7	1	1	2	0.427469361	0.656329829	5.34E-10
Rv0612	hypothetical protein	0.3	-0.8	0.7	1	2	2	0.031707068	9.17E-09	3.54E-08
Rv3179	hypothetical protein	0.0	-0.6	0.7	1	2	2	0.932296863	9.46E-06	7.40E-09
Rv0087	hycE	-0.9	0.8	0.7	2	2	2	1.32E-11	2.10E-08	5.95E-10
Rv1850	ureC	-0.2	0.8	0.7	1	2	2	0.170128191	5.72E-10	4.59E-10
Rv2632c	hypothetical protein	0.5	-0.9	0.7	1	2	2	0.001318403	6.08E-14	3.56E-09
Rv1139c	hypothetical protein	0.4	-0.4	0.7	1	1	2	0.032708642	0.021154929	1.17E-06
Rv1160	mutT2	0.0	0.6	0.7	1	2	2	0.863617071	0.009107044	4.36E-06
Rv3703c	etgB	-0.1	-0.2	0.7	1	1	2	0.369262867	0.097093593	5.56E-09
Rv3897c	hypothetical protein	0.3	0.0	0.7	1	1	2	0.057914522	0.932406835	6.32E-07
Rv2551c	hypothetical protein	0.2	-1.4	0.7	1	3	2	0.16180425	5.33E-31	5.84E-08
Rv3494c	mce4F	0.1	0.4	0.7	1	1	2	0.318951308	0.002400477	5.18E-10
Rv1977	hypothetical protein	0.5	-1.1	0.7	1	2	2	0.000459992	1.06E-15	6.91E-09
Rv1161	narG	0.4	-0.8	0.7	1	2	2	0.001206757	5.89E-15	3.31E-10
Rv0980c	PE PGRS18	-1.0	0.3	0.7	2	1	2	8.02E-08	0.052997133	7.34E-09
Rv1029	kdpA	-0.4	0.3	0.7	1	1	2	0.006456999	0.014898446	2.20E-09
Rv0465c	HTH-type transcriptional regulator	-0.3	-1.8	0.7	1	4	2	0.008972885	3.05E-59	4.92E-10
Rv2756c	hsdM	0.0	0.7	0.7	1	2	2	0.80505269	3.93E-08	1.24E-09
Rv3798	insertion sequence element IS1557 tr	-0.1	0.2	0.7	1	1	2	0.272456808	0.049902055	1.26E-10
Rv2424c	transposase	0.4	0.1	0.7	1	1	2	0.00181963	0.600702694	2.96E-10
Rv0035	fadD34	0.0	0.4	0.7	1	1	2	0.896091942	0.001047989	9.17E-10
Rv1466	hypothetical protein	0.5	-2.3	0.7	1	5	2	0.004295064	6.81E-85	2.89E-08
Rv3555c	hypothetical protein	0.7	-0.8	0.7	2	2	2	2.98E-05	4.23E-08	5.80E-08
Rv1946c	lppG	1.2	-1.8	0.7	2	3	2	3.77E-20	2.79E-53	9.72E-09
Rv0457c	peptidase	0.4	-0.6	0.7	1	2	2	0.002018757	6.23E-08	4.10E-10
Rv0878c	PPE13	0.7	-0.2	0.7	2	1	2	6.17E-07	0.025027141	1.47E-10
Rv3343c	PPE54	-0.5	0.4	0.7	1	1	2	0.00891994	0.00039588	5.48E-06
Rv2055c	rpsR2	-1.3	0.1	0.7	2	1	2	1.91E-06	0.751196476	6.07E-05
Rv0413	mutT3	-0.2	-0.7	0.7	1	2	2	0.306650836	3.81E-07	1.36E-07
Rv1658	argG	0.2	-0.3	0.7	1	1	2	0.073435665	0.008767384	6.81E-08
Rv2397c	cysA1	1.0	-0.5	0.7	2	1	2	5.02E-13	0.000239149	3.63E-09
Rv1256c	cyp130	-0.7	-0.3	0.7	2	1	2	0.000154745	0.015292794	4.79E-09
Rv0785	KsdD-like steroid dehydrogenase	0.0	-0.2	0.7	1	1	2	0.986518361	0.135070379	2.49E-09
Rv0739	hypothetical protein	-0.4	0.4	0.7	1	1	2	0.016849852	0.00652254	2.59E-08
Rv2294	cystathionine beta-lyase	0.1	0.7	0.7	1	2	2	0.503278602	1.04E-05	1.43E-08
Rv0262c	aac	0.1	-1.2	0.7	1	2	2	0.719779978	5.86E-18	2.78E-06
Rv1114	vapC32	0.6	0.0	0.7	2	1	2	0.000287574	0.980583137	1.46E-07
Rv3621c	PPE65	0.1	0.1	0.7	1	1	2	0.500084869	0.348979545	9.49E-08
Rv1401	membrane protein	-0.4	-0.3	0.7	1	1	2	0.017277516	0.096538403	8.53E-08

Rv3039c	echA17	-0.2	0.3	0.7	1	1	2	0.230524587	0.043187835	2.83E-07
Rv2323c	hypothetical protein	-0.1	-2.0	0.7	1	4	2	0.343128158	2.71E-60	7.94E-08
Rv3417c	groEL1	0.4	-0.8	0.7	1	2	2	0.000124556	6.53E-15	1.15E-09
Rv1831	hypothetical protein	-0.1	-1.6	0.7	1	3	2	0.276574638	9.47E-67	1.10E-08
Rv1895	zinc-binding alcohol dehydrogenase	0.4	-0.4	0.7	1	1	2	0.006812183	0.00603098	2.15E-08
Rv3069	fluoride ion transporter CrcB	-0.3	-0.5	0.7	1	1	2	0.089886244	0.004288794	7.75E-05
Rv1014c	pth	-0.5	0.4	0.7	1	1	2	0.006999029	0.064030936	1.04E-05
Rv2776c	oxidoreductase	0.7	-0.9	0.7	2	2	2	4.48E-06	7.77E-13	4.40E-08
Rv0258c	hypothetical protein	-0.2	-1.6	0.7	1	3	2	0.346189437	7.25E-24	5.80E-07
Rv3218	hypothetical protein	-0.2	0.7	0.7	1	2	2	0.176703185	1.06E-05	8.04E-09
Rv0594	mce2F	-0.3	0.2	0.7	1	1	2	0.022990916	0.080345393	2.09E-09
Rv1026	hypothetical protein	-0.1	-0.3	0.7	1	1	2	0.624209732	0.029308218	9.23E-08
Rv2855	mtr	1.4	-0.8	0.7	3	2	2	8.91E-28	9.60E-10	3.40E-08
Rv0438c	moeA2	0.1	-0.1	0.7	1	1	2	0.334006622	0.491265628	1.17E-07
Rv2492	hypothetical protein	-0.3	-1.3	0.7	1	2	2	0.178268795	9.04E-15	8.11E-05
Rv3363c	hypothetical protein	0.1	0.5	0.6	1	1	2	0.647454302	0.036443222	3.73E-05
Rv3410c	guaB3	0.5	-0.3	0.6	1	1	2	0.000151452	0.006628675	2.18E-08
Rv0070c	glyA2	0.2	0.9	0.6	1	2	2	0.150655456	1.46E-08	1.42E-08
Rv3391	acrA1	-0.5	0.0	0.6	1	1	2	0.000690593	0.74519081	2.59E-08
Rv3322c	methyltransferase	-0.1	0.2	0.6	1	1	2	0.660745751	0.43223229	1.00E-05
Rv1453	transcriptional activator protein	0.4	0.2	0.6	1	1	2	0.005960916	0.209376848	8.17E-09
Rv2118c	tRNA (adenine(58)-N(1))-methyltrans	0.0	0.3	0.6	1	1	2	0.794101371	0.041301814	9.48E-07
Rv3440c	hypothetical protein	0.0	-0.3	0.6	1	1	2	0.85858599	0.061083025	1.02E-05
Rv2486	echA14	0.0	-0.4	0.6	1	1	2	0.878996027	0.003163312	4.45E-07
Rv3393	iunH	0.1	0.2	0.6	1	1	2	0.604396513	0.248861288	9.17E-07
Rv2207	cobT	-0.4	-0.6	0.6	1	1	2	0.022397999	0.000447039	9.93E-07
Rv1748	hypothetical protein	0.2	-0.9	0.6	1	2	2	0.247963272	1.12E-10	5.50E-07
Rv2229c	hypothetical protein	1.2	0.1	0.6	2	1	2	1.45E-18	0.595729192	2.22E-07
Rv2017	transcriptional regulator	-0.2	-0.4	0.6	1	1	2	0.156862635	0.002300312	1.02E-07
Rv2015c	hypothetical protein	0.4	-0.7	0.6	1	2	2	0.02023986	1.07E-07	1.17E-06
Rv2859c	glutamine amidotransferase	-0.5	-0.2	0.6	1	1	2	0.000508832	0.091882118	2.85E-08
Rv1986	amino acid transporter	-0.4	-0.9	0.6	1	2	2	0.010175373	2.39E-09	3.67E-06
Rv0111	acyltransferase	-0.1	0.6	0.6	1	2	2	0.249795591	3.61E-05	1.13E-08
Rv3516	echA19	-0.6	0.7	0.6	2	2	2	2.03E-05	3.18E-05	1.89E-07
Rv0188	transmembrane protein	0.1	-2.3	0.6	1	5	2	0.599032568	8.17E-78	9.35E-09
Rv3091	hypothetical protein	-0.8	-0.4	0.6	2	1	2	3.69E-06	0.000282953	1.50E-08
Rv1739c	sulfate ABC transporter permease	2.4	-1.9	0.6	5	4	2	1.52E-90	1.80E-67	2.48E-07
Rv0453	PPE11	-0.4	0.5	0.6	1	1	2	0.000938063	0.000393285	2.50E-08
Rv3012c	gatC	-0.3	0.9	0.6	1	2	2	0.176871623	5.42E-05	3.73E-05
Rv2638	hypothetical protein	-0.3	-1.4	0.6	1	3	2	0.146414894	1.25E-19	3.18E-06
Rv2254c	integral membrane protein	-0.1	-0.6	0.6	1	1	2	0.550641287	0.001411264	2.50E-05
Rv1379	pyrR	-0.2	0.5	0.6	1	1	2	0.249632953	0.002974119	7.06E-07
Rv2192c	trpD	-0.2	-0.2	0.6	1	1	2	0.318124421	0.132976274	3.62E-07
Rv2482c	plsB2	0.5	-1.4	0.6	1	3	2	1.32E-05	4.65E-31	1.25E-07
Rv1236	sugA	0.5	-0.5	0.6	1	1	2	8.91E-05	8.97E-05	2.19E-07
Rv2005c	universal stress protein	2.3	-3.8	0.6	5	14	2	2.45E-64	0	1.62E-07

Rv1819c	bacA	-0.4	1.1	0.6	1	2	2	0.001202185	7.67E-20	9.08E-08
Rv0627	vapC5	-0.5	-0.5	0.6	1	1	2	0.006583394	0.00259992	3.92E-06
Rv0269c	hypothetical protein	-0.7	0.0	0.6	2	1	2	3.73E-06	0.925690289	3.21E-07
Rv2759c	vapC42	0.4	0.3	0.6	1	1	2	0.014448962	0.092408012	5.21E-06
Rv3569c	hsaD	0.4	0.2	0.6	1	1	2	0.005975777	0.219351825	2.93E-07
Rv2177c	transposase	0.2	0.0	0.6	1	1	2	0.0770499	0.764101215	9.87E-08
Rv3032	glycogen synthase	0.0	1.1	0.6	1	2	2	0.93626735	7.28E-09	8.16E-07
Rv1812c	dehydrogenase	0.2	0.3	0.6	1	1	2	0.127749472	0.013577772	3.43E-08
Rv1499	hypothetical protein	-0.1	-0.2	0.6	1	1	2	0.520688989	0.242800445	2.26E-05
Rv0867c	rpfA	0.2	0.1	0.6	1	1	2	0.091896955	0.85159195	8.05E-07
Rv1128c	hypothetical protein	0.9	-1.6	0.6	2	3	2	4.06E-12	7.58E-49	3.51E-07
Rv0121c	hypothetical protein	-0.9	-1.1	0.6	2	2	2	1.29E-05	5.04E-11	4.86E-05
Rv0731c	S-adenosylmethionine-dependent me	-0.8	0.3	0.6	2	1	2	0.001160114	0.081931935	1.20E-06
Rv0987	adhesion component ABC transporter	0.0	0.7	0.6	1	2	2	0.959638696	5.46E-10	1.03E-06
Rv3509c	ilvX	0.2	0.1	0.6	1	1	2	0.118188594	0.505228407	1.78E-07
Rv1765c	hypothetical protein	0.3	-0.6	0.6	1	2	2	0.048192967	5.47E-07	1.81E-06
Rv2821c	CRISPR type III-associated RAMP prot	1.7	-0.9	0.6	3	2	2	1.19E-36	1.20E-10	1.48E-05
Rv2076c	hypothetical protein	0.6	-1.4	0.6	2	3	2	0.000738785	3.44E-21	0.000187569
Rv0117	oxyS	0.7	0.1	0.6	2	1	2	9.11E-07	0.546322265	6.84E-07
Rv2070c	cobK	0.4	0.1	0.6	1	1	2	0.047636302	0.495527935	1.16E-05
Rv0064A	vapB1	0.3	-2.5	0.6	1	6	2	0.127351922	2.64E-72	0.000114272
Rv0232	transcriptional regulator	0.5	-1.7	0.6	1	3	2	0.000184192	2.71E-35	2.34E-07
Rv0541c	integral membrane protein	0.3	0.1	0.6	1	1	2	0.059145407	0.549949144	5.78E-07
Rv1538c	ansA	-0.4	0.1	0.6	1	1	2	0.006504727	0.697276038	1.32E-06
Rv3236c	integral membrane transport protein	-0.4	-0.3	0.6	1	1	2	0.003936881	0.025923982	1.18E-07
Rv3605c	hypothetical protein	-0.4	-0.2	0.6	1	1	2	0.019695362	0.153500494	2.25E-06
Rv2911	dacB2	-0.7	0.9	0.6	2	2	2	1.03E-05	1.03E-06	5.47E-06
Rv1692	phosphatase	0.4	0.1	0.6	1	1	2	0.007098337	0.426896598	7.49E-06
Rv0071	maturase	-0.4	-0.1	0.6	1	1	2	0.004351802	0.572808043	3.00E-06
Rv2083	hypothetical protein	0.0	0.4	0.6	1	1	2	0.765510818	0.077866141	5.74E-06
Rv3000	transmembrane protein	0.7	0.4	0.6	2	1	2	1.00E-06	0.012958605	4.26E-06
Rv2180c	integral membrane protein	-0.1	-0.2	0.6	1	1	1	0.501611642	0.216331502	1.84E-05
Rv2277c	glycerolphosphodiesterase	-0.2	0.4	0.6	1	1	1	0.149948965	0.000386054	2.46E-07
Rv2047c	hypothetical protein	-0.1	0.5	0.6	1	1	1	0.242360469	0.000304698	7.23E-08
Rv0339c	transcriptional regulator	-0.6	0.4	0.6	2	1	1	5.96E-06	0.000760544	1.12E-07
Rv3497c	mce4C	0.5	0.3	0.6	1	1	1	5.80E-05	0.017890029	1.98E-06
Rv3589	mutY	-1.6	0.6	0.6	3	2	1	4.22E-17	0.000175442	1.62E-05
Rv1392	metK	0.5	-0.3	0.6	1	1	1	7.04E-05	0.012572335	1.04E-06
Rv3122	hypothetical protein	-0.6	0.8	0.6	1	2	1	1.78E-05	1.39E-08	0.00015558
Rv1957	SecB-like chaperone	1.3	-1.6	0.6	2	3	1	2.04E-24	2.46E-41	3.34E-05
Rv1355c	moeY	-0.7	-0.7	0.6	2	2	1	2.90E-08	8.59E-09	1.35E-07
Rv2048c	pks12	0.9	0.6	0.6	2	2	1	1.47E-05	2.04E-06	1.83E-05
Rv2721c	hypothetical protein	0.1	1.0	0.6	1	2	1	0.674812608	3.05E-22	4.33E-07
Rv3279c	birA	0.5	0.1	0.6	1	1	1	3.22E-05	0.594589919	6.89E-06
Rv1691	hypothetical protein	-0.6	0.2	0.6	2	1	1	0.000781759	0.354004033	3.88E-05
Rv2141c	hypothetical protein	-0.1	-0.1	0.6	1	1	1	0.441887004	0.632114528	1.03E-06

Rv0449c	hypothetical protein	-0.4	0.1	0.6	1	1	1	0.001747932	0.419401925	5.06E-07
Rv2434c	transmembrane protein	-0.4	0.5	0.6	1	1	1	0.003511336	0.000741583	2.56E-06
Rv2549c	vapC20	-0.1	0.3	0.6	1	1	1	0.778514312	0.12844287	8.67E-05
Rv1058	fadD14	0.7	0.0	0.6	2	1	1	6.66E-08	0.948063475	3.33E-06
Rv3894c	eccC2	0.4	0.0	0.6	1	1	1	0.000886418	0.642741931	7.24E-08
Rv3700c	egtE	0.5	-0.4	0.6	1	1	1	0.000322838	0.002371588	5.50E-06
Rv0097	oxidoreductase	-0.3	0.7	0.6	1	2	1	0.035025829	1.49E-06	1.86E-05
Rv1820	ilvG	0.1	-0.7	0.6	1	2	1	0.355705182	7.15E-10	6.14E-07
Rv2235	transmembrane protein	0.9	-0.8	0.6	2	2	1	8.67E-12	1.36E-08	3.16E-06
Rv1520	sugar transferase	-0.1	-0.6	0.6	1	2	1	0.334646493	2.14E-06	5.63E-06
Rv3567c	hsaB	-0.1	-0.7	0.6	1	2	1	0.696896608	3.38E-06	3.32E-05
Rv3707c	hypothetical protein	0.3	-0.2	0.6	1	1	1	0.058594856	0.054099942	1.90E-06
Rv3386	transposase	-0.7	-0.1	0.6	2	1	1	5.53E-05	0.739766124	1.89E-05
Rv2553c	membrane protein	0.2	-0.9	0.6	1	2	1	0.180675591	1.99E-15	6.72E-06
Rv3037c	S-adenosylmethionine-dependent me	-0.5	-0.2	0.6	1	1	1	0.016292454	0.147983479	1.52E-05
Rv0879c	transmembrane protein	-1.0	0.0	0.6	2	1	1	0.000116028	0.966394056	0.000844004
Rv3348	transposase	0.3	-1.0	0.6	1	2	1	0.069037897	5.64E-18	9.71E-06
Rv2006	otsB1	0.8	-1.9	0.5	2	4	1	1.10E-12	3.98E-68	3.09E-07
Rv2201	asnB	0.0	0.6	0.5	1	2	1	0.689122447	1.56E-08	1.38E-06
Rv0668	rpoC	0.6	0.9	0.5	2	2	1	2.11E-08	1.78E-22	8.48E-06
Rv0527	ccdA	0.4	0.1	0.5	1	1	1	0.00842358	0.394069566	4.51E-05
Rv1353c	HTH-type transcriptional regulator	0.7	-1.2	0.5	2	2	1	4.38E-06	6.35E-18	3.16E-05
Rv0180c	transmembrane protein	0.3	0.0	0.5	1	1	1	0.014831012	0.902546112	3.08E-06
Rv1848	ureA	-0.3	0.9	0.5	1	2	1	0.152423306	3.75E-06	0.00010494
Rv3194c	hypothetical protein	0.0	0.2	0.5	1	1	1	0.775268243	0.100789499	7.94E-06
Rv3844	transposase	0.3	-1.0	0.5	1	2	1	0.058126315	4.93E-18	1.63E-05
Rv2715	hydrolase	-0.6	1.0	0.5	1	2	1	4.54E-07	7.31E-19	1.05E-06
Rv2059	hypothetical protein	0.0	0.7	0.5	1	2	1	0.767049751	3.49E-08	3.60E-06
Rv2827c	hypothetical protein	-0.7	0.4	0.5	2	1	1	1.41E-07	0.000844868	1.52E-06
Rv1187	rocA	-1.1	-0.1	0.5	2	1	1	9.90E-09	0.449638696	4.41E-05
Rv3144c	PPE52	0.3	0.4	0.5	1	1	1	0.024041528	0.001577364	8.25E-05
Rv0182c	sigG	0.3	-0.1	0.5	1	1	1	0.012015597	0.450872345	1.16E-05
Rv3178a	hypothetical protein CDS	-0.9	-0.6	0.5	2	1	1	4.00E-06	0.002476247	0.000541505
Rv3340	metC	-0.5	-0.3	0.5	1	1	1	2.65E-05	0.006481494	8.38E-07
Rv1557	mmpL6	-0.7	-0.3	0.5	2	1	1	2.11E-07	0.004639338	2.05E-06
Rv3035	hypothetical protein	0.2	-0.5	0.5	1	1	1	0.125102312	0.000229724	2.32E-05
Rv0021c	hypothetical protein	0.2	-0.1	0.5	1	1	1	0.245186896	0.596443915	1.25E-05
Rv3356c	folD	0.3	-0.6	0.5	1	1	1	0.058528209	2.33E-05	3.83E-05
Rv3016	lpqA	0.4	-1.2	0.5	1	2	1	0.00524292	1.28E-20	1.88E-05
Rv0104	hypothetical protein	0.0	-0.6	0.5	1	2	1	0.951351118	5.23E-07	4.03E-05
Rv2538c	aroB	-0.1	0.2	0.5	1	1	1	0.409673778	0.161957503	5.82E-06
Rv2849c	cobO	0.4	-0.9	0.5	1	2	1	0.018407757	1.57E-10	0.000409469
Rv3760	membrane protein	-0.1	-0.4	0.5	1	1	1	0.647783315	0.026227376	0.000359632
Rv3745c	hypothetical protein	-0.7	0.3	0.5	2	1	1	0.002113015	0.254962492	0.002280741
Rv3639c	hypothetical protein	-0.2	-0.1	0.5	1	1	1	0.164711658	0.635870626	2.80E-05
Rv0098	fcoT	0.0	0.3	0.5	1	1	1	0.98009458	0.070797762	0.000221902

Rv3698	hypothetical protein	-0.6	0.1	0.5	1	1	1	3.17E-06	0.228939336	3.38E-06
Rv1799	lppT	-0.1	-0.3	0.5	1	1	1	0.607326932	0.144613167	0.003842745
Rv3419c	gcp	0.1	0.0	0.5	1	1	1	0.450248855	0.857307965	6.42E-06
Rv3072c	hypothetical protein	0.0	0.4	0.5	1	1	1	0.823104224	0.024355432	6.30E-05
Rv3670	ephE	0.2	0.6	0.5	1	1	1	0.266350527	0.000181432	6.42E-05
Rv2877c	integral membrane protein	0.3	-0.5	0.5	1	1	1	0.042431762	0.000186384	8.15E-06
Rv3452	cut4	-0.6	-0.3	0.5	2	1	1	0.000157882	0.056953677	0.000119841
Rv0082	oxidoreductase	-1.9	0.7	0.5	4	2	1	1.16E-19	0.000521725	0.00055988
Rv1922	lipoprotein	0.1	-0.9	0.5	1	2	1	0.637775282	1.57E-11	7.79E-06
Rv0607	hypothetical protein	0.4	-0.4	0.5	1	1	1	0.013943422	0.011473696	0.000184241
Rv1081c	membrane protein	-1.5	0.5	0.5	3	1	1	6.26E-17	0.001304899	0.000464204
Rv1962c	vapC35	-0.2	0.1	0.5	1	1	1	0.20608293	0.734501858	3.68E-05
Rv3602c	panC	-0.5	0.3	0.5	1	1	1	0.009212967	0.060859018	9.22E-05
Rv1345	mbtM	0.4	0.1	0.5	1	1	1	0.003584463	0.436213969	1.25E-05
Rv0282	eccA3	-0.2	0.4	0.5	1	1	1	0.095385709	0.00030051	1.19E-05
Rv0218	transmembrane protein	-1.1	0.1	0.5	2	1	1	2.38E-11	0.606363159	9.61E-06
Rv2617c	transmembrane protein	0.6	-0.4	0.5	2	1	1	1.06E-05	0.00108588	0.000842402
Rv3318	sdhA	0.8	0.2	0.5	2	1	1	4.69E-09	0.058253914	1.21E-05
Rv1473	macrolide ABC transporter ATP-bindir	-0.2	1.0	0.5	1	2	1	0.202873723	1.41E-16	7.98E-06
Rv0181c	hypothetical protein	0.5	-0.1	0.5	1	1	1	0.000356976	0.519342146	9.73E-05
Rv2852c	mqo	0.5	-0.9	0.5	1	2	1	9.30E-06	2.45E-16	4.29E-05
Rv2694c	hypothetical protein	-0.6	-1.5	0.5	2	3	1	0.001285573	5.62E-47	7.55E-05
Rv2620c	transmembrane protein	-0.4	0.0	0.5	1	1	1	0.032976219	0.88891693	0.000142504
Rv3813c	hypothetical protein	0.7	-0.7	0.5	2	2	1	3.31E-05	1.20E-07	7.82E-05
Rv3068c	pgmA	0.1	-0.3	0.5	1	1	1	0.487506462	0.014729242	1.67E-05
Rv1913	hypothetical protein	0.4	-0.6	0.5	1	1	1	0.016488718	0.000317525	0.000403902
Rv1774	oxidoreductase	-0.6	0.3	0.5	2	1	1	4.28E-06	0.010228129	5.60E-05
Rv1976c	hypothetical protein	0.6	-1.0	0.5	2	2	1	2.08E-05	1.63E-12	0.00012668
Rv0294	tam	-0.3	-0.2	0.5	1	1	1	0.050001178	0.257700456	9.07E-05
Rv3607c	folB	0.2	0.1	0.5	1	1	1	0.109545157	0.334556859	0.000105947
Rv1523	methyltransferase	-0.5	0.9	0.5	1	2	1	0.001200713	1.68E-08	0.000157704
Rv1805c	hypothetical protein	0.1	-2.0	0.5	1	4	1	0.421868052	1.53E-48	2.68E-05
Rv3566c	nat	0.2	-0.9	0.5	1	2	1	0.191110597	4.19E-15	9.88E-05
Rv2746c	pgsA3	-0.6	0.1	0.5	2	1	1	6.63E-05	0.588011871	0.000164816
Rv3373	echA18	0.6	0.5	0.5	1	1	1	2.48E-05	0.003906403	0.000105905
Rv0237	lpqI	-0.1	1.5	0.5	1	3	1	0.62522152	2.63E-23	1.99E-05
Rv0307c	hypothetical protein	0.0	-2.4	0.5	1	5	1	0.838402561	1.93E-72	0.000650945
Rv0858c	dapC	0.0	-0.3	0.5	1	1	1	0.83631433	0.060761315	0.000303161
Rv2062c	cobN	-0.2	0.6	0.5	1	1	1	0.125658255	6.55E-06	1.09E-05
Rv0289	espG3	-0.8	0.4	0.5	2	1	1	3.09E-07	5.79E-05	5.96E-05
Rv2300c	metallo-hydrolase	0.0	0.0	0.5	1	1	1	0.787731996	0.842301271	0.000401176
Rv2040c	sugar ABC transporter permease	0.2	-0.9	0.5	1	2	1	0.116636655	2.95E-11	0.000171437
Rv1814	erg3	0.1	0.0	0.5	1	1	1	0.249146623	0.904369501	5.29E-05
Rv3300c	hypothetical protein	0.2	0.0	0.5	1	1	1	0.129307389	0.889305963	0.000120901
Rv0912	transmembrane protein	-0.2	-0.3	0.5	1	1	1	0.10338625	0.048260218	0.000283009
Rv2843	hypothetical protein	0.3	-0.2	0.5	1	1	1	0.060505476	0.129908337	0.00045189

Rv2419c	gpgP	0.4	0.0	0.5	1	1	1	0.014965825	0.994257747	0.000407528
Rv1228	lpqX	-0.7	0.0	0.5	2	1	1	0.000494347	0.820159006	5.06E-05
Rv0667	rpoB	0.5	0.5	0.5	1	1	1	2.09E-05	2.19E-06	0.000241122
Rv3437	transmembrane protein	0.1	-0.9	0.5	1	2	1	0.730972681	1.39E-07	0.000898889
Rv2500c	fadE19	0.1	-1.8	0.5	1	3	1	0.344214888	3.11E-58	0.000683272
Rv0537c	integral membrane protein	0.2	0.0	0.5	1	1	1	0.130399145	0.665490439	4.18E-05
Rv2958c	PGL/p-HBAD biosynthesis glycosyltra	0.6	0.7	0.5	2	2	1	4.60E-07	1.19E-08	0.000159365
Rv0582	vapC26	0.3	0.2	0.5	1	1	1	0.154020291	0.335667721	0.002775654
Rv1069c	hypothetical protein	0.3	0.8	0.5	1	2	1	0.005084281	2.01E-11	5.35E-05
Rv0069c	sdaA	0.4	1.1	0.5	1	2	1	0.001562069	8.09E-15	7.87E-05
Rv0275c	transcriptional regulator	-0.9	-1.4	0.5	2	3	1	1.24E-07	5.37E-29	0.000821784
Rv1072	transmembrane protein	-0.3	-0.9	0.4	1	2	1	0.001159707	1.24E-16	8.02E-05
Rv1767	hypothetical protein	0.6	-0.6	0.4	1	2	1	0.001990663	0.000244173	0.001176198
Rv0303	dehydrogenase/reductase	-0.1	0.3	0.4	1	1	1	0.558913172	0.014455276	0.000104527
Rv3627c	hypothetical protein	0.2	-1.1	0.4	1	2	1	0.235603835	6.83E-22	4.69E-05
Rv2872	vapC43	-0.4	0.1	0.4	1	1	1	0.021302609	0.426003913	0.001315748
Rv1423	whiA	1.1	-0.8	0.4	2	2	1	4.56E-14	4.25E-11	0.000150245
Rv2304c	hypothetical protein	0.6	-0.8	0.4	2	2	1	0.000449884	1.11E-05	0.003587877
Rv3572	hypothetical protein	-0.4	0.0	0.4	1	1	1	0.017112155	0.87400657	0.000350876
Rv0392c	ndhA	0.2	-0.1	0.4	1	1	1	0.079333353	0.366307874	6.87E-05
Rv1399c	nlhH	-0.5	0.4	0.4	1	1	1	0.000769865	0.014154846	0.000236905
Rv0941c	hypothetical protein	0.8	-1.2	0.4	2	2	1	1.31E-09	3.71E-22	0.00037996
Rv0792c	transcriptional regulator	-1.2	-0.3	0.4	2	1	1	1.20E-18	0.01250621	9.77E-05
Rv0284	eccC3	0.1	0.5	0.4	1	1	1	0.259205706	3.38E-07	0.000156528
Rv0343	iniC	-0.2	-0.3	0.4	1	1	1	0.064168214	0.01455806	5.21E-05
Rv3303c	lpdA	-0.4	0.2	0.4	1	1	1	0.007894669	0.082120085	0.000609544
Rv1760	diacylglycerol acyltransferase	0.1	-1.0	0.4	1	2	1	0.302107366	6.29E-20	0.000185729
Rv0624	vapC30	0.2	-0.3	0.4	1	1	1	0.238241976	0.029529405	0.00164599
Rv2662	hypothetical protein	-1.1	-0.4	0.4	2	1	1	1.76E-11	0.001963906	0.000351852
Rv0400c	fadE7	-0.3	-0.3	0.4	1	1	1	0.199088582	0.00980615	0.000358068
Rv3525c	siderophore-binding protein	-1.0	0.7	0.4	2	2	1	1.11E-09	3.86E-06	0.004742265
Rv1391	dfp	0.6	-0.9	0.4	2	2	1	3.46E-09	4.70E-18	0.000642924
Rv2914c	pknI	-0.5	0.4	0.4	1	1	1	0.000192625	0.002432291	0.000220641
Rv1800	PPE28	0.2	-1.4	0.4	1	3	1	0.147000929	2.22E-35	0.00017421
Rv0230c	php	-0.7	0.5	0.4	2	1	1	4.94E-06	8.99E-06	0.000161441
Rv1920	membrane protein	0.1	-0.6	0.4	1	2	1	0.549713078	8.24E-07	0.000556199
Rv1302	rfe	0.2	0.0	0.4	1	1	1	0.203303176	0.933270523	0.000128019
Rv0349	hypothetical protein	0.4	-0.2	0.4	1	1	1	0.006342828	0.060586034	0.002331186
Rv0926c	hypothetical protein	-0.2	0.3	0.4	1	1	1	0.13998949	0.045855127	0.000260998
Rv1681	moeX	0.3	-0.3	0.4	1	1	1	0.006921426	0.048501551	0.000294052
Rv1683	bifunctional long-chain acyl-CoA synt	0.3	-0.9	0.4	1	2	1	0.024501239	7.90E-17	6.25E-05
Rv3164c	moxR3	-0.1	-0.1	0.4	1	1	1	0.671881945	0.52135833	0.003400785
Rv1713	engA	0.0	1.1	0.4	1	2	1	0.930501551	3.02E-20	0.000309324
Rv1158c	hypothetical protein	-0.4	1.2	0.4	1	2	1	0.017483315	8.81E-12	0.001531893
Rv3889c	espG2	-0.1	0.5	0.4	1	1	1	0.670462354	0.001392237	0.000596482
Rv1524	glycosyltransferase	0.5	0.7	0.4	1	2	1	0.000106082	9.67E-06	0.000433559

Rv0666	membrane protein	-0.2	0.5	0.4	1	1	1	0.415750809	0.018445121	0.00701299
Rv3662c	hypothetical protein	-0.7	-1.0	0.4	2	2	1	1.78E-05	1.10E-17	0.000401491
Rv2514c	hypothetical protein	0.4	-0.1	0.4	1	1	1	0.00269692	0.703654096	0.001137899
Rv0110	integral membrane protein	-0.1	0.7	0.4	1	2	1	0.413101614	1.50E-07	0.000231443
Rv3885c	eccE2	0.3	0.6	0.4	1	1	1	0.016331747	1.12E-05	0.000578111
Rv0357c	purA	0.5	0.5	0.4	1	1	1	5.36E-05	0.000120334	0.00031302
Rv3354	hypothetical protein	-0.7	0.1	0.4	2	1	1	0.000292426	0.612288129	0.005208477
Rv1529	fadD24	0.2	-0.2	0.4	1	1	1	0.200957503	0.026160446	0.000612941
Rv0550c	vapB3	0.0	-0.8	0.4	1	2	1	0.954968557	1.95E-06	0.012278693
Rv2922c	smc	0.4	-0.3	0.4	1	1	1	0.000140054	0.001497074	0.000157478
Rv0610c	hypothetical protein	-0.7	0.5	0.4	2	1	1	1.79E-05	0.001750273	0.000629311
Rv1138c	oxidoreductase	0.0	0.2	0.4	1	1	1	0.8047459	0.254867856	0.001227429
Rv3496c	mce4D	0.5	0.7	0.4	1	2	1	4.26E-05	3.08E-07	0.000431773
Rv3061c	fadE22	0.5	-0.5	0.4	1	1	1	2.85E-05	9.86E-08	0.000159328
Rv0697	dehydrogenase	-1.3	1.0	0.4	2	2	1	2.40E-16	6.10E-11	0.000461333
Rv2266	cyp124	-0.4	-0.3	0.4	1	1	1	0.007949734	0.025130306	0.000524471
Rv0923c	hypothetical protein	0.7	-0.5	0.4	2	1	1	4.72E-07	2.92E-06	0.000332716
Rv1493	mutB	0.5	-0.9	0.4	1	2	1	0.000240137	9.05E-18	0.000293987
Rv0835	lpqQ	-0.2	0.2	0.4	1	1	1	0.355127599	0.132281442	0.0027673
Rv1284	canA	0.1	-2.1	0.4	1	4	1	0.620006132	2.44E-58	0.00374964
Rv1063c	NTE family protein	0.5	-1.6	0.4	1	3	1	0.002090754	1.18E-31	0.000910712
Rv0654	carotenoid cleavage oxygenase	-0.9	0.5	0.4	2	1	1	3.31E-12	8.16E-05	0.000461847
Rv3169	hypothetical protein	0.6	-0.5	0.4	1	1	1	0.001085555	6.39E-06	0.000499013
Rv1660	pks10	-0.4	0.2	0.4	1	1	1	0.011839269	0.145245213	0.000996048
Rv1428c	hypothetical protein	0.0	0.5	0.4	1	1	1	0.734271591	0.000585366	0.000971529
Rv1706c	PPE23	-0.7	-0.4	0.4	2	1	1	9.50E-05	0.003051152	0.000690816
Rv2884	transcriptional regulator	0.9	-0.8	0.4	2	2	1	2.58E-10	9.45E-10	0.001631431
Rv0677c	mmpS5	-1.5	0.4	0.4	3	1	1	3.14E-28	4.68E-05	0.01974603
Rv3488	hypothetical protein	-1.4	0.3	0.4	3	1	1	2.24E-09	0.199401938	0.012768172
Rv2478c	hypothetical protein	-0.7	0.1	0.4	2	1	1	3.01E-06	0.505139331	0.001513178
Rv2404c	lepA	0.5	0.4	0.4	1	1	1	6.28E-05	0.000163918	0.000333833
Rv3041c	ABC transporter ATP-binding protein	-0.2	1.0	0.4	1	2	1	0.162153223	7.81E-10	0.000858765
Rv1647	adenylate cyclase	-0.2	-0.2	0.4	1	1	1	0.116271775	0.071531465	0.001221682
Rv3689	transmembrane protein	-0.2	0.0	0.4	1	1	1	0.058990042	0.779951133	0.000755463
Rv2851c	GCN5-like N-acetyltransferase	0.6	-1.3	0.4	1	2	1	2.36E-06	2.48E-30	0.000581829
Rv1696	recN	0.2	0.5	0.4	1	1	1	0.148329974	8.03E-06	0.000422936
Rv2550c	vapB20	0.1	-0.2	0.4	1	1	1	0.705554901	0.340896446	0.007503957
Rv1983	PE PGRS35	0.1	-0.1	0.4	1	1	1	0.237635544	0.318828718	0.000881229
Rv0979c	hypothetical protein	-1.5	0.7	0.4	3	2	1	8.67E-12	0.002750645	0.010784246
Rv3324c	moaC3	-0.1	0.5	0.4	1	1	1	0.676462324	0.001607379	0.002748907
Rv0993	galU	-0.3	0.7	0.4	1	2	1	0.040229129	2.18E-09	0.000431788
Rv3772	hisC2	-0.5	0.4	0.4	1	1	1	0.000118019	0.003984859	0.001456609
Rv2034	ArsR family HTH-type transcriptional r	-2.4	0.2	0.4	5	1	1	9.73E-22	0.137148572	0.006269346
Rv0125	pepA	0.3	-0.2	0.4	1	1	1	0.010290538	0.078737633	0.00076752
Rv0128	transmembrane protein	0.4	0.2	0.4	1	1	1	0.001063086	0.097015601	0.001370091
Rv1985c	HTH-type transcriptional regulator	0.1	-0.3	0.4	1	1	1	0.457721142	0.024997344	0.000677327

Rv2972c	hypothetical protein	1.1	-0.1	0.4	2	1	1	1.44E-15	0.534427033	0.00238308
Rv3667	acs	0.5	-0.4	0.4	1	1	1	2.16E-06	0.000352347	0.000431132
Rv0321	dcd	-0.2	0.6	0.4	1	1	1	0.220660452	0.000605788	0.006037693
Rv1873	hypothetical protein	-1.3	0.3	0.4	2	1	1	1.92E-08	0.102993508	0.009721002
Rv2389c	rpfD	1.7	-0.7	0.4	3	2	1	4.33E-33	8.97E-07	0.006015633
Rv2433c	hypothetical protein	-0.2	0.1	0.4	1	1	1	0.177720703	0.619651665	0.008950768
Rv2633c	hypothetical protein	-0.1	-0.7	0.4	1	2	1	0.3171658	5.06E-08	0.013555414
Rv0743c	hypothetical protein	-0.3	-0.5	0.4	1	1	1	0.018794742	0.000186915	0.001912884
Rv3764c	tcrY	0.5	-0.5	0.4	1	1	1	0.000122274	4.22E-05	0.001775437
Rv3790	dprE1	-0.4	0.9	0.4	1	2	1	0.002390413	3.27E-11	0.001067564
Rv3906c	hypothetical protein	1.6	0.2	0.4	3	1	1	1.96E-17	0.144440715	0.006791837
Rv1346	mbtN	0.4	-0.2	0.4	1	1	1	0.00396657	0.046212344	0.001925933
Rv3498c	mce4B	0.2	0.8	0.4	1	2	1	0.283352143	1.45E-10	0.001254496
Rv2968c	integral membrane protein	1.2	-0.2	0.4	2	1	1	7.60E-20	0.045483636	0.001632367
Rv1048c	hypothetical protein	-1.0	-0.3	0.4	2	1	1	3.36E-11	0.009385727	0.002024069
Rv0671	lpqP	0.8	-2.3	0.4	2	5	1	1.49E-13	2.01E-124	0.00342524
Rv3761c	fadE36	0.1	0.0	0.4	1	1	1	0.492202094	0.955876155	0.002135913
Rv0421c	hypothetical protein	0.2	0.3	0.4	1	1	1	0.366478945	0.135931386	0.014636327
Rv2205c	hypothetical protein	0.1	-0.2	0.4	1	1	1	0.587921583	0.223186148	0.002206454
Rv0437c	psd	-0.2	-0.1	0.4	1	1	1	0.214492963	0.537830477	0.004849594
Rv3770c	hypothetical protein	0.7	-0.9	0.4	2	2	1	0.0001191	4.21E-11	0.005763755
Rv2614c	thrS	0.4	0.2	0.4	1	1	1	6.91E-05	0.064195202	0.000870713
Rv3692	moxR2	-0.1	0.2	0.4	1	1	1	0.263736113	0.087399647	0.004789485
Rv3671c	serine protease	-0.2	0.8	0.4	1	2	1	0.217401692	4.61E-09	0.002848865
Rv0992c	5-formyltetrahydrofolate cyclo-ligase	-1.4	0.4	0.4	3	1	1	2.01E-12	0.004668825	0.005168764
Rv3371	diacyglycerol O-acyltransferase	0.2	-0.4	0.4	1	1	1	0.143682767	0.001176773	0.003034042
Rv0265c	iron ABC transporter substrate-bindin	-0.5	0.0	0.4	1	1	1	0.008719766	0.759372109	0.004903816
Rv3527	hypothetical protein	0.7	-0.3	0.3	2	1	1	8.69E-05	0.034905628	0.014012613
Rv2618	hypothetical protein	0.1	-0.2	0.3	1	1	1	0.528714811	0.109098784	0.007439152
Rv3272	hypothetical protein	-0.5	1.3	0.3	1	2	1	7.20E-05	6.18E-34	0.002342111
Rv1250	MFS-type drug transporter	-0.2	-1.1	0.3	1	2	1	0.138307635	4.29E-22	0.00267042
Rv3056	dinP	-0.3	0.3	0.3	1	1	1	0.023303213	0.047625608	0.003101543
Rv0482	murB	-0.6	-0.1	0.3	2	1	1	6.51E-06	0.277891515	0.003099846
Rv0246	integral membrane protein	0.6	0.9	0.3	2	2	1	9.45E-07	2.11E-19	0.007192583
Rv3714c	hypothetical protein	0.6	0.7	0.3	2	2	1	5.37E-06	6.14E-07	0.004745642
Rv3330	dacB1	-0.5	0.4	0.3	1	1	1	0.001709377	0.001682568	0.003785046
Rv1996	universal stress protein	1.3	-1.2	0.3	3	2	1	3.02E-21	6.40E-23	0.018711579
Rv1962A	vapB35	0.0	-0.3	0.3	1	1	1	0.774943977	0.092176687	0.024709953
Rv0406c	beta lactamase-like protein	-0.5	0.3	0.3	1	1	1	0.002008917	0.051850454	0.010411863
Rv0348	transcriptional regulator	0.6	-0.3	0.3	1	1	1	2.91E-05	0.018327123	0.006096632
Rv1548c	PPE21	0.6	0.3	0.3	1	1	1	7.65E-06	0.004427441	0.002305407
Rv0994	moeA1	0.1	0.0	0.3	1	1	1	0.44091503	0.977528018	0.002747082
Rv3032A	hypothetical protein	0.6	1.3	0.3	1	2	1	0.000334545	4.77E-10	0.019188272
Rv1781c	malQ	0.0	-0.9	0.3	1	2	1	0.983798601	5.91E-13	0.003665613
Rv2597	membrane protein	-0.2	0.1	0.3	1	1	1	0.193353346	0.576338137	0.008180074
Rv0918	hypothetical protein	-0.4	-0.3	0.3	1	1	1	0.013680519	0.066927783	0.018178534

Rv0236c	aftD	-0.8	0.6	0.3	2	2	1	1.09E-06	6.16E-07	0.001628731
Rv2639c	integral membrane protein	0.1	-0.6	0.3	1	1	1	0.498767634	1.51E-06	0.0081597
Rv3335c	integral membrane protein	0.4	-0.3	0.3	1	1	1	0.000747021	0.023124013	0.004684863
Rv3840	transcriptional regulator	-0.4	0.0	0.3	1	1	1	0.055827129	0.795684715	0.023707873
Rv1928c	short-chain type dehydrogenase/redu	1.6	-1.5	0.3	3	3	1	2.23E-39	6.08E-35	0.006323508
Rv0311	hypothetical protein	0.4	-2.5	0.3	1	6	1	0.002813446	4.08E-120	0.004201367
Rv1273c	drug ABC transporter ATP-binding prot	-0.7	0.7	0.3	2	2	1	6.64E-09	4.95E-09	0.002737223
Rv1750c	fadD1	0.0	0.0	0.3	1	1	1	0.709617064	0.695795403	0.006716715
Rv3668c	protease	0.0	-0.1	0.3	1	1	1	0.847831799	0.319205873	0.013089979
Rv3221c	TB7.3	0.0	0.5	0.3	1	1	1	0.913614885	1.02E-07	0.015734121
Rv2819c	CRISPR type III-associated RAMP prot	1.1	-0.3	0.3	2	1	1	4.88E-15	0.027147758	0.012998248
Rv0516c	anti-anti-sigma factor	0.9	-0.5	0.3	2	1	1	3.33E-09	0.000769955	0.024790201
Rv2978c	transposase	0.4	-0.8	0.3	1	2	1	8.15E-05	1.80E-09	0.002374455
Rv2933	ppsC	-0.9	1.5	0.3	2	3	1	4.85E-17	8.63E-61	0.006170291
Rv0409	ackA	0.3	-0.3	0.3	1	1	1	0.086124663	0.014638347	0.007700067
Rv1282c	oppC	-0.1	0.0	0.3	1	1	1	0.625019944	0.760735668	0.007185448
Rv2522c	hypothetical protein	0.2	0.0	0.3	1	1	1	0.087310248	0.922219261	0.005291973
Rv3220c	two component sensor kinase	0.1	0.8	0.3	1	2	1	0.332701197	3.17E-14	0.002407684
Rv3705c	hypothetical protein	-0.2	0.4	0.3	1	1	1	0.322263761	0.004625435	0.011512053
Rv1704c	cycA	0.6	0.1	0.3	1	1	1	3.04E-06	0.27550591	0.00381093
Rv1959c	parE1	0.5	-1.1	0.3	1	2	1	0.000957198	3.35E-17	0.018668133
Rv0234c	gabD1	-0.1	-0.5	0.3	1	1	1	0.446206077	9.51E-05	0.005040121
Rv3397c	phyA	-0.4	-0.2	0.3	1	1	1	0.005139257	0.098145284	0.012364391
Rv3438	hypothetical protein	-0.7	0.5	0.3	2	1	1	2.55E-05	0.003265305	0.013914148
Rv1258c	multidrug-efflux transporter	-2.2	0.4	0.3	4	1	1	2.77E-49	0.0007853	0.00648478
Rv1151c	NAD-dependent protein deacylase	0.2	-0.2	0.3	1	1	1	0.319384358	0.201107736	0.010936818
Rv1166	lpqW	-0.2	0.0	0.3	1	1	1	0.199714941	0.743921778	0.003999685
Rv2660c	hypothetical protein	-2.6	-0.8	0.3	6	2	1	2.17E-67	7.99E-13	0.007428997
Rv3862c	whiB6	-1.5	-0.3	0.3	3	1	1	2.55E-10	0.092415285	0.029176122
Rv3269	hypothetical protein	0.0	-0.1	0.3	1	1	1	0.877334178	0.773808374	0.062944301
Rv1228a	hypothetical protein CDS	0.0	0.2	0.3	1	1	1	0.76757919	0.106628647	0.020064032
Rv1377c	transferase	0.4	0.2	0.3	1	1	1	0.006902813	0.256115921	0.015948605
Rv3226c	hypothetical protein	0.8	-0.7	0.3	2	2	1	1.58E-09	8.29E-08	0.011807086
Rv0365c	hypothetical protein	0.0	0.3	0.3	1	1	1	0.763561826	0.010443884	0.016991512
Rv0204c	transmembrane protein	0.4	0.6	0.3	1	2	1	0.000260072	3.64E-08	0.006429881
Rv0115	hddA	0.7	0.1	0.3	2	1	1	7.05E-07	0.352590847	0.006937981
Rv0391	metZ	0.3	0.2	0.3	1	1	1	0.046620078	0.137715136	0.010450657
Rv0549c	vapC3	0.6	-1.1	0.3	1	2	1	5.92E-05	2.43E-16	0.025006861
Rv3126c	hypothetical protein	-0.1	-0.1	0.3	1	1	1	0.751109213	0.667907376	0.030263647
Rv1597	hypothetical protein	0.6	-0.1	0.3	2	1	1	2.48E-06	0.657902227	0.010967287
Rv2734	hypothetical protein	1.0	0.8	0.3	2	2	1	4.84E-18	4.29E-09	0.010320162
Rv3307	deoD	0.1	0.2	0.3	1	1	1	0.685783228	0.204925846	0.023142639
Rv1113	vapB32	0.0	0.0	0.3	1	1	1	0.923117646	0.856819631	0.068608396
Rv1811	mgtC	0.7	0.0	0.3	2	1	1	2.94E-07	0.76976437	0.01654704
Rv1020	mfd	-0.7	0.0	0.3	2	1	1	1.69E-08	0.709632319	0.006036009
Rv2011c	hypothetical protein	0.0	-1.0	0.3	1	2	1	0.7079967	9.14E-19	0.025221916

Rv2495c	bkdC	0.4	-1.8	0.3	1	3	1	5.06E-05	8.12E-78	0.006189444
Rv3361c	hypothetical protein	0.7	0.3	0.3	2	1	1	1.34E-06	0.087362007	0.022742649
Rv1073	hypothetical protein	0.7	-1.2	0.3	2	2	1	1.03E-07	2.73E-36	0.010642122
Rv1591	transmembrane protein	0.0	0.9	0.3	1	2	1	0.716729091	4.51E-12	0.009913512
Rv3309c	upp	-0.3	-0.1	0.3	1	1	1	0.041167579	0.430699682	0.013901489
Rv1126c	hypothetical protein	0.9	-0.9	0.3	2	2	1	1.41E-10	9.66E-12	0.015715917
Rv1457c	antibiotic ABC transporter permease	-0.6	0.8	0.3	2	2	1	1.37E-05	2.15E-06	0.030927308
Rv3108	hypothetical protein	0.0	0.2	0.3	1	1	1	0.752549019	0.253889116	0.015802762
Rv1472	echA12	-0.3	0.7	0.3	1	2	1	0.005808252	3.58E-10	0.007062492
Rv1567c	membrane protein	-0.2	1.3	0.3	1	2	1	0.141818299	7.37E-18	0.053089937
Rv3609c	folE	0.2	-0.2	0.3	1	1	1	0.183086719	0.121069521	0.03876722
Rv0498	hypothetical protein	0.4	0.6	0.3	1	1	1	0.001039002	0.000131073	0.017105887
Rv1786	ferredoxin	0.1	-0.5	0.3	1	1	1	0.768830363	0.004785899	0.062998647
Rv1590	hypothetical protein	-0.5	0.7	0.3	1	2	1	0.05212508	0.000496101	0.052670546
Rv1430	PE16	0.5	-1.6	0.3	1	3	1	6.42E-05	1.83E-48	0.00963478
Rv3606c	folK	-0.3	0.3	0.3	1	1	1	0.104793934	0.062003665	0.014993526
Rv2403c	lppR	1.4	0.2	0.3	3	1	1	9.79E-36	0.113349854	0.013343451
Rv0459	hypothetical protein	-0.4	0.4	0.3	1	1	1	0.017369442	0.033164266	0.04672513
Rv1815	hypothetical protein	0.8	0.8	0.3	2	2	1	4.97E-10	2.45E-09	0.020472924
Rv0425c	ctpH	0.1	-0.3	0.3	1	1	1	0.493061836	0.000845801	0.010475783
Rv2308	hypothetical protein	0.5	-0.3	0.3	1	1	1	0.000143788	0.014967077	0.025586797
Rv0440	groEL2	1.0	-0.3	0.3	2	1	1	7.88E-17	0.002179019	0.008707403
Rv3799c	accD4	-0.2	0.5	0.3	1	1	1	0.086027676	1.08E-08	0.007569442
Rv2870c	dxr	0.2	-0.3	0.3	1	1	1	0.1007061	0.011588944	0.014453823
Rv2365c	hypothetical protein	0.0	-0.9	0.3	1	2	1	0.9966517	6.61E-08	0.074526694
Rv2049c	hypothetical protein	0.0	-0.1	0.3	1	1	1	0.826756758	0.448917305	0.063187082
Rv3626c	hypothetical protein	-0.1	-0.9	0.3	1	2	1	0.412509703	4.34E-10	0.019199013
Rv0982	mprB	0.5	-2.0	0.3	1	4	1	1.86E-05	2.22E-82	0.018412389
Rv2898c	hypothetical protein	0.7	0.1	0.3	2	1	1	4.57E-06	0.738426759	0.045196398
Rv2447c	folC	0.6	0.3	0.3	1	1	1	0.000379203	0.014601614	0.020332161
Rv0163	hypothetical protein	-0.7	0.6	0.3	2	2	1	6.45E-05	0.000328613	0.062019474
Rv2307B	hypothetical protein	-0.4	0.4	0.3	1	1	1	0.025143595	0.027533528	0.066485065
Rv2672	protease	-0.3	0.7	0.3	1	2	1	0.014152573	1.21E-09	0.01897734
Rv2563	glutamine ABC transporter permease	-0.2	0.9	0.3	1	2	1	0.198362281	8.89E-08	0.02569464
Rv2524c	fas	-0.9	1.1	0.3	2	2	1	6.67E-10	2.43E-33	0.011864639
Rv3603c	hypothetical protein	-0.6	0.4	0.3	2	1	1	0.000192882	0.004329943	0.027482384
Rv2228c	multifunctional RNASE H/alpha-ribazo	0.6	0.6	0.3	2	1	1	1.31E-07	2.71E-05	0.029235004
Rv0296c	sulfatase	0.3	0.3	0.3	1	1	1	0.008557833	0.006694697	0.025442145
Rv0901	arfC	-0.3	0.0	0.3	1	1	1	0.053044176	0.900805169	0.045167893
Rv2301	cut2	0.0	0.2	0.3	1	1	1	0.984150772	0.155902905	0.091972507
Rv2568c	hypothetical protein	0.1	0.2	0.3	1	1	1	0.443557016	0.104080853	0.035043777
Rv1680	hypothetical protein	0.5	0.2	0.3	1	1	1	0.000126817	0.208212964	0.048440913
Rv0569	hypothetical protein	1.3	-1.1	0.2	2	2	1	4.62E-23	1.56E-20	0.033202454
Rv0899	arfA	-0.2	-0.4	0.2	1	1	1	0.066159009	0.004048099	0.033092962
Rv3900c	hypothetical protein	0.1	0.3	0.2	1	1	1	0.519478786	0.010147136	0.034006036
Rv3911	sigM	-0.8	0.5	0.2	2	1	1	5.51E-07	0.000580101	0.056169878

Rv0435c	ATPase	0.2	0.4	0.2	1	1	1	0.143415761	0.000881078	0.03186283
Rv3065	mmr	-0.6	0.6	0.2	1	2	1	0.001350705	0.000485342	0.099472716
Rv1892	membrane protein	0.6	-0.8	0.2	2	2	1	4.67E-05	9.82E-08	0.076189276
Rv2976c	ung	1.2	0.0	0.2	2	1	1	7.53E-19	0.785502566	0.050157669
Rv0408	pta	0.4	0.7	0.2	1	2	1	0.000610386	3.42E-10	0.035958472
Rv3568c	hsaC	0.2	-0.8	0.2	1	2	1	0.082389621	4.42E-11	0.034992555
Rv3078	hab	0.0	-0.7	0.2	1	2	1	0.980114478	1.67E-05	0.094359587
Rv3531c	hypothetical protein	-0.3	-0.8	0.2	1	2	1	0.034784719	4.69E-12	0.038543195
Rv1319c	adenylate cyclase	-0.3	1.3	0.2	1	2	1	0.007947189	1.47E-30	0.052711129
Rv1237	sugB	0.1	-0.3	0.2	1	1	1	0.576238231	0.014133386	0.068367107
Rv2611c	phosphatidylinositol mannoside acylt	0.5	0.3	0.2	1	1	1	0.003225336	0.053775946	0.055926355
Rv0825c	hypothetical protein	0.2	-0.7	0.2	1	2	1	0.098303549	9.11E-09	0.064518059
Rv1950c	hypothetical protein	1.1	-0.3	0.2	2	1	1	4.02E-09	0.211735159	0.214243522
Rv2916c	ffh	0.2	-0.6	0.2	1	2	1	0.115405372	1.14E-07	0.069686807
Rv2494	vapC38	0.7	-1.8	0.2	2	4	1	2.19E-07	1.93E-70	0.047646223
Rv1650	pheT	0.4	0.4	0.2	1	1	1	0.00322831	0.001079666	0.035440753
Rv3198c	uvrD2	-0.1	0.6	0.2	1	1	1	0.536744184	2.50E-06	0.039985911
Rv1082	mca	-0.2	-1.2	0.2	1	2	1	0.077547156	1.06E-24	0.03370559
Rv2847c	cysG	-1.1	-0.3	0.2	2	1	1	1.98E-16	0.013914319	0.057684926
Rv2377c	hypothetical protein	1.2	-1.7	0.2	2	3	1	2.21E-09	6.03E-17	0.1975927
Rv1318c	adenylate cyclase	-0.9	1.4	0.2	2	3	1	1.83E-06	5.89E-31	0.052636206
Rv0030	hypothetical protein	0.2	-0.5	0.2	1	1	1	0.218487666	0.000324516	0.090424225
Rv2511	orn	-1.0	0.2	0.2	2	1	1	2.91E-09	0.192528568	0.088878653
Rv1077	cbs	0.3	0.2	0.2	1	1	1	0.01550423	0.040359585	0.04113943
Rv3451	cut3	0.3	-0.3	0.2	1	1	1	0.016898182	0.006098643	0.057154275
Rv1606	hisI	0.0	-0.7	0.2	1	2	1	0.77848417	5.45E-07	0.077478347
Rv3879c	espK	-0.6	0.0	0.2	1	1	1	4.80E-06	0.733394677	0.055444771
Rv3403c	hypothetical protein	0.0	0.6	0.2	1	1	1	0.978266718	3.10E-06	0.067025209
Rv1005c	para-aminobenzoate synthase compo	-1.1	0.3	0.2	2	1	1	2.47E-10	0.017123686	0.062409544
Rv0996	transmembrane protein	0.3	-0.6	0.2	1	2	1	0.048156972	5.67E-10	0.098014314
Rv3907c	pcnA	1.2	0.6	0.2	2	2	1	4.73E-25	3.26E-05	0.058722473
Rv2337c	hypothetical protein	0.6	0.2	0.2	2	1	1	2.95E-06	0.165083292	0.070974358
Rv3815c	acyltransferase	1.0	-1.2	0.2	2	2	1	2.01E-12	3.14E-25	0.099857158
Rv0310c	hypothetical protein	0.6	-0.1	0.2	2	1	1	8.76E-06	0.577562566	0.112336045
Rv2899c	fdhD	0.8	0.0	0.2	2	1	1	2.23E-09	0.92812827	0.0830207
Rv1232c	hypothetical protein	0.1	0.2	0.2	1	1	1	0.46800399	0.032104959	0.053649239
Rv1490	membrane protein	0.1	0.4	0.2	1	1	1	0.583899926	0.001826246	0.073226456
Rv1909c	furA	0.7	-2.0	0.2	2	4	1	2.39E-08	3.33E-75	0.144088007
Rv2090	5'-3' exonuclease	0.6	-0.3	0.2	2	1	1	4.24E-06	0.009797743	0.075190591
Rv0675	echA5	-0.7	0.2	0.2	2	1	1	4.72E-07	0.088358545	0.060793278
Rv3896c	hypothetical protein	-0.8	0.2	0.2	2	1	1	2.14E-05	0.21389473	0.127057376
Rv1366	hypothetical protein	0.1	0.3	0.2	1	1	1	0.410665735	0.025267112	0.082007432
Rv2344c	dgt	-0.6	0.1	0.2	2	1	1	0.000134303	0.398717169	0.085023492
Rv0492A	hypothetical protein	-1.9	0.5	0.2	4	1	1	1.27E-17	0.011392803	0.158273068
Rv2786c	ribF	0.4	0.1	0.2	1	1	1	0.000469613	0.68312551	0.085947658
Rv3231c	hypothetical protein	-0.4	0.5	0.2	1	1	1	0.014432027	0.00557067	0.142620436

Rv2402	trehalase	0.6	0.1	0.2	1	1	1	6.81E-06	0.315127412	0.075830933
Rv3754	tyrA	0.2	-0.3	0.2	1	1	1	0.075785341	0.006465584	0.086691549
Rv2830c	vapB22	-0.5	-0.1	0.2	1	1	1	0.00518404	0.322005634	0.163520074
Rv3103c	hypothetical protein	-0.8	0.2	0.2	2	1	1	7.05E-06	0.148118084	0.238413226
Rv2188c	pimB	-0.1	-0.5	0.2	1	1	1	0.387623171	0.000114179	0.096940908
Rv2163c	pbpB	0.5	-0.3	0.2	1	1	1	1.10E-06	0.001769628	0.074459984
Rv3160c	TetR family transcriptional regulator	-0.2	1.0	0.2	1	2	1	0.079281897	6.66E-24	0.087582206
Rv2724c	fadE20	0.0	0.5	0.2	1	1	1	0.91322244	2.37E-07	0.131802509
Rv1179c	hypothetical protein	0.1	0.1	0.2	1	1	1	0.5850978	0.173619602	0.064196322
Rv0383c	hypothetical protein	-0.8	0.6	0.2	2	1	1	1.17E-10	1.60E-08	0.085722219
Rv0272c	hypothetical protein	0.3	-1.3	0.2	1	3	1	0.023999461	1.30E-34	0.083321428
Rv3696c	glpK	0.0	0.0	0.2	1	1	1	0.727964321	0.874667887	0.0852592
Rv1634	multidrug-efflux transporter	0.0	-1.6	0.2	1	3	1	0.721173392	4.56E-34	0.112510793
Rv2330c	lppP	-0.1	-0.4	0.2	1	1	1	0.670041827	0.000856937	0.112033942
Rv2448c	valS	0.6	0.0	0.2	2	1	1	1.61E-07	0.657396372	0.064296146
Rv0946c	pgi	0.0	0.2	0.2	1	1	1	0.679729003	0.089847165	0.100627492
Rv1270c	lprA	0.1	0.6	0.2	1	2	1	0.379798591	9.49E-06	0.103564236
Rv0554	bpoC	1.3	0.9	0.2	2	2	1	9.42E-20	1.01E-08	0.115229399
Rv0845	sensor histidine kinase NarS	-0.2	0.3	0.2	1	1	1	0.275556199	0.027263475	0.093840108
Rv0793	monooxygenase	-1.6	0.3	0.2	3	1	1	1.10E-14	0.118425164	0.172085459
Rv3124	moaR1	-0.8	0.5	0.2	2	1	1	0.000100433	0.007295038	0.267049302
Rv2411c	hypothetical protein	0.1	0.0	0.2	1	1	1	0.447023326	0.853434502	0.090927568
Rv3914	trxC	2.1	-0.6	0.2	4	2	1	7.96E-50	1.60E-07	0.124080256
Rv1648	transmembrane protein	0.3	-0.2	0.2	1	1	1	0.046808221	0.04056483	0.098122788
Rv0988	hypothetical protein	0.3	1.1	0.2	1	2	1	0.010664162	5.97E-23	0.155479896
Rv3765c	tcrX	0.4	-0.6	0.2	1	2	1	0.002519722	9.08E-07	0.110150081
Rv0155	pntAa	-0.8	1.5	0.2	2	3	1	1.32E-09	2.31E-20	0.10435083
Rv1378c	hypothetical protein	-0.7	0.4	0.2	2	1	1	0.001168971	0.250045865	0.630588994
Rv0044c	oxidoreductase	0.6	0.6	0.2	1	1	1	1.45E-05	6.36E-05	0.129539496
Rv0949	uvrD1	0.5	-0.2	0.2	1	1	1	1.33E-05	0.075748584	0.09716616
Rv2420c	hypothetical protein	0.8	0.1	0.2	2	1	1	2.14E-08	0.485584894	0.178475299
Rv3510c	hypothetical protein	-0.1	0.4	0.2	1	1	1	0.427180497	0.001425205	0.171511936
Rv2997	dehydrogenase	0.7	-0.7	0.2	2	2	1	1.38E-09	1.35E-10	0.147594188
Rv1242	vapC33	0.7	0.9	0.2	2	2	1	4.22E-08	2.34E-09	0.138206878
Rv1199c	insertion sequence element IS1081 tr	0.3	0.5	0.2	1	1	1	0.024081961	4.78E-06	0.111643475
Rv0882	transmembrane protein	-0.6	0.4	0.2	2	1	1	0.003340243	0.087078644	0.259245417
Rv3115	transposase	0.3	0.5	0.2	1	1	1	0.020339848	4.55E-06	0.113879395
Rv1422	hypothetical protein	0.8	-0.5	0.2	2	1	1	2.19E-10	4.20E-05	0.117307937
Rv0159c	PE3	0.3	0.6	0.2	1	1	1	0.039567685	1.92E-06	0.123495344
Rv1679	fadE16	0.4	0.2	0.2	1	1	1	0.002691472	0.070375991	0.167514816
Rv3756c	proZ	0.5	-0.2	0.2	1	1	1	0.005464139	0.17604489	0.161262577
Rv2079	hypothetical protein	-0.3	0.6	0.2	1	2	1	0.007957025	1.11E-07	0.113274181
Rv3726	dehydrogenase	0.8	0.6	0.2	2	1	1	4.15E-09	1.27E-05	0.167348098
Rv1143	mcr	0.1	0.5	0.2	1	1	1	0.404841639	0.000295842	0.214393284
Rv0405	pks6	0.7	0.4	0.2	2	1	1	1.46E-05	0.000228555	0.205562181
Rv3848	transmembrane protein	0.5	-1.6	0.2	1	3	1	0.001572292	5.11E-46	0.147218266

Rv2416c	eis	-1.9	0.8	0.2	4	2	1	1.74E-40	7.10E-10	0.161738471
Rv0200	transmembrane protein	-0.2	0.4	0.2	1	1	1	0.261244753	0.003672714	0.169762507
Rv0842	integral membrane protein	-2.4	-0.4	0.2	5	1	1	5.91E-47	0.004542613	0.216301946
Rv3382c	4-hydroxy-3-methylbut-2-enyl diphosp	-0.3	-0.3	0.2	1	1	1	0.025873345	0.026102236	0.160058387
Rv2085	hypothetical protein	-0.4	-0.2	0.2	1	1	1	0.014690374	0.250197756	0.245076498
Rv1207	folP2	0.1	0.1	0.2	1	1	1	0.593835125	0.368954771	0.20310221
Rv1365c	rsfA	0.1	0.0	0.2	1	1	1	0.539261668	0.806476355	0.281296437
Rv1532c	hypothetical protein	0.2	0.2	0.2	1	1	1	0.156309513	0.157598267	0.222556215
Rv3901c	membrane protein	0.3	0.1	0.2	1	1	1	0.034741567	0.325259895	0.189175646
Rv2761c	hsdS	0.1	0.2	0.2	1	1	1	0.276719779	0.094224061	0.181957102
Rv1010	ksgA	0.4	0.5	0.2	1	1	1	0.011045477	0.001044472	0.19912379
Rv1897c	D-tyrosyl-tRNA(Tyr) deacylase	0.3	-0.3	0.2	1	1	1	0.087599439	0.057755071	0.252126605
Rv0547c	oxidoreductase	-0.3	0.0	0.2	1	1	1	0.156378969	0.752041208	0.21050303
Rv3647c	hypothetical protein	-0.3	-0.1	0.2	1	1	1	0.100296067	0.611437652	0.219423286
Rv1914c	hypothetical protein	0.9	-0.3	0.2	2	1	1	1.49E-08	0.03802923	0.265341054
Rv2510c	hypothetical protein	-0.2	0.9	0.1	1	2	1	0.217444746	4.02E-14	0.190952717
Rv2443	dctA	-0.1	0.1	0.1	1	1	1	0.269781136	0.186038452	0.214366954
Rv2506	TetR family transcriptional regulator	-0.1	-0.3	0.1	1	1	1	0.713698037	0.031002159	0.264467235
Rv0256c	PPE2	-0.6	0.1	0.1	1	1	1	1.62E-06	0.611827657	0.228826696
Rv3275c	purE	0.3	0.1	0.1	1	1	1	0.049465305	0.61515564	0.284478344
Rv0245	oxidoreductase	0.0	-1.1	0.1	1	2	1	0.823112944	1.73E-22	0.288873298
Rv2793c	truB	-0.9	-0.1	0.1	2	1	1	2.94E-05	0.367315938	0.270988552
Rv3632	membrane protein	0.1	0.4	0.1	1	1	1	0.695494024	0.015359468	0.322641351
Rv2557	hypothetical protein	0.9	-1.8	0.1	2	3	1	5.18E-12	9.03E-60	0.25006275
Rv3898c	hypothetical protein	0.4	0.1	0.1	1	1	1	0.014208107	0.568848557	0.308093848
Rv1047	transposase	0.3	0.4	0.1	1	1	1	0.015689875	1.77E-05	0.212813788
Rv3023c	transposase	0.3	0.4	0.1	1	1	1	0.015985299	1.81E-05	0.213156326
Rv0394c	hypothetical protein	-0.4	0.4	0.1	1	1	1	0.006374054	0.003691869	0.249069064
Rv2512c	insertion sequence element IS1081 tr	0.3	0.4	0.1	1	1	1	0.01526373	1.77E-05	0.214276003
Rv0801	hypothetical protein	-0.6	0.6	0.1	1	1	1	0.019494059	0.000855405	0.337160555
Rv3721c	dnaZX	0.6	-0.5	0.1	2	1	1	3.15E-06	1.05E-06	0.211846463
Rv1366A	hypothetical protein	-0.1	-0.4	0.1	1	1	1	0.751196892	0.032436047	0.381304088
Rv2996c	serA1	0.0	-1.0	0.1	1	2	1	0.764259607	2.48E-19	0.241902938
Rv3238c	integral membrane protein	0.2	-1.0	0.1	1	2	1	0.195583539	2.77E-14	0.307651939
Rv0064	transmembrane protein	0.4	-1.8	0.1	1	4	1	0.000539965	9.95E-72	0.31800882
Rv2054	hypothetical protein	-0.2	1.0	0.1	1	2	1	0.257169807	4.37E-11	0.313460609
Rv2733c	(dimethylallyl)adenosine tRNA methyl	-0.1	0.4	0.1	1	1	1	0.391721322	0.00032966	0.228049888
Rv0959	hypothetical protein	-0.7	-0.4	0.1	2	1	1	4.31E-07	0.000690906	0.278016889
Rv0441c	hypothetical protein	-0.4	-0.8	0.1	1	2	1	0.024427561	9.25E-06	0.463094997
Rv2332	mez	0.0	0.3	0.1	1	1	1	0.940417184	0.020213935	0.262042998
Rv0150c	hypothetical protein	0.6	-0.7	0.1	2	2	1	0.000781205	2.08E-06	0.371514937
Rv3362c	ATP/GTP-binding protein	0.6	1.0	0.1	1	2	1	0.000101686	1.03E-09	0.340928408
Rv0839	hypothetical protein	-2.0	0.6	0.1	4	1	1	1.43E-32	2.20E-06	0.273960174
Rv0224c	methyltransferase	-0.6	-0.4	0.1	1	1	1	0.005668527	0.001847417	0.335476951
Rv2678c	hemE	-0.3	-0.3	0.1	1	1	1	0.107141968	0.016457272	0.308100238
Rv2212	adenylyl cyclase	-0.7	0.2	0.1	2	1	1	6.29E-05	0.107991145	0.322372507

Rv1024	membrane protein	0.1	0.5	0.1	1	1	1	0.366017042	0.000956082	0.316151152
Rv3718c	hypothetical protein	0.9	0.7	0.1	2	2	1	4.49E-12	4.14E-05	0.349125827
Rv3518c	cyp142	0.5	0.4	0.1	1	1	1	2.20E-05	0.001111054	0.309611913
Rv0533c	fabH	-0.9	0.9	0.1	2	2	1	1.77E-11	4.13E-14	0.293126397
Rv2400c	subI	0.4	0.4	0.1	1	1	1	0.001421454	0.000517837	0.301991541
Rv3334	MerR family transcriptional regulator	-0.3	-1.1	0.1	1	2	1	0.032412589	1.61E-20	0.323753676
Rv1364c	sigma factor regulatory protein	0.1	-1.2	0.1	1	2	1	0.522549524	5.12E-24	0.3475745
Rv0213c	methyltransferase	0.3	-0.5	0.1	1	1	1	0.010006645	2.20E-06	0.328109961
Rv0803	purL	0.2	0.6	0.1	1	1	1	0.116090566	3.95E-07	0.329289561
Rv2722	hypothetical protein	-0.8	1.2	0.1	2	2	1	4.59E-07	9.79E-14	0.368528982
Rv2143	hypothetical protein	-0.3	-0.3	0.1	1	1	1	0.0598534	0.014760175	0.362224955
Rv2018	hypothetical protein	0.2	-0.1	0.1	1	1	1	0.084162445	0.598933972	0.344685549
Rv2885c	transposase	1.1	-1.1	0.1	2	2	1	4.02E-18	7.67E-16	0.316775592
Rv2176	pknL	-0.3	-0.4	0.1	1	1	1	0.020916156	0.000994225	0.353971561
Rv2918c	glnD	0.0	-1.8	0.1	1	3	1	0.873074486	5.61E-62	0.319500607
Rv1712	cmk	0.0	1.1	0.1	1	2	1	0.966872897	2.63E-18	0.35532369
Rv1894c	hypothetical protein	1.6	-1.5	0.1	3	3	1	1.27E-36	1.37E-45	0.334559292
Rv2762c	hypothetical protein	0.7	0.0	0.1	2	1	1	1.93E-05	0.925517036	0.453809851
Rv2410c	hypothetical protein	0.8	0.0	0.1	2	1	1	2.80E-09	0.694517464	0.376446976
Rv3672c	hypothetical protein	0.2	0.6	0.1	1	1	1	0.25246838	8.48E-05	0.394548091
Rv1708	initiation inhibitor protein	0.1	1.1	0.1	1	2	1	0.481158788	6.29E-22	0.387268357
Rv3693	membrane protein	-0.2	-0.4	0.1	1	1	1	0.070410959	0.000313688	0.379600375
Rv2120c	integral membrane protein	0.3	0.2	0.1	1	1	1	0.076511583	0.119679861	0.459541992
Rv0854	hypothetical protein	-0.2	-0.2	0.1	1	1	1	0.168168942	0.084105707	0.442129337
Rv0447c	ufaA1	-0.5	0.6	0.1	1	2	1	0.000629165	1.12E-06	0.368689736
Rv1350	fabG2	0.0	-0.6	0.1	1	2	1	0.958745885	8.48E-07	0.400071011
Rv0285	PE5	-0.5	0.6	0.1	1	2	1	0.000681091	2.11E-06	0.407536446
Rv3328c	sigJ	-0.9	0.5	0.1	2	1	1	5.47E-07	0.000435035	0.448520193
Rv3254	hypothetical protein	-0.3	0.1	0.1	1	1	1	0.023396212	0.231417585	0.416532058
Rv2133c	hypothetical protein	0.3	-1.6	0.1	1	3	1	0.008610742	5.96E-33	0.443392277
Rv1071c	echA9	-0.4	0.9	0.1	1	2	1	0.003682582	1.61E-11	0.428393418
Rv3733c	hypothetical protein	0.3	-0.2	0.1	1	1	1	0.037284248	0.106038504	0.465099944
Rv3499c	mce4A	0.5	0.7	0.1	1	2	1	2.94E-06	5.90E-09	0.431049305
Rv2140c	TB18.6	0.2	0.0	0.1	1	1	1	0.085828367	0.93534505	0.485609254
Rv0439c	dehydrogenase/reductase	-0.6	1.0	0.1	2	2	1	5.96E-05	3.95E-11	0.50104122
Rv1287	HTH-type transcriptional regulator	-0.1	-0.5	0.1	1	1	1	0.552904574	0.000389429	0.492391422
Rv1978	hypothetical protein	0.5	-0.1	0.1	1	1	1	0.000861398	0.315824593	0.45950776
Rv2716	hypothetical protein	-0.6	1.3	0.1	1	2	1	1.32E-05	7.57E-30	0.46762848
Rv1281c	oppD	-0.1	-0.5	0.1	1	1	1	0.586351204	6.57E-06	0.447302782
Rv1862	adhA	-0.1	0.0	0.1	1	1	1	0.501449798	0.745822694	0.463555903
Rv3600c	type III pantothenate kinase	0.0	0.3	0.1	1	1	1	0.964798294	0.028998131	0.522518196
Rv0024	NLP/P60 family protein	-0.1	0.5	0.1	1	1	1	0.393172136	0.000348034	0.510447274
Rv1580c	phage protein	-0.3	0.2	0.1	1	1	1	0.091904196	0.425244772	0.600191554
Rv0727c	fucA	-0.9	-0.5	0.1	2	1	1	2.40E-08	0.0001307	0.536865
Rv2394	ggtB	0.5	0.2	0.1	1	1	1	0.000921754	0.196975122	0.472865808
Rv3081	hypothetical protein	-0.5	-0.7	0.1	1	2	1	8.20E-05	5.41E-10	0.484182779

Rv0334	rmlA	0.2	0.5	0.1	1	1	1	0.176131922	4.25E-05	0.500329988
Rv0342	iniA	-0.7	0.0	0.1	2	1	1	5.34E-11	0.688075106	0.472938422
Rv2995c	leuB	-0.2	-0.8	0.1	1	2	1	0.256089001	1.40E-07	0.539726317
Rv0816c	thiX	0.7	0.2	0.1	2	1	1	3.30E-06	0.177705682	0.594104348
Rv2351c	plcA	-0.5	0.6	0.1	1	2	1	0.000118808	8.92E-08	0.544972815
Rv0583c	lpqN	0.5	0.0	0.1	1	1	1	0.001539899	0.969781541	0.557632536
Rv1413	hypothetical protein	-0.3	-0.6	0.1	1	1	1	0.100505095	0.000877284	0.634932351
Rv2866	relG	-0.3	-0.7	0.1	1	2	1	0.263087192	6.50E-05	0.661167089
Rv1824	hypothetical protein	0.2	0.6	0.1	1	1	1	0.127602939	0.000991345	0.615759063
Rv1061	hypothetical protein	0.0	0.0	0.1	1	1	1	0.990397133	0.995207417	0.621715115
Rv0877	hypothetical protein	0.4	-0.8	0.1	1	2	1	0.003096122	3.78E-09	0.59987174
Rv1695	ppnK	0.2	0.8	0.1	1	2	1	0.160526623	8.27E-10	0.565892609
Rv2582	ppiB	0.5	-0.1	0.1	1	1	1	5.58E-05	0.258964946	0.608463777
Rv2409c	hypothetical protein	0.6	0.1	0.1	2	1	1	3.07E-06	0.636457452	0.61363524
Rv3243c	hypothetical protein	0.4	-0.6	0.1	1	2	1	0.005511829	3.15E-07	0.620175754
Rv2435c	cyclase	-0.8	0.6	0.1	2	2	1	1.30E-10	3.31E-08	0.593503104
Rv2221c	glnE	0.3	-0.1	0.1	1	1	1	0.015953774	0.210097162	0.625128413
Rv2621c	transcriptional regulator	-1.2	0.3	0.1	2	1	1	8.29E-17	0.019740246	0.678783806
Rv2469c	hypothetical protein	-0.4	0.0	0.1	1	1	1	0.004478437	0.730650108	0.669053822
Rv3779	transmembrane protein	-0.1	0.3	0.1	1	1	1	0.490653852	0.011836043	0.632517535
Rv1988	erm(37)	0.3	0.5	0.1	1	1	1	0.039327874	0.000107714	0.685611279
Rv1864c	hypothetical protein	0.4	0.5	0.1	1	1	1	0.001294219	0.000886745	0.679112805
Rv1328	glgP	0.3	0.5	0.1	1	1	1	0.019441394	3.28E-06	0.640915225
Rv1354c	hypothetical protein	0.6	-0.8	0.1	2	2	1	6.12E-08	3.31E-11	0.65098731
Rv3737	transmembrane protein	-1.1	0.1	0.0	2	1	1	2.25E-13	0.250744984	0.674094113
Rv1217c	tetronasin ABC transporter integral m	-0.9	1.6	0.0	2	3	1	3.49E-12	6.06E-25	0.673564856
Rv0528	transmembrane protein	-0.4	0.7	0.0	1	2	1	0.004445449	1.19E-10	0.689267801
Rv1181	pks4	0.5	-1.1	0.0	1	2	1	3.42E-07	2.40E-30	0.67107533
Rv0956	purN	0.1	-0.3	0.0	1	1	1	0.303071081	0.021951592	0.749913879
Rv0153c	ptbB	0.5	0.2	0.0	1	1	1	3.34E-05	0.042938051	0.718058478
Rv0223c	aldehyde dehydrogenase	0.2	-0.2	0.0	1	1	1	0.483657942	0.047708835	0.744253958
Rv0367c	hypothetical protein	-0.5	-0.9	0.0	1	2	1	0.004350797	8.15E-08	0.793831347
Rv1492	mutA	-0.3	-0.4	0.0	1	1	1	0.094427799	0.000138712	0.750639491
Rv1933c	fadE18	0.5	0.4	0.0	1	1	1	0.010956082	0.019068196	0.796012991
Rv1927	hypothetical protein	1.9	-1.8	0.0	4	4	1	3.03E-47	2.95E-60	0.775368143
Rv2558	hypothetical protein	0.3	-2.6	0.0	1	6	1	0.067801771	4.91E-99	0.769238885
Rv3161c	dioxygenase	-0.6	0.6	0.0	2	1	1	6.96E-05	5.13E-09	0.731978725
Rv1193	fadD36	0.6	-0.4	0.0	2	1	1	1.93E-07	0.000638249	0.762297709
Rv0665	vapC8	0.1	0.5	0.0	1	1	1	0.608071128	0.009431927	0.83050504
Rv3794	embA	0.0	1.3	0.0	1	2	1	0.949395861	3.20E-16	0.755656644
Rv3566A	hypothetical protein	0.6	-2.0	0.0	2	4	1	0.003802085	1.24E-45	0.846185392
Rv0783c	emrB	0.3	-0.7	0.0	1	2	1	0.010332634	2.13E-09	0.781116067
Rv0052	hypothetical protein	0.2	0.3	0.0	1	1	1	0.310123961	0.073107467	0.820997574
Rv1255c	HTH-type transcriptional regulator	-0.3	-0.2	0.0	1	1	1	0.083536346	0.274741178	0.832582915
Rv2303c	antibiotic-resistance protein	1.2	-0.3	0.0	2	1	1	5.92E-15	0.030564476	0.823113194
Rv1525	wbbL2	0.6	-0.2	0.0	1	1	1	3.31E-05	0.091847627	0.815101553

Rv2943A	transposase	0.2	1.2	0.0	1	2	1	0.118701875	4.34E-10	0.835451034
Rv0893c	S-adenosylmethionine-dependent me	-0.3	1.1	0.0	1	2	1	0.007687028	2.50E-17	0.803630711
Rv1473A	transcriptional regulator	0.7	0.3	0.0	2	1	1	0.000227259	0.110767743	0.870119633
Rv0203	hypothetical protein	0.1	0.8	0.0	1	2	1	0.472694084	6.64E-06	0.848376446
Rv1351	hypothetical protein	1.9	-2.5	0.0	4	6	1	1.48E-27	1.97E-61	0.878657592
Rv1841c	hypothetical protein	0.0	0.7	0.0	1	2	1	0.7713727	1.71E-09	0.839181358
Rv2984	ppk1	0.4	-0.3	0.0	1	1	1	0.000454842	0.005407204	0.840796813
Rv2273	transmembrane protein	-0.4	1.5	0.0	1	3	1	0.065308457	2.94E-10	0.911320689
Rv0568	cyp135B1	-0.6	-0.5	0.0	2	1	1	0.000119539	2.77E-05	0.883513041
Rv3585	radA	0.4	0.1	0.0	1	1	1	0.015317685	0.321093375	0.880669945
Rv1773c	transcriptional regulator	-0.4	0.2	0.0	1	1	1	0.013916132	0.108465394	0.90405812
Rv3704c	gshA	-0.4	-0.2	0.0	1	1	1	0.001489511	0.07852571	0.901109755
Rv0356c	hypothetical protein	0.0	1.0	0.0	1	2	1	0.78000686	4.33E-08	0.929539447
Rv1296	thrB	0.8	0.0	0.0	2	1	1	4.39E-09	0.779878129	0.919206567
Rv1861	transmembrane protein	0.1	0.1	0.0	1	1	1	0.60134107	0.427301817	0.935629548
Rv3418c	groES	0.4	-0.2	0.0	1	1	1	0.000685156	0.06618262	0.931677618
Rv0531	membrane protein	-0.1	0.2	0.0	1	1	1	0.524856056	0.298936603	0.936845978
Rv0273c	transcriptional regulator	0.2	-0.7	0.0	1	2	1	0.147943007	4.69E-07	0.963500323
Rv2683	hypothetical protein	0.5	0.5	0.0	1	1	1	0.000615945	1.56E-05	0.962575285
Rv3057c	short chain alcohol dehydrogenase/re	-0.3	0.9	0.0	1	2	1	0.014267786	1.60E-10	0.968413969
Rv0116c	ldtA	0.0	-1.4	0.0	1	3	1	0.906203164	5.98E-29	0.973894456
Rv2542	hypothetical protein	0.3	0.0	0.0	1	1	1	0.005744858	0.814123697	0.995056064
Rv1629	polA	0.2	-1.0	0.0	1	2	1	0.029859603	1.26E-19	0.989267013
Rv3073c	hypothetical protein	-0.6	0.0	0.0	2	1	1	0.019412329	0.822729828	0.984061988
Rv3401	glycosyl hydrolase	0.1	0.5	0.0	1	1	1	0.590897636	9.16E-06	0.97395204
Rv3736	AraC/XylS family transcriptional regul	0.0	-0.5	0.0	1	1	1	0.866610184	2.33E-05	0.971798795
Rv2187	fadD15	-0.1	-0.4	0.0	1	1	1	0.577767946	0.001337068	0.965825272
Rv2232	ptkA	-0.2	-0.4	0.0	1	1	1	0.23579882	0.000534908	0.966476613
Rv3212	hypothetical protein	-0.4	0.0	0.0	1	1	1	0.000288473	0.705632665	0.958713283
Rv0497	transmembrane protein	-0.1	0.3	0.0	1	1	1	0.700219301	0.026433237	0.957542748
Rv3304	hypothetical protein	0.0	0.4	0.0	1	1	1	0.756883777	0.004302567	0.95769334
Rv1180	pks3	-0.3	-1.5	0.0	1	3	1	0.013517697	1.40E-40	0.935713483
Rv3701c	egtD	-0.1	-0.1	0.0	1	1	1	0.403437568	0.264421654	0.939949503
Rv2564	glnQ	0.4	1.1	0.0	1	2	1	0.004002843	4.20E-14	0.931509984
Rv2446c	integral membrane protein	1.6	0.1	0.0	3	1	1	5.84E-25	0.448603402	0.930671009
Rv0446c	transmembrane protein	0.2	0.4	0.0	1	1	1	0.08480355	0.001562737	0.921100655
Rv2760c	vapB42	-1.0	0.4	0.0	2	1	1	0.000212155	0.138404425	0.945793518
Rv0999	hypothetical protein	-0.1	0.1	0.0	1	1	1	0.337556333	0.266031335	0.918604528
Rv2455c	2-oxoglutarate oxidoreductase subun	0.2	-0.6	0.0	1	2	1	0.061716224	6.01E-08	0.880060402
Rv3215	entC	0.0	0.2	0.0	1	1	1	0.845989589	0.052295726	0.883129233
Rv1279	GMC-type oxidoreductase	0.6	0.3	0.0	2	1	1	1.45E-07	0.025839936	0.875649015
Rv2413c	hypothetical protein	-0.1	-0.5	0.0	1	1	1	0.61885504	0.001638648	0.889389719
Rv2042c	hypothetical protein	0.8	-1.6	0.0	2	3	1	2.91E-08	2.62E-42	0.890789465
Rv1162	narH	-0.2	-1.1	0.0	1	2	1	0.12391121	8.97E-31	0.873574347
Rv2438A	hypothetical protein	-0.2	0.0	0.0	1	1	1	0.34727826	0.908927201	0.894821982
Rv2189c	hypothetical protein	0.4	0.4	0.0	1	1	1	0.031026757	0.013961355	0.880600713

Rv0581	vapB26	-1.8	0.3	0.0	4	1	1	3.14E-34	0.077383742	0.867478696
Rv1070c	echA8	-0.3	1.4	0.0	1	3	1	0.02760387	1.94E-28	0.848704682
Rv0481c	hypothetical protein	-0.1	-0.3	0.0	1	1	1	0.341497361	0.057501409	0.864864993
Rv3735	hypothetical protein	0.7	-1.6	0.0	2	3	1	1.10E-05	9.35E-35	0.855073712
Rv2698	transmembrane protein	-0.2	0.3	0.0	1	1	1	0.437743915	0.075210566	0.86058325
Rv0773c	ggtA	0.1	0.8	0.0	1	2	1	0.546360956	1.11E-12	0.82711677
Rv1059	hypothetical protein	0.0	-0.3	0.0	1	1	1	0.840175727	0.014979196	0.838684561
Rv3237c	hypothetical protein	-0.1	-0.8	0.0	1	2	1	0.480762785	1.79E-10	0.83479282
Rv0202c	mmpL11	-0.4	-0.2	0.0	1	1	1	0.000601915	0.109178976	0.813269674
Rv0526	thioredoxin	-0.1	0.5	0.0	1	1	1	0.681781988	0.001360503	0.848293784
Rv3629c	integral membrane protein	-0.2	0.5	0.0	1	1	1	0.231635621	6.09E-05	0.813711052
Rv0191	MFS-type transporter	-0.4	0.0	0.0	1	1	1	0.002653358	0.996043064	0.804316943
Rv2640c	ArsR family transcriptional regulator	0.2	-0.8	0.0	1	2	1	0.195050448	1.10E-09	0.816497876
Rv3909	hypothetical protein	-0.6	1.1	0.0	2	2	1	0.000109449	1.28E-23	0.77660163
Rv0812	4-amino-4-deoxychorismate lyase	0.0	0.1	0.0	1	1	1	0.702064821	0.295815572	0.790288217
Rv0228	acyltransferase	-0.8	0.5	0.0	2	1	1	6.20E-10	0.000210197	0.762347493
Rv1208	gpgS	0.5	0.1	0.0	1	1	1	3.78E-05	0.641776091	0.769837171
Rv3807c	decaprenylphosphoryl-5-phosphoribo	0.1	0.6	0.0	1	2	1	0.51327683	1.49E-05	0.7899006
Rv3480c	diacyglycerol O-acyltransferase	0.3	-0.1	0.0	1	1	1	0.019521756	0.369996008	0.74517157
Rv2104a	hypothetical protein CDS	-0.1	-0.7	0.0	1	2	1	0.469479749	4.15E-05	0.816733478
Rv1336	cysM	0.0	-0.9	0.0	1	2	1	0.958833597	3.05E-17	0.752304821
Rv2994	MFS-type transporter	-0.3	0.1	0.0	1	1	1	0.013570765	0.315367473	0.762600395
Rv1202	dapE	0.3	0.6	0.0	1	1	1	0.013991775	1.25E-05	0.741260148
Rv1403c	methyltransferase	0.5	-0.6	0.0	1	2	1	0.007613286	0.000450955	0.811962101
Rv2390c	hypothetical protein	1.4	-0.2	0.0	3	1	1	1.36E-19	0.194711486	0.778917864
Rv0114	gmhB	1.1	0.3	0.0	2	1	1	5.73E-13	0.017623806	0.72451692
Rv2395	integral membrane protein	0.1	0.4	0.0	1	1	1	0.45462577	0.000247902	0.691258767
Rv1244	lpqZ	-0.2	0.4	0.0	1	1	1	0.272328948	0.011565082	0.726642618
Rv0015c	pknA	-0.2	0.6	0.0	1	1	1	0.15976987	4.48E-08	0.685850534
Rv3196A	hypothetical protein	0.3	0.6	0.0	1	2	1	0.068709633	0.001619655	0.750529806
Rv3030	S-adenosylmethionine-dependent me	-0.2	1.6	0.0	1	3	1	0.094777355	3.64E-28	0.728890406
Rv0780	purC	-0.9	0.7	0.0	2	2	1	2.96E-09	3.29E-06	0.702683416
Rv1326c	glgB	0.1	-1.5	0.0	1	3	1	0.52192875	9.85E-50	0.708032178
Rv1868	hypothetical protein	-0.5	0.4	0.0	1	1	1	0.000207471	0.000134642	0.654484533
Rv2763c	dfrA	0.4	-0.3	0.0	1	1	1	0.010917783	0.020623034	0.708696354
Rv0403c	mmpS1	-0.8	0.9	-0.1	2	2	1	5.10E-08	3.33E-07	0.692413881
Rv0103c	ctpB	0.4	0.1	-0.1	1	1	1	0.000263703	0.372758884	0.634443087
Rv0557	mgtA	-0.2	0.3	-0.1	1	1	1	0.074917707	0.034546734	0.646653659
Rv2388c	hemN	0.0	0.2	-0.1	1	1	1	0.738373685	0.035204995	0.655691577
Rv2932	ppsB	-0.8	1.6	-0.1	2	3	1	1.08E-10	4.77E-61	0.6236149
Rv1283c	oppB	-0.7	1.3	-0.1	2	2	1	2.18E-06	4.57E-23	0.629291418
Rv0505c	serB1	0.1	-0.3	-0.1	1	1	1	0.527112923	0.007773406	0.646816612
Rv1272c	drug ABC transporter ATP-binding prot	0.0	0.7	-0.1	1	2	1	0.800441188	1.09E-09	0.610968722
Rv0676c	mmpL5	-0.5	0.8	-0.1	1	2	1	9.09E-07	3.95E-17	0.652304109
Rv0560c	benzoquinone methyltransferase	-0.3	-0.7	-0.1	1	2	1	0.042341769	8.14E-07	0.689437649
Rv1918c	PPE35	-0.4	0.7	-0.1	1	2	1	0.000483051	1.90E-12	0.590452644

Rv2681	hypothetical protein	-0.4	0.9	-0.1	1	2	1	0.052696645	1.04E-13	0.608898299
Rv1610	membrane protein	0.8	0.3	-0.1	2	1	1	2.73E-10	0.031322033	0.647053606
Rv0473	transmembrane protein	0.3	-0.6	-0.1	1	2	1	0.026378288	1.71E-08	0.596633027
Rv0292	eccE3	-0.1	-1.9	-0.1	1	4	1	0.63061646	2.33E-70	0.592102663
Rv2609c	membrane protein	0.5	0.3	-0.1	1	1	1	0.00014551	0.008962714	0.597902144
Rv1729c	S-adenosylmethionine-dependent me	0.6	-0.1	-0.1	2	1	1	1.80E-06	0.681702754	0.599572576
Rv0681	transcriptional regulator	0.2	-0.1	-0.1	1	1	1	0.202741855	0.566906712	0.584489731
Rv2345	transmembrane protein	-0.1	-0.5	-0.1	1	1	1	0.346239954	2.95E-06	0.564420847
Rv2844	hypothetical protein	0.5	0.1	-0.1	1	1	1	0.001282612	0.718348558	0.629608068
Rv2139	pyrD	0.1	0.3	-0.1	1	1	1	0.710421721	0.033858673	0.56188025
Rv0397A	hypothetical protein	1.0	-1.0	-0.1	2	2	1	4.19E-13	1.02E-14	0.593325116
Rv1711	RNA pseudouridine synthase	0.5	1.4	-0.1	1	3	1	8.97E-05	4.48E-27	0.543371851
Rv1056	hypothetical protein	-0.4	-0.1	-0.1	1	1	1	0.02119396	0.478933889	0.554154287
Rv3395A	membrane protein	0.9	0.2	-0.1	2	1	1	4.87E-07	0.128992815	0.562331495
Rv1367c	hypothetical protein	0.2	-0.3	-0.1	1	1	1	0.247900442	0.02876208	0.526448564
Rv3814c	acyltransferase	0.7	-0.7	-0.1	2	2	1	1.87E-07	2.49E-09	0.56003184
Rv0890c	HTH-type transcriptional regulator	-0.5	1.1	-0.1	1	2	1	7.31E-05	1.55E-24	0.496417674
Rv2449c	trans-acting enoyl reductase	0.8	-0.6	-0.1	2	1	1	1.66E-05	3.48E-07	0.522898406
Rv2699c	hypothetical protein	0.2	0.3	-0.1	1	1	1	0.279171411	0.057471548	0.594171223
Rv1375	hypothetical protein	-0.4	-0.3	-0.1	1	1	1	0.001335484	0.035677355	0.498565808
Rv0480c	amidohydrolase	-0.8	0.3	-0.1	2	1	1	2.33E-07	0.041719266	0.568036484
Rv0580c	hypothetical protein	0.1	-1.3	-0.1	1	2	1	0.431285164	3.18E-21	0.53882904
Rv2613c	AP-4-A phosphorylase	0.5	0.4	-0.1	1	1	1	0.000722142	0.002173861	0.563637331
Rv3317	sdhD	0.6	0.4	-0.1	2	1	1	3.20E-05	0.005268514	0.512434949
Rv1390	rpoZ	0.3	-0.9	-0.1	1	2	1	0.005644676	3.96E-17	0.574431246
Rv1678	integral membrane protein	-0.3	0.4	-0.1	1	1	1	0.021080707	0.001651512	0.463011511
Rv1254	acyltransferase	-0.1	0.0	-0.1	1	1	1	0.638136863	0.873897496	0.411231349
Rv3305c	amiA1	0.5	-0.3	-0.1	1	1	1	0.000223561	0.006247852	0.428880267
Rv2008c	hypothetical protein	-0.3	-1.4	-0.1	1	3	1	0.042764768	4.55E-28	0.512551561
Rv3691	hypothetical protein	-0.2	0.3	-0.1	1	1	1	0.142612232	0.015655611	0.428691294
Rv1317c	alkA	0.0	0.2	-0.1	1	1	1	0.80256182	0.073694843	0.39639529
Rv0900	arfB	0.2	0.0	-0.1	1	1	1	0.175682026	0.916713434	0.533160098
Rv2045c	lipT	0.2	-0.6	-0.1	1	2	1	0.034492911	4.19E-07	0.390620175
Rv3385c	vapB46	-1.9	0.0	-0.1	4	1	1	1.50E-15	0.859910591	0.533526215
Rv2324	AsnC family transcriptional regulator	-0.3	-1.6	-0.1	1	3	1	0.083742978	1.91E-35	0.476444755
Rv3773c	hypothetical protein	0.3	0.0	-0.1	1	1	1	0.061842201	0.828348872	0.432513817
Rv2213	pepB	0.1	0.3	-0.1	1	1	1	0.626103086	0.009483656	0.403654583
Rv2284	lipM	0.1	0.2	-0.1	1	1	1	0.655221818	0.189069107	0.394668153
Rv3423c	alr	-0.5	0.5	-0.1	1	1	1	0.000155478	1.61E-06	0.372293442
Rv0744c	transcriptional regulator	-0.3	-1.1	-0.1	1	2	1	0.058730997	5.05E-20	0.403290134
Rv1412	ribC	-0.4	-0.2	-0.1	1	1	1	0.01153945	0.107102329	0.411018753
Rv3372	otsB2	-1.0	0.4	-0.1	2	1	1	5.95E-12	0.014098007	0.391673852
Rv3581c	ispF	-0.5	-0.9	-0.1	1	2	1	2.95E-05	9.78E-11	0.334094097
Rv1023	eno	0.3	0.5	-0.1	1	1	1	0.004283164	3.05E-05	0.34542224
Rv1785c	cyp143	0.6	-1.1	-0.1	2	2	1	4.67E-07	4.10E-20	0.346729696
Rv1589	bioB	0.2	1.1	-0.1	1	2	1	0.119293299	1.27E-19	0.353328477

Rv2206	transmembrane protein	-0.5	-0.3	-0.1	1	1	1	0.000356108	0.023649057	0.401943996
Rv0436c	pssA	-0.3	0.6	-0.1	1	2	1	0.01625718	2.17E-06	0.356883906
Rv1439c	hypothetical protein	-0.6	0.8	-0.1	1	2	1	9.32E-05	5.97E-08	0.384053219
Rv0362	mgtE	0.3	0.9	-0.1	1	2	1	0.006818125	4.36E-12	0.340265969
Rv0470A	hypothetical protein	0.3	-0.2	-0.1	1	1	1	0.032915723	0.164183388	0.402114818
Rv0127	mak	0.1	0.2	-0.1	1	1	1	0.432779548	0.13414503	0.319904777
Rv2841c	nusA	1.0	-0.2	-0.1	2	1	1	1.93E-15	0.060273103	0.335711769
Rv0486	mshA	0.2	-1.1	-0.1	1	2	1	0.147279409	8.42E-29	0.307008817
Rv0422c	thiD	0.5	0.0	-0.1	1	1	1	0.000460447	0.740411043	0.382049845
Rv0045c	hydrolase	0.0	-0.3	-0.1	1	1	1	0.801679276	0.006715238	0.366346147
Rv3365c	hypothetical protein	-0.4	1.0	-0.1	1	2	1	0.003862168	2.72E-16	0.305423092
Rv0080	hypothetical protein	1.8	-0.8	-0.1	4	2	1	1.27E-48	2.48E-10	0.357175766
Rv1421	hypothetical protein	0.4	-0.4	-0.1	1	1	1	0.000148719	0.000452397	0.293879923
Rv2152c	murC	0.0	0.7	-0.1	1	2	1	0.987273288	9.28E-09	0.274115399
Rv0939	bifunctional 2-hydroxyhepta-2,4-dien	0.1	-1.9	-0.1	1	4	1	0.303450336	1.47E-78	0.266417474
Rv1213	glgC	0.2	-1.1	-0.1	1	2	1	0.078924482	1.28E-19	0.294742423
Rv3828c	resolvase	1.2	0.2	-0.1	2	1	1	7.32E-14	0.032017219	0.259926122
Rv3256c	hypothetical protein	-0.3	-0.1	-0.1	1	1	1	0.035292895	0.380959902	0.303530878
Rv1595	nadB	0.2	-0.1	-0.1	1	1	1	0.055931894	0.25723361	0.254040749
Rv2393	che1	0.7	-0.2	-0.1	2	1	1	2.33E-09	0.152083257	0.238427738
Rv3775	lipE	0.1	0.5	-0.1	1	1	1	0.333050132	1.64E-05	0.356907343
Rv1122	gnd2	1.4	-0.3	-0.1	3	1	1	6.97E-27	0.01330751	0.255223527
Rv0336	hypothetical protein	0.5	-0.4	-0.1	1	1	1	0.015646242	0.013418656	0.400782949
Rv1694	tlyA	-0.6	0.9	-0.1	2	2	1	0.001788063	2.19E-11	0.30554885
Rv2100	hypothetical protein	0.3	0.0	-0.1	1	1	1	0.04590001	0.86376951	0.372719938
Rv2679	echA15	-0.2	-0.2	-0.1	1	1	1	0.434300229	0.068261166	0.315671169
Rv1022	lpqU	0.0	0.0	-0.1	1	1	1	0.875263047	0.789766599	0.296790443
Rv1234	transmembrane protein	-0.4	-0.5	-0.1	1	1	1	0.001799232	9.29E-05	0.261710742
Rv0250c	hypothetical protein	-0.3	-0.6	-0.1	1	2	1	0.052477852	2.60E-10	0.318616385
Rv0860	fadB	0.4	-0.8	-0.1	1	2	1	4.24E-05	4.99E-18	0.205526324
Rv2675c	hypothetical protein	0.6	-0.6	-0.1	2	2	1	2.80E-08	2.68E-07	0.225340746
Rv1885c	chorismate mutase	1.3	0.4	-0.1	2	1	1	2.86E-17	0.00978938	0.286197573
Rv2174	mptA	-0.2	0.2	-0.1	1	1	1	0.04696373	0.144416847	0.227472637
Rv0513	transmembrane protein	-0.3	1.1	-0.1	1	2	1	0.019375203	3.18E-18	0.223972134
Rv0515	hypothetical protein	0.5	-0.4	-0.1	1	1	1	0.016698909	0.015930073	0.372696522
Rv2955c	hypothetical protein	-1.0	1.5	-0.1	2	3	1	1.49E-09	1.43E-24	0.269348258
Rv3360	hypothetical protein	0.4	-0.5	-0.1	1	1	1	0.008620551	0.000238445	0.315749297
Rv3673c	membrane-anchored thioredoxin-like	-0.1	0.6	-0.1	1	1	1	0.352299564	2.73E-05	0.257400216
Rv3441c	mrsA	-0.3	0.5	-0.1	1	1	1	0.020515743	0.000125862	0.229958796
Rv2735c	hypothetical protein	0.7	1.1	-0.1	2	2	1	1.65E-08	1.35E-19	0.26119236
Rv0168	yrbE1B	0.0	0.5	-0.1	1	1	1	0.711708459	1.19E-05	0.198816241
Rv2095c	pafC	-0.2	-0.6	-0.1	1	1	1	0.206077429	6.95E-05	0.274038087
Rv3827c	transposase	0.9	-0.3	-0.1	2	1	1	2.42E-09	0.026014819	0.168608842
Rv1893	hypothetical protein	1.1	-1.1	-0.1	2	2	1	8.86E-12	2.36E-11	0.355482385
Rv2396	PE PGRS41	-0.4	-0.6	-0.1	1	2	1	0.005165316	4.95E-08	0.325519627
Rv3789	GtrA family protein	-0.3	0.8	-0.1	1	2	1	0.037666484	1.34E-07	0.236051056

Rv2357c	glyS	0.3	0.3	-0.1	1	1	1	0.007407678	0.007458506	0.186505213
Rv3261	fbiA	-0.2	0.7	-0.1	1	2	1	0.219985139	8.60E-09	0.207051615
Rv2338c	moeW	0.1	0.5	-0.1	1	1	1	0.319904444	8.71E-06	0.230902064
Rv1290c	hypothetical protein	-0.2	0.6	-0.2	1	2	1	0.094865974	3.33E-07	0.180481367
Rv2082	hypothetical protein	0.1	0.9	-0.2	1	2	1	0.517761381	2.33E-13	0.286188387
Rv0957	purH	0.3	0.7	-0.2	1	2	1	0.022150664	3.76E-08	0.184730902
Rv1640c	lysX	-0.2	-0.4	-0.2	1	1	1	0.036023054	0.000253434	0.145652461
Rv0750	hypothetical protein	0.0	-0.8	-0.2	1	2	1	0.737347439	1.02E-08	0.199043293
Rv2818c	CRISPR-associated protein Csm6	1.1	0.0	-0.2	2	1	1	6.01E-24	0.747468498	0.211943234
Rv3695	membrane protein	-0.2	-0.1	-0.2	1	1	1	0.056318176	0.460765658	0.173815931
Rv3597c	lsr2	-0.1	0.5	-0.2	1	1	1	0.43270446	5.28E-06	0.155810479
Rv1724c	hypothetical protein	0.4	-1.2	-0.2	1	2	1	0.007953106	2.94E-18	0.283257976
Rv3158	nuoN	0.1	0.7	-0.2	1	2	1	0.567386274	1.41E-12	0.152205955
Rv1025	hypothetical protein	-0.2	0.7	-0.2	1	2	1	0.209057107	3.81E-06	0.203376106
Rv3786c	hypothetical protein	-0.4	0.5	-0.2	1	1	1	0.00043583	3.02E-05	0.154846996
Rv2418c	hypothetical protein	0.3	0.2	-0.2	1	1	1	0.062980841	0.145938892	0.160850117
Rv0407	fgd1	-0.1	0.7	-0.2	1	2	1	0.231761219	5.01E-10	0.146473655
Rv3299c	atsB	-0.2	0.6	-0.2	1	2	1	0.141960679	3.66E-09	0.127540085
Rv0712	hypothetical protein	0.1	-1.3	-0.2	1	2	1	0.320744455	1.37E-29	0.148653774
Rv3483c	hypothetical protein	-0.1	0.5	-0.2	1	1	1	0.374865941	0.000151044	0.159683435
Rv0940c	oxidoreductase	0.5	-1.0	-0.2	1	2	1	7.54E-05	1.08E-19	0.13648496
Rv1454c	qor	-0.2	-0.3	-0.2	1	1	1	0.199482547	0.060873513	0.199595114
Rv1335	cysO	0.7	-1.0	-0.2	2	2	1	1.32E-07	2.13E-16	0.194247226
Rv3052c	nrdI	-0.3	-0.1	-0.2	1	1	1	0.02063394	0.373610644	0.164066981
Rv0247c	succinate dehydrogenase iron-sulfur s	1.1	1.4	-0.2	2	3	1	5.61E-15	1.11E-44	0.279324442
Rv0456B	mazE1	0.2	0.3	-0.2	1	1	1	0.229302103	0.213403413	0.331583217
Rv1245c	short-chain type dehydrogenase/redu	-0.4	0.3	-0.2	1	1	1	0.000941993	0.02441965	0.152528669
Rv3734c	tgs2	0.3	-0.8	-0.2	1	2	1	0.009310211	1.72E-12	0.110445242
Rv3757c	proW	0.1	-0.1	-0.2	1	1	1	0.495834212	0.477574696	0.14524588
Rv1835c	serine esterase	-0.1	0.7	-0.2	1	2	1	0.327788113	6.14E-10	0.108143564
Rv3888c	membrane protein	-0.1	0.5	-0.2	1	1	1	0.397975361	0.000106061	0.111191711
Rv0173	lprK	0.7	1.7	-0.2	2	3	1	2.30E-09	2.02E-44	0.133377157
Rv1238	sugC	0.2	-0.5	-0.2	1	1	1	0.219836596	1.21E-05	0.104645628
Rv0354c	PPE7	0.4	0.3	-0.2	1	1	1	0.009270159	0.00623749	0.093980671
Rv3876	espI	0.1	0.6	-0.2	1	2	1	0.542667747	2.94E-08	0.188932919
Rv3143	response regulator	0.9	0.5	-0.2	2	1	1	6.55E-09	0.001369646	0.181472405
Rv1709	scpA	0.1	1.6	-0.2	1	3	1	0.197388597	2.35E-47	0.100582777
Rv3825c	pks2	0.2	-1.5	-0.2	1	3	1	0.023385873	5.77E-41	0.069219989
Rv1896c	S-adenosyl-L-methionine-dependent r	0.8	-0.2	-0.2	2	1	1	9.59E-10	0.067151781	0.119343961
Rv0814c	hypothetical protein	1.1	-0.2	-0.2	2	1	1	2.98E-19	0.168997098	0.113356824
Rv1170	mshB	-0.3	-0.5	-0.2	1	1	1	0.019186262	5.69E-05	0.110514595
Rv0167	yrbE1A	-0.1	1.2	-0.2	1	2	1	0.261950203	1.19E-21	0.110340243
Rv3319	sdhB	0.7	0.7	-0.2	2	2	1	3.98E-07	1.74E-07	0.120734855
Rv1623c	cydA	0.9	-1.0	-0.2	2	2	1	8.14E-15	2.12E-16	0.127356511
Rv3493c	Mce associated protein	0.6	0.6	-0.2	2	2	1	1.56E-05	3.09E-05	0.142307588
Rv0514	transmembrane protein	0.0	0.7	-0.2	1	2	1	0.794186208	7.54E-06	0.139003648

Rv1079	metB	0.3	1.0	-0.2	1	2	1	0.01393082	2.47E-20	0.068696895
Rv2325c	hypothetical protein	0.8	-0.8	-0.2	2	2	1	4.75E-11	1.20E-13	0.081974389
Rv3612c	hypothetical protein	0.1	-0.6	-0.2	1	2	1	0.286221544	5.60E-08	0.099717846
Rv3739c	PPE67	-0.5	0.7	-0.2	1	2	1	0.000986813	3.09E-07	0.147824033
Rv1741	vapC34	0.4	-0.6	-0.2	1	2	1	0.014108224	0.001405596	0.201026648
Rv3634c	galE1	-0.2	1.1	-0.2	1	2	1	0.119208968	5.93E-16	0.104059724
Rv0171	mce1C	0.1	1.6	-0.2	1	3	1	0.459129751	7.50E-45	0.062407997
Rv1746	pknF	-0.3	0.7	-0.2	1	2	1	0.00553825	2.51E-10	0.067951136
Rv2981c	ddlA	0.2	0.6	-0.2	1	1	1	0.137906819	3.36E-06	0.072063647
Rv1041c	IS2-like transposase	0.2	-1.1	-0.2	1	2	1	0.118290956	2.01E-27	0.062317634
Rv0065	vapC1	1.5	-1.9	-0.2	3	4	1	3.39E-34	5.88E-64	0.128415987
Rv3067	hypothetical protein	-0.4	0.4	-0.2	1	1	1	0.003617087	0.010564379	0.09000316
Rv0789c	hypothetical protein	1.0	-1.1	-0.2	2	2	1	6.21E-16	1.55E-21	0.083447612
Rv1458c	antibiotic ABC transporter ATP-bindin	0.6	0.2	-0.2	2	1	1	0.010803015	0.140221285	0.067916754
Rv1357c	hypothetical protein	0.2	-0.1	-0.2	1	1	1	0.118386585	0.228611477	0.068333269
Rv0519c	membrane protein	-0.2	0.7	-0.2	1	2	1	0.270345277	6.01E-08	0.107495876
Rv1168c	PPE17	-1.4	-0.8	-0.2	3	2	1	2.74E-24	6.32E-10	0.082424699
Rv1776c	transcriptional regulator	-0.3	-0.3	-0.2	1	1	1	0.12658632	0.038006597	0.116329693
Rv3121	cyp141	0.8	0.9	-0.2	2	2	1	3.48E-10	5.67E-14	0.058904383
Rv1877	MFS-type transporter	0.5	0.2	-0.2	1	1	1	5.13E-06	0.046497449	0.060587159
Rv2888c	amiC	0.3	0.6	-0.2	1	1	1	0.019752149	3.58E-07	0.056049259
Rv0998	acetyltransferase Pat	0.1	-0.2	-0.2	1	1	1	0.360487178	0.156223744	0.06938436
Rv1470	trxA	0.8	-0.6	-0.2	2	2	1	3.61E-08	3.76E-05	0.108072886
Rv3100c	smpB	0.8	0.4	-0.2	2	1	1	2.55E-09	0.010101311	0.110445952
Rv3411c	guaB2	0.3	-0.2	-0.2	1	1	1	0.003801073	0.020034539	0.051948616
Rv2425c	hypothetical protein	0.4	0.8	-0.2	1	2	1	0.000797829	9.92E-11	0.070095846
Rv3859c	gltB	1.0	-0.6	-0.2	2	2	1	1.11E-21	3.27E-10	0.056967677
Rv3633	hypothetical protein	0.8	0.3	-0.2	2	1	1	2.85E-10	0.055032038	0.107652954
Rv2895c	viuB	0.3	-0.1	-0.2	1	1	1	0.012450821	0.590077262	0.069877914
Rv0067c	transcriptional regulator	0.0	0.5	-0.2	1	1	1	0.912603062	0.000277169	0.106594645
Rv1672c	integral membrane transport protein	-0.1	-0.7	-0.2	1	2	1	0.452999489	1.66E-08	0.085624759
Rv1842c	hypothetical protein	-0.2	0.6	-0.2	1	1	1	0.229623792	6.76E-07	0.050962206
Rv0478	deoC	0.6	-1.6	-0.2	2	3	1	4.49E-07	3.59E-44	0.07760268
Rv3434c	transmembrane protein	-0.2	0.9	-0.2	1	2	1	0.070878636	1.57E-12	0.067871299
Rv1917c	PPE34	-0.7	0.2	-0.2	2	1	1	0.000611079	0.145785906	0.131139635
Rv1446c	opcA	0.5	0.3	-0.2	1	1	1	0.039213406	0.007054064	0.053282191
Rv3159c	PPE53	-0.3	1.3	-0.2	1	2	1	0.046556076	2.57E-29	0.070618009
Rv2758c	vapB21	0.1	0.7	-0.2	1	2	1	0.631993683	0.000428257	0.146850657
Rv2700	hypothetical protein	0.3	0.1	-0.2	1	1	1	0.07127348	0.435100328	0.074572009
Rv1953	vapC14	-0.2	-2.3	-0.2	1	5	1	0.432204304	3.43E-43	0.113443187
Rv2900c	fdhF	0.3	-0.1	-0.2	1	1	1	0.00329611	0.249672434	0.036860402
Rv1078	hypothetical protein	0.5	1.0	-0.2	1	2	1	5.50E-06	2.25E-20	0.048473522
Rv2401	hypothetical protein	-0.1	0.5	-0.2	1	1	1	0.550698126	0.004028449	0.104878237
Rv2119	hypothetical protein	-0.2	0.8	-0.2	1	2	1	0.173456405	2.79E-07	0.062713365
Rv2770c	PPE44	0.0	0.0	-0.2	1	1	1	0.762858632	0.962177118	0.055800853
Rv3778c	aminotransferase	0.2	0.0	-0.2	1	1	1	0.070812021	0.717019127	0.052354

Rv2869c	rip	0.5	-0.6	-0.2	1	1	1	2.04E-05	5.78E-07	0.046198673
Rv2155c	murD	-0.3	0.7	-0.2	1	2	1	0.022467679	1.86E-09	0.041680755
Rv0945	oxidoreductase	0.0	0.2	-0.2	1	1	1	0.922077497	0.242543518	0.082545002
Rv3430a	hypothetical protein CDS	0.0	-0.4	-0.2	1	1	1	0.862958388	0.05598317	0.208593112
Rv1627c	nonspecific lipid-transfer protein	0.2	-0.3	-0.2	1	1	1	0.16659341	0.001083421	0.039251916
Rv1910c	hypothetical protein	0.4	-0.5	-0.2	1	1	1	0.004905508	0.000230854	0.05796753
Rv3795	embB	-0.1	1.4	-0.2	1	3	1	0.281218832	8.69E-26	0.027918108
Rv2312	hypothetical protein	0.2	-0.5	-0.2	1	1	1	0.33101655	0.006901397	0.083393484
Rv1159	pimE	0.7	-1.0	-0.2	2	2	1	5.96E-09	2.12E-24	0.028383784
Rv0740	hypothetical protein	0.1	-0.4	-0.2	1	1	1	0.471854958	0.002643465	0.049882727
Rv1816	HTH-type transcriptional regulator	-0.3	-0.4	-0.2	1	1	1	0.05959132	0.002927671	0.060391752
Rv1373	glycolipid sulfotransferase	0.0	-1.0	-0.2	1	2	1	0.760559678	2.00E-14	0.091860599
Rv0081	HTH-type transcriptional regulator	0.4	0.3	-0.2	1	1	1	0.006598775	0.083881444	0.103886278
Rv2788	sirR	-0.6	-0.3	-0.2	1	1	1	0.003455141	0.064382344	0.055973278
Rv3312c	hypothetical protein	0.0	0.2	-0.2	1	1	1	0.893763816	0.084396061	0.046149959
Rv3400	hydrolase	0.6	-0.3	-0.2	1	1	1	1.29E-06	0.010506615	0.038906811
Rv3134c	universal stress protein	0.0	0.3	-0.2	1	1	1	0.997607752	0.005973118	0.03956227
Rv1749c	integral membrane protein	0.1	-0.2	-0.2	1	1	1	0.567729428	0.099384504	0.054975699
Rv3455c	truA	0.1	0.8	-0.2	1	2	1	0.63467706	2.20E-10	0.027927681
Rv0345	hypothetical protein	0.1	0.1	-0.2	1	1	1	0.563547843	0.567329245	0.054254547
Rv1494	mazE4	0.0	-0.9	-0.2	1	2	1	0.984392373	6.06E-11	0.131190139
Rv1118c	hypothetical protein	-0.6	0.4	-0.2	2	1	1	4.58E-06	0.002175007	0.03975796
Rv0962c	lprP	0.4	-0.3	-0.2	1	1	1	0.02135389	0.007284519	0.035140957
Rv3118	hypothetical protein	1.0	-0.1	-0.2	2	1	1	1.40E-14	0.610829907	0.041858471
Rv0491	regX3	1.1	-1.6	-0.2	2	3	1	6.08E-08	7.32E-58	0.024495542
Rv1086	(2Z,6E)-farnesyl diphosphate synthas	-0.1	-0.8	-0.2	1	2	1	0.582875463	2.50E-12	0.039474453
Rv3623	lpqG	0.0	0.6	-0.2	1	2	1	0.892668602	1.77E-06	0.035567638
Rv0390	hypothetical protein	0.4	0.6	-0.2	1	1	1	0.02570235	0.001204123	0.096041503
Rv2421c	nadD	-0.6	0.4	-0.2	2	1	1	0.000193786	0.01001848	0.054388331
Rv3378c	diterpene synthase	0.1	0.5	-0.2	1	1	1	0.467162814	0.000849488	0.090097559
Rv0508	hypothetical protein	0.9	-0.4	-0.2	2	1	1	4.21E-12	0.00832219	0.071909909
Rv1690	lprJ	-0.1	1.3	-0.2	1	2	1	0.472517861	5.34E-11	0.060340255
Rv2921c	ftsY	0.2	-0.7	-0.3	1	2	1	0.076784641	1.44E-07	0.02855035
Rv3776	hypothetical protein	-0.2	0.3	-0.3	1	1	1	0.439368997	0.381303021	0.484938895
Rv1782	eccB5	0.9	-1.4	-0.3	2	3	1	9.94E-10	4.57E-42	0.021711443
Rv1131	prpC	-1.2	0.3	-0.3	2	1	1	4.76E-16	0.019814144	0.033032477
Rv2285	diacylglycerol acyltransferase	0.4	-0.4	-0.3	1	1	1	0.000188239	0.000558362	0.025451854
Rv2075c	hypothetical protein	-0.2	0.2	-0.3	1	1	1	0.0708255	0.10354704	0.024921053
Rv2031c	hspX	1.4	-0.9	-0.3	3	2	1	3.04E-16	5.26E-15	0.085467287
Rv2610c	pimA	0.7	0.4	-0.3	2	1	1	2.49E-08	0.000139844	0.029773285
Rv3276c	purK	-0.2	0.5	-0.3	1	1	1	0.103650192	6.37E-05	0.019636561
Rv0596c	vapB4	-1.0	0.4	-0.3	2	1	1	1.22E-05	0.062953082	0.124270553
Rv0468	fadB2	0.5	0.0	-0.3	1	1	1	8.07E-05	0.7129092	0.028350906
Rv3601c	panD	0.0	0.5	-0.3	1	1	1	0.845027504	0.003232561	0.054253972
Rv0626	vapB5	-1.4	0.1	-0.3	3	1	1	8.84E-18	0.452394871	0.051451746
Rv1420	uvrC	0.8	-0.6	-0.3	2	2	1	2.49E-13	3.43E-08	0.013169401

Rv1341	non-canonical purine NTP pyrophospl	0.5	0.0	-0.3	1	1	1	0.000117692	0.994116228	0.037008462
Rv0051	transmembrane protein	0.6	0.1	-0.3	1	1	1	8.63E-07	0.624422762	0.017282155
Rv2297	hypothetical protein	0.5	-0.2	-0.3	1	1	1	6.11E-05	0.089975667	0.027506448
Rv0529	ccsA	0.3	0.5	-0.3	1	1	1	0.040925141	5.17E-06	0.015739252
Rv0427c	xthA	0.5	0.2	-0.3	1	1	1	0.000242166	0.165990667	0.022651607
Rv1116	hypothetical protein	0.6	1.1	-0.3	1	2	1	0.001469024	3.01E-06	0.098468756
Rv3708c	asd	0.2	0.0	-0.3	1	1	1	0.111701338	0.857422458	0.020134849
Rv0248c	succinate dehydrogenase flavoprotei	0.4	1.3	-0.3	1	3	1	0.000110583	7.64E-47	0.028105653
Rv1702c	hypothetical protein	0.3	0.8	-0.3	1	2	1	0.07395783	2.89E-10	0.061647483
Rv3104c	transmembrane protein	0.4	-0.3	-0.3	1	1	1	0.000431916	0.03237093	0.013056107
Rv1459c	alpha-(1->6)-mannopyranosyltransfer	0.1	0.2	-0.3	1	1	1	0.649092475	0.020529333	0.010461698
Rv0205	transmembrane protein	0.2	0.9	-0.3	1	2	1	0.130887051	3.34E-13	0.01030205
Rv2293c	hypothetical protein	-0.1	0.5	-0.3	1	1	1	0.472084649	7.98E-05	0.01578577
Rv2021c	transcriptional regulator	0.0	-0.2	-0.3	1	1	1	0.939378107	0.19091238	0.026226484
Rv2782c	pepR	0.1	0.4	-0.3	1	1	1	0.473815407	0.000650846	0.00942033
Rv3306c	amiB1	-0.2	0.4	-0.3	1	1	1	0.115535656	0.001819901	0.009659442
Rv0036c	hypothetical protein	-0.1	0.6	-0.3	1	2	1	0.532636544	1.53E-06	0.013243427
Rv2592c	ruvB	-0.3	1.2	-0.3	1	2	1	0.136536856	1.33E-17	0.015223931
Rv0613c	hypothetical protein	0.0	0.9	-0.3	1	2	1	0.734863416	6.66E-16	0.008031542
Rv2982c	gpdA2	-0.8	1.0	-0.3	2	2	1	1.17E-09	2.45E-14	0.014600359
Rv0862c	hypothetical protein	-0.1	-0.4	-0.3	1	1	1	0.700397648	0.000285895	0.016011545
Rv0518	hypothetical protein	-0.3	0.8	-0.3	1	2	1	0.026180742	2.89E-09	0.010185089
Rv1231c	membrane protein	0.0	0.2	-0.3	1	1	1	0.904961433	0.060035675	0.011427459
Rv2453c	mobA	0.8	-1.0	-0.3	2	2	1	3.05E-10	2.22E-13	0.016786046
Rv0076c	membrane protein	0.2	0.9	-0.3	1	2	1	0.092401901	7.18E-09	0.024563115
Rv3225c	GCN5-like N-acetyltransferase	0.7	-0.1	-0.3	2	1	1	8.09E-08	0.519066643	0.005324055
Rv0328	transcriptional regulator	-1.1	-0.3	-0.3	2	1	1	1.62E-16	0.005234808	0.011617067
Rv3235	hypothetical protein	-0.3	0.1	-0.3	1	1	1	0.044573529	0.287656153	0.013223229
Rv3777	oxidoreductase	-0.1	0.0	-0.3	1	1	1	0.53555922	0.80487585	0.00879176
Rv0775	hypothetical protein	0.2	-0.7	-0.3	1	2	1	0.082708511	3.72E-09	0.010729505
Rv1333	hydrolase	0.3	-1.2	-0.3	1	2	1	0.035793288	7.15E-22	0.009056358
Rv0027	hypothetical protein	1.1	-1.1	-0.3	2	2	1	1.59E-12	5.97E-15	0.02934631
Rv1083	hypothetical protein	-0.7	-1.4	-0.3	2	3	1	0.000131311	2.22E-20	0.030206536
Rv1176c	hypothetical protein	-0.7	0.8	-0.3	2	2	1	3.88E-06	3.02E-06	0.018894222
Rv2619c	hypothetical protein	0.4	0.1	-0.3	1	1	1	0.012517101	0.529230307	0.033683413
Rv3495c	lprN	0.4	0.8	-0.3	1	2	1	0.000398657	1.35E-10	0.005049502
Rv2840c	hypothetical protein	1.1	-0.2	-0.3	2	1	1	2.30E-13	0.021727045	0.016885951
Rv0312	hypothetical protein	-0.5	0.1	-0.3	1	1	1	2.26E-05	0.597881119	0.002495187
Rv1248c	multifunctional 2-oxoglutarate dehydı	0.5	0.0	-0.3	1	1	1	1.42E-06	0.811233609	0.002188691
Rv0201c	hypothetical protein	0.2	0.2	-0.3	1	1	1	0.15214853	0.242913881	0.008574767
Rv2604c	snoP	0.6	0.7	-0.3	1	2	1	2.52E-05	1.50E-06	0.008585543
Rv1645c	hypothetical protein	-0.2	0.0	-0.3	1	1	1	0.134182314	0.844356656	0.006269536
Rv0623	vapB30	-2.1	-0.5	-0.3	4	1	1	1.83E-15	0.001791066	0.009549764
Rv0670	end	0.4	-2.4	-0.3	1	5	1	0.000160795	2.46E-118	0.00331947
Rv3910	peptidoglycan biosynthesis protein	-0.5	1.1	-0.3	1	2	1	0.00049716	6.64E-25	0.002098265
Rv3389c	htdY	0.0	1.1	-0.3	1	2	1	0.863913033	4.25E-16	0.011371936

Rv1060	hypothetical protein	0.4	-0.3	-0.3	1	1	1	0.009073614	0.016489567	0.008037093
Rv3479	transmembrane protein	0.1	0.6	-0.3	1	2	1	0.261855942	5.93E-10	0.001163633
Rv1607	chaA	0.6	0.4	-0.3	1	1	1	1.39E-06	0.000512816	0.004347509
Rv1594	nadA	0.5	0.1	-0.3	1	1	1	0.00118259	0.501506128	0.002524025
Rv3132c	devS	0.8	-0.1	-0.3	2	1	1	9.39E-13	0.23365707	0.001395038
Rv1106c	3 beta-hydroxysteroid dehydrogenase	-0.1	1.0	-0.3	1	2	1	0.547879154	3.28E-15	0.002937458
Rv3628	ppa	-0.1	0.7	-0.3	1	2	1	0.387501355	2.78E-06	0.005747752
Rv1546	hypothetical protein	-0.3	0.8	-0.3	1	2	1	0.015620844	7.85E-09	0.02983155
Rv0286	PPE4	-0.1	0.7	-0.3	1	2	1	0.298372159	1.32E-11	0.001735371
Rv1633	uvrB	0.4	-0.7	-0.3	1	2	1	0.002401874	2.06E-12	0.00155034
Rv2417c	DegV domain-containing protein	-0.3	0.3	-0.3	1	1	1	0.013897571	0.005638595	0.002405294
Rv2387	hypothetical protein	0.2	0.5	-0.3	1	1	1	0.029303232	5.43E-05	0.00189033
Rv0333	hypothetical protein	-0.4	0.7	-0.3	1	2	1	0.032782554	1.92E-05	0.01179075
Rv1531	hypothetical protein	0.7	0.1	-0.3	2	1	1	1.75E-08	0.354546429	0.006221375
Rv0102	integral membrane protein	0.3	-0.1	-0.3	1	1	1	0.00964632	0.398593972	0.001530661
Rv0921	resolvase	0.0	-0.2	-0.3	1	1	1	0.742015644	0.090065243	0.002604008
Rv2931	ppsA	-0.5	1.4	-0.3	1	3	1	8.33E-07	5.01E-45	0.001470547
Rv1839c	vapB13	-0.4	-0.1	-0.4	1	1	1	0.047980075	0.696770814	0.027773551
Rv3521	hypothetical protein	-0.1	0.2	-0.4	1	1	1	0.49873411	0.135678454	0.003613448
Rv1743	pknE	0.5	0.3	-0.4	1	1	1	2.90E-05	0.017467683	0.001101218
Rv2580c	hisS	0.8	-0.4	-0.4	2	1	1	5.65E-11	3.46E-05	0.002733524
Rv1775	hypothetical protein	-0.1	0.1	-0.4	1	1	1	0.451641352	0.246499277	0.003115603
Rv2975a	50S ribosomal protein L28 CDS	0.3	0.6	-0.4	1	2	1	0.123054906	0.000506077	0.015633583
Rv3329	aminotransferase	-0.1	0.8	-0.4	1	2	1	0.391695113	1.40E-09	0.001843427
Rv3435c	transmembrane protein	0.1	0.8	-0.4	1	2	1	0.703087529	4.53E-10	0.002959206
Rv2965c	kdtB	-0.5	0.9	-0.4	1	2	1	0.001378059	2.11E-11	0.007476529
Rv3405c	HTH-type transcriptional regulator	-0.2	0.3	-0.4	1	1	1	0.213864855	0.042023537	0.011189999
Rv1752	hypothetical protein	-0.4	1.3	-0.4	1	2	1	0.044453919	7.17E-14	0.006919319
Rv1498c	methyltransferase	0.5	0.5	-0.4	1	1	1	0.000275637	0.000960468	0.005517109
Rv1033c	trcR	-0.9	1.1	-0.4	2	2	1	1.92E-08	3.46E-15	0.002144732
Rv0955	integral membrane protein	-0.4	0.1	-0.4	1	1	1	0.001068419	0.244363543	0.001200153
Rv2440c	obg	0.5	0.9	-0.4	1	2	1	1.29E-06	2.58E-17	0.002160549
Rv2227	hypothetical protein	0.5	0.2	-0.4	1	1	1	0.000610007	0.089165823	0.005080365
Rv2736c	recX	0.6	1.0	-0.4	2	2	1	5.02E-06	9.52E-15	0.003080595
Rv2392	cysH	1.2	-0.3	-0.4	2	1	1	8.11E-24	0.000936518	0.005681397
Rv0680c	transmembrane protein	-0.3	0.0	-0.4	1	1	1	0.037154807	0.721845445	0.003509373
Rv3232c	ppk2	0.4	0.4	-0.4	1	1	1	0.005931082	0.003016637	0.00164834
Rv1635c	mannosyltransferase	-0.2	-1.0	-0.4	1	2	1	0.245478839	1.16E-21	0.000848143
Rv3244c	lpqB	-0.4	0.0	-0.4	1	1	1	0.010819479	0.876701322	0.001432003
Rv3916c	hypothetical protein	0.3	-1.3	-0.4	1	2	1	0.009092564	6.85E-36	0.001660739
Rv1400c	lipI	-0.9	0.8	-0.4	2	2	1	2.90E-06	1.26E-08	0.00288923
Rv1327c	glgE	0.5	-1.0	-0.4	1	2	1	4.66E-05	3.14E-23	0.000569837
Rv2944	insertion sequence element IS1533 tr	-0.1	1.4	-0.4	1	3	1	0.492994558	1.04E-21	0.002992261
Rv2871	vapB43	-1.8	-0.2	-0.4	4	1	1	4.10E-15	0.275867575	0.01415174
Rv0811c	hypothetical protein	-0.5	-0.9	-0.4	1	2	1	9.35E-05	3.45E-19	0.00093146
Rv1252c	lprE	0.1	0.9	-0.4	1	2	1	0.48601128	4.49E-09	0.005072454

Rv0511	hemD	-0.7	1.0	-0.4	2	2	1	1.57E-05	5.12E-23	0.001277356
Rv2450c	rpfE	-0.9	1.4	-0.4	2	3	1	2.57E-06	2.39E-20	0.009691681
Rv2454c	2-oxoglutarate oxidoreductase subun	0.5	-0.4	-0.4	1	1	1	0.000315032	2.04E-05	0.00077046
Rv2567	hypothetical protein	-0.3	0.9	-0.4	1	2	1	0.012981938	1.43E-15	0.000644423
Rv1564c	treX	-0.1	0.9	-0.4	1	2	1	0.527375472	7.46E-18	0.000995192
Rv2459	MFS-type transporter	0.5	-0.1	-0.4	1	1	1	5.78E-07	0.408273132	0.000387679
Rv2702	ppgK	-0.6	0.6	-0.4	2	1	1	6.12E-06	3.38E-06	0.001114584
Rv0698	hypothetical protein	-0.7	0.9	-0.4	2	2	1	6.62E-09	1.45E-13	0.00447403
Rv0774c	hypothetical protein	-0.6	0.4	-0.4	2	1	1	0.000145284	0.002321897	0.000768578
Rv1424c	membrane protein	0.4	-0.3	-0.4	1	1	1	0.004707681	0.019661066	0.006131111
Rv2219A	membrane protein	0.3	0.2	-0.4	1	1	1	0.013940326	0.215570915	0.002493843
Rv3812	PE PGRS62	-0.2	0.3	-0.4	1	1	1	0.11911869	0.014939479	0.000365577
Rv3025c	iscS	0.2	-0.2	-0.4	1	1	1	0.041586147	0.029763913	0.000388292
Rv1874	hypothetical protein	-0.5	0.4	-0.4	1	1	1	2.78E-05	0.001884824	0.005266148
Rv2532c	hypothetical protein	0.7	0.3	-0.4	2	1	1	1.01E-07	0.054709094	0.002342399
Rv3669	transmembrane protein	0.2	0.7	-0.4	1	2	1	0.173798316	4.16E-08	0.00100708
Rv1710	scpB	0.3	1.1	-0.4	1	2	1	0.017162802	1.37E-19	0.000969267
Rv3199c	nudC	0.3	0.3	-0.4	1	1	1	0.010339764	0.005866638	0.000814859
Rv1728c	hypothetical protein	0.5	0.4	-0.4	1	1	1	0.000438538	0.001053474	0.002117204
Rv0827c	kmtR	-0.9	-0.6	-0.4	2	1	1	5.91E-05	8.85E-05	0.002953758
Rv2019	hypothetical protein	0.0	0.2	-0.4	1	1	1	0.889009518	0.186539619	0.014556675
Rv0897c	oxidoreductase	0.1	-0.9	-0.4	1	2	1	0.443729956	2.59E-17	0.000293191
Rv2456c	MFS-type transporter	0.0	0.6	-0.4	1	1	1	0.971155184	8.88E-07	0.000576049
Rv0530	hypothetical protein	-0.6	-0.1	-0.4	2	1	1	2.07E-07	0.196859658	0.000773675
Rv2518c	ldtB	-0.3	-0.2	-0.4	1	1	1	0.00598287	0.034880315	0.003139977
Rv2907c	rimM	-0.2	1.0	-0.4	1	2	1	0.287918303	9.83E-11	0.005401869
Rv2399c	cysT	0.7	0.2	-0.4	2	1	1	6.24E-10	0.024229963	0.00026243
Rv1943c	mazE5	-0.2	0.5	-0.4	1	1	1	0.256063946	0.002981974	0.003958703
Rv0172	mce1D	0.1	1.6	-0.4	1	3	1	0.39642147	3.40E-52	0.000168769
Rv0865	mog	0.5	-1.0	-0.4	1	2	1	0.001126057	3.27E-17	0.000637647
Rv0914c	lipid carrier protein or keto acyl-CoA tl	-0.3	-0.2	-0.4	1	1	1	0.010437151	0.150411585	0.000342131
Rv3819	hypothetical protein	0.9	0.2	-0.4	2	1	1	2.67E-10	0.29956433	0.003186303
Rv0725c	hypothetical protein	1.0	-1.3	-0.4	2	2	1	6.43E-18	4.06E-34	0.000560636
Rv1001	arcA	-0.4	-1.4	-0.4	1	3	1	0.004213052	1.18E-40	0.000754629
Rv2102	hypothetical protein	0.1	0.6	-0.4	1	1	1	0.256730588	3.11E-06	0.000383212
Rv0074	hypothetical protein	0.4	1.5	-0.4	1	3	1	0.006824358	1.96E-34	0.000373477
Rv2794c	pptT	0.4	0.4	-0.4	1	1	1	0.015366122	0.005124089	0.000609504
Rv1249c	membrane protein	-0.6	0.1	-0.4	2	1	1	0.005721267	0.361555183	0.001212851
Rv3036c	TB22.2	0.1	0.2	-0.4	1	1	1	0.393652518	0.057790994	0.000220968
Rv3125c	PPE49	-0.1	0.8	-0.4	1	2	1	0.580219992	1.20E-09	0.002062771
Rv2663	hypothetical protein	-0.1	-0.5	-0.4	1	1	1	0.452643425	0.00056714	0.002222202
Rv1665	pks11	-0.5	0.8	-0.4	1	2	1	0.000136544	3.98E-11	0.000289294
Rv2362c	recO	-0.6	0.2	-0.4	1	1	1	3.81E-06	0.114999264	0.000292185
Rv1522c	mmpL12	-0.1	0.9	-0.4	1	2	1	0.325079663	4.97E-19	0.000139108
Rv2527	vapC17	0.9	-0.4	-0.4	2	1	1	8.26E-11	0.004095569	0.001795835
Rv1445c	devB	-0.5	0.8	-0.4	1	2	1	0.001915849	3.17E-09	0.000855982

Rv0126	treS	0.4	0.5	-0.4	1	1	1	0.000840321	5.07E-06	0.000329406
Rv1770	hypothetical protein	0.1	0.2	-0.4	1	1	1	0.511331355	0.134974592	0.000258112
Rv0189c	ilvD	-0.3	0.3	-0.4	1	1	1	0.008633898	0.002679357	9.30E-05
Rv2314c	hypothetical protein	0.3	-0.6	-0.4	1	2	1	0.065169645	3.64E-09	0.000181238
Rv0542c	menE	-0.2	0.6	-0.4	1	2	1	0.206782716	3.83E-06	0.000437242
Rv2101	helZ	-0.4	1.1	-0.4	1	2	1	0.003972332	1.74E-20	6.46E-05
Rv0222	echA1	0.0	1.5	-0.4	1	3	1	0.886304374	3.05E-28	0.000280819
Rv1127c	ppdK	0.0	-0.2	-0.4	1	1	1	0.704439673	0.095554669	0.000129555
Rv1900c	lipJ	-0.4	0.1	-0.4	1	1	1	0.018596701	0.57165665	0.000113675
Rv0525	hypothetical protein	0.2	0.3	-0.4	1	1	1	0.164317552	0.0441382	0.000881118
Rv3197A	whiB7	-0.1	-1.0	-0.4	1	2	1	0.627325275	6.35E-11	0.00369702
Rv2516c	hypothetical protein	-0.1	-0.4	-0.4	1	1	1	0.589917384	0.000167961	0.000108685
Rv3803c	fbpD	0.2	0.1	-0.4	1	1	1	0.043126126	0.376664403	0.000339904
Rv3604c	transmembrane protein	-0.3	0.4	-0.4	1	1	1	0.012592265	0.001028888	8.23E-05
Rv0913c	dioxygenase	0.4	0.2	-0.4	1	1	1	0.00517557	0.051347006	8.14E-05
Rv0420c	transmembrane protein	0.6	-0.3	-0.4	1	1	1	6.95E-06	0.020117497	0.000249432
Rv1684	hypothetical protein	0.6	-0.9	-0.4	2	2	1	1.78E-05	6.89E-10	0.001826911
Rv3105c	prfB	0.2	-0.1	-0.4	1	1	1	0.039496939	0.182101956	7.03E-05
Rv2630	hypothetical protein	0.8	-1.0	-0.4	2	2	1	2.02E-11	4.42E-17	0.003700991
Rv2016	hypothetical protein	-0.6	-0.4	-0.4	2	1	1	4.60E-06	0.00017183	0.000738117
Rv0166	fadD5	-0.2	1.8	-0.4	1	3	1	0.215007219	5.08E-51	4.33E-05
Rv1153c	omt	0.2	-1.4	-0.4	1	3	1	0.197010403	7.27E-40	0.000150057
Rv0241c	htdX	0.3	1.2	-0.4	1	2	1	0.002612808	2.85E-27	7.82E-05
Rv1689	tyrS	0.2	1.4	-0.4	1	3	1	0.236484697	7.70E-18	0.000126688
Rv3211	rhlE	0.5	0.6	-0.4	1	2	1	2.31E-05	1.99E-10	1.92E-05
Rv0589	mce2A	0.0	0.4	-0.4	1	1	1	0.995553014	0.00090186	0.000117865
Rv1205	hypothetical protein	-0.4	0.7	-0.4	1	2	1	0.000703876	3.68E-08	9.85E-05
Rv3436c	glmS	-0.1	0.6	-0.4	1	2	1	0.468461296	1.12E-07	6.60E-05
Rv2335	cysE	-0.2	1.1	-0.4	1	2	1	0.312434005	2.81E-10	0.000579212
Rv2298	oxidoreductase	-0.1	0.5	-0.4	1	1	1	0.357612882	2.78E-05	0.000133346
Rv1246c	relE	0.0	0.8	-0.4	1	2	1	0.778422768	1.04E-07	0.000958838
Rv3576	lppH	-0.7	1.4	-0.4	2	3	1	1.51E-07	2.38E-29	0.000191004
Rv1825	hypothetical protein	0.3	1.1	-0.4	1	2	1	0.045410361	1.52E-18	0.000205339
Rv0696	mycofactocin biosynthesis glycosyltra	-0.3	1.1	-0.4	1	2	1	0.169817	3.57E-23	4.60E-05
Rv3482c	membrane protein	0.1	0.9	-0.4	1	2	1	0.441541099	1.91E-14	9.46E-05
Rv3858c	gltD	1.4	-0.4	-0.4	3	1	1	4.24E-39	0.000138717	3.45E-05
Rv3690	membrane protein	-0.6	0.6	-0.4	1	2	1	5.57E-05	2.84E-06	0.000239991
Rv1941	short-chain type dehydrogenase/redu	0.1	0.4	-0.4	1	1	1	0.562138885	0.000129202	0.000121607
Rv3234c	tgs3	0.0	0.7	-0.5	1	2	1	0.804831723	5.09E-08	0.000136904
Rv3059	cyp136	0.3	-0.2	-0.5	1	1	1	0.00288501	0.064355111	0.000107774
Rv1542c	glbN	0.5	0.0	-0.5	1	1	1	0.002779781	0.995323342	0.000634901
Rv1731	gabD2	0.4	-0.5	-0.5	1	1	1	0.000135772	9.14E-06	5.39E-05
Rv2544	lppB	-0.1	0.0	-0.5	1	1	1	0.417947377	0.952916302	0.000250252
Rv2444c	rne	-0.1	0.0	-0.5	1	1	1	0.214736095	0.683677465	1.21E-05
Rv1294	thrA	0.2	0.4	-0.5	1	1	1	0.217393397	0.00024505	2.07E-05
Rv1777	cyp144	-0.5	0.8	-0.5	1	2	1	2.34E-05	1.29E-10	4.97E-05

Rv3291c	lrpA	0.8	-0.8	-0.5	2	2	1	1.31E-09	5.75E-08	0.000521857
Rv2596	vapC40	-0.7	0.0	-0.5	2	1	1	1.09E-06	0.901934376	0.000292617
Rv3610c	ftsH	0.3	-0.1	-0.5	1	1	1	0.03068864	0.399095797	5.98E-05
Rv0504c	hypothetical protein	0.5	-0.3	-0.5	1	1	1	0.003260827	0.007451851	0.000175553
Rv0813c	hypothetical protein	1.0	-0.5	-0.5	2	1	1	9.59E-14	0.000222665	0.000345748
Rv3420c	rimI	0.1	-0.9	-0.5	1	2	1	0.493138335	6.32E-12	4.19E-05
Rv1362c	membrane protein	-0.1	0.0	-0.5	1	1	1	0.400077883	0.870191032	0.000113187
Rv2437	transmembrane protein	1.0	-0.8	-0.5	2	2	1	6.69E-11	9.81E-11	0.000315446
Rv1295	thrC	0.7	0.1	-0.5	2	1	1	6.77E-10	0.20391905	5.24E-05
Rv2084	hypothetical protein	-0.1	1.0	-0.5	1	2	1	0.662044702	1.09E-15	6.41E-05
Rv3064c	integral membrane protein	-0.1	0.6	-0.5	1	1	1	0.577695542	0.000189874	0.000360985
Rv2906c	trmD	0.0	0.4	-0.5	1	1	1	0.827350518	0.001048614	8.68E-05
Rv0510	hemC	0.6	1.1	-0.5	2	2	1	6.06E-07	4.65E-21	1.28E-05
Rv3806c	ubiA	0.5	0.9	-0.5	1	2	1	1.68E-05	8.91E-15	0.000117618
Rv0871	cspB	0.0	0.8	-0.5	1	2	1	0.945554277	3.22E-06	0.000800745
Rv3802c	membrane protein	0.9	-0.9	-0.5	2	2	1	7.23E-17	4.20E-20	8.04E-05
Rv3884c	eccA2	0.7	0.8	-0.5	2	2	1	7.66E-09	1.26E-14	1.68E-05
Rv2979c	resolvase	0.8	0.1	-0.5	2	1	1	6.23E-11	0.596187628	0.000108741
Rv2860c	glnA4	-0.1	0.7	-0.5	1	2	1	0.460041295	1.33E-09	4.16E-05
Rv0512	hemB	-0.1	1.2	-0.5	1	2	1	0.626563919	1.55E-26	2.23E-05
Rv0402c	mmpL1	-0.3	0.9	-0.5	1	2	1	0.012345306	7.03E-21	6.30E-06
Rv2405	hypothetical protein	-1.1	-0.6	-0.5	2	2	1	4.32E-13	7.91E-06	9.44E-05
Rv0018c	pstP	-0.3	0.8	-0.5	1	2	1	0.034208102	2.26E-14	0.000250306
Rv1593c	hypothetical protein	0.7	-0.5	-0.5	2	1	1	2.05E-10	6.50E-06	7.34E-05
Rv1512	epiA	0.2	0.9	-0.5	1	2	1	0.149970476	3.31E-11	6.37E-05
Rv0609A	hypothetical protein	1.0	0.2	-0.5	2	1	1	2.12E-13	0.080384941	0.000125442
Rv1337	integral membrane protein	0.2	-0.8	-0.5	1	2	1	0.055779552	6.02E-13	1.25E-05
Rv1789	PPE26	-0.2	0.3	-0.5	1	1	1	0.048440445	0.004311061	8.61E-05
Rv3046c	hypothetical protein	0.6	0.0	-0.5	2	1	1	0.001835489	0.760451773	0.000256991
Rv2526	vapB17	-0.9	-0.6	-0.5	2	2	1	1.16E-05	0.000148763	0.000801798
Rv0419	lpqM	0.2	0.0	-0.5	1	1	1	0.150920894	0.950269054	6.78E-06
Rv1301	threonylcarbamoyl-AMP synthase	0.6	0.1	-0.5	1	1	1	9.34E-07	0.345455324	5.17E-05
Rv0169	mce1A	-0.1	1.4	-0.5	1	3	1	0.395628793	6.30E-37	7.66E-06
Rv3908	mutT4	-0.2	0.5	-0.5	1	1	1	0.207089161	5.57E-05	2.86E-05
Rv2181	alpha-(1-2)-phosphatidylinositol man	0.0	-0.3	-0.5	1	1	1	0.790002364	0.038390534	7.76E-06
Rv2173	idsA2	-0.8	0.3	-0.5	2	1	1	9.30E-09	0.033926775	2.01E-05
Rv1368	lprF	-0.2	1.2	-0.5	1	2	1	0.071271699	2.11E-19	2.20E-05
Rv3253c	cationic amino acid transport integral	-0.3	0.3	-0.5	1	1	1	0.007545316	0.001610491	1.59E-05
Rv2865	relF	-0.6	-0.3	-0.5	1	1	1	0.000352733	0.021354477	0.000300407
Rv2203	membrane protein	0.1	0.0	-0.5	1	1	1	0.398893276	0.650258826	2.45E-05
Rv0004	hypothetical protein	0.7	0.0	-0.5	2	1	1	9.48E-07	0.786685998	3.11E-05
Rv2493	vapB38	-0.1	-1.1	-0.5	1	2	1	0.664549975	7.69E-20	0.000309506
Rv0777	purB	0.2	0.2	-0.5	1	1	1	0.194788261	0.036176872	5.37E-06
Rv1860	apa	-1.0	1.5	-0.5	2	3	1	1.38E-13	1.98E-29	0.000282302
Rv0587	yrbE2A	-0.5	0.2	-0.5	1	1	1	9.10E-05	0.108365046	8.55E-06
Rv0043c	HTH-type transcriptional regulator	0.9	-0.1	-0.5	2	1	1	1.62E-15	0.347233393	1.11E-05

Rv0922	transposase	0.4	-0.2	-0.5	1	1	1	0.00063199	0.045389225	4.48E-06
Rv0758	phoR	0.3	-0.8	-0.5	1	2	1	0.027709818	9.65E-14	1.07E-05
Rv2657c	prophage protein	-0.4	-1.7	-0.5	1	3	1	0.012877745	7.74E-31	0.000741566
Rv0588	hypothetical protein	0.0	0.1	-0.5	1	1	1	0.995792247	0.317315013	6.41E-06
Rv2682c	dxs1	-0.4	0.9	-0.5	1	2	1	0.000461244	3.67E-17	2.73E-05
Rv0726c	S-adenosylmethionine-dependent me	0.4	-0.1	-0.5	1	1	1	0.001585498	0.423245194	2.51E-05
Rv0778	cyp126	-0.3	-0.3	-0.5	1	1	1	0.026824014	0.007142579	1.32E-05
Rv3805c	aftB	0.2	0.8	-0.5	1	2	1	0.189738244	4.75E-16	9.01E-06
Rv0012	membrane protein	0.3	-0.4	-0.5	1	1	1	0.009671906	0.001637528	3.27E-05
Rv3263	DNA methylase	0.3	0.5	-0.5	1	1	1	0.023238274	6.09E-05	1.20E-05
Rv1534	transcriptional regulator	-0.2	0.7	-0.5	1	2	1	0.176405649	1.69E-06	5.18E-05
Rv2242	hypothetical protein	0.0	0.3	-0.5	1	1	1	0.923293363	0.005137307	8.68E-06
Rv3137	histidinol-phosphatase	-0.7	0.9	-0.5	2	2	1	1.32E-08	1.13E-13	1.02E-05
Rv2467	pepN	0.5	0.5	-0.5	1	1	1	3.72E-05	1.07E-06	2.88E-06
Rv2184c	hypothetical protein	0.3	0.0	-0.5	1	1	1	0.032163569	0.782279842	1.41E-05
Rv3631	transferase	-0.1	1.2	-0.5	1	2	1	0.38690309	4.85E-15	4.90E-05
Rv1141c	echA11	0.4	-0.2	-0.5	1	1	1	0.019487633	0.073640208	2.46E-05
Rv0495c	hypothetical protein	1.1	-0.4	-0.5	2	1	1	4.42E-23	0.00053172	8.28E-06
Rv0154c	fadE2	-0.5	0.7	-0.5	1	2	1	0.00158319	6.64E-12	3.67E-06
Rv2774c	hypothetical protein	0.0	0.4	-0.5	1	1	1	0.954227404	0.000132732	1.91E-06
Rv2257c	hypothetical protein	0.1	1.6	-0.5	1	3	1	0.287550534	3.56E-20	1.93E-05
Rv1447c	zwf2	0.8	0.2	-0.5	2	1	1	7.46E-09	0.082479257	5.22E-06
Rv2092c	helY	-0.2	0.2	-0.5	1	1	1	0.096133178	0.078878473	3.17E-06
Rv0026	hypothetical protein	-0.1	-0.6	-0.5	1	1	1	0.221409486	3.40E-07	3.25E-06
Rv0633c	hypothetical protein	0.9	-0.1	-0.5	2	1	1	1.07E-14	0.278280523	6.83E-06
Rv2211c	gcvT	-0.1	0.6	-0.5	1	2	1	0.431665712	1.08E-06	9.23E-06
Rv3258c	hypothetical protein	-0.3	0.5	-0.5	1	1	1	0.034198423	0.001517302	9.60E-05
Rv1596	nadC	0.1	0.2	-0.5	1	1	1	0.517636858	0.141445706	2.31E-06
Rv0496	hypothetical protein	0.0	0.1	-0.5	1	1	1	0.888505032	0.416408245	6.85E-06
Rv2583c	relA	0.3	-1.0	-0.5	1	2	1	0.017576759	3.19E-19	1.29E-06
Rv1299	prfA	0.0	1.3	-0.5	1	2	1	0.961391407	1.47E-28	1.59E-05
Rv2547	vapB19	-1.3	0.6	-0.5	2	2	1	5.76E-13	0.000224173	0.000429294
Rv2508c	integral membrane protein	0.1	0.0	-0.5	1	1	1	0.375187352	0.790954755	1.86E-06
Rv1536	ileS	0.4	0.3	-0.5	1	1	1	0.000219592	0.002477394	1.62E-06
Rv3796	hypothetical protein	-0.6	1.4	-0.5	2	3	1	8.84E-08	1.24E-37	1.57E-06
Rv1509	hypothetical protein	0.3	0.5	-0.5	1	1	1	0.047993363	1.95E-05	3.49E-05
Rv3152	nuoH	0.3	1.2	-0.5	1	2	1	0.005129616	7.42E-23	6.05E-06
Rv1449c	tkt	0.4	0.4	-0.5	1	1	1	4.81E-05	0.00026999	5.24E-07
Rv0761c	adhB	0.9	0.3	-0.5	2	1	1	1.00E-16	0.005948035	2.14E-06
Rv2845c	proS	-0.5	1.2	-0.5	1	2	1	0.000123546	2.10E-26	1.69E-06
Rv0240	vapC24	0.6	0.1	-0.5	2	1	1	6.14E-08	0.595707896	2.81E-05
Rv0227c	membrane protein	0.1	0.6	-0.5	1	1	1	0.362048604	4.08E-07	1.01E-06
Rv2151c	ftsQ	0.0	-0.4	-0.5	1	1	1	0.961354688	0.00044177	2.48E-06
Rv3200c	transmembrane cation transporter	0.6	0.5	-0.5	2	1	1	2.89E-08	8.64E-06	3.32E-06
Rv0225	hypothetical protein	-0.9	1.7	-0.5	2	3	1	5.71E-14	5.08E-28	2.53E-06
Rv2798c	hypothetical protein	0.2	1.0	-0.5	1	2	1	0.248991448	5.53E-11	2.26E-05

Rv1442	bisC	0.0	-0.2	-0.5	1	1	1	0.745526137	0.055264917	7.31E-07
Rv2183c	hypothetical protein	-0.2	0.1	-0.5	1	1	1	0.127993051	0.437992191	7.86E-05
Rv1007c	metS	-0.6	0.8	-0.5	1	2	1	5.70E-06	1.28E-12	4.08E-06
Rv1425	diacyglycerol O-acyltransferase	-0.3	0.2	-0.5	1	1	1	0.007938598	0.050834799	3.74E-06
Rv3818	hypothetical protein	0.9	-0.2	-0.5	2	1	1	8.19E-16	0.041796762	5.56E-06
Rv2331A	hypothetical protein	0.0	0.1	-0.5	1	1	1	0.855860641	0.594308283	0.000207813
Rv1639c	hypothetical protein	0.5	-0.6	-0.5	1	1	1	1.75E-05	3.81E-08	4.43E-07
Rv0522	gabP	0.0	-0.2	-0.5	1	1	1	0.762672028	0.086065378	8.70E-07
Rv2655c	prophage protein	-0.4	-0.8	-0.5	1	2	1	0.001029319	7.74E-13	1.07E-06
Rv1744c	membrane protein	0.3	-0.3	-0.5	1	1	1	0.035560826	0.019142925	2.05E-05
Rv2795c	hypothetical protein	0.7	0.6	-0.5	2	2	1	2.61E-09	2.00E-07	7.08E-07
Rv1783	eccC5	0.8	-1.2	-0.5	2	2	1	1.30E-12	1.55E-38	1.13E-06
Rv1241	vapB33	0.9	1.1	-0.5	2	2	1	1.76E-11	1.52E-11	0.000105259
Rv2308a	hypothetical protein CDS	1.1	-0.5	-0.6	2	1	1	9.75E-12	0.00014516	4.06E-05
Rv0280	PPE3	-1.5	1.3	-0.6	3	2	1	8.12E-31	4.49E-34	2.72E-06
Rv3526	kshA	-0.6	0.3	-0.6	1	1	1	6.07E-07	0.019215324	9.62E-07
Rv3767c	S-adenosylmethionine-dependent me	0.1	-0.5	-0.6	1	1	1	0.601097055	4.88E-06	1.21E-06
Rv3902c	hypothetical protein	0.4	0.3	-0.6	1	1	1	0.042683535	0.068173859	0.000365643
Rv2771c	hypothetical protein	-0.1	0.5	-0.6	1	1	1	0.634453756	0.000124659	3.58E-06
Rv2223c	carboxylesterase B	-0.1	0.5	-0.6	1	1	1	0.418925822	2.10E-05	2.13E-06
Rv1389	gmk	-0.3	0.8	-0.6	1	2	1	0.00564353	3.53E-12	0.000190596
Rv0187	O-methyltransferase	-0.2	0.3	-0.6	1	1	1	0.063836145	0.010363499	2.39E-06
Rv1625c	cya	0.3	-1.0	-0.6	1	2	1	0.015615006	1.43E-18	9.81E-07
Rv1491c	TVP38/TMEM64 family membrane pro	0.2	-0.2	-0.6	1	1	1	0.174649239	0.077637703	2.16E-06
Rv0040c	mtc28	-1.1	1.8	-0.6	2	3	1	1.26E-21	4.90E-51	4.88E-06
Rv0875c	hypothetical protein	0.3	-0.8	-0.6	1	2	1	0.006029671	5.50E-11	3.06E-06
Rv0484c	short-chain type oxidoreductase	-0.6	0.7	-0.6	1	2	1	0.000172468	2.26E-07	2.62E-06
Rv1275	lprC	1.1	-0.6	-0.6	2	2	1	4.06E-19	3.23E-07	2.94E-06
Rv2135c	hypothetical protein	0.1	-0.3	-0.6	1	1	1	0.478334368	0.009236308	3.21E-06
Rv1157c	hypothetical protein	-0.1	0.4	-0.6	1	1	1	0.580383483	0.000194022	4.57E-06
Rv3133c	devR	1.0	0.1	-0.6	2	1	1	3.54E-19	0.628343165	2.71E-06
Rv3868	eccA1	0.7	-0.7	-0.6	2	2	1	3.35E-12	5.20E-13	4.67E-06
Rv2791c	transposase	0.8	-0.4	-0.6	2	1	1	7.53E-11	0.003206844	1.16E-07
Rv3114	hypothetical protein	-0.5	-0.7	-0.6	1	2	1	0.003353794	5.34E-06	5.05E-05
Rv1916	aceAb	0.9	-0.8	-0.6	2	2	1	6.34E-15	1.10E-12	8.34E-07
Rv2307A	hypothetical protein	-0.6	0.7	-0.6	2	2	1	0.00010779	2.04E-06	3.78E-05
Rv0938	ligD	0.2	-1.1	-0.6	1	2	1	0.177110985	6.05E-28	2.70E-07
Rv1905c	aao	0.0	0.0	-0.6	1	1	1	0.695942201	0.878477447	3.11E-06
Rv1624c	membrane protein	0.3	-0.5	-0.6	1	1	1	0.012687364	3.90E-06	7.09E-07
Rv1130	prpD	-4.4	0.7	-0.6	22	2	1	1.14E-154	9.55E-12	8.47E-08
Rv1443c	hypothetical protein	-0.1	-0.7	-0.6	1	2	1	0.461415968	4.11E-07	1.69E-05
Rv0888	hypothetical protein	0.0	0.2	-0.6	1	1	1	0.948241844	0.036890674	1.86E-07
Rv0555	menD	0.3	0.8	-0.6	1	2	1	0.070446228	2.03E-13	1.22E-07
Rv3151	nuoG	0.6	1.2	-0.6	2	2	1	1.36E-06	5.42E-30	1.32E-07
Rv3323c	moaX	-0.5	0.9	-0.6	1	2	1	0.000379466	4.24E-09	5.72E-06
Rv2202c	adoK	0.0	0.1	-0.6	1	1	1	0.743814107	0.320768271	5.38E-08

Rv0902c	prrB	-0.3	-0.6	-0.6	1	1	1	0.066759357	3.01E-07	1.91E-07
Rv0548c	menB	-0.5	0.2	-0.6	1	1	1	0.001195102	0.111565975	2.78E-07
Rv2531c	amino acid decarboxylase	0.3	0.8	-0.6	1	2	1	0.010525392	8.78E-16	1.61E-07
Rv0595c	vapC4	-0.3	1.0	-0.6	1	2	1	0.036208337	4.63E-09	2.12E-05
Rv2858c	aldC	0.0	0.5	-0.6	1	1	1	0.802692286	1.65E-06	1.91E-07
Rv2725c	hflX	-0.5	0.9	-0.6	1	2	1	2.20E-06	1.31E-18	8.41E-07
Rv2125	hypothetical protein	-0.1	0.0	-0.6	1	1	2	0.58631366	0.810167146	2.86E-07
Rv2876	transmembrane protein	0.3	-0.2	-0.6	1	1	2	0.05129955	0.081297885	3.61E-06
Rv2299c	htpG	0.9	0.8	-0.6	2	2	2	2.08E-16	2.89E-16	3.51E-08
Rv0928	pstS3	0.1	-0.2	-0.6	1	1	2	0.62041403	0.02118015	1.09E-07
Rv1592c	hypothetical protein	-0.2	0.8	-0.6	1	2	2	0.127160559	8.65E-16	4.66E-08
Rv1929c	hypothetical protein	-0.2	-0.9	-0.6	1	2	2	0.118989348	3.83E-10	8.91E-08
Rv3732	hypothetical protein	0.0	0.5	-0.6	1	1	2	0.907776165	9.34E-06	7.55E-07
Rv1338	murI	0.1	-0.5	-0.6	1	1	2	0.343349913	1.16E-05	1.73E-06
Rv1342c	hypothetical protein	0.3	0.1	-0.6	1	1	2	0.037247706	0.419885335	2.55E-05
Rv0199	membrane protein	-0.5	0.7	-0.6	1	2	2	0.000214134	5.96E-07	3.11E-07
Rv3748	hypothetical protein	0.5	0.4	-0.6	1	1	2	0.002226576	0.016072227	1.36E-05
Rv2772c	transmembrane protein	-0.3	0.4	-0.6	1	1	2	0.017753075	0.005465182	3.45E-06
Rv2175c	DNA-binding protein	-0.7	-0.3	-0.6	2	1	2	0.000899743	0.084481777	1.60E-05
Rv1009	rpfB	0.1	0.7	-0.6	1	2	2	0.460590261	1.61E-08	8.19E-08
Rv2537c	aroD	0.1	0.5	-0.6	1	1	2	0.618586151	0.00047027	9.59E-07
Rv3120	hypothetical protein	0.5	0.0	-0.6	1	1	2	5.80E-05	0.987860706	1.57E-07
Rv3316	sdhC	0.8	0.4	-0.6	2	1	2	2.52E-09	0.005342162	4.56E-06
Rv2589	gabT	0.0	-0.3	-0.6	1	1	2	0.930318727	0.004427683	2.44E-08
Rv2197c	transmembrane protein	0.5	0.0	-0.6	1	1	2	1.13E-06	0.909073421	3.82E-08
Rv0507	mmpL2	-0.4	1.4	-0.6	1	3	2	0.000153267	2.92E-39	3.99E-08
Rv3302c	glpD2	-0.3	0.5	-0.6	1	1	2	0.017560325	1.72E-06	1.30E-08
Rv3247c	tmk	-0.1	0.7	-0.6	1	2	2	0.741548535	7.42E-06	3.39E-06
Rv1682	hypothetical protein	0.7	-0.2	-0.6	2	1	2	8.71E-09	0.136602812	1.29E-07
Rv0876c	transmembrane protein	0.2	-0.4	-0.6	1	1	2	0.0570093	7.60E-05	7.14E-09
Rv1513	hypothetical protein	0.6	0.9	-0.6	2	2	2	2.56E-07	2.66E-12	7.33E-07
Rv2436	rbsK	-1.1	0.6	-0.6	2	1	2	2.95E-15	2.36E-05	1.05E-07
Rv1257c	oxidoreductase	-0.8	0.8	-0.6	2	2	2	2.88E-10	8.59E-12	1.91E-08
Rv2521	bcp	1.5	-0.4	-0.6	3	1	2	6.41E-36	0.001043486	7.54E-07
Rv3217c	integral membrane protein	-0.5	0.1	-0.6	1	1	2	0.000181428	0.450581742	9.58E-08
Rv2171	lppM	0.6	-0.2	-0.6	2	1	2	1.58E-06	0.092078303	1.83E-07
Rv0341	iniB	-2.3	0.5	-0.6	5	1	2	3.46E-33	6.98E-07	4.85E-06
Rv0444c	rskA	-0.2	-0.1	-0.6	1	1	2	0.19042046	0.230968685	5.34E-06
Rv3740c	diacyglycerol O-acyltransferase	0.2	0.2	-0.6	1	1	2	0.152996028	0.064118141	9.33E-09
Rv1823	hypothetical protein	-0.1	0.7	-0.6	1	2	2	0.58196648	1.32E-08	1.61E-07
Rv3820c	papA2	0.6	0.1	-0.6	1	1	2	8.77E-08	0.224364041	1.89E-08
Rv3265c	wbbL1	-0.6	0.4	-0.6	1	1	2	0.001049549	0.006614232	1.16E-06
Rv3273	transmembrane carbonic anhydrase	0.7	0.8	-0.6	2	2	2	1.03E-12	3.57E-15	6.76E-08
Rv1545	hypothetical protein	-0.1	0.6	-0.6	1	2	2	0.475667471	9.21E-05	6.49E-06
Rv0859	acyltransferase	0.1	-0.6	-0.6	1	2	2	0.291814435	3.73E-09	2.31E-08
Rv1533	monooxygenase	-1.0	1.2	-0.6	2	2	2	4.86E-15	9.40E-26	2.27E-08

Rv1843c	guaB1	0.4	-0.3	-0.6	1	1	2	0.000434212	0.002501257	8.88E-09
Rv2706c	hypothetical protein	-0.6	-0.3	-0.6	1	1	2	9.16E-06	0.059706779	1.49E-05
Rv3528c	hypothetical protein	1.1	0.3	-0.6	2	1	2	7.56E-13	0.048039076	3.64E-07
Rv0656c	vapC6	-0.4	0.4	-0.6	1	1	2	0.004896144	0.005734535	1.85E-07
Rv2258c	transcriptional regulator	0.8	0.7	-0.6	2	2	2	0.0583974	2.71E-10	3.01E-09
Rv2749	hypothetical protein	-0.3	0.4	-0.6	1	1	2	0.044552647	0.009657572	2.49E-06
Rv3255c	manA	0.2	0.2	-0.6	1	1	2	0.125848435	0.048869158	1.70E-08
Rv2536	transmembrane protein	0.3	0.5	-0.6	1	1	2	0.030978508	0.000130416	5.99E-08
Rv0892	monooxygenase	-0.5	1.0	-0.6	1	2	2	0.000607949	8.64E-19	2.21E-09
Rv0756c	hypothetical protein	-0.2	-1.0	-0.6	1	2	2	0.179242104	4.48E-14	1.84E-07
Rv2473	hypothetical protein	0.2	0.2	-0.6	1	1	2	0.147670118	0.065508306	1.46E-07
Rv2315c	hypothetical protein	-0.1	0.1	-0.7	1	1	2	0.254648688	0.40398861	3.96E-09
Rv0920c	transposase	-0.7	1.1	-0.7	2	2	2	1.26E-06	1.60E-18	5.70E-08
Rv3557c	HTH-type transcriptional regulator Kst	0.6	0.1	-0.7	2	1	2	1.26E-06	0.489357616	1.18E-07
Rv1952	vapB14	-1.5	0.3	-0.7	3	1	2	2.47E-11	0.076525431	3.97E-06
Rv1497	lipL	0.2	0.2	-0.7	1	1	2	0.059975951	0.184418043	6.54E-08
Rv3890c	esxC	-0.1	1.3	-0.7	1	2	2	0.49878568	1.62E-14	4.29E-06
Rv2216	epimerase family protein	0.2	0.0	-0.7	1	1	2	0.314864006	0.742218185	2.88E-08
Rv3024c	trmU	-0.2	-0.2	-0.7	1	1	2	0.135087833	0.156906601	3.54E-08
Rv1836c	hypothetical protein	0.5	0.4	-0.7	1	1	2	2.72E-07	0.000960859	8.23E-09
Rv0257	hypothetical protein	0.2	-0.3	-0.7	1	1	2	0.06804171	0.012953308	5.81E-09
Rv2757c	vapC21	-0.3	1.2	-0.7	1	2	2	0.043995576	1.74E-13	1.89E-06
Rv3791	dprE2	0.2	1.7	-0.7	1	3	2	0.079147331	3.02E-31	1.51E-06
Rv1879	hypothetical protein	0.9	-0.5	-0.7	2	1	2	5.39E-14	2.11E-06	1.10E-08
Rv1229c	mrp	-0.4	-0.6	-0.7	1	1	2	0.00252312	2.74E-06	1.03E-08
Rv0869c	moaA2	-0.3	0.8	-0.7	1	2	2	0.024133824	3.54E-11	3.15E-09
Rv3574	kstR	-0.1	-0.2	-0.7	1	1	2	0.55746304	0.15118968	1.90E-07
Rv0418	lpqL	-0.1	0.5	-0.7	1	1	2	0.608760326	3.41E-07	5.00E-10
Rv1172c	PE12	0.0	0.0	-0.7	1	1	2	0.980599484	0.945642645	5.66E-09
Rv3747	hypothetical protein	0.7	0.6	-0.7	2	2	2	1.50E-08	1.31E-05	4.76E-08
Rv2198c	mmpS3	0.1	1.2	-0.7	1	2	2	0.366597532	1.39E-27	5.79E-08
Rv0340	hypothetical protein	-0.4	0.2	-0.7	1	1	2	0.001646043	0.0454884	1.26E-08
Rv1253	deaD	0.0	-0.5	-0.7	1	1	2	0.941535168	1.01E-05	8.85E-10
Rv1121	zwf1	0.5	-0.5	-0.7	1	1	2	1.28E-05	2.57E-05	1.20E-09
Rv1858	modB	-0.2	0.3	-0.7	1	1	2	0.080252493	0.017243195	1.04E-08
Rv0242c	fabG4	-0.3	1.4	-0.7	1	3	2	0.060230402	1.07E-39	1.91E-10
Rv2889c	tsf	0.9	0.5	-0.7	2	1	2	7.41E-16	4.66E-05	4.78E-09
Rv3259	hypothetical protein	1.5	0.3	-0.7	3	1	2	6.14E-17	0.055853035	9.68E-07
Rv3010c	pfkA	0.7	-1.1	-0.7	2	2	2	4.27E-10	3.35E-29	2.56E-10
Rv1417	membrane protein	0.7	0.3	-0.7	2	1	2	5.06E-06	0.039517555	1.39E-08
Rv0861c	ercc3	0.2	-0.1	-0.7	1	1	2	0.25228998	0.198620581	1.05E-07
Rv3598c	lysS	0.8	1.3	-0.7	2	2	2	1.79E-11	3.88E-27	3.56E-10
Rv2349c	plcC	-0.8	1.3	-0.7	2	2	2	1.93E-11	4.51E-29	1.86E-09
Rv2894c	xerC	-0.4	1.2	-0.7	1	2	2	0.041942073	4.07E-20	5.54E-09
Rv0579	hypothetical protein	-0.4	-0.8	-0.7	1	2	2	0.012109624	1.61E-11	3.20E-09
Rv2704	hypothetical protein	0.4	0.0	-0.7	1	1	2	0.028027095	0.978694885	2.19E-07

Rv2154c	ftsW	0.3	1.5	-0.7	1	3	2	0.012767632	4.88E-37	4.41E-11
Rv0645c	mmaA1	0.0	0.8	-0.7	1	2	2	0.859027667	8.65E-12	1.14E-08
Rv0434	hypothetical protein	-0.2	0.9	-0.7	1	2	2	0.15966642	5.46E-12	2.90E-09
Rv0471c	hypothetical protein	-0.2	0.1	-0.7	1	1	2	0.09384596	0.311883008	2.11E-09
Rv3715c	recR	0.8	0.9	-0.7	2	2	2	1.89E-11	2.78E-13	1.64E-09
Rv1771	L-gulono-1,4-lactone dehydrogenase	1.5	-0.8	-0.7	3	2	2	1.34E-44	1.03E-12	1.03E-09
Rv2209	integral membrane protein	-0.1	-0.2	-0.7	1	1	2	0.54008644	0.099908005	6.48E-11
Rv2107	PE22	0.9	0.4	-0.7	2	1	2	9.35E-12	0.020231171	4.16E-08
Rv2543	lppA	0.1	0.0	-0.7	1	1	2	0.278690936	0.773379589	2.07E-07
Rv3675	membrane protein	-0.9	0.3	-0.7	2	1	2	7.16E-10	0.027065123	6.61E-09
Rv2565	NTE family protein	-0.2	1.0	-0.7	1	2	2	0.070613603	1.60E-20	2.17E-09
Rv0885	hypothetical protein	-1.8	1.6	-0.7	3	3	2	2.67E-42	1.96E-39	2.10E-10
Rv0523c	hypothetical protein	-1.0	1.2	-0.7	2	2	2	2.38E-11	8.80E-16	1.84E-08
Rv2533c	nusB	-0.8	0.6	-0.7	2	2	2	2.14E-05	4.86E-06	3.67E-08
Rv3396c	guaA	0.6	0.4	-0.7	2	1	2	4.04E-08	8.21E-05	3.78E-10
Rv0936	pstA2	0.0	0.8	-0.7	1	2	2	0.928221704	3.11E-12	6.74E-10
Rv0029	hypothetical protein	-0.5	0.1	-0.7	1	1	2	0.001161964	0.566213943	1.62E-09
Rv3753c	hypothetical protein	-1.0	1.1	-0.7	2	2	2	6.17E-12	1.25E-14	6.11E-09
Rv2024c	hypothetical protein	0.2	0.6	-0.7	1	2	2	0.149309646	6.68E-09	9.25E-11
Rv1209	hypothetical protein	-0.3	-0.7	-0.7	1	2	2	0.19441082	1.24E-05	1.26E-07
Rv0176	Mce associated transmembrane prote	0.4	1.6	-0.7	1	3	2	0.000343036	3.69E-46	5.32E-11
Rv2946c	pks1	0.1	1.7	-0.7	1	3	2	0.400158656	3.72E-41	6.80E-10
Rv1074c	fadA3	-0.2	0.7	-0.7	1	2	2	0.124548199	2.74E-09	1.30E-10
Rv0907	hypothetical protein	0.8	0.7	-0.7	2	2	2	2.98E-10	1.18E-11	3.61E-10
Rv3758c	proV	0.3	-0.5	-0.7	1	1	2	0.02301573	7.31E-06	7.68E-11
Rv2991	hypothetical protein	-1.6	0.2	-0.7	3	1	2	7.26E-21	0.163669311	9.45E-09
Rv2799	membrane protein	-1.2	1.3	-0.7	2	2	2	1.90E-22	1.36E-26	1.20E-10
Rv0048c	membrane protein	-0.9	0.4	-0.7	2	1	2	1.94E-10	0.000907807	5.50E-10
Rv2463	lipP	0.0	0.0	-0.7	1	1	2	0.686489552	0.929687999	1.59E-11
Rv0786c	hypothetical protein	0.7	0.4	-0.7	2	1	2	6.07E-08	0.001752789	1.11E-08
Rv3157	nuoM	0.2	0.6	-0.7	1	1	2	0.090785757	2.09E-08	1.10E-10
Rv2764c	thyA	0.5	-1.3	-0.7	1	3	2	6.62E-06	1.57E-30	6.47E-09
Rv1740	vapB34	0.1	-0.3	-0.7	1	1	2	0.697112366	0.076615368	6.85E-06
Rv2751	hypothetical protein	0.3	-0.3	-0.7	1	1	2	0.005223797	0.021347132	6.71E-09
Rv2374c	hrcA	0.1	-0.4	-0.7	1	1	2	0.5092256	0.000166206	2.54E-12
Rv3823c	mmpL8	0.1	0.3	-0.7	1	1	2	0.477743124	0.00223359	6.22E-13
Rv2313c	hypothetical protein	0.4	-0.3	-0.7	1	1	2	0.001726022	0.001972937	3.97E-11
Rv0755c	PPE12	0.1	0.2	-0.7	1	1	2	0.438345255	0.10828384	1.83E-10
Rv0139	oxidoreductase	-0.8	1.9	-0.7	2	4	2	2.00E-09	1.17E-48	2.95E-11
Rv1766	hypothetical protein	0.8	-0.6	-0.7	2	2	2	3.40E-10	5.47E-07	7.73E-09
Rv1628c	hypothetical protein	0.1	-0.7	-0.7	1	2	2	0.352652275	2.76E-08	3.42E-11
Rv2296	haloalkane dehalogenase	-0.4	0.1	-0.8	1	1	2	0.020129606	0.596134477	8.89E-12
Rv1541c	lprI	0.6	-0.4	-0.8	1	1	2	1.15E-05	0.001546217	1.25E-10
Rv1474c	transcriptional regulator	0.6	0.1	-0.8	2	1	2	8.39E-07	0.509024872	6.45E-08
Rv0760c	hypothetical protein	1.4	-0.2	-0.8	3	1	2	7.08E-32	0.082449822	5.39E-09
Rv1822	pgsA2	0.1	0.7	-0.8	1	2	2	0.222259751	1.04E-07	3.23E-11

Rv3883c	mycP1	-0.5	0.9	-0.8	1	2	2	0.00319479	1.36E-17	4.69E-12
Rv2523c	acpS	-1.1	1.8	-0.8	2	3	2	5.56E-07	1.26E-30	8.81E-10
Rv0713	transmembrane protein	-0.3	0.3	-0.8	1	1	2	0.032211962	0.024126145	1.07E-11
Rv2857c	3-oxoacyl-ACP reductase	0.4	0.4	-0.8	1	1	2	0.001276778	0.000356019	1.82E-11
Rv0663	atsD	0.3	0.1	-0.8	1	1	2	0.005860574	0.354198921	3.85E-13
Rv3149	nuoE	0.0	1.9	-0.8	1	4	2	0.878237239	1.06E-34	4.75E-09
Rv2593c	ruvA	0.1	1.7	-0.8	1	3	2	0.695366385	2.53E-27	8.88E-10
Rv1154c	hypothetical protein	-0.4	0.3	-0.8	1	1	2	0.001389338	0.025074312	5.00E-08
Rv1334	mec	0.3	-0.7	-0.8	1	2	2	0.009138488	1.22E-12	4.23E-09
Rv1769	hypothetical protein	0.4	0.4	-0.8	1	1	2	0.000268978	4.59E-05	1.78E-12
Rv1448c	tal	1.5	-0.6	-0.8	3	1	2	4.47E-38	6.06E-08	4.91E-12
Rv3706c	hypothetical protein	-0.6	1.2	-0.8	1	2	2	3.12E-05	9.83E-20	2.08E-07
Rv3413c	anti-sigma-D factor RsdA	0.2	0.0	-0.8	1	1	2	0.256886793	0.821305329	5.42E-12
Rv0088	polyketide cyclase/dehydrase	0.0	-0.4	-0.8	1	1	2	0.742426513	0.002171817	8.43E-11
Rv3384c	vapC46	-0.5	0.5	-0.8	1	1	2	0.010005822	0.001085595	1.34E-09
Rv2789c	fadE21	-0.1	0.2	-0.8	1	1	2	0.571825712	0.040949768	1.27E-11
Rv1902c	nanT	-0.3	0.6	-0.8	1	1	2	0.007817477	2.34E-07	6.97E-11
Rv2708c	hypothetical protein	-0.1	-0.1	-0.8	1	1	2	0.583782409	0.630895042	2.07E-10
Rv1201c	dapD	1.0	0.6	-0.8	2	2	2	3.85E-15	2.82E-07	7.20E-12
Rv2237	hypothetical protein	-0.2	0.4	-0.8	1	1	2	0.242362172	0.001558655	2.91E-10
Rv0461	transmembrane protein	-0.1	0.5	-0.8	1	1	2	0.643035301	0.00184525	5.33E-08
Rv3048c	nrdF2	-0.3	0.6	-0.8	1	2	2	0.010842289	5.83E-10	2.52E-08
Rv0079	hypothetical protein	0.6	-0.4	-0.8	2	1	2	0.001336288	0.001900154	1.18E-10
Rv0149	quinone oxidoreductase	0.1	0.4	-0.8	1	1	2	0.727492051	0.001487848	1.59E-11
Rv3093c	oxidoreductase	-1.5	1.7	-0.8	3	3	2	4.45E-19	1.44E-40	5.21E-12
Rv0112	gca	0.2	0.7	-0.8	1	2	2	0.147348149	1.06E-09	4.13E-10
Rv0808	purF	0.0	-0.5	-0.8	1	1	2	0.856405543	6.85E-07	3.22E-13
Rv1942c	mazF5	0.5	0.6	-0.8	1	2	2	0.00125333	2.60E-07	2.18E-09
Rv2775	GCN5-like N-acetyltransferase	0.8	0.1	-0.8	2	1	2	3.19E-10	0.500511491	2.16E-09
Rv1240	mdh	0.1	1.0	-0.8	1	2	2	0.294130427	1.16E-15	2.25E-12
Rv1140	integral membrane protein	0.1	-0.2	-0.8	1	1	2	0.589920992	0.079467008	3.22E-12
Rv2426c	hypothetical protein	0.4	1.3	-0.8	1	3	2	0.000129126	6.84E-29	5.67E-12
Rv1019	transcriptional regulator	-0.3	-0.8	-0.8	1	2	2	0.024535546	1.05E-14	2.70E-12
Rv3268	hypothetical protein	-0.2	0.8	-0.8	1	2	2	0.209065137	1.06E-08	6.16E-11
Rv2326c	ABC transporter ATP-binding protein	0.6	-0.4	-0.8	1	1	2	4.35E-06	0.000109019	1.33E-11
Rv0003	recF	0.5	0.5	-0.8	1	1	2	0.000201845	2.96E-07	2.36E-12
Rv2096c	pafB	0.6	-0.3	-0.8	2	1	2	6.06E-08	0.00339265	8.47E-13
Rv1434	hypothetical protein	-0.1	1.4	-0.8	1	3	2	0.688081426	9.93E-06	0.000315757
Rv0288	esxH	0.2	0.8	-0.8	1	2	2	0.048011887	8.04E-14	2.26E-11
Rv2676c	hypothetical protein	-0.3	-0.3	-0.8	1	1	2	0.026014392	0.010231639	2.11E-10
Rv2124c	metH	0.4	-0.2	-0.8	1	1	2	0.000191637	0.013584817	1.82E-14
Rv1638A	hypothetical protein	0.8	-0.4	-0.8	2	1	2	1.19E-07	0.006806416	1.71E-07
Rv1480	hypothetical protein	0.4	0.2	-0.8	1	1	2	0.000171591	0.042818968	7.05E-13
Rv1052	hypothetical protein	0.1	-0.4	-0.8	1	1	2	0.420602757	0.003648121	2.67E-09
Rv3150	nuoF	0.4	1.3	-0.8	1	3	2	0.000510262	2.94E-32	4.50E-13
Rv0509	hemA	0.6	0.1	-0.8	2	1	2	4.38E-10	0.24653063	1.07E-14

Rv0787A	hypothetical protein	0.3	0.2	-0.8	1	1	2	0.048270978	0.14181033	3.26E-09
Rv2256a	hypothetical protein CDS	1.4	-0.7	-0.8	3	2	2	3.13E-25	5.06E-07	1.38E-08
Rv0075	aminotransferase	0.2	1.1	-0.8	1	2	2	0.11741673	4.67E-18	1.05E-13
Rv0653c	transcriptional regulator	-0.1	1.5	-0.8	1	3	2	0.324336045	3.43E-25	7.36E-11
Rv0023	transcriptional regulator	-0.7	0.9	-0.8	2	2	2	2.65E-07	6.49E-10	2.96E-11
Rv0894	transcriptional regulator	0.0	1.2	-0.8	1	2	2	0.783597656	5.38E-27	4.67E-13
Rv3390	lpqD	-0.7	0.4	-0.8	2	1	2	8.27E-08	0.000364426	6.70E-12
Rv0467	icl1	0.7	-0.6	-0.8	2	1	2	6.26E-07	5.12E-06	1.47E-10
Rv3646c	topA	0.4	1.0	-0.8	1	2	2	0.001344656	3.67E-20	1.84E-13
Rv3430c	transposase	-0.2	0.6	-0.8	1	2	2	0.227302147	6.83E-09	3.16E-14
Rv1290A	hypothetical protein	-0.4	1.1	-0.8	1	2	2	0.005294899	4.23E-16	4.35E-09
Rv3102c	ftsE	0.0	0.6	-0.8	1	2	2	0.71699656	1.16E-06	6.17E-12
Rv0170	mce1B	-0.2	1.4	-0.8	1	3	2	0.304857355	8.60E-34	2.16E-11
Rv1795	eccD5	0.6	0.2	-0.8	1	1	2	2.74E-07	0.114291158	1.37E-12
Rv3877	eccD1	0.3	0.0	-0.8	1	1	2	0.017138729	0.718657245	1.04E-14
Rv1292	argS	0.1	0.7	-0.8	1	2	2	0.561741569	1.61E-09	1.68E-14
Rv3147	nuoC	-0.2	1.3	-0.8	1	2	2	0.047502353	5.13E-24	3.61E-12
Rv2366c	transmembrane protein	0.4	0.0	-0.8	1	1	2	0.001129182	0.730162122	4.55E-14
Rv1265	hypothetical protein	-1.0	-0.6	-0.8	2	1	2	2.07E-11	8.71E-09	5.77E-12
Rv3784	dTDP-glucose 4,6-dehydratase	-0.1	1.0	-0.8	1	2	2	0.647223055	6.75E-17	2.71E-13
Rv2525c	hypothetical protein	-1.0	1.0	-0.8	2	2	2	1.51E-12	4.55E-14	4.17E-11
Rv1289	hypothetical protein	0.5	-0.2	-0.8	1	1	2	0.000447867	0.207817438	1.93E-11
Rv3570c	hsaA	-0.7	1.6	-0.8	2	3	2	6.42E-09	8.62E-50	2.16E-14
Rv3766	hypothetical protein	0.5	-0.4	-0.8	1	1	2	3.75E-05	0.001692041	2.42E-11
Rv2339	mmpL9	0.1	0.1	-0.8	1	1	2	0.167921607	0.432995547	1.94E-15
Rv1617	pykA	0.9	0.6	-0.8	2	2	2	1.23E-14	3.04E-08	1.78E-12
Rv3492c	Mce associated protein	1.2	0.7	-0.8	2	2	2	8.17E-16	9.28E-08	7.94E-12
Rv1488	hypothetical protein	0.0	-0.2	-0.8	1	1	2	0.717845364	0.036828137	2.07E-12
Rv2781c	oxidoreductase	0.2	0.0	-0.8	1	1	2	0.258194429	0.9858571	1.39E-13
Rv2022c	hypothetical protein	-0.6	0.1	-0.8	2	1	2	5.29E-07	0.62814323	4.30E-13
Rv2452c	hypothetical protein	1.0	0.0	-0.8	2	1	2	1.33E-10	0.80442507	5.55E-08
Rv3092c	integral membrane protein	-0.7	-0.8	-0.8	2	2	2	0.000604686	6.33E-10	4.39E-14
Rv2680	hypothetical protein	0.0	-0.2	-0.8	1	1	2	0.907301674	0.018366186	2.74E-13
Rv3826	fadD23	0.3	0.6	-0.8	1	2	2	0.016931832	6.70E-08	2.91E-12
Rv2581c	glyoxalase II	0.5	0.0	-0.8	1	1	2	0.00013057	0.659722015	1.13E-12
Rv0942	hypothetical protein	0.3	-1.3	-0.8	1	3	2	0.008537559	1.72E-23	2.51E-11
Rv2935	ppsE	-0.3	1.3	-0.8	1	2	2	0.000946399	3.25E-40	5.93E-13
Rv3685c	cyp137	-0.8	0.9	-0.8	2	2	2	4.35E-13	3.34E-18	8.71E-15
Rv1021	nucleoside triphosphate pyrophospho	-0.2	0.4	-0.8	1	1	2	0.186917023	0.001102873	3.12E-14
Rv3214	gpm2	-0.4	0.2	-0.8	1	1	2	0.010627253	0.084803021	3.34E-11
Rv3866	espG1	0.4	-0.6	-0.9	1	1	2	0.002564794	5.01E-07	1.94E-14
Rv2535c	pepQ	-0.3	0.6	-0.9	1	2	2	0.01573841	3.99E-08	1.15E-14
Rv3049c	monooxygenase	-0.1	0.9	-0.9	1	2	2	0.366863558	4.13E-20	6.66E-11
Rv0066c	icd2	1.2	-0.2	-0.9	2	1	2	3.06E-25	0.102531026	8.48E-16
Rv3881c	espB	0.4	0.4	-0.9	1	1	2	0.006059209	1.13E-05	1.42E-15
Rv0007	membrane protein	-0.1	0.4	-0.9	1	1	2	0.434997674	0.000472911	5.85E-11

Rv3131	NAD(P)H nitroreductase	1.0	-0.1	-0.9	2	1	2	5.53E-12	0.493960541	1.58E-10
Rv1649	pheS	-0.1	0.8	-0.9	1	2	2	0.259098025	2.93E-12	4.59E-15
Rv1169c	lipX	-1.2	-1.9	-0.9	2	4	2	1.37E-19	1.57E-63	5.29E-10
Rv3808c	glfT2	-0.4	0.8	-0.9	1	2	2	0.001600907	3.90E-14	1.80E-16
Rv0759c	hypothetical protein	1.0	-0.3	-0.9	2	1	2	3.14E-12	0.044814477	4.19E-13
Rv2520c	membrane protein	0.5	-0.1	-0.9	1	1	2	1.25E-05	0.362587101	1.55E-12
Rv3809c	glf	-0.4	0.7	-0.9	1	2	2	0.00055569	1.50E-10	1.34E-13
Rv0404	fadD30	0.1	0.5	-0.9	1	1	2	0.411447855	0.000100998	1.31E-11
Rv0866	moaE2	0.0	0.0	-0.9	1	1	2	0.770122994	0.747590033	8.07E-11
Rv3596c	clpC1	-0.2	0.1	-0.9	1	1	2	0.049266438	0.172257915	1.70E-13
Rv1609	trpE	0.2	1.0	-0.9	1	2	2	0.073324762	7.29E-20	3.74E-16
Rv0609	vapC28	0.6	0.3	-0.9	2	1	2	1.41E-06	0.028840113	1.48E-11
Rv0316	muconolactone isomerase	-0.9	1.1	-0.9	2	2	2	1.60E-08	6.24E-16	5.81E-10
Rv1300	hemK	0.0	0.2	-0.9	1	1	2	0.726672688	0.080306071	5.47E-16
Rv3588c	canB	-0.4	0.9	-0.9	1	2	2	0.004262294	1.48E-11	1.03E-12
Rv1561	vapC11	-0.3	0.4	-0.9	1	1	2	0.050698734	0.011948407	1.96E-10
Rv0889c	citA	-0.3	0.0	-0.9	1	1	2	0.032062105	0.782625262	1.35E-14
Rv3520c	coenzyme F420-dependent oxidoredu	0.4	0.0	-0.9	1	1	2	0.001399661	0.739604535	2.43E-15
Rv2476c	gdh	0.5	0.3	-0.9	1	1	2	4.11E-07	0.001120789	7.03E-17
Rv0658c	integral membrane protein	0.2	0.1	-0.9	1	1	2	0.193892697	0.203385096	2.42E-14
Rv0276	hypothetical protein	-0.7	-0.4	-0.9	2	1	2	3.13E-07	0.000768542	1.22E-14
Rv0819	mshD	0.2	0.0	-0.9	1	1	2	0.169855121	0.79199562	4.00E-13
Rv2391	sirA	1.6	-0.2	-0.9	3	1	2	4.24E-56	0.090856962	1.70E-12
Rv0157A	hypothetical protein	-0.3	1.2	-0.9	1	2	2	0.050955168	9.41E-09	4.27E-09
Rv1356c	hypothetical protein	-0.2	0.3	-0.9	1	1	2	0.112467051	0.005397216	1.65E-10
Rv2364c	era	-0.3	0.2	-0.9	1	1	2	0.068179452	0.150040403	8.25E-13
Rv3044	fecB	-0.2	1.1	-0.9	1	2	2	0.05994623	6.90E-19	1.78E-15
Rv2748c	ftsK	-0.1	0.8	-0.9	1	2	2	0.577452804	5.01E-17	6.74E-17
Rv3182	hypothetical protein	-1.3	-0.9	-0.9	2	2	2	1.42E-07	2.08E-14	6.89E-12
Rv2856	nicT	0.0	0.9	-0.9	1	2	2	0.888258992	1.79E-17	3.26E-15
Rv1444c	hypothetical protein	-0.1	0.4	-0.9	1	1	2	0.676162946	0.015124655	3.55E-11
Rv2959c	rhamnosyl O-methyltransferase	0.8	1.3	-0.9	2	2	2	4.64E-07	8.88E-32	3.52E-15
Rv3162c	integral membrane protein	0.3	1.6	-0.9	1	3	2	0.091114124	2.17E-39	2.05E-11
Rv3867	espH	0.8	-1.0	-0.9	2	2	2	1.54E-08	1.85E-27	4.29E-11
Rv3156	nuoL	0.4	1.1	-0.9	1	2	2	0.000669492	9.36E-24	7.05E-16
Rv1210	tagA	0.8	-0.3	-0.9	2	1	2	1.05E-11	0.004479273	2.63E-14
Rv3042c	serB2	0.2	0.9	-0.9	1	2	2	0.116902056	2.45E-17	1.12E-15
Rv1505c	hypothetical protein	0.4	1.1	-0.9	1	2	2	0.000734708	2.80E-22	3.33E-12
Rv1015c	rplY	-0.2	1.1	-0.9	1	2	2	0.185576116	1.35E-22	5.02E-14
Rv2061c	hypothetical protein	0.9	0.9	-0.9	2	2	2	6.79E-10	6.83E-10	2.87E-12
Rv3311	hypothetical protein	-0.3	0.9	-0.9	1	2	2	0.011313323	6.25E-19	1.07E-15
Rv1215c	hypothetical protein	-0.7	-0.1	-0.9	2	1	2	8.97E-07	0.352686857	1.18E-16
Rv0314c	membrane protein	-0.1	-0.6	-0.9	1	2	2	0.279849367	8.06E-08	1.97E-14
Rv1372	alpha-pyrone synthesis polyketide syr	-0.6	-1.1	-0.9	2	2	2	0.000688026	1.76E-13	4.83E-09
Rv2007c	fdxA	2.2	-1.6	-0.9	5	3	2	5.50E-77	2.68E-50	1.38E-14
Rv1359	transcriptional regulator	0.0	-1.2	-0.9	1	2	2	0.824512375	9.74E-28	7.68E-16

Rv3191c	transposase	1.3	0.1	-0.9	3	1	2	6.43E-24	0.572893317	3.28E-16
Rv2108	PPE36	0.6	0.1	-0.9	2	1	2	1.39E-06	0.206265048	1.13E-15
Rv1616	hypothetical protein	0.1	-0.2	-0.9	1	1	2	0.421108619	0.334269562	3.12E-09
Rv1618	tesB1	0.6	0.9	-0.9	1	2	2	4.33E-07	2.66E-16	1.62E-16
Rv0295c	hypothetical protein	-0.1	1.4	-0.9	1	3	2	0.283154045	3.04E-29	2.54E-15
Rv1132	hypothetical protein	0.2	0.9	-0.9	1	2	2	0.033893728	2.36E-17	1.81E-17
Rv1343c	lprD	-0.1	0.4	-0.9	1	1	2	0.497359952	0.008035241	3.45E-12
Rv0157	pntB	-0.3	2.2	-0.9	1	5	2	0.001930105	1.31E-91	3.75E-17
Rv2747	argA	0.4	0.9	-0.9	1	2	2	0.00578245	4.68E-09	6.14E-12
Rv3915	peptidoglycan hydrolase	-0.2	0.2	-0.9	1	1	2	0.173164467	0.187372352	9.85E-15
Rv0025	hypothetical protein	0.0	0.3	-0.9	1	1	2	0.8467722	0.019304532	7.90E-13
Rv1559	ilvA	-0.2	1.0	-0.9	1	2	2	0.168698396	2.67E-19	3.71E-17
Rv0908	ctpE	0.7	1.0	-0.9	2	2	2	1.00E-05	5.32E-24	6.34E-18
Rv1018c	glmU	-0.5	0.7	-0.9	1	2	2	4.82E-05	1.08E-11	8.90E-17
Rv0615	integral membrane protein	-0.3	-1.2	-0.9	1	2	2	0.015064968	1.92E-24	4.42E-10
Rv3154	nuoJ	0.2	1.0	-0.9	1	2	2	0.127494047	4.30E-19	6.30E-17
Rv2957	PGL/p-HBAD biosynthesis glycosyltra	1.0	1.4	-0.9	2	3	2	2.83E-17	4.71E-28	3.66E-15
Rv0678	hypothetical protein	-0.1	-0.4	-0.9	1	1	2	0.49006092	1.07E-05	1.24E-19
Rv2890c	rpsB	1.0	0.6	-0.9	2	2	2	6.88E-19	4.97E-09	5.41E-17
Rv2225	panB	-0.1	-0.9	-0.9	1	2	2	0.539648368	1.77E-15	7.92E-17
Rv1280c	oppA	-0.2	0.7	-0.9	1	2	2	0.208319809	2.18E-12	4.61E-17
Rv2705c	hypothetical protein	-0.4	0.1	-0.9	1	1	2	0.005944522	0.300357871	5.38E-12
Rv1845c	blaR	-0.7	-0.3	-0.9	2	1	2	0.00070017	0.023677665	3.84E-13
Rv2060	integral membrane protein	0.4	0.9	-0.9	1	2	2	0.001152053	9.92E-09	1.68E-11
Rv2471	aglA	0.3	-0.5	-0.9	1	1	2	0.010410937	2.87E-05	4.31E-18
Rv2214c	ephD	0.3	0.7	-0.9	1	2	2	0.013755618	1.02E-12	2.18E-18
Rv0925c	hypothetical protein	0.2	1.2	-0.9	1	2	2	0.160438488	9.23E-23	1.47E-13
Rv1854c	ndh	0.3	-0.9	-0.9	1	2	2	0.00154158	5.06E-15	3.38E-14
Rv1274	lprB	0.6	-0.1	-0.9	1	1	2	2.32E-05	0.596313374	3.37E-16
Rv2438c	nadE	0.4	-0.3	-0.9	1	1	2	0.00123286	0.012118337	2.54E-18
Rv1315	murA	0.0	0.8	-1.0	1	2	2	0.938445732	9.38E-12	1.54E-16
Rv2067c	hypothetical protein	0.6	1.3	-1.0	2	2	2	2.55E-07	1.74E-30	2.72E-13
Rv0911	hypothetical protein	0.2	1.0	-1.0	1	2	2	0.170917421	8.36E-14	3.49E-14
Rv2777c	hypothetical protein	0.0	0.0	-1.0	1	1	2	0.916688631	0.890221011	9.12E-16
Rv0884c	serC	-0.7	0.3	-1.0	2	1	2	9.48E-11	0.003595285	2.03E-17
Rv2750	dehydrogenase	-0.3	0.5	-1.0	1	1	2	0.043871816	0.000834398	3.08E-16
Rv2149c	yfiH	0.0	-0.2	-1.0	1	1	2	0.784009727	0.065872932	4.98E-16
Rv0679c	hypothetical protein	-0.1	-0.4	-1.0	1	1	2	0.494656891	0.000186382	6.49E-18
Rv2584c	apt	0.6	-0.6	-1.0	2	1	2	5.96E-06	8.30E-07	8.05E-15
Rv0426c	transmembrane protein	0.5	0.1	-1.0	1	1	2	0.00034101	0.32189148	1.47E-15
Rv3301c	phoY1	0.7	0.5	-1.0	2	1	2	4.35E-06	1.04E-05	5.69E-16
Rv0156	pntAb	0.0	2.2	-1.0	1	5	2	0.82492148	6.59E-45	4.13E-12
Rv3028c	fixB	-0.2	1.1	-1.0	1	2	2	0.42333341	6.21E-17	1.68E-16
Rv1565c	acyltransferase	-0.3	1.6	-1.0	1	3	2	0.001564734	4.67E-40	3.69E-19
Rv0597c	hypothetical protein	-0.4	-0.2	-1.0	1	1	2	0.010465993	0.030214828	7.33E-18
Rv0113	gmhA	0.0	0.8	-1.0	1	2	2	0.737171429	1.71E-10	1.82E-15

Rv2998	hypothetical protein	-0.3	-0.2	-1.0	1	1	2	0.053171236	0.1172299	1.91E-14
Rv2579	dhaA	-0.9	1.4	-1.0	2	3	2	2.69E-08	1.85E-20	1.47E-11
Rv1901	cinA	-0.3	1.2	-1.0	1	2	2	0.020461524	5.58E-22	2.68E-16
Rv0806c	cpsY	0.6	0.0	-1.0	2	1	2	9.09E-08	0.981365339	9.63E-19
Rv3101c	ftsX	0.5	0.6	-1.0	1	2	2	5.17E-05	3.66E-08	8.14E-18
Rv3880c	espL	0.5	0.2	-1.0	1	1	2	0.000221594	0.154961098	2.38E-17
Rv3094c	hypothetical protein	-2.0	2.6	-1.0	4	6	2	7.93E-49	1.32E-86	1.66E-18
Rv0308	integral membrane protein	0.6	-0.3	-1.0	2	1	2	7.20E-08	0.022337668	1.82E-17
Rv1101c	hypothetical protein	-0.4	0.4	-1.0	1	1	2	0.000849811	0.000348594	3.24E-15
Rv0873	fadE10	0.3	0.3	-1.0	1	1	2	0.021633573	0.002003101	1.82E-20
Rv3725	oxidoreductase	0.0	0.6	-1.0	1	2	2	0.827814809	2.55E-06	8.77E-17
Rv1042c	IS2-like transposase	0.4	-1.0	-1.0	1	2	2	0.001596322	5.12E-21	1.11E-16
Rv2328	PE23	0.3	0.5	-1.0	1	1	2	0.019113499	0.000112139	1.15E-12
Rv0445c	sigK	0.1	0.4	-1.0	1	1	2	0.726744845	0.004947198	8.78E-14
Rv2210c	ilvE	0.3	0.3	-1.0	1	1	2	0.008719204	0.005430417	2.44E-18
Rv3442c	rpsI	0.5	1.2	-1.0	1	2	2	3.33E-05	3.33E-20	1.01E-16
Rv2575	membrane protein	0.8	-0.8	-1.0	2	2	2	1.89E-13	5.40E-14	6.66E-16
Rv3190c	hypothetical protein	0.2	0.8	-1.0	1	2	2	0.071570682	7.03E-12	2.85E-18
Rv3139	fadE24	0.6	-0.1	-1.0	2	1	2	1.50E-09	0.152929288	9.36E-19
Rv0788	purQ	0.7	-0.3	-1.0	2	1	2	1.31E-07	0.014595771	7.97E-16
Rv0293c	hypothetical protein	0.4	-2.3	-1.0	1	5	2	0.001075974	6.54E-88	7.17E-17
Rv1100	hypothetical protein	0.0	-0.8	-1.0	1	2	2	0.966793114	1.42E-10	5.45E-15
Rv0543c	hypothetical protein	1.1	0.0	-1.0	2	1	2	9.25E-18	0.853908372	2.32E-14
Rv3674c	nth	0.2	0.5	-1.0	1	1	2	0.166357995	0.000117193	1.95E-16
Rv2970c	lipN	0.3	0.5	-1.0	1	1	2	0.002410756	5.25E-06	5.63E-20
Rv1489A	hypothetical protein	-0.2	-0.3	-1.0	1	1	2	0.230975621	0.005557485	5.22E-16
Rv0452	transcriptional regulator	-0.4	1.9	-1.0	1	4	2	0.015703061	4.49E-52	5.39E-19
Rv2696c	hypothetical protein	-0.4	0.6	-1.0	1	1	2	0.000283067	1.72E-07	5.90E-16
Rv1363c	membrane protein	-0.2	0.1	-1.0	1	1	2	0.079266285	0.257967279	5.41E-18
Rv0489	gpm1	-0.1	0.6	-1.0	1	2	2	0.290375743	3.86E-07	3.44E-16
Rv3240c	secA1	0.5	-0.1	-1.0	1	1	2	3.36E-07	0.228828262	5.03E-17
Rv1316c	ogt	0.3	1.0	-1.0	1	2	2	0.028545677	1.00E-17	1.71E-15
Rv3050c	AsnC family transcriptional regulator	0.5	0.0	-1.0	1	1	2	8.64E-05	0.730308847	1.32E-15
Rv2169c	transmembrane protein	0.3	-2.0	-1.0	1	4	2	0.038063954	1.72E-54	2.60E-14
Rv1323	fadA4	0.5	-0.4	-1.0	1	1	2	1.33E-05	3.25E-06	1.78E-18
Rv1218c	tetronasin ABC transporter ATP-bindir	-0.7	2.9	-1.0	2	8	2	7.85E-11	1.61E-153	7.16E-12
Rv1320c	adenylate cyclase	-0.5	1.4	-1.0	1	3	2	0.000292006	6.47E-40	4.34E-14
Rv1167c	transcriptional regulator	-0.5	0.5	-1.0	1	1	2	1.34E-05	0.000994465	9.33E-18
Rv0017c	rodA	-0.2	1.0	-1.0	1	2	2	0.136784757	4.95E-24	2.81E-18
Rv0818	transcriptional regulator	-0.2	-0.3	-1.0	1	1	2	0.134414865	0.014177962	6.93E-20
Rv2043c	pncA	0.3	-1.6	-1.0	1	3	2	0.005930723	7.13E-49	7.83E-16
Rv0337c	aspC	0.1	0.9	-1.0	1	2	2	0.401388584	1.03E-17	3.64E-20
Rv0617	vapC29	-0.5	0.5	-1.0	1	1	2	0.013155862	0.001249608	1.11E-15
Rv1721c	vapB12	-1.9	1.3	-1.0	4	2	2	5.50E-12	5.17E-07	2.89E-06
Rv3616c	espA	0.9	-1.9	-1.0	2	4	2	2.37E-13	6.02E-65	5.93E-15
Rv2342	hypothetical protein	0.1	0.2	-1.0	1	1	2	0.372221964	0.1650681	5.54E-15

Rv2753c	dapA	-0.6	-0.3	-1.0	1	1	2	0.001509878	0.020626712	1.11E-19
Rv2343c	dnaG	-0.5	1.1	-1.0	1	2	2	1.85E-05	1.07E-25	3.02E-21
Rv0477	hypothetical protein	0.5	-1.7	-1.0	1	3	2	0.000208707	5.10E-39	9.36E-16
Rv1644	tsnR	0.0	0.8	-1.0	1	2	2	0.984461443	1.44E-10	4.15E-17
Rv1111c	hypothetical protein	-0.2	1.2	-1.0	1	2	2	0.172011935	2.09E-29	1.43E-16
Rv0019c	fhaB	0.5	0.3	-1.0	1	1	2	2.47E-05	0.020920332	8.62E-19
Rv1487	hypothetical protein	-0.3	0.3	-1.0	1	1	2	0.047905329	0.018024211	8.73E-13
Rv1810	hypothetical protein	0.5	0.1	-1.0	1	1	2	1.15E-05	0.544437097	1.40E-13
Rv2605c	tesB2	1.0	0.4	-1.0	2	1	2	1.26E-13	0.000125312	5.71E-19
Rv1560	vapB11	-0.8	0.3	-1.0	2	1	2	4.70E-05	0.064304211	2.66E-10
Rv2530c	vapC39	-0.4	1.1	-1.0	1	2	2	0.010038501	4.65E-14	9.50E-17
Rv1508c	membrane protein	0.0	1.0	-1.0	1	2	2	0.833260673	8.02E-23	6.21E-19
Rv0966c	hypothetical protein	-0.3	0.3	-1.0	1	1	2	0.028045799	0.022291449	5.30E-16
Rv2292c	hypothetical protein	0.3	-0.4	-1.0	1	1	2	0.042344688	0.004368029	4.58E-12
Rv2134c	hypothetical protein	0.1	-1.1	-1.0	1	2	2	0.349265157	2.06E-25	1.93E-19
Rv3008	hypothetical protein	-0.1	0.0	-1.0	1	1	2	0.535965359	0.738361884	7.46E-19
Rv0165c	mce1R	0.2	1.8	-1.0	1	3	2	0.148250468	4.28E-44	1.33E-20
Rv0651	rpIJ	-1.5	2.0	-1.0	3	4	2	3.60E-14	3.47E-41	3.01E-16
Rv0192	hypothetical protein	-1.2	1.2	-1.0	2	2	2	3.05E-18	4.60E-28	1.12E-21
Rv0608	vapB28	-0.2	0.3	-1.0	1	1	2	0.492981847	0.032192923	1.43E-16
Rv1566c	hypothetical protein	-1.3	3.1	-1.0	2	8	2	2.28E-14	1.16E-145	9.67E-19
Rv0158	transcriptional regulator	0.0	1.0	-1.1	1	2	2	0.897922969	3.00E-13	2.40E-18
Rv1911c	lppC	0.0	-0.2	-1.1	1	1	2	0.746248991	0.033469391	2.07E-19
Rv1352	hypothetical protein	0.4	-1.0	-1.1	1	2	2	0.002121994	8.61E-20	3.34E-19
Rv1859	modC	0.0	-0.5	-1.1	1	1	2	0.745057702	3.84E-05	8.05E-20
Rv2697c	dut	-1.1	0.8	-1.1	2	2	2	1.76E-13	3.69E-09	7.94E-12
Rv3058c	TetR family transcriptional regulator	0.2	0.8	-1.1	1	2	2	0.042086122	6.17E-13	3.04E-20
Rv1498A	hypothetical protein	-0.3	0.5	-1.1	1	1	2	0.083502576	0.00172799	1.71E-12
Rv3146	nuoB	-0.3	1.8	-1.1	1	4	2	0.050240699	7.23E-50	6.41E-20
Rv2792c	resolvase	0.8	0.0	-1.1	2	1	2	2.69E-11	0.789842264	3.19E-19
Rv2259	mscR	0.7	0.7	-1.1	2	2	2	0.064693446	1.27E-12	8.74E-16
Rv3171c	hpx	-1.3	0.7	-1.1	3	2	2	1.84E-13	9.74E-10	1.44E-18
Rv2969c	hypothetical protein	0.5	0.1	-1.1	1	1	2	0.000219834	0.376067099	8.82E-18
Rv3711c	dnaQ	-0.4	0.2	-1.1	1	1	2	0.001675792	0.068687382	2.17E-17
Rv2548	vapC19	-0.8	0.7	-1.1	2	2	2	4.63E-09	2.57E-08	4.37E-17
Rv1149	transposase	0.4	-1.1	-1.1	1	2	2	0.001796812	3.17E-27	3.05E-22
Rv1220c	methyltransferase	-1.0	1.2	-1.1	2	2	2	1.99E-14	7.37E-19	1.26E-16
Rv2868c	gcpE	0.5	-0.4	-1.1	1	1	2	1.24E-06	0.000261166	1.14E-23
Rv1478	peptidoglycan endopeptidase RipB	-0.4	2.0	-1.1	1	4	2	0.017488954	4.07E-55	3.51E-20
Rv0500	proC	-0.6	1.7	-1.1	1	3	2	1.05E-06	1.29E-45	8.65E-19
Rv3009c	gatB	0.8	0.1	-1.1	2	1	2	5.35E-15	0.500517177	6.26E-24
Rv3245c	mtrB	0.3	0.1	-1.1	1	1	2	0.004499742	0.543693144	3.00E-22
Rv2215	dlaT	0.4	0.1	-1.1	1	1	2	0.003667618	0.365613145	5.39E-24
Rv1547	dnaE1	0.7	0.6	-1.1	2	2	2	1.35E-10	3.92E-11	9.87E-25
Rv3193c	transmembrane protein	0.5	0.1	-1.1	1	1	2	7.37E-07	0.255948548	2.95E-23
Rv2882c	frr	0.1	0.2	-1.1	1	1	2	0.54663788	0.059228482	2.55E-24

Rv2752c	ribonuclease J	0.1	0.3	-1.1	1	1	2	0.241449871	0.003787607	1.03E-21
Rv3873	PPE68	0.0	0.4	-1.1	1	1	2	0.938956891	0.000524592	1.69E-18
Rv1418	lprH	0.2	0.6	-1.1	1	2	2	0.141423066	6.09E-08	6.15E-18
Rv3003c	ilvB1	0.3	1.0	-1.1	1	2	2	0.002710193	2.65E-22	1.83E-22
Rv3882c	eccE1	-0.1	1.0	-1.1	1	2	2	0.435351464	3.31E-20	3.96E-23
Rv2590	fadD9	0.5	-0.8	-1.1	1	2	2	4.53E-06	1.72E-13	3.96E-21
Rv3274c	fadE25	0.5	0.6	-1.1	1	2	2	1.30E-06	6.23E-09	4.79E-23
Rv2842c	ribosome maturation factor RimP	0.8	-0.2	-1.1	2	1	2	6.87E-12	0.097499829	7.38E-23
Rv1544	ketoacyl reductase	0.4	0.6	-1.1	1	2	2	0.001747025	9.91E-07	5.61E-20
Rv3864	espE	-0.2	0.3	-1.1	1	1	2	0.16582719	0.017157892	1.02E-19
Rv1693	hypothetical protein	-0.3	1.2	-1.1	1	2	2	0.046745866	1.41E-10	3.13E-12
Rv1701	tyrosine recombinase XerD	0.3	0.5	-1.1	1	1	2	0.013857434	2.12E-05	1.58E-20
Rv2574	hypothetical protein	-0.3	-0.2	-1.1	1	1	2	0.034425643	0.074666722	1.17E-18
Rv1984c	cfp21	0.6	0.8	-1.1	1	2	2	9.88E-07	1.84E-12	6.02E-16
Rv0016c	pbpA	-0.3	1.1	-1.1	1	2	2	0.001603267	5.25E-28	5.00E-23
Rv2069	sigC	-0.5	1.6	-1.1	1	3	2	0.001827637	3.95E-28	1.07E-17
Rv0148	short-chain type dehydrogenase/redu	-0.1	0.5	-1.1	1	1	2	0.70806912	2.65E-05	3.47E-22
Rv3613c	hypothetical protein	0.9	-1.6	-1.1	2	3	2	1.87E-11	2.60E-40	3.84E-16
Rv3117	cysA3	1.5	0.3	-1.1	3	1	2	5.19E-44	0.014375673	8.22E-25
Rv3781	rfbE	0.6	0.8	-1.1	1	2	2	6.78E-07	1.80E-12	1.08E-21
Rv1730c	penicillin-binding protein	0.0	-0.8	-1.1	1	2	2	0.744430666	2.26E-11	5.79E-18
Rv0041	leuS	-0.3	-0.4	-1.1	1	1	2	0.015436566	3.58E-05	1.01E-23
Rv2908c	hypothetical protein	0.5	1.2	-1.1	1	2	2	0.002438734	2.74E-12	1.24E-14
Rv1247c	relB	-0.2	0.8	-1.1	1	2	2	0.206099386	2.26E-06	2.73E-10
Rv3717	hypothetical protein	0.5	1.7	-1.1	1	3	2	0.002124341	5.02E-36	3.00E-19
Rv0133	GCN5-like N-acetyltransferase	-0.4	1.0	-1.1	1	2	2	0.018207377	1.58E-16	2.93E-22
Rv2587c	secD	0.4	0.4	-1.1	1	1	2	0.000195573	7.86E-05	3.75E-25
Rv1264	adenylyl cyclase	-0.8	0.5	-1.1	2	1	2	6.18E-05	8.73E-06	3.09E-22
Rv3821	integral membrane protein	0.4	-1.3	-1.1	1	2	2	0.000638237	2.36E-28	1.19E-23
Rv1260	oxidoreductase	0.1	1.2	-1.1	1	2	2	0.431635125	3.60E-27	1.59E-23
Rv0981	mprA	0.4	-2.0	-1.1	1	4	2	0.002851732	2.69E-65	1.31E-23
Rv0657c	vapB6	-0.1	-0.3	-1.1	1	1	2	0.772866639	0.040103858	5.37E-17
Rv3285	accA3	0.5	-0.6	-1.1	1	2	2	1.36E-05	3.51E-09	3.99E-25
Rv1508A	hypothetical protein	-0.3	0.9	-1.1	1	2	2	0.080400482	1.13E-08	1.36E-16
Rv2130c	mshC	0.4	-0.5	-1.1	1	1	2	9.12E-05	2.69E-06	1.94E-25
Rv0146	S-adenosylmethionine-dependent me	0.2	0.3	-1.1	1	1	2	0.171121011	0.001716896	1.63E-24
Rv0490	senX3	0.9	-1.3	-1.1	2	2	2	2.57E-07	1.85E-43	8.30E-24
Rv0249c	succinate dehydrogenase membrane	-0.7	1.3	-1.1	2	3	2	6.24E-08	1.70E-25	3.19E-14
Rv1891	hypothetical protein	-0.2	-0.2	-1.1	1	1	2	0.042590479	0.121823207	6.34E-22
Rv3458c	rpsD	0.3	0.9	-1.1	1	2	2	0.011790001	1.06E-17	5.46E-21
Rv3641c	fic	-0.1	0.3	-1.1	1	1	2	0.239076299	0.006246428	4.85E-23
Rv1700	NUDIX hydrolase	0.8	0.4	-1.1	2	1	2	1.08E-09	0.003113829	2.69E-20
Rv1099c	glpX	0.3	1.7	-1.1	1	3	2	0.010031572	7.10E-39	1.20E-23
Rv2790c	ltp1	0.4	0.4	-1.1	1	1	2	0.000197791	0.000388308	4.89E-20
Rv2276	cyp121	0.5	2.1	-1.1	1	4	2	3.90E-06	1.12E-68	3.23E-25
Rv3810	pirG	0.0	1.0	-1.1	1	2	2	0.898188852	2.20E-21	5.42E-16

Rv1405c	methyltransferase	0.8	-1.7	-1.1	2	3	2	7.09E-06	7.46E-33	4.48E-13
Rv2247	accD6	-1.0	1.5	-1.1	2	3	2	1.64E-17	3.73E-53	3.07E-24
Rv3282	hypothetical protein	0.1	1.1	-1.1	1	2	2	0.484534168	6.69E-18	2.55E-21
Rv1788	PE18	-1.1	0.8	-1.1	2	2	2	2.00E-09	1.18E-05	1.26E-15
Rv0809	purM	0.3	-0.8	-1.1	1	2	2	0.006614694	1.19E-16	5.21E-26
Rv1314c	cob(I)yrinic acid a,c-diamide adenosy	-0.4	0.9	-1.1	1	2	2	0.000751493	1.51E-12	4.58E-20
Rv3727	oxidoreductase	-0.3	1.7	-1.1	1	3	2	0.002127926	1.05E-57	7.59E-24
Rv0906	hypothetical protein	0.4	0.4	-1.1	1	1	2	0.006670882	0.00164807	1.12E-24
Rv2883c	pyrH	0.1	-0.1	-1.1	1	1	2	0.499772811	0.234140774	1.35E-20
Rv2307D	hypothetical protein	-0.6	-0.2	-1.1	1	1	2	0.006960115	0.305485511	7.89E-14
Rv0815c	cysA2	1.5	0.2	-1.1	3	1	2	9.20E-47	0.028420862	4.54E-27
Rv1178	aminotransferase	0.0	1.0	-1.1	1	2	2	0.803712724	4.92E-20	1.13E-22
Rv2131c	cysQ	0.0	-0.5	-1.1	1	1	2	0.945369933	2.75E-05	1.27E-23
Rv1293	lysA	0.2	0.4	-1.2	1	1	2	0.030028229	2.53E-05	1.67E-22
Rv2068c	blaC	-1.6	1.7	-1.2	3	3	2	9.71E-22	4.99E-44	2.58E-23
Rv2779c	Lrp/AsnC family transcriptional regula	-1.2	1.5	-1.2	2	3	2	7.58E-19	7.57E-25	7.72E-19
Rv3392c	cmaA1	-0.1	1.4	-1.2	1	3	2	0.567938923	7.25E-31	1.18E-23
Rv3045	adhC	0.1	1.2	-1.2	1	2	2	0.383950397	4.25E-27	7.75E-27
Rv0485	transcriptional regulator	0.0	0.1	-1.2	1	1	2	0.895737219	0.357715207	3.18E-24
Rv1637c	hypothetical protein	0.0	0.2	-1.2	1	1	2	0.93679083	0.136663524	3.11E-24
Rv0138	hypothetical protein	-1.4	2.1	-1.2	3	4	2	3.15E-24	4.92E-47	3.67E-21
Rv0856	hypothetical protein	0.7	-0.6	-1.2	2	2	2	6.82E-07	3.28E-08	1.12E-20
Rv0063a	hypothetical protein CDS	-1.1	-1.9	-1.2	2	4	2	4.41E-06	3.35E-30	4.42E-17
Rv1433	hypothetical protein	0.2	1.6	-1.2	1	3	2	0.08921351	8.17E-43	1.71E-24
Rv2468c	hypothetical protein	-0.5	0.6	-1.2	1	2	2	0.003999096	3.15E-06	2.79E-21
Rv3816c	acyltransferase	0.2	-0.6	-1.2	1	1	2	0.120231473	7.47E-08	2.61E-23
Rv0690c	hypothetical protein	0.0	0.3	-1.2	1	1	2	0.985992265	0.001656616	3.76E-26
Rv1485	hemZ	0.4	0.6	-1.2	1	2	2	0.001388573	8.34E-08	1.55E-24
Rv3119	moaE1	0.6	1.0	-1.2	1	2	2	1.23E-05	3.46E-13	2.60E-20
Rv1511	gmdA	-0.5	1.3	-1.2	1	2	2	0.000504623	5.11E-28	3.78E-24
Rv2360c	hypothetical protein	0.3	-0.3	-1.2	1	1	2	0.021146882	0.021255082	1.56E-21
Rv1000c	hypothetical protein	0.3	0.0	-1.2	1	1	2	0.025675431	0.749166166	5.72E-20
Rv0147	aldehyde dehydrogenase	0.5	0.2	-1.2	1	1	2	4.19E-06	0.036547233	2.50E-23
Rv2219	transmembrane protein	0.4	0.1	-1.2	1	1	2	0.000587718	0.494865283	7.18E-23
Rv3587c	membrane protein	0.1	-0.5	-1.2	1	1	2	0.502898618	0.000196069	8.33E-22
Rv0274	hypothetical protein	-0.6	-0.7	-1.2	2	2	2	5.37E-06	3.56E-09	3.12E-22
Rv1778c	hypothetical protein	-0.4	0.5	-1.2	1	1	2	0.003117339	2.88E-05	1.01E-21
Rv3763	lpqH	0.1	0.8	-1.2	1	2	2	0.436403609	4.31E-08	4.74E-17
Rv0475	hbhA	0.0	-0.2	-1.2	1	1	2	0.901323805	0.114840501	1.70E-17
Rv1882c	short-chain type dehydrogenase/redu	0.5	0.4	-1.2	1	1	2	2.88E-05	0.003984765	1.81E-21
Rv1216c	integral membrane protein	-0.1	0.4	-1.2	1	1	2	0.634858596	0.000330882	3.83E-17
Rv1340	rphA	-0.3	0.3	-1.2	1	1	2	0.139638771	0.003825991	7.64E-24
Rv3145	nuoA	-0.4	2.1	-1.2	1	4	2	0.00249348	3.09E-52	3.28E-23
Rv0429c	def	-1.2	2.3	-1.2	2	5	2	3.68E-10	1.75E-46	1.65E-20
Rv3785	hypothetical protein	-0.9	0.8	-1.2	2	2	2	1.41E-09	2.08E-14	1.97E-22
Rv0664	vapB8	-1.6	1.2	-1.2	3	2	2	2.08E-18	1.87E-12	1.55E-11

Rv1797	eccE5	0.6	0.2	-1.2	1	1	2	6.89E-06	0.096700872	2.16E-27
Rv0270	fadD2	-0.3	-1.0	-1.2	1	2	2	0.006655054	4.27E-21	1.74E-26
Rv2474c	hypothetical protein	-0.5	0.5	-1.2	1	1	2	8.48E-05	1.33E-05	2.84E-24
Rv0208c	tRNA (guanine-N(7)-)-methyltransfera	-0.5	2.0	-1.2	1	4	2	0.000158812	5.26E-58	2.53E-24
Rv1506c	hypothetical protein	0.1	1.1	-1.2	1	2	2	0.287629769	5.91E-15	7.00E-23
Rv0997	hypothetical protein	0.5	0.7	-1.2	1	2	2	0.002349881	1.06E-07	1.53E-21
Rv3034c	acetyltransferase	-0.3	-0.1	-1.2	1	1	2	0.03888395	0.443243868	4.95E-25
Rv2519	PE26	0.0	0.2	-1.2	1	1	2	0.764156667	0.119776595	4.02E-29
Rv0315	beta-1,3-glucanase	-0.8	1.3	-1.2	2	2	2	1.19E-10	5.02E-26	1.46E-24
Rv0135c	transcriptional regulator	-0.1	-0.4	-1.2	1	1	2	0.260931634	0.000296495	4.21E-27
Rv1182	papA3	1.0	-1.0	-1.2	2	2	2	1.52E-20	9.15E-20	7.89E-30
Rv2224c	caeA	0.5	-0.6	-1.2	1	1	2	5.41E-06	8.32E-07	4.56E-29
Rv0134	ephF	-0.3	0.3	-1.2	1	1	2	0.012103345	0.007505742	7.78E-25
Rv3684	lyase	0.4	-0.9	-1.2	1	2	2	0.000199783	1.49E-20	5.96E-29
Rv1646	PE17	-0.7	0.3	-1.2	2	1	2	4.72E-08	0.023469162	3.11E-28
Rv1065	hypothetical protein	-2.1	-1.2	-1.2	4	2	2	3.94E-32	1.85E-15	1.50E-24
Rv2406c	hypothetical protein	-2.0	1.3	-1.2	4	2	2	1.32E-29	1.55E-18	8.32E-22
Rv3586	DNA integrity scanning protein DisA	0.0	0.3	-1.2	1	1	2	0.735491534	0.025455924	5.56E-22
Rv3141	fadB4	-0.1	0.1	-1.2	1	1	2	0.485302909	0.459845257	1.27E-27
Rv3582c	ispD	0.1	-0.9	-1.2	1	2	2	0.19208451	1.73E-22	3.40E-31
Rv3099c	hypothetical protein	0.0	-0.1	-1.2	1	1	2	0.689923003	0.270703902	5.69E-28
Rv0346c	ansP2	-0.5	1.8	-1.2	1	4	2	8.21E-07	2.53E-69	1.00E-30
Rv3723	transmembrane protein	0.0	0.1	-1.2	1	1	2	0.687071186	0.20899781	2.59E-26
Rv2329c	narK1	0.7	0.9	-1.2	2	2	2	7.02E-11	7.40E-20	2.79E-26
Rv1923	lipD	0.2	0.2	-1.2	1	1	2	0.088972601	0.064250622	1.68E-27
Rv1981c	nrdF1	0.4	0.2	-1.2	1	1	2	0.00546176	0.020926337	4.26E-23
Rv0734	mapA	0.3	1.4	-1.2	1	3	2	0.038577398	7.25E-29	1.82E-23
Rv3310	sapM	-0.8	1.3	-1.2	2	2	2	8.64E-10	8.23E-28	2.10E-27
Rv2773c	dapB	-0.5	1.1	-1.2	1	2	2	3.87E-06	2.68E-21	1.06E-28
Rv2386a	hypothetical protein CDS	-0.3	0.2	-1.2	1	1	2	0.033264998	0.097218673	6.01E-20
Rv0431	tuberculin-like peptide	-0.4	0.9	-1.2	1	2	2	0.013497398	2.40E-10	6.88E-22
Rv2274c	mazF8	-0.5	1.5	-1.2	1	3	2	0.000982355	2.60E-19	8.69E-20
Rv2132	hypothetical protein	-0.5	-0.4	-1.2	1	1	2	0.000124042	0.00298808	7.41E-18
Rv0014c	pknB	-0.2	1.3	-1.2	1	2	2	0.080408355	8.52E-38	8.58E-28
Rv1171	hypothetical protein	-0.5	0.6	-1.2	1	1	2	2.28E-05	2.13E-05	2.19E-24
Rv0424c	hypothetical protein	1.2	-0.3	-1.3	2	1	2	1.26E-20	0.03660836	6.25E-21
Rv3699	hypothetical protein	0.3	-0.1	-1.3	1	1	2	0.017484196	0.501527209	1.36E-24
Rv2867c	GCN5-like N-acetyltransferase	-0.2	-0.8	-1.3	1	2	2	0.156330746	2.24E-10	4.42E-30
Rv1312	hypothetical protein	0.9	0.4	-1.3	2	1	2	3.97E-12	0.000165746	1.10E-26
Rv2656c	prophage protein	-0.1	-1.0	-1.3	1	2	2	0.617269431	1.48E-16	4.32E-19
Rv3852	hns	0.0	0.2	-1.3	1	1	2	0.902082813	0.109383898	4.99E-23
Rv1481	membrane protein	0.3	0.6	-1.3	1	1	2	0.089783435	2.49E-07	3.78E-28
Rv3547	ddn	0.1	0.5	-1.3	1	1	2	0.250456022	5.94E-05	2.97E-24
Rv2217	lipB	-0.1	-0.6	-1.3	1	1	2	0.835276805	1.71E-05	6.28E-20
Rv0073	glutamine ABC transporter ATP-bindir	0.2	1.8	-1.3	1	4	2	0.052742109	1.70E-61	2.80E-29
Rv1374c	hypothetical protein	-0.1	0.4	-1.3	1	1	2	0.418973267	0.000527299	6.42E-19

Rv1200	integral membrane transport protein	0.6	0.3	-1.3	1	1	2	0.000100587	0.037035545	1.57E-29
Rv0059	hypothetical protein	0.4	1.3	-1.3	1	3	2	0.004473847	5.89E-35	1.37E-23
Rv3148	nuoD	0.2	2.3	-1.3	1	5	2	0.063691276	2.97E-95	1.25E-28
Rv3891c	esxD	-0.2	1.4	-1.3	1	3	2	0.293178731	9.26E-28	3.71E-26
Rv2350c	plcB	-1.2	1.9	-1.3	2	4	2	3.30E-24	1.19E-68	8.89E-32
Rv3863	hypothetical protein	-0.6	0.2	-1.3	1	1	2	9.62E-08	0.057896449	4.55E-33
Rv2359	zur	0.2	-1.0	-1.3	1	2	2	0.110514497	3.99E-18	1.16E-26
Rv3414c	sigD	-0.5	1.5	-1.3	1	3	2	0.000122137	9.14E-37	3.72E-28
Rv3004	cfp6	-0.6	0.6	-1.3	2	2	2	0.00483616	4.69E-05	4.53E-23
Rv2260	hypothetical protein	0.3	0.8	-1.3	1	2	2	0.007195034	1.99E-11	6.84E-27
Rv1720c	vapC12	-0.5	0.7	-1.3	1	2	2	0.00108988	1.29E-07	6.93E-20
Rv3893c	PE36	-1.6	1.0	-1.3	3	2	2	2.84E-12	4.90E-10	2.15E-18
Rv2603c	transcriptional regulator	0.5	0.3	-1.3	1	1	2	1.93E-05	0.002727323	3.68E-28
Rv2585c	lipoprotein	0.0	0.8	-1.3	1	2	2	0.730584454	1.14E-12	2.82E-28
Rv1747	ABC transporter ATP-binding protein/p	0.1	0.5	-1.3	1	1	2	0.231850534	7.36E-08	1.83E-33
Rv1451	protoheme IX farnesyltransferase	0.2	0.0	-1.3	1	1	2	0.132677024	0.81070412	1.11E-31
Rv2817c	CRISPR-associated endonuclease Ca	1.5	0.7	-1.3	3	2	2	1.61E-45	3.44E-12	1.24E-29
Rv1558	hypothetical protein	0.0	1.1	-1.3	1	2	2	0.867913393	1.04E-16	9.43E-26
Rv3266c	rmlD	0.3	-0.4	-1.3	1	1	2	0.01224969	9.72E-05	8.14E-29
Rv2302	hypothetical protein	0.1	0.0	-1.3	1	1	2	0.281817379	0.858332223	7.32E-22
Rv1876	bfrA	1.4	0.5	-1.3	3	1	2	2.64E-20	0.000116001	2.55E-25
Rv2186c	hypothetical protein	-0.5	0.4	-1.3	1	1	2	0.000188184	0.002563909	5.83E-27
Rv3264c	manB	0.0	0.7	-1.3	1	2	2	0.660980728	9.86E-10	2.16E-33
Rv3383c	idsB	-0.4	1.6	-1.3	1	3	2	0.000675752	7.42E-46	5.50E-22
Rv0049	hypothetical protein	-0.1	-0.5	-1.3	1	1	2	0.53587516	0.000183941	1.35E-26
Rv2010	vapC15	0.3	-0.6	-1.3	1	2	2	0.006681762	6.95E-09	7.67E-21
Rv1175c	fadH	-0.6	0.7	-1.3	1	2	2	7.77E-06	1.27E-09	6.30E-35
Rv1598c	hypothetical protein	-0.7	0.7	-1.3	2	2	2	3.39E-07	2.05E-05	4.08E-25
Rv3741c	oxidoreductase	1.0	-0.3	-1.3	2	1	2	2.33E-19	0.010732567	1.65E-27
Rv3312b	hypothetical protein CDS	-0.6	0.8	-1.3	1	2	2	0.000371668	1.38E-06	8.24E-21
Rv1898	hypothetical protein	0.3	-0.7	-1.3	1	2	2	0.031069678	2.51E-09	8.76E-27
Rv0546c	hypothetical protein	-0.1	1.1	-1.3	1	2	2	0.431375123	2.34E-14	3.62E-22
Rv1479	moxR1	0.5	0.4	-1.3	1	1	2	4.24E-06	1.69E-05	4.50E-35
Rv1855c	oxidoreductase	0.4	-0.8	-1.3	1	2	2	0.004940548	3.39E-13	5.46E-29
Rv1844c	gnd1	0.0	0.2	-1.3	1	1	2	0.842269247	0.047776112	2.52E-31
Rv3661	hypothetical protein	0.5	0.5	-1.3	1	1	2	1.51E-05	6.33E-06	5.17E-30
Rv2138	lppL	0.1	0.1	-1.3	1	1	2	0.537452641	0.206381161	2.68E-30
Rv1540	RNA pseudouridine synthase	0.5	-0.3	-1.3	1	1	2	6.63E-05	0.010390303	2.83E-29
Rv3853	rraA	-0.1	-0.2	-1.3	1	1	2	0.671500098	0.100268191	3.98E-28
Rv1847	esterase	-0.9	-0.1	-1.3	2	1	2	0.026066612	0.289744653	1.61E-27
Rv3709c	ask	0.1	0.3	-1.3	1	1	3	0.37784436	0.001468628	1.48E-34
Rv0198c	zmp1	0.6	0.2	-1.3	2	1	3	7.05E-09	0.023513829	9.58E-36
Rv0313	hypothetical protein	-0.3	-1.8	-1.3	1	3	3	0.022497121	1.96E-56	1.64E-25
Rv0558	menH	0.1	-0.3	-1.3	1	1	3	0.332807354	0.008422387	1.17E-30
Rv0562	grcC1	0.0	0.5	-1.3	1	1	3	0.905204258	1.72E-05	2.91E-30
Rv2358	smtB	-0.8	-0.5	-1.3	2	1	3	5.59E-10	2.50E-05	5.08E-28

Rv0935	pstC1	-0.2	0.9	-1.3	1	2	3	0.189987631	1.59E-17	2.87E-27
Rv2703	sigA	0.7	-0.2	-1.3	2	1	3	1.24E-11	0.030309476	2.69E-34
Rv2248	hypothetical protein	-0.6	1.7	-1.3	1	3	3	2.26E-06	2.29E-44	1.17E-29
Rv2218	lipA	0.3	0.0	-1.3	1	1	3	0.011115444	0.788244727	4.80E-33
Rv3614c	espD	0.4	-1.9	-1.3	1	4	3	0.000669017	4.79E-78	8.88E-32
Rv2272	transmembrane protein	0.9	1.0	-1.3	2	2	3	2.60E-12	1.82E-13	1.03E-27
Rv2136c	undecaprenyl-diphosphatase	0.3	-0.5	-1.3	1	1	3	0.019410967	6.12E-06	5.58E-31
Rv2517c	hypothetical protein	-1.5	0.1	-1.3	3	1	3	4.28E-33	0.626211669	3.28E-31
Rv1779c	integral membrane protein	0.3	0.4	-1.3	1	1	3	0.004960776	0.001089133	1.75E-33
Rv1398c	vapB10	-1.7	-1.1	-1.4	3	2	3	1.53E-13	0.00062731	3.85E-30
Rv2920c	amt	0.4	-1.6	-1.4	1	3	3	0.022069784	1.28E-62	6.16E-36
Rv2428	ahpC	1.8	0.5	-1.4	3	1	3	4.93E-51	2.61E-06	9.96E-22
Rv0002	dnaN	0.2	1.3	-1.4	1	3	3	0.044285362	5.61E-39	9.69E-37
Rv3001c	ilvC	0.8	0.9	-1.4	2	2	3	1.90E-12	9.71E-17	7.97E-34
Rv3140	fadE23	0.8	0.0	-1.4	2	1	3	2.80E-11	0.96641796	2.04E-27
Rv0058	dnaB	1.0	0.9	-1.4	2	2	3	1.08E-19	3.13E-18	3.57E-34
Rv0695	mycofactocin system creatinine amid	-0.2	1.3	-1.4	1	2	3	0.175100795	4.01E-32	2.53E-32
Rv3780	hypothetical protein	0.1	0.8	-1.4	1	2	3	0.301674861	1.43E-10	2.03E-31
Rv1098c	fum	0.3	1.9	-1.4	1	4	3	0.003381031	1.61E-65	3.31E-35
Rv1880c	cyp140	0.7	-0.5	-1.4	2	1	3	9.43E-10	3.24E-07	1.39E-36
Rv1476	membrane protein	-0.1	1.2	-1.4	1	2	3	0.661444288	1.42E-19	2.20E-31
Rv1863c	integral membrane protein	0.1	0.8	-1.4	1	2	3	0.43986795	1.86E-14	1.15E-31
Rv2103c	vapC37	0.1	0.5	-1.4	1	1	3	0.436833009	5.17E-05	1.63E-27
Rv2148c	hypothetical protein	0.3	0.4	-1.4	1	1	3	0.019430906	0.001001094	7.75E-29
Rv3523	ltp3	-1.2	0.7	-1.4	2	2	3	9.84E-17	1.62E-08	3.41E-25
Rv3230c	stearoyl-CoA 9-desaturase electron tr	-2.6	0.5	-1.4	6	1	3	1.34E-69	0.000269625	1.97E-25
Rv3843c	transmembrane protein	0.6	-1.0	-1.4	1	2	3	2.63E-07	6.96E-26	1.05E-36
Rv1915	aceAa	0.5	-0.5	-1.4	1	1	3	3.39E-06	3.29E-06	2.51E-35
Rv2129c	oxidoreductase	0.6	0.2	-1.4	2	1	3	1.55E-07	0.0893422	1.56E-28
Rv2361c	decaprenyl diphosphate synthase	0.2	0.6	-1.4	1	2	3	0.073730815	2.93E-08	7.60E-35
Rv0423c	thiC	0.1	0.5	-1.4	1	1	3	0.201895406	2.80E-06	1.62E-33
Rv0799c	hypothetical protein	-0.1	1.6	-1.4	1	3	3	0.318134688	3.34E-46	4.17E-35
Rv1856c	oxidoreductase	-0.8	2.1	-1.4	2	4	3	1.21E-08	8.31E-61	2.50E-25
Rv0050	ponA1	0.7	0.1	-1.4	2	1	3	6.28E-11	0.184332581	1.67E-39
Rv0524	hemL	-0.3	0.6	-1.4	1	2	3	0.045850988	4.68E-09	1.71E-34
Rv0931c	pknD	0.4	-0.1	-1.4	1	1	3	0.00013508	0.478836683	5.55E-39
Rv1979c	permease	0.8	0.2	-1.4	2	1	3	1.03E-11	0.129082955	2.87E-33
Rv1899c	lppD	-0.7	-0.1	-1.4	2	1	3	3.56E-07	0.317822872	2.21E-22
Rv2606c	snzP	-0.1	0.2	-1.4	1	1	3	0.664901394	0.114922452	4.22E-35
Rv1919c	hypothetical protein	0.3	1.0	-1.4	1	2	3	0.102332236	3.25E-17	1.15E-29
Rv2288	hypothetical protein	-0.3	1.2	-1.4	1	2	3	0.012431691	3.68E-21	4.42E-30
Rv0757	phoP	0.1	-0.6	-1.4	1	2	3	0.357989329	3.66E-09	4.28E-35
Rv1698	mctB	0.1	2.0	-1.4	1	4	3	0.577140774	9.62E-62	3.75E-35
Rv1152	transcriptional regulator	-0.4	-1.3	-1.4	1	3	3	0.002203346	9.63E-36	6.49E-26
Rv3051c	nrdE	0.3	0.0	-1.4	1	1	3	0.00713967	0.904691955	3.49E-18
Rv3053c	nrdH	-0.4	-0.1	-1.4	1	1	3	0.016657426	0.263404494	6.07E-21

Rv0891c	hypothetical protein	-1.3	2.1	-1.4	3	4	3	5.44E-27	1.28E-80	8.10E-34
Rv1796	mycP5	0.1	-0.1	-1.4	1	1	3	0.232083092	0.226679991	1.03E-36
Rv1409	ribG	-0.1	0.2	-1.4	1	1	3	0.478268545	0.027332742	2.54E-35
Rv2427c	proA	-0.2	1.0	-1.4	1	2	3	0.164200354	2.14E-17	2.77E-34
Rv0047c	hypothetical protein	-1.7	-0.2	-1.4	3	1	3	2.01E-42	0.119616325	6.29E-27
Rv2985	mutT1	-0.1	1.3	-1.4	1	2	3	0.582082285	3.83E-32	3.42E-38
Rv3824c	papA1	0.1	0.0	-1.4	1	1	3	0.238557205	0.747889894	7.42E-36
Rv3697c	vapC48	-0.4	0.2	-1.4	1	1	3	0.002188141	0.204607015	1.83E-28
Rv2142c	parE2	0.5	-0.8	-1.4	1	2	3	0.000266813	3.39E-10	6.53E-21
Rv0544c	transmembrane protein	-0.2	1.5	-1.4	1	3	3	0.120723368	5.19E-20	7.59E-25
Rv2256c	hypothetical protein	0.6	-1.2	-1.4	2	2	3	3.41E-05	1.06E-20	5.49E-30
Rv1982c	vapC36	0.5	-0.3	-1.4	1	1	3	7.18E-05	0.008087861	2.21E-29
Rv2081c	transmembrane protein	-0.3	0.8	-1.4	1	2	3	0.026439179	5.07E-13	4.58E-34
Rv2709	transmembrane protein	-1.0	0.0	-1.4	2	1	3	4.98E-15	0.81726348	5.22E-34
Rv2534c	efp	-0.3	1.0	-1.4	1	2	3	0.125858882	8.24E-14	1.99E-35
Rv1097c	hypothetical protein	0.1	1.5	-1.4	1	3	3	0.367336839	1.61E-36	1.78E-36
Rv0506	mmpS2	0.0	1.8	-1.4	1	4	3	0.955109833	2.21E-46	1.38E-33
Rv0038	hypothetical protein	0.1	-0.1	-1.4	1	1	3	0.668623513	0.530000359	1.07E-33
Rv2925c	rnc	0.7	-0.5	-1.4	2	1	3	6.95E-11	2.18E-05	1.38E-39
Rv3096	hypothetical protein	-0.3	2.0	-1.4	1	4	3	0.009547707	5.09E-69	6.43E-40
Rv2475c	hypothetical protein	0.3	0.6	-1.4	1	2	3	0.004151642	1.32E-06	4.72E-33
Rv2902c	rnhB	0.8	0.2	-1.4	2	1	3	7.25E-12	0.160802308	5.90E-32
Rv0281	S-adenosylmethionine-dependent me	-0.3	0.3	-1.4	1	1	3	0.054826936	0.007494618	2.81E-38
Rv3846	sodA	0.8	0.3	-1.4	2	1	3	2.51E-07	0.017462135	6.79E-38
Rv1419	hypothetical protein	0.9	-1.1	-1.4	2	2	3	6.73E-12	4.26E-28	5.28E-39
Rv1108c	xseA	0.1	1.3	-1.4	1	3	3	0.262970521	5.91E-37	3.88E-40
Rv0309	hypothetical protein	0.1	0.3	-1.4	1	1	3	0.240914175	0.00263041	1.02E-38
Rv3443c	rplM	0.2	1.7	-1.4	1	3	3	0.188861587	1.28E-35	5.74E-30
Rv3615c	espC	1.5	-2.6	-1.4	3	6	3	2.12E-32	1.08E-134	8.29E-24
Rv2665	hypothetical protein	-1.3	0.3	-1.5	2	1	3	8.21E-19	0.020037658	1.40E-23
Rv0466	hypothetical protein	-0.2	1.7	-1.5	1	3	3	0.110026949	7.98E-45	7.90E-31
Rv2144c	transmembrane protein	0.4	0.4	-1.5	1	1	3	0.001705344	0.000711732	7.12E-25
Rv1881c	lppE	1.0	-0.5	-1.5	2	1	3	1.48E-18	1.53E-06	6.84E-35
Rv0691c	mycofactocin biosynthesis transcripti	-0.3	0.4	-1.5	1	1	3	0.011538045	0.001031807	3.02E-30
Rv0652	rplL	-0.3	2.2	-1.5	1	5	3	0.105929103	6.93E-42	6.25E-27
Rv2445c	ndkA	0.7	0.5	-1.5	2	1	3	4.41E-08	0.000110218	1.74E-28
Rv1192	hypothetical protein	-0.1	-1.0	-1.5	1	2	3	0.60382016	8.35E-17	5.78E-38
Rv3694c	transmembrane protein	0.0	-0.2	-1.5	1	1	3	0.868409047	0.129043102	3.95E-43
Rv3738c	PPE66	-1.4	1.2	-1.5	3	2	3	4.56E-22	3.42E-25	2.49E-39
Rv2594c	ruvC	0.3	1.9	-1.5	1	4	3	0.007863194	4.23E-61	4.92E-39
Rv3153	nuoI	0.2	1.1	-1.5	1	2	3	0.041126881	1.32E-21	1.94E-34
Rv0798c	cfp29	0.2	0.8	-1.5	1	2	3	0.17399836	1.26E-13	3.73E-38
Rv3404c	hypothetical protein	-0.7	0.8	-1.5	2	2	3	3.65E-09	4.89E-13	1.60E-38
Rv3007c	oxidoreductase	-0.7	0.7	-1.5	2	2	3	4.62E-09	2.71E-09	8.49E-35
Rv0950c	hypothetical protein	-0.2	0.8	-1.5	1	2	3	0.063943777	3.69E-09	2.61E-39
Rv3127	hypothetical protein	1.6	-1.2	-1.5	3	2	3	7.58E-51	3.22E-35	5.82E-36

Rv1092c	coaA	-0.5	1.4	-1.5	1	3	3	1.78E-05	2.98E-40	3.16E-40
Rv2808	hypothetical protein	0.9	0.4	-1.5	2	1	3	9.27E-10	0.001761291	5.60E-28
Rv1298	rpmE	-0.6	1.0	-1.5	2	2	3	0.000740407	7.91E-21	1.67E-27
Rv3870	eccCa1	-0.3	0.1	-1.5	1	1	3	0.030404781	0.144587908	8.87E-33
Rv1043c	hypothetical protein	-0.2	0.2	-1.5	1	1	3	0.173866574	0.142776252	3.86E-39
Rv1212c	glgA	0.5	-0.6	-1.5	1	2	3	4.82E-06	2.20E-08	1.96E-37
Rv3241c	hypothetical protein	-0.1	0.7	-1.5	1	2	3	0.344559769	3.05E-08	1.33E-33
Rv2737c	recA	0.3	1.8	-1.5	1	3	3	0.004523619	8.94E-71	1.02E-36
Rv2909c	rpsP	-0.2	1.7	-1.5	1	3	3	0.167839185	1.81E-34	1.41E-33
Rv2971	oxidoreductase	0.5	0.7	-1.5	1	2	3	1.45E-06	1.12E-11	1.88E-41
Rv3720	fatty acid synthase	0.6	-0.2	-1.5	1	1	3	1.16E-07	0.023550939	1.01E-43
Rv2780	ald	-0.5	-0.6	-1.5	1	1	3	1.81E-05	1.33E-08	5.05E-44
Rv0605	IS1536 family serine type transposase	0.9	0.0	-1.5	2	1	3	1.08E-09	0.755746033	1.28E-32
Rv0868c	moaD2	0.0	1.3	-1.5	1	2	3	0.847455222	3.55E-19	2.36E-26
Rv3427c	transposase	-0.4	0.4	-1.5	1	1	3	0.000319212	0.000253637	2.50E-39
Rv1096	glycosyl hydrolase	0.1	0.7	-1.5	1	2	3	0.400124427	2.20E-09	1.57E-38
Rv0302	transcriptional regulator	-0.4	0.6	-1.5	1	2	3	0.00230867	5.14E-09	2.88E-42
Rv1837c	glcB	0.6	0.6	-1.5	1	2	3	3.73E-07	1.12E-10	7.83E-42
Rv1271c	hypothetical protein	0.2	1.1	-1.5	1	2	3	0.174961547	3.36E-19	1.28E-36
Rv2878c	mpt53	0.4	0.2	-1.5	1	1	3	0.000436065	0.061069462	3.51E-37
Rv0919	GCN5-like N-acetyltransferase	0.7	0.3	-1.5	2	1	3	4.24E-09	0.005558487	1.17E-38
Rv2674	msrB	-0.5	0.7	-1.5	1	2	3	0.000129668	2.99E-09	3.18E-35
Rv0711	atsA	-0.5	0.6	-1.5	1	1	3	3.71E-06	1.63E-08	7.36E-45
Rv1615	membrane protein	-0.3	-0.3	-1.5	1	1	3	0.104774642	0.345826007	3.67E-27
Rv0997a	hypothetical protein CDS	0.4	-0.1	-1.5	1	1	3	0.003886717	0.570135779	3.47E-36
Rv1821	secA2	0.1	0.6	-1.5	1	1	3	0.428896805	1.31E-09	5.58E-46
Rv0545c	pitA	0.0	1.5	-1.5	1	3	3	0.887563074	7.11E-47	6.41E-46
Rv3681c	whiB4	0.1	-0.4	-1.5	1	1	3	0.28087055	0.000174075	3.29E-34
Rv2992c	gltS	-0.4	2.3	-1.5	1	5	3	0.000253874	5.54E-99	4.19E-45
Rv1738	hypothetical protein	1.6	-1.7	-1.5	3	3	3	1.38E-35	7.01E-56	1.11E-31
Rv1903	membrane protein	-0.5	1.5	-1.5	1	3	3	0.000179967	2.99E-37	1.85E-33
Rv0646c	lipG	-1.0	2.1	-1.5	2	4	3	6.10E-09	1.12E-63	1.29E-41
Rv3524	membrane protein	-1.0	1.1	-1.5	2	2	3	1.70E-16	6.62E-25	2.09E-42
Rv2960c	hypothetical protein	0.7	1.1	-1.5	2	2	3	9.24E-06	9.50E-13	8.50E-29
Rv2990c	hypothetical protein	-1.2	1.3	-1.5	2	2	3	5.99E-25	1.70E-25	4.41E-34
Rv1144	oxidoreductase	0.4	-0.3	-1.5	1	1	3	0.051180051	0.019162006	7.73E-37
Rv3043c	ctaD	-0.4	1.6	-1.5	1	3	3	0.000533244	5.44E-55	7.46E-49
Rv2513	hypothetical protein	0.3	0.0	-1.5	1	1	3	0.019280013	0.958706288	1.07E-40
Rv2367c	endoribonuclease	-0.3	0.0	-1.5	1	1	3	0.069871138	0.964163643	2.54E-36
Rv2926c	hypothetical protein	0.6	-0.8	-1.5	1	2	3	1.52E-07	1.18E-10	4.74E-45
Rv2586c	secF	-0.7	0.1	-1.5	2	1	3	5.15E-08	0.261273488	9.96E-42
Rv2783c	gpsI	0.9	0.6	-1.5	2	2	3	1.37E-16	2.29E-10	7.02E-49
Rv3116	moeB2	0.5	0.7	-1.6	1	2	3	3.28E-05	4.16E-10	1.49E-44
Rv0930	pstA1	0.5	-0.6	-1.6	1	2	3	9.58E-06	6.56E-11	2.08E-39
Rv0857	hypothetical protein	-0.5	-0.4	-1.6	1	1	3	0.000737745	0.002262577	1.44E-37
Rv0749A	hypothetical protein	-1.1	0.5	-1.6	2	1	3	2.65E-09	0.000647184	2.21E-24

Rv2951c	phthiodiolone/phenolphthiodiolone d	0.0	1.8	-1.6	1	3	3	0.77775949	4.80E-58	2.54E-44
Rv3783	rfbD	-0.2	1.3	-1.6	1	2	3	0.040872582	9.65E-26	3.98E-40
Rv3719	hypothetical protein	0.6	0.2	-1.6	2	1	3	5.11E-07	0.019305693	1.48E-46
Rv0979A	rpmF	-1.1	1.9	-1.6	2	4	3	6.26E-06	1.00E-19	4.15E-22
Rv0178	Mce associated membrane protein	0.3	1.2	-1.6	1	2	3	0.012383175	1.36E-27	2.35E-48
Rv1886c	fbpB	0.3	1.2	-1.6	1	2	3	0.011426069	1.31E-24	6.32E-43
Rv0530A	hypothetical protein	-1.1	-0.1	-1.6	2	1	3	7.51E-15	0.62991876	1.35E-35
Rv0644c	mmaA2	-0.5	1.6	-1.6	1	3	3	0.000110863	7.10E-51	1.90E-41
Rv3871	eccCb1	-0.1	-0.2	-1.6	1	1	3	0.222060208	0.057768965	3.24E-49
Rv3683	hypothetical protein	-0.3	0.7	-1.6	1	2	3	0.003847567	4.79E-12	2.52E-46
Rv3878	espJ	-0.6	0.4	-1.6	2	1	3	2.53E-08	0.000438585	3.70E-47
Rv0611c	hypothetical protein	-0.3	-1.1	-1.6	1	2	3	0.006097818	7.45E-20	2.18E-26
Rv3872	PE35	-0.2	0.2	-1.6	1	1	3	0.129354299	0.160579609	6.92E-45
Rv0028	hypothetical protein	0.6	-1.1	-1.6	2	2	3	5.94E-08	3.66E-21	1.51E-38
Rv0179c	lprO	0.1	1.7	-1.6	1	3	3	0.552338154	6.12E-56	1.41E-38
Rv0364	transmembrane protein	0.1	2.0	-1.6	1	4	3	0.407191383	3.80E-67	2.08E-44
Rv0556	transmembrane protein	0.1	1.0	-1.6	1	2	3	0.614839904	8.37E-16	7.85E-39
Rv0844c	narL	-0.7	1.5	-1.6	2	3	3	9.04E-07	2.45E-26	4.32E-35
Rv2472	hypothetical protein	-0.6	0.7	-1.6	2	2	3	6.73E-07	1.18E-08	3.08E-40
Rv0883c	hypothetical protein	-1.0	1.4	-1.6	2	3	3	1.18E-18	8.13E-35	1.51E-41
Rv1093	glyA1	-0.3	0.0	-1.6	1	1	3	0.007069099	0.973869507	2.20E-49
Rv0830	S-adenosylmethionine-dependent me	0.6	0.8	-1.6	2	2	3	1.06E-06	4.54E-13	1.25E-38
Rv2809	hypothetical protein	1.1	0.2	-1.6	2	1	3	1.04E-09	0.084541267	1.38E-49
Rv0287	esxG	0.0	0.7	-1.6	1	2	3	0.884438676	3.41E-07	4.29E-40
Rv3749c	hypothetical protein	0.9	0.5	-1.6	2	1	3	1.32E-11	6.39E-06	1.27E-41
Rv0129c	fbpC	0.1	2.3	-1.6	1	5	3	0.291650908	2.82E-111	2.14E-51
Rv3198A	glutaredoxin protein	0.3	1.2	-1.6	1	2	3	0.011024764	2.08E-19	4.29E-28
Rv1133c	metE	0.4	0.9	-1.6	1	2	3	0.00222158	1.49E-21	6.48E-54
Rv2240c	hypothetical protein	0.3	1.4	-1.6	1	3	3	0.032572625	2.15E-21	3.30E-34
Rv0686	membrane protein	0.2	0.5	-1.6	1	1	3	0.177407329	3.81E-05	8.76E-44
Rv0749	vapC31	1.0	-0.8	-1.6	2	2	3	1.26E-13	3.12E-14	1.63E-44
Rv2146c	transmembrane protein	0.4	-1.0	-1.6	1	2	3	0.000200397	5.65E-14	5.46E-44
Rv1734c	hypothetical protein	0.7	-2.1	-1.6	2	4	3	3.95E-07	1.11E-67	1.05E-31
Rv3624c	hpt	-0.2	0.8	-1.6	1	2	3	0.133973099	1.85E-11	7.71E-39
Rv1732c	hypothetical protein	0.3	0.0	-1.6	1	1	3	0.019141343	0.873260675	3.07E-43
Rv2451	hypothetical protein	0.0	0.6	-1.6	1	1	3	0.738651821	0.000204982	1.45E-35
Rv1410c	aminoglycosides/tetracycline-transpo	0.0	0.2	-1.6	1	1	3	0.743107155	0.0399358	1.15E-54
Rv2468A	hypothetical protein	-0.5	1.0	-1.6	1	2	3	0.001657322	3.11E-15	1.22E-37
Rv1440	secG	0.3	1.5	-1.6	1	3	3	0.032120543	5.07E-28	1.91E-35
Rv3002c	ilvN	0.7	1.2	-1.6	2	2	3	2.08E-08	3.40E-20	8.87E-40
Rv3485c	short-chain type dehydrogenase/redu	-0.1	1.6	-1.6	1	3	3	0.180788927	9.78E-54	5.33E-51
Rv2336	hypothetical protein	0.2	2.0	-1.6	1	4	3	0.137150866	6.50E-65	3.56E-46
Rv3847	hypothetical protein	0.1	0.0	-1.6	1	1	3	0.280314736	0.718665672	1.19E-42
Rv0174	mce1F	0.4	1.4	-1.6	1	3	3	0.000354768	9.53E-51	4.15E-47
Rv0243	fadA2	-0.7	1.5	-1.6	2	3	3	1.10E-07	3.66E-49	4.15E-54
Rv3074	hypothetical protein	-0.1	0.7	-1.6	1	2	3	0.777888319	2.65E-05	4.26E-24

Rv3869	eccB1	-0.2	0.2	-1.7	1	1	3	0.023840997	0.057911942	3.26E-53
Rv1339	hypothetical protein	-0.5	0.3	-1.7	1	1	3	0.000374487	0.015802018	1.81E-48
Rv1477	ripA	-0.6	2.5	-1.7	1	6	3	0.000477505	1.81E-99	3.69E-54
Rv0586	mce2R	-1.2	1.0	-1.7	2	2	3	6.71E-21	2.15E-18	9.29E-47
Rv0054	ssb	0.0	0.8	-1.7	1	2	3	0.950768032	4.25E-12	5.05E-42
Rv0463	membrane protein	0.7	-0.5	-1.7	2	1	3	4.97E-07	1.93E-05	3.32E-43
Rv3677c	beta lactamase	0.9	0.4	-1.7	2	1	3	2.57E-16	0.000138331	7.67E-47
Rv0072	glutamine ABC transporter permease	0.3	1.7	-1.7	1	3	3	0.00429872	1.56E-59	3.53E-53
Rv0443	hypothetical protein	0.0	-0.2	-1.7	1	1	3	0.982867758	0.148116323	2.92E-50
Rv2237A	hypothetical protein	0.4	0.4	-1.7	1	1	3	0.01047614	0.003866082	3.03E-35
Rv3716c	hypothetical protein	0.7	1.1	-1.7	2	2	3	1.64E-07	1.31E-15	1.37E-40
Rv2929	hypothetical protein	0.3	1.6	-1.7	1	3	3	0.044992858	1.25E-28	5.44E-37
Rv1838c	vapC13	-0.9	0.1	-1.7	2	1	3	3.48E-08	0.400992355	9.86E-25
Rv0954	transmembrane protein	-0.7	1.0	-1.7	2	2	3	1.80E-07	1.40E-19	1.16E-49
Rv3865	espF	0.0	0.0	-1.7	1	1	3	0.908478864	0.985515786	1.32E-33
Rv0338c	iron-sulfur-binding reductase	0.0	0.8	-1.7	1	2	3	0.831419475	1.23E-18	3.69E-59
Rv2127	ansP1	0.5	0.9	-1.7	1	2	3	4.81E-05	6.99E-21	1.64E-56
Rv2112c	dop	-0.1	0.9	-1.7	1	2	3	0.223092508	7.96E-14	2.54E-54
Rv1703c	methyltransferase	0.4	0.0	-1.7	1	1	3	0.000321337	0.693065786	2.45E-39
Rv3774	echA21	-0.8	1.4	-1.7	2	3	3	1.35E-05	6.51E-35	3.94E-49
Rv1110	lytB2	0.5	0.0	-1.7	1	1	3	1.39E-06	0.96330921	2.90E-55
Rv2881c	A	0.2	1.0	-1.7	1	2	3	0.048890991	3.30E-24	9.32E-53
Rv0211	pckA	1.3	-0.9	-1.7	3	2	3	1.67E-37	7.47E-21	2.95E-55
Rv1948c	hypothetical protein	-0.2	0.8	-1.7	1	2	3	0.158486223	1.12E-08	2.82E-26
Rv3281	accE5	1.0	0.7	-1.7	2	2	3	0.010041107	3.31E-09	3.14E-47
Rv1080c	greA	0.4	1.0	-1.7	1	2	3	0.00178148	3.12E-23	9.91E-27
Rv3098A	PemK-like protein	-0.2	-0.3	-1.7	1	1	3	0.229870858	0.010987618	3.08E-37
Rv0820	phoT	0.7	-0.6	-1.7	2	2	3	6.55E-08	4.78E-09	1.92E-50
Rv0137c	msrA	-0.8	0.4	-1.7	2	1	3	2.66E-11	3.60E-05	3.51E-45
Rv2182c	1-acylglycerol-3-phosphate O-acyltra	0.0	-0.7	-1.7	1	2	3	0.939560959	4.42E-11	7.94E-51
Rv2432c	hypothetical protein	-0.2	0.0	-1.7	1	1	3	0.378863367	0.919775474	6.05E-43
Rv0937c	mku	0.2	0.5	-1.7	1	1	3	0.093398804	2.32E-06	4.81E-52
Rv0055	rpsR1	0.4	1.2	-1.7	1	2	3	0.012079251	6.87E-15	2.95E-36
Rv2507	hypothetical protein	-0.8	0.5	-1.7	2	1	3	1.04E-14	1.26E-05	2.30E-36
Rv0616A	vapB29	-0.5	0.1	-1.7	1	1	3	0.001624463	0.54411717	4.52E-44
Rv3257c	pmmA	-0.3	0.7	-1.7	1	2	3	0.014276658	7.52E-12	6.55E-53
Rv1486c	hypothetical protein	-0.1	-0.2	-1.7	1	1	3	0.381979905	0.145284336	1.38E-50
Rv3208A	TB9.4	-0.1	0.6	-1.7	1	1	3	0.636404908	1.02E-05	2.22E-37
Rv2956	hypothetical protein	0.7	1.4	-1.7	2	3	3	2.67E-07	3.29E-21	1.06E-36
Rv3277	transmembrane protein	-0.3	1.5	-1.7	1	3	3	0.031190272	6.06E-33	8.46E-47
Rv3029c	fixA	0.5	1.3	-1.7	1	3	3	1.90E-06	6.64E-35	1.36E-50
Rv0730	GCN5-like N-acetyltransferase	-0.9	1.0	-1.7	2	2	3	1.50E-14	4.03E-20	4.48E-49
Rv0061c	hypothetical protein	0.8	-1.1	-1.7	2	2	3	3.60E-13	5.49E-20	2.92E-40
Rv3459c	rpsK	0.4	1.2	-1.7	1	2	3	0.000723785	2.51E-24	3.21E-50
Rv0460	hypothetical protein	-0.5	1.1	-1.7	1	2	3	0.001524622	1.85E-15	2.49E-42
Rv0236A	hypothetical protein	-1.9	2.7	-1.7	4	6	3	9.56E-15	7.78E-95	3.05E-43

Rv3465	rmlC	0.0	1.6	-1.7	1	3	3	0.82709576	5.43E-30	2.96E-47
Rv3138	pflA	-0.4	-0.2	-1.7	1	1	3	0.000358234	0.120836183	2.92E-51
Rv3917c	parB	-0.3	0.2	-1.7	1	1	3	0.014677968	0.052259015	8.90E-62
Rv2530A	vapB39	-0.3	0.8	-1.7	1	2	3	0.037146186	3.71E-08	6.40E-38
Rv0013	trpG	-0.6	1.0	-1.7	1	2	3	0.000240565	1.32E-18	4.08E-44
Rv1507c	hypothetical protein	-0.2	2.1	-1.7	1	4	3	0.227394466	8.67E-62	4.53E-41
Rv2940c	mas	-0.1	1.1	-1.7	1	2	3	0.409832617	2.62E-32	2.15E-64
Rv3155	nuoK	0.0	2.0	-1.8	1	4	3	0.778856728	8.29E-44	2.38E-35
Rv2275	cyclo(L-tyrosyl-L-tyrosyl) synthase	0.1	2.1	-1.8	1	4	3	0.43585316	2.30E-71	7.43E-54
Rv0948c	chorismate mutase	-0.4	1.2	-1.8	1	2	3	0.005164065	3.40E-15	6.05E-39
Rv0057	hypothetical protein	0.0	2.1	-1.8	1	4	3	0.986752347	6.87E-47	2.56E-50
Rv3130c	tgs1	1.8	-0.3	-1.8	4	1	3	1.81E-36	0.012826932	4.46E-46
Rv0006	gyrA	0.0	0.3	-1.8	1	1	3	0.762416447	0.001177261	1.11E-55
Rv1438	tpi	0.1	1.9	-1.8	1	4	3	0.378628894	1.83E-64	4.67E-56
Rv0474	HTH-type transcriptional regulator	0.0	-0.9	-1.8	1	2	3	0.950655817	6.66E-17	3.32E-50
Rv0934	pstS1	-0.2	1.9	-1.8	1	4	3	0.034030281	5.83E-66	5.15E-54
Rv3682	ponA2	-0.2	1.5	-1.8	1	3	3	0.087561173	1.33E-53	1.93E-56
Rv0145	S-adenosylmethionine-dependent me	-0.4	0.8	-1.8	1	2	3	0.002275834	3.38E-12	3.01E-59
Rv0903c	prrA	-0.8	-0.4	-1.8	2	1	3	1.24E-10	2.05E-05	2.55E-58
Rv3210c	hypothetical protein	-0.3	1.8	-1.8	1	4	3	0.072124558	1.28E-56	4.12E-57
Rv0500A	DNA-binding protein	0.0	0.3	-1.8	1	1	3	0.833962405	0.088720169	7.45E-42
Rv2629	hypothetical protein	0.2	-0.3	-1.8	1	1	3	0.114563506	0.001894622	8.34E-54
Rv3722c	hypothetical protein	0.7	0.4	-1.8	2	1	3	5.44E-08	0.000117924	4.84E-60
Rv3687c	rsfB	-0.2	1.8	-1.8	1	3	3	0.14709038	2.24E-52	8.53E-52
Rv1311	atpC	0.5	0.4	-1.8	1	1	3	0.00031763	0.002063111	1.85E-49
Rv3688c	hypothetical protein	-0.3	1.5	-1.8	1	3	3	0.015957979	5.06E-31	3.25E-49
Rv3456c	rplQ	0.6	0.4	-1.8	2	1	3	5.53E-08	1.87E-05	1.44E-62
Rv3098c	hypothetical protein	-0.5	0.3	-1.8	1	1	3	2.30E-05	0.011898847	1.05E-57
Rv1321	endonuclease NucS	0.3	0.7	-1.8	1	2	4	0.029632424	1.89E-10	8.36E-54
Rv3593	lpqF	-0.4	1.5	-1.8	1	3	4	0.00060965	1.17E-50	4.98E-61
Rv2077c	transmembrane protein	-0.7	-1.5	-1.8	2	3	4	3.09E-09	2.65E-36	2.90E-61
Rv2509	short-chain type dehydrogenase/redu	-0.8	1.4	-1.8	2	3	4	6.28E-11	9.50E-31	9.95E-52
Rv1500	glycosyltransferase	-0.1	1.0	-1.8	1	2	4	0.563637615	5.51E-15	1.93E-37
Rv1904	hypothetical protein	-0.1	0.7	-1.8	1	2	4	0.415285054	5.84E-08	6.07E-51
Rv0933	pstB	-1.0	2.2	-1.8	2	5	4	3.97E-18	1.63E-79	7.46E-57
Rv1408	rpe	-1.1	1.3	-1.8	2	2	4	7.54E-21	2.25E-26	2.58E-57
Rv2602	vapC41	0.4	0.4	-1.8	1	1	4	0.000832633	0.000515083	7.80E-57
Rv3172c	hypothetical protein	-0.1	0.6	-1.8	1	2	4	0.566750328	6.08E-05	9.74E-30
Rv2220	glnA1	1.1	1.0	-1.8	2	2	4	1.02E-26	1.18E-22	4.48E-63
Rv2178c	aroG	-0.4	1.2	-1.8	1	2	4	0.001237737	8.78E-29	4.93E-64
Rv0108c	hypothetical protein	0.2	-0.1	-1.8	1	1	4	0.174942795	0.391461976	3.42E-31
Rv0787	hypothetical protein	-0.8	0.9	-1.8	2	2	4	6.16E-09	2.03E-12	7.46E-52
Rv1194c	hypothetical protein	0.1	-0.1	-1.8	1	1	4	0.196954829	0.57444619	1.18E-63
Rv3478	PPE60	0.2	0.9	-1.8	1	2	4	0.164742844	2.57E-20	7.87E-68
Rv0864	moaC2	0.5	-0.8	-1.8	1	2	4	5.71E-06	3.53E-15	8.57E-66
Rv3426	PPE58	-0.5	0.5	-1.9	1	1	4	0.000425855	0.000116577	3.03E-54

Rv2462c	tig	0.6	0.6	-1.9	1	2	4	1.14E-07	1.18E-09	2.16E-63
Rv0268c	hypothetical protein	-0.4	-0.4	-1.9	1	1	4	0.00030419	0.001083826	1.51E-33
Rv2246	kasB	-1.4	1.9	-1.9	3	4	4	3.50E-25	3.13E-81	7.03E-59
Rv3676	crp	0.1	0.7	-1.9	1	2	4	0.416523822	1.33E-08	8.48E-55
Rv3486	hypothetical protein	0.0	1.0	-1.9	1	2	4	0.787306228	2.95E-15	3.73E-52
Rv3060c	GntR family transcriptional regulator	-0.4	0.5	-1.9	1	1	4	0.00058308	1.44E-06	1.01E-59
Rv0643c	mmaA3	-0.1	1.8	-1.9	1	3	4	0.270244402	3.77E-58	4.21E-65
Rv0951	sucC	0.4	1.3	-1.9	1	2	4	0.000830791	5.88E-40	5.37E-70
Rv2172c	hypothetical protein	0.0	0.4	-1.9	1	1	4	0.691468982	0.00016718	1.13E-49
Rv2104c	vapB37	-0.4	0.3	-1.9	1	1	4	0.000773755	0.037772863	7.53E-46
Rv2595	vapB40	-1.2	0.0	-1.9	2	1	4	1.21E-13	0.93677862	5.09E-38
Rv0634c	glyoxalase II	0.6	-0.2	-1.9	2	1	4	6.06E-08	0.037215469	3.32E-59
Rv1045	hypothetical protein	-0.1	0.8	-1.9	1	2	4	0.289217037	3.83E-16	9.41E-66
Rv2988c	leuC	-1.7	2.2	-1.9	3	5	4	1.45E-37	3.52E-85	5.47E-60
Rv2185c	TB16.3	0.6	0.6	-1.9	1	1	4	4.21E-05	2.57E-07	2.96E-59
Rv1057	hypothetical protein	0.4	0.8	-1.9	1	2	4	0.003443121	2.90E-13	3.74E-57
Rv0361	membrane protein	-0.6	2.1	-1.9	2	4	4	3.47E-06	3.63E-84	1.57E-68
Rv3856c	hypothetical protein	-0.1	0.5	-1.9	1	1	4	0.185158822	2.20E-06	8.71E-64
Rv0317c	glpQ2	-0.2	0.9	-1.9	1	2	4	0.091626904	5.17E-18	9.93E-59
Rv0959A	vapB9	-1.0	0.8	-1.9	2	2	4	8.76E-05	4.35E-05	4.78E-23
Rv1183	mmpL10	-0.1	0.1	-1.9	1	1	4	0.595777827	0.163941563	1.21E-71
Rv0221	diacyglycerol O-acyltransferase	-0.1	2.4	-1.9	1	5	4	0.348377557	2.46E-115	8.26E-68
Rv2412	rpsT	0.8	0.1	-1.9	2	1	4	1.26E-09	0.502528015	1.09E-36
Rv0363c	fba	0.0	1.6	-1.9	1	3	4	0.685211175	6.60E-52	8.08E-68
Rv0277c	vapC25	1.0	-1.0	-1.9	2	2	4	1.75E-14	4.84E-17	1.46E-61
Rv1361c	PPE19	0.2	0.9	-1.9	1	2	4	0.146543076	1.39E-21	4.18E-73
Rv1632c	hypothetical protein	-1.5	1.8	-1.9	3	3	4	1.84E-14	3.13E-47	2.66E-57
Rv1608c	bcpB	0.4	0.5	-1.9	1	1	4	0.000792967	2.06E-05	2.70E-45
Rv1397c	vapC10	-0.3	-0.9	-1.9	1	2	4	0.007344435	9.54E-19	9.58E-36
Rv1982A	vapB36	-0.2	0.0	-1.9	1	1	4	0.103145995	0.861086802	9.86E-39
Rv0960	vapC9	-0.4	0.6	-1.9	1	1	4	0.024036832	0.000126458	4.81E-37
Rv3584	lpqE	0.5	1.6	-1.9	1	3	4	5.69E-05	3.24E-36	3.26E-50
Rv1017c	prsA	-0.1	1.9	-1.9	1	4	4	0.452764088	2.41E-63	4.10E-63
Rv1813c	hypothetical protein	0.2	0.1	-1.9	1	1	4	0.187747027	0.615426538	2.77E-63
Rv0433	carboxylate-amine ligase	0.5	1.6	-1.9	1	3	4	2.41E-05	2.25E-49	1.16E-65
Rv3250c	rubB	0.8	-0.7	-1.9	2	2	4	2.35E-08	2.53E-07	2.79E-55
Rv0732	secY	0.0	1.9	-1.9	1	4	4	0.693011217	3.02E-74	6.32E-71
Rv3919c	gid	0.6	1.4	-1.9	2	3	4	9.29E-05	6.35E-31	1.10E-50
Rv1926c	mpt63	1.5	-2.9	-1.9	3	8	4	9.36E-17	7.18E-117	5.10E-52
Rv1306	atpF	1.2	-0.2	-1.9	2	1	4	7.21E-16	0.013086028	5.17E-67
Rv1095	phoH2	0.1	0.4	-1.9	1	1	4	0.354774648	0.00084367	6.60E-74
Rv3038c	hypothetical protein	-1.1	0.5	-1.9	2	1	4	1.76E-18	1.30E-05	1.86E-70
Rv1307	atpH	1.0	0.3	-1.9	2	1	4	1.99E-21	0.008632077	4.27E-70
Rv0042c	transcriptional regulator	-0.9	1.9	-1.9	2	4	4	4.77E-12	1.05E-55	2.43E-58
Rv1631	coaE	0.3	1.1	-1.9	1	2	4	0.002262702	5.96E-27	3.38E-73
Rv3744	nmtR	-1.3	0.9	-1.9	3	2	4	8.91E-14	2.88E-13	2.69E-57

Rv2080	lppJ	-0.1	0.9	-1.9	1	2	4	0.526025435	1.02E-11	7.17E-62
RV1984a	hypothetical protein CDS	-0.5	0.5	-1.9	1	1	4	0.000720017	4.21E-05	1.53E-47
Rv1310	atpD	0.6	0.7	-1.9	2	2	4	1.47E-08	9.76E-13	1.19E-75
Rv1924c	hypothetical protein	-0.6	0.6	-1.9	1	2	4	3.14E-06	7.67E-08	6.06E-62
Rv1304	atpB	1.0	-0.1	-1.9	2	1	4	2.10E-18	0.159241465	4.42E-60
Rv1416	ribH	0.5	0.5	-1.9	1	1	4	1.19E-05	3.91E-06	6.38E-60
Rv1303	hypothetical protein	0.7	-0.3	-1.9	2	1	4	3.10E-09	0.003270569	7.78E-69
Rv1388	mihF	-0.8	2.0	-2.0	2	4	4	5.77E-05	4.74E-71	9.51E-69
Rv0703	rplW	0.7	1.4	-2.0	2	3	4	6.88E-09	1.82E-34	7.84E-61
Rv1543	oxidoreductase	0.4	0.7	-2.0	1	2	4	0.000367599	6.21E-11	3.39E-59
Rv1142c	echA10	-0.8	0.6	-2.0	2	2	4	3.26E-10	9.14E-07	6.88E-66
Rv2930	fadD26	0.0	1.0	-2.0	1	2	4	0.740550143	6.46E-27	9.28E-48
Rv1539	lspA	0.7	-0.5	-2.0	2	1	4	4.61E-10	3.12E-06	1.44E-73
Rv0046c	ino1	-0.4	-0.6	-2.0	1	2	4	0.000153012	2.64E-07	4.26E-68
Rv3208	TetR family transcriptional regulator	-0.4	0.9	-2.0	1	2	4	0.008333304	5.59E-17	2.30E-58
Rv3519	hypothetical protein	-0.2	-0.5	-2.0	1	1	4	0.161377086	2.76E-05	5.47E-67
Rv0932c	pstS2	-0.3	0.3	-2.0	1	1	4	0.010907581	0.001186087	7.62E-57
Rv0733	adk	0.6	2.1	-2.0	1	4	4	1.58E-05	1.06E-84	1.57E-68
Rv2461c	clpP1	1.4	-0.8	-2.0	3	2	4	1.14E-27	2.25E-15	9.54E-51
Rv0472c	HTH-type transcriptional regulator	0.1	0.3	-2.0	1	1	4	0.34901871	0.008402178	4.97E-68
Rv3249c	TetR family transcriptional regulator	0.8	-0.3	-2.0	2	1	4	2.74E-15	0.002392437	1.01E-63
Rv3811	hypothetical protein	-0.4	1.4	-2.0	1	3	4	0.001039184	1.43E-36	4.66E-80
Rv1828	HTH-type transcriptional regulator	-0.1	0.0	-2.0	1	1	4	0.443650422	0.666003537	5.92E-65
Rv2442c	rplU	0.6	1.3	-2.0	2	2	4	1.58E-08	4.21E-29	1.23E-52
Rv0053	rpsF	-0.7	1.4	-2.0	2	3	4	0.00210198	9.87E-25	2.96E-54
Rv2987c	leuD	-0.9	1.6	-2.0	2	3	4	9.13E-15	1.32E-40	2.23E-68
Rv0647c	hypothetical protein	-1.3	2.3	-2.0	2	5	4	9.51E-18	2.87E-88	3.79E-76
Rv2243	fabD	-2.8	0.9	-2.0	7	2	4	1.22E-81	1.96E-09	4.51E-76
Rv0206c	mmpL3	-0.3	1.4	-2.0	1	3	4	0.005552287	2.13E-48	1.83E-84
Rv0479c	membrane protein	0.3	0.2	-2.0	1	1	4	0.008330286	0.062562265	1.59E-71
Rv2942	mmpL7	0.2	0.6	-2.0	1	2	4	0.097225297	7.56E-10	6.52E-78
Rv2291	sseB	-0.1	-0.4	-2.0	1	1	4	0.586550395	4.66E-05	3.23E-76
Rv1829	hypothetical protein	0.3	-0.2	-2.0	1	1	4	0.010128978	0.041529178	4.46E-58
Rv2947c	pks15	0.3	2.2	-2.0	1	5	4	0.128816015	3.09E-90	1.23E-43
Rv2093c	tatC	-0.3	0.7	-2.0	1	2	4	0.005713433	1.64E-12	7.64E-70
Rv1016c	lpqT	0.1	0.9	-2.0	1	2	4	0.222522066	1.48E-13	1.46E-61
Rv1233c	hypothetical protein	-0.8	1.7	-2.0	2	3	4	4.17E-11	5.48E-37	1.39E-65
Rv1507A	hypothetical protein	-0.1	1.6	-2.0	1	3	4	0.662229461	3.57E-33	1.24E-62
Rv1196	PPE18	0.6	0.7	-2.0	2	2	4	2.84E-05	5.59E-13	5.60E-84
Rv2477c	macrolide ABC transporter ATP-bindir	0.2	1.4	-2.0	1	3	4	0.05673395	1.46E-34	4.77E-81
Rv3267	hypothetical protein	-0.2	0.8	-2.0	1	2	4	0.073175694	1.81E-17	1.73E-79
Rv0870c	integral membrane protein	0.0	1.1	-2.0	1	2	4	0.928549289	8.19E-16	4.69E-63
Rv3409c	choD	0.3	-0.4	-2.0	1	1	4	0.012432107	0.000225062	1.22E-85
Rv2441c	rpmA	0.5	0.8	-2.0	1	2	4	4.15E-05	2.24E-10	4.03E-59
Rv3710	leuA	0.0	0.6	-2.0	1	2	4	0.747896896	1.90E-10	8.10E-76
Rv3336c	trpS	-0.8	1.2	-2.0	2	2	4	1.13E-07	2.57E-24	6.85E-66

Rv3460c	rpsM	1.0	0.4	-2.0	2	1	4	2.67E-13	0.000213236	1.14E-65
Rv3213c	SOJ/ParA-like protein	-0.3	0.7	-2.0	1	2	4	0.043102989	1.33E-10	9.98E-67
Rv3769	hypothetical protein	-0.3	0.2	-2.1	1	1	4	0.034808008	0.068321712	3.05E-62
Rv3425	PPE57	-0.4	0.4	-2.1	1	1	4	0.018434685	0.002932229	2.16E-67
Rv1484	inhA	-0.5	1.3	-2.1	1	2	4	2.65E-05	2.68E-30	1.26E-74
Rv1535	hypothetical protein	-0.9	1.1	-2.1	2	2	4	8.86E-11	3.12E-11	4.73E-55
Rv1677	dsbF	0.1	0.8	-2.1	1	2	4	0.504506724	4.27E-13	7.64E-70
Rv3822	hypothetical protein	-0.8	0.7	-2.1	2	2	4	1.32E-14	7.63E-10	5.31E-81
Rv0724	sppA	0.1	-0.6	-2.1	1	1	4	0.150789108	2.73E-07	5.72E-85
Rv1155a	hypothetical protein CDS	0.1	-0.8	-2.1	1	2	4	0.727141649	1.55E-10	1.34E-50
Rv1980c	mpt64	1.6	0.3	-2.1	3	1	4	2.11E-46	0.002121212	1.20E-84
Rv3457c	rpoA	0.4	0.7	-2.1	1	2	4	0.000623366	1.06E-10	1.35E-73
Rv2989	transcriptional regulator	-2.7	1.3	-2.1	7	2	4	1.31E-76	1.11E-26	4.14E-74
Rv1107c	xseB	0.3	1.3	-2.1	1	2	4	0.023050272	3.29E-24	1.42E-66
Rv0175	Mce associated membrane protein	0.0	1.1	-2.1	1	2	4	0.934649056	4.45E-26	1.97E-84
Rv2953	trans-acting enoyl reductase	-0.3	2.4	-2.1	1	5	4	0.01870918	2.76E-89	1.84E-67
Rv1308	atpA	0.9	0.2	-2.1	2	1	4	3.24E-18	0.122251529	7.31E-89
Rv1643	rplT	0.7	1.1	-2.1	2	2	4	2.19E-10	7.88E-23	1.31E-55
Rv3697A	vapB48	-0.5	0.5	-2.1	1	1	4	0.000106389	0.000206854	7.86E-52
Rv1173	fbiC	-0.2	0.3	-2.1	1	1	4	0.051427569	0.001276686	1.89E-78
Rv0822c	hypothetical protein	-1.2	2.1	-2.1	2	4	4	2.06E-23	8.91E-102	1.32E-91
Rv1437	pgk	0.2	2.0	-2.1	1	4	4	0.109011792	9.23E-80	3.04E-74
Rv2274A	mazE8	-0.7	2.0	-2.1	2	4	4	2.75E-07	3.89E-40	6.88E-55
Rv0952	sucD	0.6	1.7	-2.1	2	3	4	7.87E-05	2.32E-53	3.41E-66
Rv2114	hypothetical protein	-0.1	-0.2	-2.1	1	1	4	0.386121475	0.069015196	5.69E-80
Rv2241	aceE	0.5	1.1	-2.1	1	2	4	3.08E-07	2.80E-30	1.50E-89
Rv0060	hypothetical protein	0.4	-0.4	-2.1	1	1	4	0.000441504	5.54E-06	4.68E-62
Rv3312A	pilin	-1.8	1.1	-2.1	4	2	4	3.24E-46	1.94E-17	3.18E-64
Rv2784c	lppU	0.4	0.4	-2.1	1	1	4	0.001165708	0.000202568	1.09E-78
Rv0598c	vapC27	-0.5	1.2	-2.1	1	2	4	6.72E-05	6.42E-31	1.06E-78
Rv0450c	mmpL4	-1.0	1.8	-2.1	2	3	4	9.13E-16	1.78E-76	3.21E-92
Rv2395B	aprB	-0.3	-0.9	-2.1	1	2	4	0.011174284	2.45E-20	5.44E-77
Rv2460c	clpP2	1.0	-0.6	-2.1	2	2	4	7.98E-18	9.75E-07	4.39E-69
Rv0487	hypothetical protein	-0.4	0.0	-2.1	1	1	4	0.016650834	0.704411675	1.33E-76
Rv0432	sodC	-0.1	1.5	-2.1	1	3	4	0.41217776	1.06E-32	3.58E-63
Rv0961	integral membrane protein	0.3	0.3	-2.1	1	1	4	0.010010749	0.001817448	3.10E-77
Rv2429	ahpD	0.8	0.5	-2.1	2	1	4	3.61E-10	2.33E-05	2.48E-67
Rv2331	hypothetical protein	-1.1	0.2	-2.1	2	1	4	2.87E-11	0.08913624	2.31E-54
Rv3207c	hypothetical protein	0.1	0.5	-2.1	1	1	4	0.541608646	3.13E-06	6.94E-85
Rv3424c	hypothetical protein	-0.1	0.2	-2.1	1	1	4	0.641402703	0.050272386	3.94E-76
Rv3755c	hypothetical protein	-0.1	-0.5	-2.1	1	1	4	0.488293847	8.52E-07	7.71E-67
Rv0910	toxin	-0.3	1.5	-2.2	1	3	4	0.024759468	3.16E-37	2.36E-65
Rv3006	lppZ	-1.5	1.5	-2.2	3	3	4	4.71E-20	7.34E-45	8.39E-72
Rv3464	rmlB	0.1	2.1	-2.2	1	4	4	0.678004546	2.00E-93	3.69E-87
Rv3849	espR	-0.2	0.7	-2.2	1	2	4	0.044839324	3.32E-10	7.98E-76
Rv2295	hypothetical protein	-0.6	0.7	-2.2	2	2	4	9.36E-08	4.32E-10	1.63E-64

Rv2601A	vapB41	-0.5	0.3	-2.2	1	1	4	0.000280615	0.004213823	1.23E-69
Rv1159A	4a-hydroxytetrahydrobiopterin dehydr	0.9	-1.9	-2.2	2	4	4	6.53E-15	4.24E-78	6.63E-61
Rv1044	hypothetical protein	-1.8	1.7	-2.2	3	3	5	1.54E-37	1.55E-49	8.38E-73
Rv3759c	proX	-0.1	-0.2	-2.2	1	1	5	0.331341544	0.023034438	1.30E-90
Rv2954c	hypothetical protein	0.2	1.7	-2.2	1	3	5	0.223416916	2.91E-52	3.47E-84
Rv2109c	prcA	0.8	-0.5	-2.2	2	1	5	7.59E-15	1.68E-07	2.08E-83
Rv2778c	hypothetical protein	-0.2	1.8	-2.2	1	4	5	0.100743519	3.58E-45	1.68E-73
Rv1219c	transcriptional regulator	-0.8	3.0	-2.2	2	8	5	3.15E-12	4.62E-162	1.82E-68
Rv3252c	alkB	0.3	0.3	-2.2	1	1	5	0.007138171	0.001428921	1.13E-85
Rv2548A	hypothetical protein	-0.2	0.8	-2.2	1	2	5	0.091762595	3.51E-14	5.25E-60
Rv0001	dnaA	-0.4	1.5	-2.2	1	3	5	0.000151677	8.34E-59	8.49E-88
Rv2952	phthiotriol/phenolphthiotriol dimycoc	-1.1	2.7	-2.2	2	7	5	4.97E-15	2.40E-124	3.10E-74
Rv2369c	hypothetical protein	-0.3	0.5	-2.2	1	1	5	0.057451052	0.00023023	2.70E-54
Rv1827	garA	0.2	-0.1	-2.2	1	1	5	0.276166762	0.614720669	5.77E-61
Rv3197	ABC transporter ATP-binding protein	-0.1	-1.2	-2.2	1	2	5	0.616968731	1.52E-35	6.98E-93
Rv0078A	hypothetical protein	0.6	-0.7	-2.2	1	2	5	3.24E-07	7.12E-09	1.53E-64
Rv3857c	membrane protein	-1.2	0.7	-2.2	2	2	5	2.89E-08	4.59E-08	6.46E-67
Rv1751	oxidoreductase	-0.7	2.0	-2.2	2	4	5	6.73E-11	2.69E-78	5.92E-89
Rv1857	modA	0.5	2.0	-2.2	1	4	5	0.000710345	8.20E-52	6.41E-64
Rv1309	atpG	1.2	0.5	-2.2	2	1	5	2.26E-27	1.18E-08	1.20E-71
Rv0632c	echA3	0.0	0.2	-2.2	1	1	5	0.978705298	0.060225025	1.04E-82
Rv1798	eccA5	0.4	0.5	-2.2	1	1	5	0.002576871	6.46E-09	1.11E-96
Rv0599c	vapB27	-1.5	1.2	-2.2	3	2	5	2.45E-25	5.12E-18	5.35E-60
Rv1262c	hypothetical protein	-1.0	2.1	-2.2	2	4	5	7.74E-13	2.35E-60	1.47E-71
Rv1772	hypothetical protein	0.1	0.2	-2.2	1	1	5	0.382494597	0.15394206	2.43E-59
Rv2097c	pafA	0.5	0.4	-2.2	1	1	5	2.10E-05	3.16E-05	2.83E-95
Rv2239c	hypothetical protein	0.1	1.3	-2.2	1	3	5	0.301642307	7.31E-35	1.56E-87
Rv0748	vapB31	0.7	-1.2	-2.2	2	2	5	1.26E-07	1.10E-34	1.90E-51
Rv3686c	hypothetical protein	-1.2	1.8	-2.2	2	3	5	5.62E-18	1.64E-34	1.29E-68
Rv0721	rpsE	0.5	2.0	-2.2	1	4	5	6.01E-05	2.12E-82	1.24E-83
Rv2512a	hypothetical protein CDS	0.0	0.1	-2.3	1	1	5	0.972210278	0.37462847	1.62E-53
Rv0464c	hypothetical protein	0.3	-0.1	-2.3	1	1	5	0.002439429	0.254216526	4.60E-87
Rv2289	cdh	-0.3	2.1	-2.3	1	4	5	0.006333823	9.08E-70	1.46E-90
Rv1305	atpE	1.0	-0.6	-2.3	2	1	5	1.49E-09	5.00E-07	2.25E-89
Rv0634A	hypothetical protein	-0.1	1.5	-2.3	1	3	5	0.301046101	1.66E-38	6.70E-75
Rv1415	ribA2	-0.1	0.5	-2.3	1	1	5	0.348085257	1.72E-05	1.67E-96
Rv3679	anion transporter ATPase	-0.1	-0.2	-2.3	1	1	5	0.259621379	0.021929445	2.61E-83
Rv1475c	acn	0.2	1.1	-2.3	1	2	5	0.123479413	2.26E-23	1.25E-77
Rv2271	hypothetical protein	1.4	1.3	-2.3	3	2	5	5.67E-28	7.25E-26	5.71E-73
Rv3918c	parA	-0.1	2.1	-2.3	1	4	5	0.270995313	1.92E-88	5.25E-85
Rv2903c	lepB	0.5	0.8	-2.3	1	2	5	0.000106553	4.30E-14	1.28E-90
Rv1699	pyrG	0.1	0.2	-2.3	1	1	5	0.515830543	0.074023953	4.36E-103
Rv2190c	endopeptidase	0.8	1.3	-2.3	2	3	5	2.92E-12	7.49E-35	3.68E-95
Rv1987	chitinase	0.3	3.1	-2.3	1	9	5	0.028317227	5.98E-171	2.08E-57
Rv2334	cysK1	0.2	2.0	-2.3	1	4	5	0.136821664	5.18E-88	1.62E-72
Rv2588c	yajC	0.7	0.5	-2.3	2	1	5	1.70E-08	5.18E-05	2.96E-60

Rv2199c	cytochrome c oxidase polypeptide 4	0.3	1.4	-2.3	1	3	5	0.00891005	2.07E-31	6.27E-85
Rv3678c	hypothetical protein	0.4	0.6	-2.3	1	1	5	0.002417097	9.60E-07	3.16E-79
Rv1611	trpC	-0.1	2.1	-2.3	1	4	5	0.326137396	2.72E-84	3.07E-101
Rv1332	transcriptional regulator	-0.2	-0.8	-2.3	1	2	5	0.12308151	1.06E-11	2.09E-86
Rv2245	kasA	-1.3	1.8	-2.3	3	3	5	4.88E-35	1.32E-66	3.64E-106
Rv3251c	rubA	0.1	0.0	-2.3	1	1	5	0.406863316	0.971676959	1.15E-48
Rv3142c	hypothetical protein	-0.3	0.6	-2.3	1	2	5	0.029961911	5.53E-07	1.20E-86
Rv2937	drrB	-0.6	1.7	-2.3	1	3	5	1.52E-07	1.48E-65	2.67E-103
Rv1636	TB15.3	0.0	0.1	-2.3	1	1	5	0.946298874	0.626399574	1.98E-92
Rv3491	hypothetical protein	-0.1	0.9	-2.3	1	2	5	0.61393691	8.50E-17	2.13E-86
Rv0056	rplI	0.9	1.3	-2.3	2	3	5	7.07E-15	9.44E-30	3.55E-69
Rv2348c	hypothetical protein	1.0	0.4	-2.3	2	1	5	4.35E-08	9.27E-05	3.10E-77
Rv1780	hypothetical protein	-0.1	0.2	-2.3	1	1	5	0.625729837	0.123110056	3.27E-83
Rv3920c	hypothetical protein	0.8	2.3	-2.3	2	5	5	1.38E-05	1.50E-97	6.98E-81
Rv3875	esxA	0.0	0.2	-2.3	1	1	5	0.841992556	0.07685531	1.86E-54
Rv1884c	rpfC	1.1	2.5	-2.3	2	6	5	2.68E-18	1.32E-95	8.19E-84
Rv3248c	sahH	0.2	0.5	-2.3	1	1	5	0.072083673	1.18E-06	2.30E-108
Rv0238	transcriptional regulator	-0.2	0.7	-2.3	1	2	5	0.083209826	1.18E-09	6.62E-71
Rv3487c	lipF	-0.6	0.5	-2.3	2	1	5	1.77E-07	0.00095613	5.69E-94
Rv1906c	hypothetical protein	-0.6	0.3	-2.3	2	1	5	0.001004917	0.006901731	3.96E-73
Rv2009	vapB15	0.1	0.3	-2.3	1	1	5	0.504403706	0.000507733	3.52E-10
Rv1483	fabG1	-0.1	1.4	-2.3	1	3	5	0.56885212	3.13E-42	9.00E-100
Rv3923c	rnpA	0.6	2.4	-2.3	2	5	5	5.43E-05	4.52E-96	2.03E-91
Rv3209	hypothetical protein	0.2	2.1	-2.3	1	4	5	0.062076281	8.38E-88	4.55E-76
Rv3924c	rpmH	0.4	2.2	-2.4	1	5	5	0.009252028	1.22E-67	1.06E-77
Rv1261c	hypothetical protein	-0.8	0.9	-2.4	2	2	5	4.61E-08	1.02E-15	2.21E-88
Rv2091c	membrane protein	0.0	0.0	-2.4	1	1	5	0.716585889	0.885893345	1.43E-96
Rv1869c	reductase	-0.2	1.0	-2.4	1	2	5	0.049960571	8.12E-22	1.56E-109
Rv0685	tuf	0.8	1.7	-2.4	2	3	5	1.88E-09	5.93E-74	2.62E-70
Rv3922c	membrane protein insertion efficiency	0.6	2.4	-2.4	2	5	5	0.000209609	1.34E-100	1.34E-69
Rv2720	lexA	-0.9	1.3	-2.4	2	2	5	2.77E-10	9.72E-37	8.98E-102
Rv2919c	glnB	0.4	-1.7	-2.4	1	3	5	0.011123612	2.22E-62	5.83E-95
Rv3280	accD5	0.9	1.3	-2.4	2	2	5	3.54E-06	3.08E-34	4.80E-110
Rv2376c	cfp2	-1.1	1.6	-2.4	2	3	5	2.22E-09	4.67E-27	1.27E-54
Rv1489	hypothetical protein	0.3	-0.2	-2.4	1	1	5	0.009519223	0.123337157	2.90E-94
Rv1626	two-component system transcriptiona	0.3	0.8	-2.4	1	2	5	0.016990819	1.10E-12	3.15E-97
Rv1501	hypothetical protein	0.0	0.8	-2.4	1	2	5	0.809351803	3.42E-09	1.98E-53
Rv0483	lprQ	0.0	1.3	-2.4	1	2	5	0.904800839	3.48E-32	1.98E-108
Rv0020c	fhaA	0.1	0.5	-2.4	1	1	5	0.264655873	3.47E-06	4.17E-117
Rv3855	ethR	-0.2	1.5	-2.4	1	3	5	0.056979075	2.43E-47	2.85E-103
Rv1887	hypothetical protein	0.1	1.2	-2.4	1	2	5	0.272269056	1.87E-32	1.09E-112
Rv0164	TB18.5	0.0	2.2	-2.4	1	4	5	0.704807499	1.29E-84	1.13E-96
Rv0636	hadB	0.3	1.7	-2.4	1	3	5	0.010127213	1.99E-56	8.88E-94
Rv3095	HTH-type transcriptional regulator	-1.7	2.7	-2.4	3	7	5	3.68E-51	9.70E-154	4.31E-111
Rv2368c	phoH1	0.2	0.3	-2.4	1	1	5	0.026456196	0.005790874	1.99E-69
Rv0462	lpdC	0.6	0.3	-2.4	2	1	5	9.48E-08	0.000872472	7.40E-107

Rv2142A	parD2	-0.2	-1.0	-2.4	1	2	5	0.178546148	3.77E-19	5.64E-53
Rv3224	iron-regulated short-chain dehydroge	-0.1	1.9	-2.4	1	4	5	0.366919369	1.70E-56	1.10E-89
Rv3842c	glpQ1	0.2	-0.1	-2.4	1	1	5	0.070510518	0.386434903	4.00E-116
Rv3412	hypothetical protein	0.4	-0.3	-2.4	1	1	5	0.001036599	0.004440551	2.85E-98
Rv3804c	fbpA	0.2	1.4	-2.4	1	3	5	0.141980491	3.37E-42	7.74E-117
Rv2936	drrA	-1.0	1.8	-2.4	2	3	5	3.73E-15	3.07E-74	2.28E-95
Rv3620c	esxW	0.8	0.6	-2.4	2	1	5	1.18E-09	2.47E-09	1.91E-115
Rv0476	transmembrane protein	0.6	-1.9	-2.4	2	4	5	6.28E-05	1.69E-57	1.39E-71
Rv0898c	hypothetical protein	-0.8	-0.7	-2.4	2	2	5	3.04E-07	6.13E-11	2.18E-95
Rv1614	lgt	0.1	1.9	-2.4	1	4	5	0.232595428	1.20E-71	6.16E-111
Rv2993c	2-hydroxyhepta-2,4-diene-1,7-dioate	-0.9	2.9	-2.5	2	7	5	5.48E-15	5.28E-144	2.75E-101
Rv1038c	esxJ	0.9	0.5	-2.5	2	1	5	1.94E-09	5.72E-09	1.05E-117
Rv3136	PPE51	-0.8	1.7	-2.5	2	3	5	9.94E-13	6.71E-62	5.76E-115
Rv1103c	mazE3	-1.3	0.9	-2.5	2	2	5	2.17E-31	2.71E-15	3.01E-108
Rv0207c	hypothetical protein	-0.2	1.0	-2.5	1	2	5	0.110861386	8.53E-25	3.66E-111
Rv2970A	hypothetical protein	0.8	-0.1	-2.5	2	1	6	2.80E-07	0.533892363	8.69E-71
Rv1794	hypothetical protein	0.0	1.1	-2.5	1	2	6	0.947430776	3.19E-32	4.39E-121
Rv0683	rpsG	1.6	0.6	-2.5	3	2	6	8.79E-31	2.68E-09	1.30E-92
Rv3377c	type B diterpene cyclase	-0.1	1.5	-2.5	1	3	6	0.418389906	2.54E-53	1.01E-109
Rv2074	pyridoxamine 5'-phosphate oxidase	-0.5	0.6	-2.5	1	2	6	0.0112132	1.39E-06	2.02E-80
Rv1932	tpx	0.5	1.0	-2.5	1	2	6	0.023647714	2.78E-14	4.03E-83
Rv3484	hypothetical protein	-0.4	1.9	-2.5	1	4	6	0.000278862	5.56E-79	2.20E-121
Rv0642c	mmaA4	-0.6	2.2	-2.5	1	5	6	8.53E-08	3.05E-81	6.06E-105
Rv1387	PPE20	-0.8	3.6	-2.5	2	12	6	7.07E-13	1.07E-297	3.81E-120
Rv1198	esxL	0.4	0.5	-2.5	1	1	6	0.022087827	1.17E-08	2.53E-118
Rv0503c	cmaA2	-0.6	1.6	-2.5	1	3	6	1.60E-06	2.37E-52	7.80E-105
Rv2556c	hypothetical protein	-0.1	0.0	-2.5	1	1	6	0.470058731	0.915825283	4.56E-101
Rv3278c	transmembrane protein	0.8	1.4	-2.5	2	3	6	2.60E-10	9.64E-33	2.17E-76
Rv2939	papA5	-0.5	1.5	-2.5	1	3	6	2.84E-05	2.20E-56	1.85E-123
Rv2928	tesA	-0.1	2.3	-2.5	1	5	6	0.504968772	1.84E-92	1.97E-105
Rv0469	umaA	0.3	0.8	-2.5	1	2	6	0.011966242	1.33E-15	2.52E-114
Rv3260c	whiB2	-0.1	0.6	-2.5	1	2	6	0.522054871	6.64E-07	5.90E-91
Rv0694	mycofactocin system heme/flavin oxi	-0.5	2.5	-2.5	1	6	6	1.30E-06	7.11E-137	7.77E-125
Rv0220	lipC	-1.3	2.1	-2.5	3	4	6	1.28E-18	3.10E-48	3.15E-117
Rv3284	hypothetical protein	0.7	-0.7	-2.5	2	2	6	6.12E-11	1.02E-09	1.63E-95
Rv3678A	hypothetical protein	-1.7	1.9	-2.5	3	4	6	9.74E-31	2.12E-47	2.96E-78
Rv1436	gap	0.9	1.3	-2.5	2	2	6	3.22E-17	6.27E-42	4.18E-119
Rv3229c	desA3	-2.1	1.8	-2.5	4	4	6	2.24E-51	2.83E-64	9.02E-98
Rv2470	glbO	-0.5	0.2	-2.5	1	1	6	0.007408387	0.021518655	3.48E-89
Rv2204c	hypothetical protein	0.7	-0.2	-2.5	2	1	6	0.000185967	0.029787486	3.29E-84
Rv1297	rho	0.0	1.0	-2.5	1	2	6	0.644920581	3.43E-22	1.90E-114
Rv3136A	hypothetical protein	-0.5	2.0	-2.5	1	4	6	2.83E-05	1.04E-67	9.94E-102
Rv3416	whiB3	-0.1	-0.7	-2.5	1	2	6	0.418830488	4.62E-09	1.45E-100
Rv1386	PE15	-1.6	3.5	-2.5	3	11	6	4.98E-29	3.44E-171	3.67E-82
Rv0229c	hypothetical protein	-1.1	1.3	-2.5	2	2	6	3.69E-22	8.14E-24	5.83E-103
Rv1521	fadD25	0.3	0.6	-2.5	1	2	6	0.004180371	1.00E-11	5.37E-125

Rv2375	hypothetical protein	0.4	1.4	-2.5	1	3	6	0.000350206	2.08E-29	4.13E-78
Rv0716	rplE	0.4	2.2	-2.5	1	4	6	0.000348671	3.26E-82	1.14E-106
Rv2111c	pup	-1.0	-0.1	-2.6	2	1	6	3.81E-12	0.313780729	2.54E-84
Rv1612	trpB	-0.4	2.4	-2.6	1	5	6	0.001419294	2.24E-124	2.02E-121
Rv0501	galE2	-0.2	0.9	-2.6	1	2	6	0.131703689	5.63E-20	4.20E-124
Rv2347c	esxP	0.9	0.6	-2.6	2	1	6	2.57E-09	1.40E-09	6.44E-125
Rv1155	pyridoxine/pyridoxamine 5'-phosphat	-0.7	1.1	-2.6	2	2	6	0.000133276	2.19E-18	3.96E-99
Rv0010c	membrane protein	0.8	0.7	-2.6	2	2	6	3.27E-12	6.91E-12	5.61E-118
Rv1197	esxK	0.9	0.6	-2.6	2	1	6	7.67E-09	1.87E-09	1.10E-126
Rv0299	toxin	-0.2	0.6	-2.6	1	1	6	0.312908342	1.58E-05	1.30E-82
Rv2128	transmembrane protein	0.7	0.5	-2.6	2	1	6	7.21E-09	9.60E-06	1.05E-92
Rv0190	hypothetical protein	-1.1	0.1	-2.6	2	1	6	3.75E-19	0.18131145	9.85E-112
Rv3429	PPE59	-0.5	0.4	-2.6	1	1	6	0.000688162	0.001875127	4.17E-101
Rv3619c	esxV	0.4	0.6	-2.6	1	1	6	0.01314607	2.93E-09	3.24E-132
Rv3651	hypothetical protein	-1.3	2.2	-2.6	2	5	6	2.96E-34	1.08E-109	1.44E-129
Rv0708	rplP	-0.4	1.9	-2.6	1	4	6	0.001482587	3.75E-66	3.17E-121
Rv2901c	hypothetical protein	0.8	0.2	-2.6	2	1	6	8.19E-05	0.087868979	7.00E-120
Rv0177	Mce associated protein	0.3	1.9	-2.6	1	4	6	0.039232328	3.46E-75	1.39E-109
Rv1642	rpmI	0.7	1.1	-2.6	2	2	6	8.18E-08	5.61E-23	5.54E-65
Rv0684	fusA1	0.7	1.3	-2.6	2	2	6	4.17E-07	1.10E-45	1.26E-122
Rv1826	gcvH	0.0	0.7	-2.6	1	2	6	0.710656563	3.84E-11	7.79E-99
Rv3246c	mtrA	-0.1	0.2	-2.6	1	1	6	0.351105727	0.022828287	7.02E-133
Rv0905	echA6	0.2	1.0	-2.6	1	2	6	0.130769117	1.33E-18	6.41E-108
Rv1037c	esxI	0.4	0.6	-2.6	1	1	6	0.013038835	3.42E-09	2.69E-134
Rv3295	TetR family transcriptional regulator	-1.6	1.6	-2.6	3	3	6	1.85E-27	2.49E-39	2.12E-108
Rv2905	lppW	-0.1	0.4	-2.6	1	1	6	0.444779422	0.001976605	6.18E-113
Rv1883c	hypothetical protein	1.0	0.8	-2.6	2	2	6	6.45E-20	3.13E-15	5.01E-116
Rv2395A	aprA	-1.8	-1.1	-2.6	3	2	6	1.46E-38	1.59E-12	1.33E-73
Rv1117	hypothetical protein	-1.0	1.1	-2.7	2	2	6	2.59E-11	9.19E-21	4.74E-102
Rv2150c	ftsZ	-0.7	0.8	-2.7	2	2	6	2.07E-08	1.96E-11	2.75E-108
Rv0720	rplR	0.2	2.4	-2.7	1	5	6	0.162862892	2.04E-79	5.10E-109
Rv3320c	vapC44	-0.5	2.1	-2.7	1	4	6	0.000150519	9.47E-79	7.01E-117
Rv0863	hypothetical protein	0.3	-0.6	-2.7	1	2	6	0.00159325	2.85E-08	3.81E-109
Rv3680	anion transporter ATPase	0.1	0.3	-2.7	1	1	6	0.309732464	0.006239229	1.68E-100
Rv0805	3',5'-cyclic adenosine monophosphat	0.7	0.2	-2.7	2	1	6	8.83E-12	0.114945117	4.54E-129
Rv1613	trpA	0.3	2.3	-2.7	1	5	6	0.015482878	4.68E-110	6.21E-122
Rv3075c	hypothetical protein	0.0	0.5	-2.7	1	1	6	0.863618845	1.63E-06	1.75E-124
Rv0641	rplA	-0.3	0.9	-2.7	1	2	6	0.019520401	4.60E-18	1.61E-129
Rv2166c	transcriptional regulator MraZ	0.0	0.3	-2.7	1	1	6	0.886237291	0.001583661	5.02E-119
Rv3477	PE31	0.2	2.2	-2.7	1	5	6	0.058800346	9.13E-85	1.93E-93
Rv0009	ppiA	0.3	1.2	-2.7	1	2	6	0.030124975	9.46E-32	9.16E-120
Rv0706	rplV	0.1	2.1	-2.7	1	4	6	0.409485588	8.67E-72	2.22E-118
Rv1324	thioredoxin	0.2	0.4	-2.7	1	1	6	0.022148436	4.14E-06	1.35E-89
Rv0638	secE1	1.1	0.1	-2.7	2	1	6	3.84E-11	0.489026956	3.20E-135
Rv1676	hypothetical protein	0.4	0.5	-2.7	1	1	6	0.000860233	3.45E-06	1.95E-115
Rv1156	hypothetical protein	0.9	-0.4	-2.7	2	1	6	8.77E-10	7.70E-05	3.68E-128

Rv1411c	lprG	-0.2	0.6	-2.7	1	1	6	0.09672792	6.90E-09	5.71E-138
Rv2115c	mpa	0.1	-0.3	-2.7	1	1	6	0.366454189	0.001474264	1.37E-123
Rv0909	antitoxin	-0.6	1.5	-2.7	1	3	6	8.81E-06	1.61E-38	2.51E-91
Rv3005c	hypothetical protein	-0.7	1.4	-2.7	2	3	7	4.39E-09	1.47E-27	5.46E-117
Rv1006	hypothetical protein	-0.4	1.7	-2.7	1	3	7	0.000384874	3.15E-50	1.83E-134
Rv0301	vapC2	-0.3	0.7	-2.7	1	2	7	0.00394637	1.06E-12	2.97E-121
Rv2196	qcrB	0.3	-0.1	-2.7	1	1	7	0.002036415	0.508464198	3.11E-138
Rv0639	nusG	0.8	0.0	-2.7	2	1	7	1.89E-10	0.987253725	1.51E-135
Rv0239	vapB24	-0.6	-0.1	-2.7	2	1	7	1.86E-07	0.570502873	3.17E-116
Rv3645	transmembrane protein	-0.6	1.4	-2.7	2	3	7	5.64E-09	1.86E-40	5.43E-149
Rv2110c	prcB	0.0	-0.2	-2.7	1	1	7	0.744437604	0.092038942	1.07E-122
Rv0718	rpsH	0.6	2.1	-2.7	2	4	7	0.000317905	1.25E-90	1.40E-132
Rv0723	rplO	0.4	2.0	-2.7	1	4	7	0.024842148	1.60E-69	3.72E-108
Rv3135	PPE50	-1.5	1.8	-2.7	3	3	7	1.18E-36	3.73E-52	4.22E-120
Rv2346c	esxO	0.3	0.6	-2.8	1	2	7	0.031498532	1.65E-10	4.56E-147
Rv0635	hadA	0.4	2.2	-2.8	1	5	7	5.38E-05	1.14E-100	5.35E-139
Rv0640	rplK	1.3	0.2	-2.8	2	1	7	3.56E-19	0.02284668	1.85E-134
Rv0637	hadC	0.5	1.6	-2.8	1	3	7	4.48E-05	2.27E-52	2.67E-125
Rv0700	rpsJ	0.9	1.0	-2.8	2	2	7	3.30E-11	9.79E-21	5.36E-129
Rv2861c	mapB	-0.6	2.1	-2.8	1	4	7	3.26E-07	1.15E-89	1.28E-135
Rv2290	lppO	-0.4	0.1	-2.8	1	1	7	3.99E-05	0.359922146	6.47E-143
Rv2195	qcrA	0.5	0.1	-2.8	1	1	7	2.43E-07	0.468719518	8.34E-138
Rv1793	esxN	0.4	0.6	-2.8	1	1	7	0.014597651	2.31E-09	2.95E-138
Rv0470c	pcaA	-0.4	0.9	-2.8	1	2	7	0.000233725	6.62E-18	6.24E-145
Rv0707	rpsC	0.0	2.2	-2.8	1	5	7	0.790800068	1.77E-100	3.92E-147
Rv0693	mycofactocin radical SAM maturase N	0.3	2.3	-2.8	1	5	7	0.028330944	4.13E-109	3.96E-142
Rv2222c	glnA2	-0.9	0.6	-2.8	2	2	7	2.70E-07	1.77E-06	5.24E-143
Rv3461c	rpmJ	0.1	0.1	-2.8	1	1	7	0.297245399	0.265087647	8.57E-119
Rv2238c	ahpE	0.1	1.1	-2.8	1	2	7	0.64587952	2.30E-26	6.51E-116
Rv0722	rpmD	0.2	2.4	-2.8	1	5	7	0.219678486	1.30E-61	5.37E-96
Rv0717	rpsN1	0.2	2.4	-2.8	1	5	7	0.264154085	1.06E-75	1.26E-105
Rv3283	sseA	-0.1	-0.6	-2.8	1	2	7	0.44144576	1.59E-08	3.55E-134
Rv3782	glfT1	-0.5	1.1	-2.8	1	2	7	6.89E-06	9.87E-26	5.12E-147
Rv3921c	membrane protein insertase YidC	0.2	2.6	-2.8	1	6	7	0.085438895	2.88E-147	3.97E-121
Rv0005	gyrB	0.4	0.1	-2.8	1	1	7	0.008787498	0.368448392	1.36E-128
Rv3742c	oxidoreductase	0.8	-0.8	-2.8	2	2	7	2.99E-09	2.37E-14	3.25E-94
Rv3490	otsA	-0.5	1.2	-2.8	1	2	7	5.36E-06	1.28E-31	1.60E-161
Rv1360	oxidoreductase	-1.4	2.5	-2.8	3	6	7	1.34E-37	2.47E-120	1.04E-156
Rv0502	hypothetical protein	-0.3	1.1	-2.8	1	2	7	0.00611907	1.47E-32	1.82E-153
Rv0702	rplD	0.8	1.9	-2.8	2	4	7	1.17E-14	2.78E-80	1.72E-134
Rv1697	hypothetical protein	-0.5	2.9	-2.8	1	7	7	7.82E-05	4.71E-153	5.11E-135
Rv0430	hypothetical protein	-2.1	2.3	-2.9	4	5	7	7.19E-30	4.38E-74	1.15E-118
Rv1174c	TB8.4	0.0	0.2	-2.9	1	1	7	0.809795725	0.036208355	5.28E-132
Rv2945c	lppX	0.2	2.5	-2.9	1	6	7	0.08655118	1.13E-137	2.97E-134
Rv1102c	mazF3	-0.8	1.3	-2.9	2	2	7	1.32E-11	9.12E-35	2.33E-103
Rv0823c	tRNA-dihydrouridine synthase	-0.3	1.2	-2.9	1	2	7	0.004399144	8.69E-30	2.97E-128

Rv2194	qcrC	0.8	0.1	-2.9	2	1	7	1.11E-10	0.244892973	2.89E-131
Rv3750c	excisionase	0.1	0.9	-2.9	1	2	7	0.527901379	1.71E-19	4.81E-153
Rv2145c	wag31	-0.4	1.0	-2.9	1	2	7	0.000832424	2.46E-20	1.84E-152
Rv3376	phosphatase	-0.2	1.1	-2.9	1	2	8	0.039248424	2.77E-25	4.33E-160
Rv3321c	vapB44	-0.5	1.8	-2.9	1	3	8	2.08E-05	2.14E-47	3.04E-114
Rv2200c	ctaC	-0.2	2.0	-2.9	1	4	8	0.038735815	1.56E-64	3.94E-168
Rv0810c	hypothetical protein	-0.4	-1.1	-2.9	1	2	8	0.031731132	1.80E-25	3.34E-105
Rv2904c	rplS	0.4	1.0	-2.9	1	2	8	2.05E-05	2.15E-26	1.68E-146
Rv2938	drrC	-0.6	1.8	-2.9	2	3	8	1.66E-08	3.79E-51	2.80E-136
Rv0634B	rpmG2	0.6	1.9	-2.9	2	4	8	2.87E-07	9.37E-50	6.32E-125
Rv0451c	mmpS4	-1.3	2.7	-3.0	3	7	8	1.88E-25	2.08E-143	2.22E-159
Rv0682	rpsL	1.0	0.3	-3.0	2	1	8	5.00E-19	0.001195684	1.01E-136
Rv1830	HTH-type transcriptional regulator	-0.4	1.7	-3.0	1	3	8	0.000155838	1.04E-69	5.82E-168
Rv1177	fdxC	0.1	0.8	-3.0	1	2	8	0.737047434	6.27E-15	7.57E-121
Rv0701	rplC	0.8	1.5	-3.0	2	3	8	1.06E-11	6.53E-56	2.87E-165
Rv2137c	hypothetical protein	-0.7	-1.0	-3.0	2	2	8	8.48E-10	4.56E-14	1.79E-146
Rv2430c	PPE41	-0.4	0.7	-3.0	1	2	8	7.99E-05	1.80E-14	4.47E-167
Rv1435c	hypothetical protein	-0.3	2.7	-3.0	1	7	8	0.052889455	2.21E-116	9.96E-132
Rv3339c	icd1	-0.3	1.0	-3.1	1	2	8	0.012565098	1.08E-20	3.78E-160
Rv2327	hypothetical protein	-0.9	0.9	-3.1	2	2	8	7.92E-09	3.02E-14	1.25E-135
Rv0144	transcriptional regulator	-0.6	2.0	-3.1	1	4	8	2.95E-07	3.60E-95	1.12E-150
Rv3462c	infA	-0.3	0.3	-3.1	1	1	9	0.114479202	0.000759375	2.77E-84
Rv1195	PE13	0.1	1.0	-3.1	1	2	9	0.330668848	1.12E-19	2.75E-131
Rv1502	hypothetical protein	0.2	0.8	-3.1	1	2	9	0.075691168	4.91E-15	1.62E-169
Rv0831c	hypothetical protein	0.1	1.7	-3.1	1	3	9	0.164141471	1.19E-59	2.43E-178
Rv1322	hypothetical protein	0.5	-0.2	-3.1	1	1	9	0.000544931	0.181020905	7.64E-144
Rv1184c	hypothetical protein	-0.3	0.9	-3.1	1	2	9	0.006723831	1.74E-18	9.70E-181
Rv2986c	hupB	-0.2	1.9	-3.2	1	4	9	0.08933871	2.07E-77	9.22E-179
Rv3219	whiB1	-0.4	0.1	-3.2	1	1	9	0.443425429	0.395829495	7.00E-176
Rv0704	rplB	0.4	2.0	-3.2	1	4	9	0.000221574	2.54E-91	8.96E-194
Rv2927c	hypothetical protein	-0.1	1.2	-3.2	1	2	9	0.213503777	6.03E-31	3.45E-166
Rv0710	rpsQ	-0.3	2.4	-3.2	1	5	9	0.023276592	5.10E-94	7.98E-158
Rv1641	infC	0.2	1.1	-3.2	1	2	9	0.090199182	9.82E-29	4.22E-190
Rv2576c	membrane protein	-0.9	-0.8	-3.2	2	2	9	2.34E-09	0.01169482	1.58E-153
Rv1404	transcriptional regulator	-0.5	1.1	-3.2	1	2	9	0.002837649	6.76E-16	1.05E-118
Rv0300	vapB2	-0.4	0.3	-3.2	1	1	9	0.000414867	0.041617276	1.96E-146
Rv0011c	cell division protein CrgA	0.3	0.7	-3.3	1	2	10	0.024419879	7.64E-09	5.21E-131
Rv0719	rplF	0.5	2.2	-3.3	1	5	10	0.000130673	1.39E-95	2.74E-178
Rv2244	acpM	-0.9	2.2	-3.3	2	5	10	1.46E-13	2.02E-76	5.28E-121
Rv0455c	hypothetical protein	-0.6	1.2	-3.3	1	2	10	2.13E-06	4.94E-29	3.03E-155
Rv0078B	hypothetical protein	-0.1	-0.5	-3.3	1	1	10	0.514756159	1.90E-07	5.52E-173
Rv2431c	PE25	-0.1	0.4	-3.3	1	1	10	0.681714397	0.001083089	7.30E-160
Rv1630	rpsA	0.1	1.5	-3.3	1	3	10	0.289370798	3.66E-48	2.25E-215
Rv3583c	RNA polymerase-binding transcriptior	-0.8	-1.0	-3.3	2	2	10	0.050784535	3.18E-20	4.41E-170
Rv3190A	hypothetical protein	0.9	0.5	-3.3	2	1	10	2.37E-07	1.11E-06	4.63E-151
Rv1331	ATP-dependent Clp protease adapter	-0.1	-0.8	-3.3	1	2	10	0.488688892	3.20E-16	1.53E-176

Rv1791	PE19	-0.8	0.2	-3.4	2	1	10	8.03E-13	0.034804049	3.42E-191
Rv0714	rplN	0.6	1.7	-3.4	2	3	10	7.57E-08	3.02E-64	4.05E-170
Rv1211	hypothetical protein	0.6	-0.6	-3.4	1	2	11	0.000534828	1.17E-09	1.78E-197
Rv2941	fadD28	0.7	0.1	-3.4	2	1	11	4.59E-09	0.608353555	4.09E-163
Rv2147c	cell division protein SepF	-0.3	1.1	-3.4	1	2	11	0.004606763	3.51E-28	5.34E-184
Rv2948c	fadD22	-0.1	2.4	-3.4	1	5	11	0.263292085	2.70E-134	2.36E-213
Rv0691A	mycofactocin precursor	-0.5	2.5	-3.5	1	6	11	7.61E-05	3.39E-91	7.99E-140
Rv2846c	efpA	-1.7	2.0	-3.5	3	4	11	1.31E-51	4.90E-78	5.24E-222
Rv0715	rplX	-0.1	2.2	-3.5	1	5	11	0.538988961	7.00E-98	5.86E-195
Rv0821c	phoY2	-0.6	-0.3	-3.5	2	1	11	3.64E-09	0.003940859	6.28E-202
Rv0709	rpmC	-0.5	2.2	-3.5	1	5	11	1.13E-05	2.18E-69	1.19E-186
Rv1846c	blaI	-0.4	0.4	-3.6	1	1	12	0.006596111	3.36E-06	2.82E-228
Rv0896	gltA2	-0.1	1.6	-3.6	1	3	12	0.215220373	1.21E-58	1.85E-238
Rv3173c	TetR/Acr family transcriptional regulat	-1.5	1.5	-3.6	3	3	12	4.13E-21	1.04E-32	4.19E-163
Rv0692	mycofactocin system protein MftB	0.3	2.2	-3.6	1	5	12	0.012263498	1.46E-83	1.69E-153
Rv3854c	ethA	-0.6	1.2	-3.6	2	2	12	4.83E-06	1.27E-28	2.54E-150
Rv0559c	hypothetical protein	-1.1	1.1	-3.7	2	2	13	2.86E-18	1.36E-24	5.86E-230
Rv0659c	mazF2	-0.4	0.4	-3.7	1	1	13	0.000789225	2.60E-05	9.60E-227
Rv0655	mkl	-1.1	2.0	-3.8	2	4	13	1.28E-27	2.82E-101	4.78E-276
Rv1871c	hypothetical protein	-0.9	0.2	-3.8	2	1	14	7.89E-16	0.084174795	2.78E-260
Rv0705	rpsS	0.3	2.2	-3.8	1	5	14	0.010714286	9.08E-82	4.95E-241
Rv1872c	lldD2	-1.1	0.8	-3.8	2	2	14	2.10E-13	6.76E-13	2.24E-287
Rv2950c	fadD29	-0.1	2.1	-3.8	1	4	14	0.509077817	5.16E-108	3.98E-274
Rv0660c	mazE2	-1.0	0.7	-3.9	2	2	14	1.09E-16	5.14E-10	3.36E-195
Rv1925	fadD31	-0.3	-0.5	-3.9	1	1	15	0.003784796	2.78E-06	2.03E-263
Rv3874	esxB	-0.2	0.4	-3.9	1	1	15	0.140147045	0.000151207	1.01E-278
Rv2785c	rpsO	0.4	0.1	-3.9	1	1	15	0.000236709	0.202948198	3.49E-186
Rv1185c	fadD21	0.0	1.2	-3.9	1	2	15	0.683549504	1.83E-36	3.12E-257
Rv1870c	hypothetical protein	0.1	1.0	-3.9	1	2	15	0.316582251	7.30E-22	1.11E-288
Rv3642c	hypothetical protein	-2.2	1.1	-4.1	5	2	17	5.73E-44	5.27E-20	2.06E-187
Rv1094	desA2	-0.5	0.8	-4.1	1	2	17	0.003475608	8.31E-17	2.40E-277
Rv0298	antitoxin	-1.2	1.1	-4.1	2	2	17	8.91E-29	4.94E-27	5.90E-189
Rv2193	ctaE	-0.2	0.6	-4.1	1	1	17	0.132203828	4.64E-09	2.87E-194
Rv1322A	hypothetical protein	-0.5	0.0	-4.1	1	1	18	0.000161333	0.914960853	3.84E-231
Rv3592	heme-degrading monooxygenase	-1.2	1.1	-4.2	2	2	18	3.95E-18	2.27E-15	1.66E-225
Rv2457c	clpX	-0.5	2.2	-4.2	1	5	18	1.35E-05	4.57E-89	2.94E-305
Rv1109c	hypothetical protein	-0.5	2.1	-4.2	1	4	19	8.46E-06	5.19E-105	0
Rv2094c	tatA	-0.7	0.7	-4.2	2	2	19	4.13E-11	6.97E-12	7.66E-296
Rv2949c	chorismate pyruvate-lyase	0.0	2.3	-4.3	1	5	20	0.88830781	2.89E-99	5.67E-294
Rv3489	hypothetical protein	-1.5	0.5	-4.3	3	1	20	7.49E-17	1.77E-05	9.82E-177
Rv3648c	cspA	0.0	1.6	-4.4	1	3	21	0.842316448	7.10E-40	2.14E-279
Rv3841	bfrB	-0.3	-0.4	-4.4	1	1	22	0.558175109	0.000200508	6.85E-269
Rv3408	vapC47	-0.7	0.5	-4.5	2	1	23	1.78E-05	2.37E-06	7.53E-264
Rv0824c	desA1	-0.7	1.6	-4.7	2	3	25	1.28E-08	5.60E-59	0
Rv3407	vapB47	-0.8	0.0	-4.8	2	1	27	3.07E-10	0.826554778	7.11E-285
