# Supplementary material for: Vitamin C potentiates the killing of Mycobacterium tuberculosis by bedaquiline through metabolic disruption
Source: mBio. 2025 Jun 25;16(8):e01484-25. doi: 10.1128/mbio.01484-25 (PMC12345178; doi:10.1128/mbio.01484-25)
Supplement: Table S2 — Primers used in study. [file mbio.01484-25-s0003.pdf]

Table S2. Primers used in this study.

Primer	Sequence
**Cosmid construction**	
Rv0967LL	TTTTTTTTCACAAAGTGTCAGCTTCTTCGGCACGTTC (DraIII)
Rv0967LR	TTTTTTTTCACTTCGTGGGTATGATTCGTAAGTGCTCCGAGTGTCTGGTCTCGTAGGTCAAGATGGCCCCGAACCG (DraIII)
Rv0967RR	TTTTTTTTCCATCTTTTGGCTGGCGAGTACCAGATCACC (Van91I)
Rv0967RL	TTTTTTTTCCATAGATTGGGCGCTAACTGGTGTCATGCTGATGTCTCACTGAGGTCTCTCCGGATGCCAGCAACATGTG (Van91I)
Rv0969RR	TTTTTTTTGCATCTTTTGCTCATCGCTTGGCCTATGCTG (BstAPI)
Rv0969RL	TTTTTTTTGCATAGATTGCTTCCATGCTTTCTGAGACGGCGAGTGTCTGGTCTCGTAGTCTCCTCGGTCAGCGTGGT (BstAPI)
**pYUB3143 construction**	
1169HS1M7A_F	TTTAAGAAGGAGATATACATAATGCACATGGTCAGCGAGC
1169HS1M7A_R	TTCGCCCTTGGAATTCCATATCACTTGTACAGCTCGTCCA

Restriction sites are indicated in brackets.
